# Supplementary material for: SWATH Based Quantitative Proteomics Reveals Significant Lipid Metabolism in Early Myopic Guinea Pig Retina
Source: Int J Mol Sci. 2021 Apr 29;22(9):4721. doi: 10.3390/ijms22094721 (PMC8124159; doi:10.3390/ijms22094721)

Figure S1. FDR analysis of combined IDA library of retina at protein level generated by Protein Pilot software

Protein-Level FDR Analysis

Proteins Identified at Critical False Discovery Rates

Number of Proteins Detected

Critical FDR	Local FDR	*Global FDR*	Global FDR from Fit
1.0%	*2720*	*3183*	**3202**
5.0%	**2979**	*3614*	*3623*
10.0%	**3095**	*4169*	*3981*

** It is recommended you use numbers in bold and avoid using numbers in italics.*

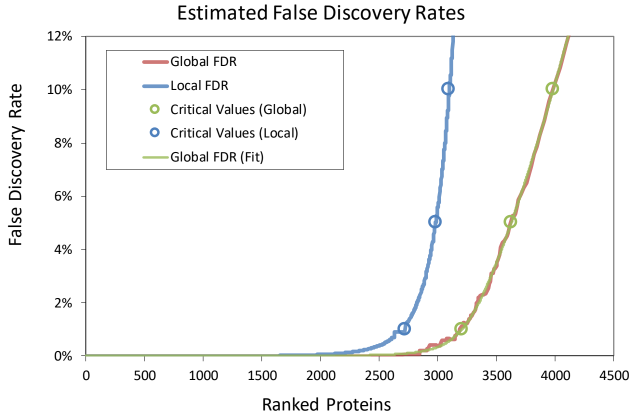

Correspondence between FDR Levels and ProteinPilot Reported Confidences

Corresponding ProteinPilot Confidence

Critical FDR	Local FDR	*Global FDR*	Global FDR from Fit
1.0%	*99.0%*	*85.9%*	**84.9%**
5.0%	**94.5%**	*47.5%*	*46.3%*
10.0%	**90.5%**	*25.9%*	*24.1%*

Corresponding Unused ProtScore

Critical FDR	Local FDR	*Global FDR*	Global FDR from Fit
1.0%	*2.000*	*0.850*	**0.820**
5.0%	**1.260**	*0.280*	*0.270*
10.0%	**1.020**	*0.130*	*0.120*

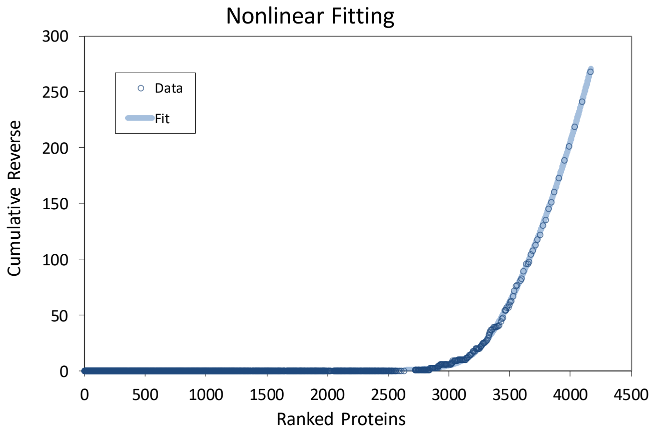

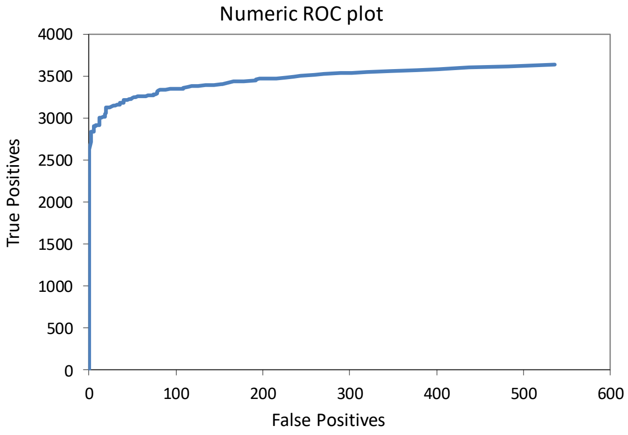

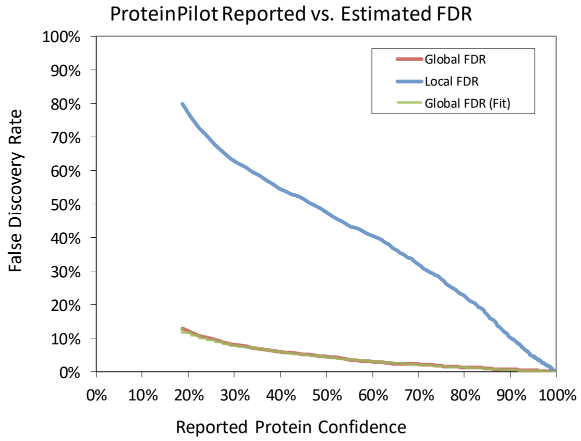

Supplement: Supplementary file 1 [file ijms-22-04721-s001.zip › Figure S1.pdf]
