# Supplementary material for: SWATH Based Quantitative Proteomics Reveals Significant Lipid Metabolism in Early Myopic Guinea Pig Retina
Source: Int J Mol Sci. 2021 Apr 29;22(9):4721. doi: 10.3390/ijms22094721 (PMC8124159; doi:10.3390/ijms22094721)

Figure S2. FDR analysis of combined IDA library of retina at peptide level generated by Protein Pilot software

Distinct Peptide Level FDR Analysis

Peptides Identified at Critical False Discovery Rates

	Number of Peptides Identified		
**Critical FDR**	**Local FDR**	*Global FDR*	**Global FDR from Fit**
**1.0%**	*18216*	*24687*	**24616**
**5.0%**	**23201**	*30618*	*30591*
**10.0%**	**25266**	*36902*	*34576*

** It is recommended you use numbers in bold and avoid using numbers in italics.*

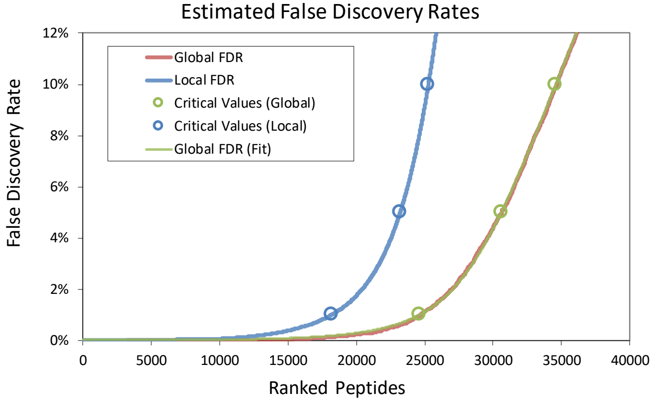

Correspondence between FDR Levels and ProteinPilot Reported Confidences

	Corresponding ProteinPilot Confidence		
**Critical FDR**	**Local FDR**	*Global FDR*	**Global FDR from Fit**
**1.0%**	*99.3%*	*92.7%*	**92.9%**
**5.0%**	**95.9%**	*59.1%*	*59.2%*
**10.0%**	**91.0%**	*37.4%*	*37.8%*

** It is recommended you use numbers in bold and avoid using numbers in italics.*

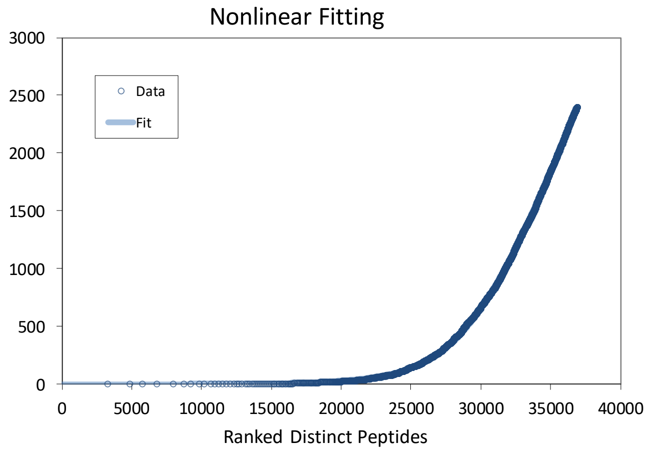

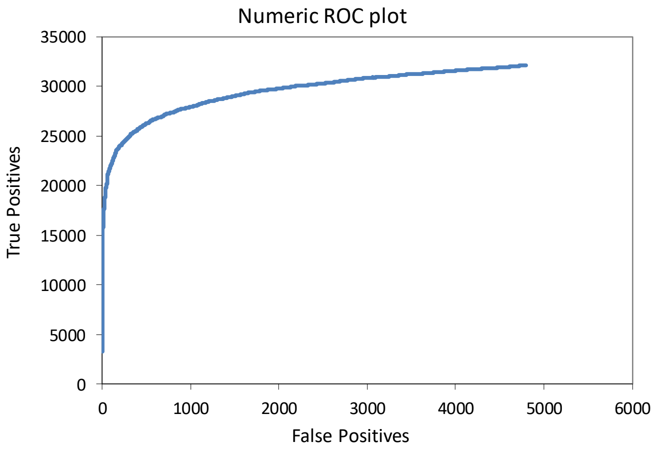

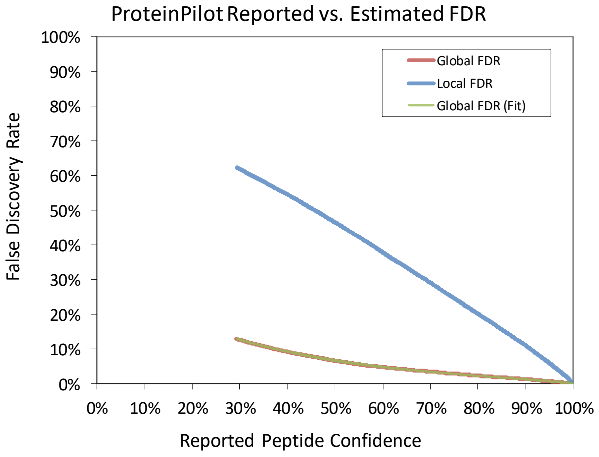

Supplement: Supplementary file 1 [file ijms-22-04721-s001.zip › Figure S2.pdf]
