# Supplementary material for: SWATH Based Quantitative Proteomics Reveals Significant Lipid Metabolism in Early Myopic Guinea Pig Retina
Source: Int J Mol Sci. 2021 Apr 29;22(9):4721. doi: 10.3390/ijms22094721 (PMC8124159; doi:10.3390/ijms22094721)

Figure S3. Network analysis of the top 2,000 identified proteins defined by the STRING database, including 1,947 nodes (proteins) and 57,986 edges (predicted functional associations) retrieved from the retinal proteins in the guinea pigs. The closely related proteins are linked by lines, while different colors represent different types of relationship. Loose proteins are isolated proteins without known linkage.

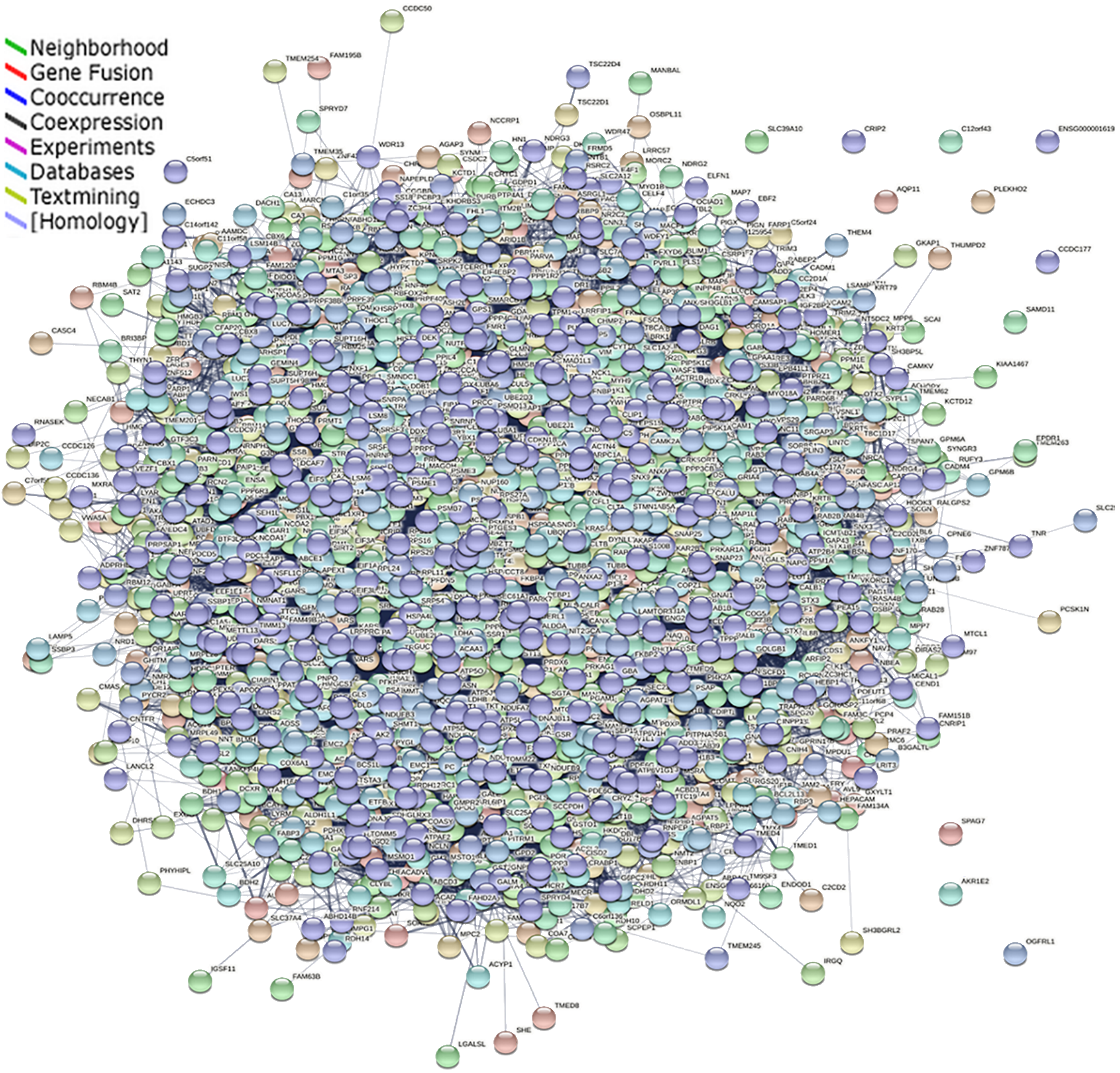

Supplement: Supplementary file 1 [file ijms-22-04721-s001.zip › Figure S3.pdf]
