# Supplementary material for: SWATH Based Quantitative Proteomics Reveals Significant Lipid Metabolism in Early Myopic Guinea Pig Retina
Source: Int J Mol Sci. 2021 Apr 29;22(9):4721. doi: 10.3390/ijms22094721 (PMC8124159; doi:10.3390/ijms22094721)
Supplement: Supplementary file 1 [file ijms-22-04721-s001.zip › Figure S4.pdf]

Figure S4. 25 significant pathways crosstalk with each other by shared common genes generated by Ingenuity Pathway Analysis

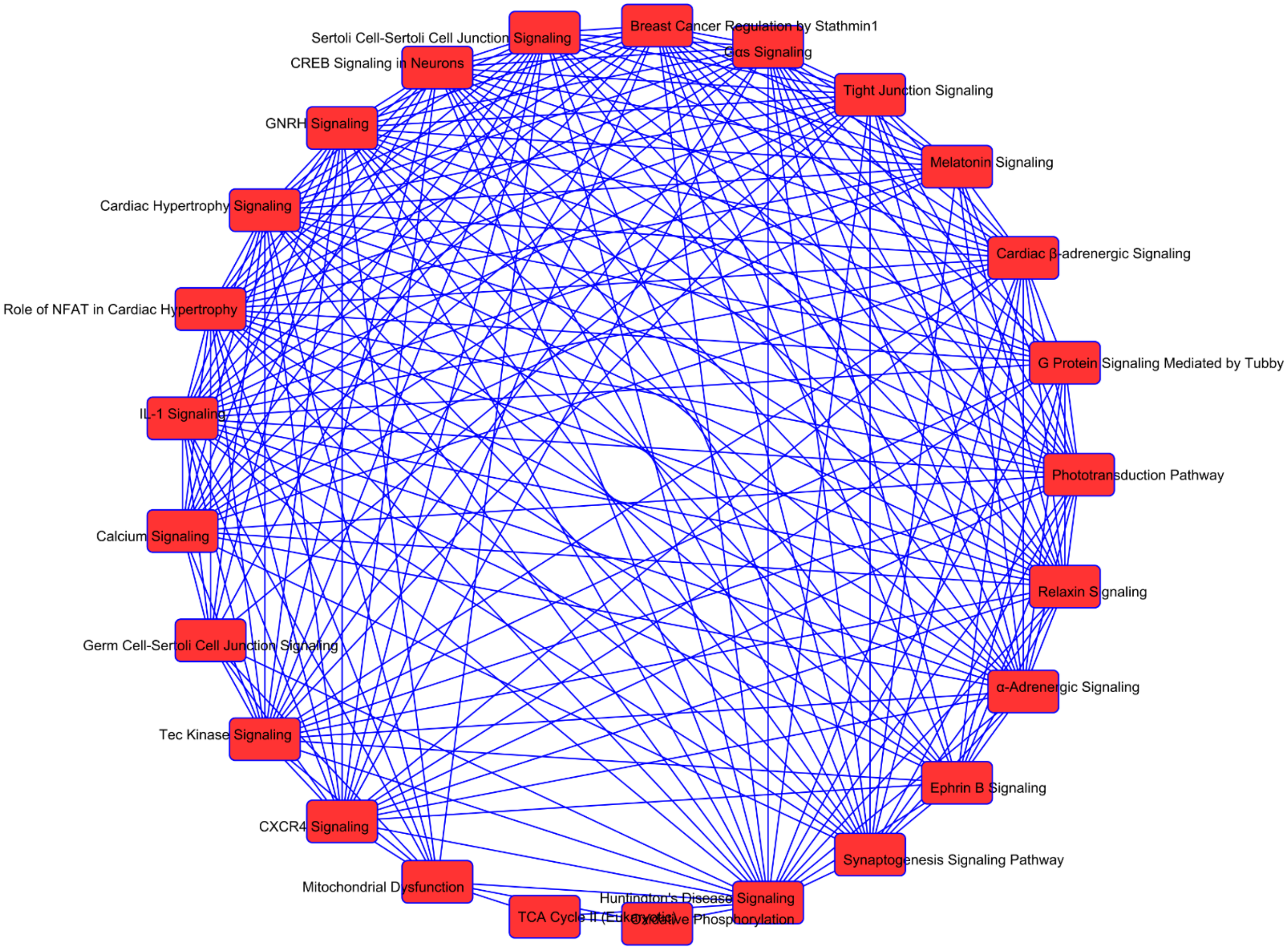

© 2000-2020 QIAGEN. All rights reserved.
