# Supplementary material for: SWATH Based Quantitative Proteomics Reveals Significant Lipid Metabolism in Early Myopic Guinea Pig Retina
Source: Int J Mol Sci. 2021 Apr 29;22(9):4721. doi: 10.3390/ijms22094721 (PMC8124159; doi:10.3390/ijms22094721)
Supplement: Supplementary file 1 [file ijms-22-04721-s001.zip › Table S1.pdf]

Table S1. The full list of 3202 identified unique proteins at global 1% FDR.

No.	Unused Score	Total score	%Cov	%Cov(50)	%Cov(95)	Accession	Name	Species	Peptides(95%)
1	263.3	263	47.22	42.08	33.73	tr|H0V2C0|H0V2C0_CAVPO	Uncharacterized protein OS=Cavia porcellus GN=DYNC1H1 PE=4 SV=1	CAVPO	133
2	229.33	229	64.24	59.55	56.87	tr|H0VEL7|H0VEL7_CAVPO	Uncharacterized protein (Fragment) OS=Cavia porcellus GN=SPTAN1 PE=4 SV=1	CAVPO	127
3	216.89	218	65.08	61.97	56.46	tr|H0VI20|H0VI20_CAVPO	Uncharacterized protein (Fragment) OS=Cavia porcellus GN=SPTBN1 PE=4 SV=1	CAVPO	116
4	179.52	180	58.34	56.71	56.23	tr|H0V795|H0V795_CAVPO	Uncharacterized protein OS=Cavia porcellus GN=MAP1B PE=4 SV=1	CAVPO	127
5	161.37	161	50.7	42.93	42.48	tr|H0VAM7|H0VAM7_CAVPO	Uncharacterized protein OS=Cavia porcellus GN=CROCC PE=4 SV=1	CAVPO	92
6	150.06	150	56.42	54.33	52.3	tr|H0V5M6|H0V5M6_CAVPO	Clathrin heavy chain OS=Cavia porcellus GN=CLTC PE=3 SV=1	CAVPO	109
7	116.68	117	40.83	36.22	31.87	tr|H0VTF1|H0VTF1_CAVPO	Uncharacterized protein (Fragment) OS=Cavia porcellus GN=ABCA4 PE=3 SV=1	CAVPO	63
8	108.95	109	94.8	92.08	90.35	tr|A4L9L4|A4L9L4_CAVPO	S-antigen OS=Cavia porcellus GN=Sag PE=2 SV=1	CAVPO	206
9	107.96	108	45.77	39.35	33.23	tr|H0V2D1|H0V2D1_CAVPO	Uncharacterized protein OS=Cavia porcellus GN=MYH10 PE=4 SV=1	CAVPO	58
10	105.97	106	21.66	14.4	8.301	tr|H0VH85|H0VH85_CAVPO	Uncharacterized protein (Fragment) OS=Cavia porcellus GN=MACF1 PE=4 SV=1	CAVPO	45
11	105.46	105	42.59	35.04	28.85	tr|H0UY83|H0UY83_CAVPO	Uncharacterized protein OS=Cavia porcellus GN=FASN PE=4 SV=1	CAVPO	54
12	105.05	105	54.6	51.4	46.11	tr|H0VJR7|H0VJR7_CAVPO	Uncharacterized protein (Fragment) OS=Cavia porcellus GN=AGL PE=4 SV=1	CAVPO	61

13	102.26	103	82.49	75.89	73.82	tr|H0VDN8|H0VDN8_CAVPO	Pyruvate kinase OS=Cavia porcellus GN=PKM PE=3 SV=1	CAVPO	130
14	96.97	97	75.58	71.21	65.21	tr|H0UXJ8|H0UXJ8_CAVPO	Uncharacterized protein OS=Cavia porcellus GN=HSP90AA1 PE=3 SV=1	CAVPO	90
15	96.78	96.9	44.99	41.73	36.03	tr|H0VTB2|H0VTB2_CAVPO	DNA topoisomerase 2 (Fragment) OS=Cavia porcellus GN=TOP2B PE=3 SV=1	CAVPO	53
16	95.74	95.8	91.78	91.78	89.86	tr|H0W3T8|H0W3T8_CAVPO	Uncharacterized protein (Fragment) OS=Cavia porcellus GN=ALDOC PE=4 SV=1	CAVPO	184
17	94.08	94.1	89.01	87.93	84.91	tr|B5A9S3|B5A9S3_CAVPO	Vimentin OS=Cavia porcellus GN=Vim PE=2 SV=1	CAVPO	141
18	89.57	89.6	36.84	30.38	25.05	tr|H0V8J7|H0V8J7_CAVPO	Uncharacterized protein OS=Cavia porcellus GN=SNRNP200 PE=4 SV=1	CAVPO	44
19	89.07	89.1	37.56	26.98	21.41	tr|H0VGR9|H0VGR9_CAVPO	Uncharacterized protein OS=Cavia porcellus GN=PRPF8 PE=4 SV=1	CAVPO	42
20	87.44	87.4	56.4	55.59	50.23	tr|H0VHR6|H0VHR6_CAVPO	Uncharacterized protein OS=Cavia porcellus GN=ACLY PE=4 SV=1	CAVPO	49
21	87.26	87.3	54.8	49.65	47.18	tr|H0VH73|H0VH73_CAVPO	Uncharacterized protein (Fragment) OS=Cavia porcellus GN=Atp1a3 PE=3 SV=1	CAVPO	60
22	86.07	86.1	82.47	79.78	78.88	tr|H0VKE0|H0VKE0_CAVPO	Uncharacterized protein OS=Cavia porcellus GN=TUBB4B PE=3 SV=1	CAVPO	139
23	85.98	86.7	66.46	58.51	53.92	tr|H0VKT6|H0VKT6_CAVPO	Uncharacterized protein OS=Cavia porcellus GN=EPB41L2 PE=4 SV=1	CAVPO	59
24	84.88	85	88.59	87.99	87.99	tr|B5AN23|B5AN23_CAVPO	Glyceraldehyde 3-phosphate dehydrogenase OS=Cavia porcellus GN=Gapdh PE=2 SV=1	CAVPO	208
25	83.53	83.7	37.14	26.67	21.4	tr|H0V7C5|H0V7C5_CAVPO	Uncharacterized protein OS=Cavia porcellus GN=TLN2 PE=4 SV=1	CAVPO	39
26	83.46	83.6	94.93	92.4	89.63	tr|A9YWS9|A9YWS9_CAVPO	Alpha-enolase OS=Cavia porcellus GN=Eno1 PE=2 SV=1	CAVPO	175

27	82.48	82.5	67.07	58.4	56.42	tr|H0VK93|H0VK93_CAVPO	Uncharacterized protein OS=Cavia porcellus GN=ACTN4 PE=4 SV=1	CAVPO	48
28	81.83	81.8	52.69	52.58	49.18	tr|H0W2R8|H0W2R8_CAVPO	Uncharacterized protein OS=Cavia porcellus GN=PDE6B PE=4 SV=1	CAVPO	46
29	80.28	80.3	60.37	58.44	57.86	tr|H0V091|H0V091_CAVPO	Reticulon (Fragment) OS=Cavia porcellus GN=RTN4 PE=4 SV=1	CAVPO	49
30	78.99	79	93.35	92.46	92.24	tr|H0W9T4|H0W9T4_CAVPO	Uncharacterized protein (Fragment) OS=Cavia porcellus GN=TUBA1B PE=3 SV=1	CAVPO	142
31	77.54	77.6	38.8	30.41	27.76	tr|H0VDA8|H0VDA8_CAVPO	Uncharacterized protein (Fragment) OS=Cavia porcellus GN=NUMA1 PE=4 SV=1	CAVPO	43
32	76.68	76.7	46.68	37.26	32.78	tr|H0VCT0|H0VCT0_CAVPO	Microtubule-associated protein OS=Cavia porcellus GN=MAP2 PE=3 SV=1	CAVPO	48
33	75.97	76	54.54	52.08	48.11	tr|H0UZR6|H0UZR6_CAVPO	Uncharacterized protein OS=Cavia porcellus GN=UBA1 PE=3 SV=1	CAVPO	49
34	75.36	75.4	41.95	38.37	36.5	tr|H0UX20|H0UX20_CAVPO	Uncharacterized protein OS=Cavia porcellus GN=CAND1 PE=4 SV=1	CAVPO	40
35	75.24	75.2	52.37	47.52	42.99	tr|H0V6T6|H0V6T6_CAVPO	Uncharacterized protein (Fragment) OS=Cavia porcellus GN=HK1 PE=3 SV=1	CAVPO	44
36	74.49	74.5	54.07	50.12	49.07	tr|H0VZJ1|H0VZJ1_CAVPO	Uncharacterized protein OS=Cavia porcellus GN=DNM1 PE=3 SV=1	CAVPO	39
37	71.87	71.9	65.13	64.62	62.18	tr|H0VLW4|H0VLW4_CAVPO	Uncharacterized protein OS=Cavia porcellus GN=ACO2 PE=4 SV=1	CAVPO	42
38	71.62	71.7	51.61	48.69	45.97	tr|H0UWJ0|H0UWJ0_CAVPO	Uncharacterized protein OS=Cavia porcellus GN=TF PE=4 SV=1	CAVPO	36
39	71.49	71.5	76.17	72.81	72.81	tr|H0VBB2|H0VBB2_CAVPO	Uncharacterized protein OS=Cavia porcellus GN=DPYSL3 PE=4 SV=1	CAVPO	60
40	71.06	71.1	65.14	61.04	59.93	tr|H0VKG1|H0VKG1_CAVPO	Uncharacterized protein (Fragment) OS=Cavia porcellus GN=VCP PE=3 SV=1	CAVPO	41

41	69.97	70	46.74	41.16	37.71	tr|H0UWT8|H0UWT8_CAVPO	Uncharacterized protein OS=Cavia porcellus GN=CADPS PE=4 SV=1	CAVPO	37
42	69.94	70	78.95	77.99	72.73	tr|H0VYU4|H0VYU4_CAVPO	Phosphoglycerate kinase OS=Cavia porcellus GN=PGK1 PE=3 SV=1	CAVPO	75
43	69.84	69.9	59.46	58.39	55.08	tr|H0VQ72|H0VQ72_CAVPO	Uncharacterized protein OS=Cavia porcellus GN=MATR3 PE=4 SV=1	CAVPO	44
44	69.61	69.6	53.86	51.96	51.25	tr|H0WCW9|H0WCW9_CAVPO	Phosphorylase OS=Cavia porcellus GN=PYGB PE=3 SV=1	CAVPO	39
45	69.1	69.1	60.79	59.95	58.52	tr|H0V2D0|H0V2D0_CAVPO	Uncharacterized protein OS=Cavia porcellus GN=HSPA4 PE=3 SV=1	CAVPO	40
46	67.73	67.7	72.84	68.62	62.94	tr|H0VDX5|H0VDX5_CAVPO	Uncharacterized protein OS=Cavia porcellus GN=CCT3 PE=3 SV=1	CAVPO	40
47	67.49	67.9	41.27	36.24	31.28	tr|H0V178|H0V178_CAVPO	Uncharacterized protein OS=Cavia porcellus GN=EPRS PE=3 SV=1	CAVPO	35
48	66.93	66.9	43.67	38.58	36.08	tr|H0UZ89|H0UZ89_CAVPO	Calcium-transporting ATPase OS=Cavia porcellus GN=ATP2A2 PE=3 SV=1	CAVPO	35
49	66.64	66.7	64.51	63.52	60.86	tr|H0V1C3|H0V1C3_CAVPO	Uncharacterized protein OS=Cavia porcellus GN=STXBP1 PE=4 SV=1	CAVPO	45
50	66.28	66.3	59.39	55.27	54.2	tr|H0VLM3|H0VLM3_CAVPO	Uncharacterized protein (Fragment) OS=Cavia porcellus GN=HSPA5 PE=3 SV=1	CAVPO	45
51	65.87	65.9	62.39	60.88	59.7	tr|H0V0G1|H0V0G1_CAVPO	Uncharacterized protein OS=Cavia porcellus GN=CANX PE=3 SV=1	CAVPO	43
52	65.41	65.5	42.46	40.61	35.09	tr|H0VFS4|H0VFS4_CAVPO	Uncharacterized protein OS=Cavia porcellus GN=DDB1 PE=4 SV=1	CAVPO	37
53	63.94	63.9	54.21	50.93	48.71	tr|H0V823|H0V823_CAVPO	Uncharacterized protein (Fragment) OS=Cavia porcellus GN=NPEPPS PE=4 SV=1	CAVPO	35
54	63.32	63.3	51	42.9	40.9	tr|H0VGF4|H0VGF4_CAVPO	Uncharacterized protein OS=Cavia porcellus GN=AP2B1 PE=4 SV=1	CAVPO	35

55	63.2	63.2	46.74	42.8	41.3	tr|H0VIB5|H0VIB5_CAVPO	Uncharacterized protein OS=Cavia porcellus GN=AP2A2 PE=4 SV=1	CAVPO	36
56	62.44	62.5	47.56	39.96	38.07	tr|H0UWB3|H0UWB3_CAVPO	Guanylate cyclase OS=Cavia porcellus GN=GUCY2D PE=3 SV=1	CAVPO	35
57	61.7	61.7	54.25	53.9	50.95	tr|H0VEJ3|H0VEJ3_CAVPO	Uncharacterized protein OS=Cavia porcellus GN=LMNB2 PE=3 SV=1	CAVPO	36
58	61.64	61.6	52.8	49.44	47.57	tr|H0VK81|H0VK81_CAVPO	Uncharacterized protein OS=Cavia porcellus GN=HNRNPU PE=4 SV=1	CAVPO	50
59	61.58	61.6	81.94	80.39	77.48	tr|H0W6W1|H0W6W1_CAVPO	Glucose-6-phosphate 1-dehydrogenase OS=Cavia porcellus GN=G6PD PE=3 SV=1	CAVPO	39
60	61.52	61.5	63.21	59.32	57.21	tr|H0VLR3|H0VLR3_CAVPO	Uncharacterized protein OS=Cavia porcellus GN=ATP6V1A PE=3 SV=1	CAVPO	37
61	61.42	61.5	46.84	44.19	39.65	tr|H0V6R2|H0V6R2_CAVPO	Uncharacterized protein (Fragment) OS=Cavia porcellus GN=ATP6AP1 PE=4 SV=1	CAVPO	38
62	61.08	66.1	65.63	64.24	60.53	tr|H0V2J1|H0V2J1_CAVPO	Uncharacterized protein OS=Cavia porcellus GN=Hspa8 PE=3 SV=1	CAVPO	73
63	60.66	60.7	52.22	49.36	47.64	tr|H0VTY8|H0VTY8_CAVPO	Uncharacterized protein (Fragment) OS=Cavia porcellus GN=EEF2 PE=4 SV=1	CAVPO	37
64	60.25	60.4	32.7	25.1	14.78	tr|H0UZ34|H0UZ34_CAVPO	Uncharacterized protein OS=Cavia porcellus GN=FLNA PE=4 SV=1	CAVPO	26
65	60.02	60.1	86.88	86.61	82.94	tr|H0VEV2|H0VEV2_CAVPO	Uncharacterized protein OS=Cavia porcellus GN=CKB PE=3 SV=1	CAVPO	87
66	59.21	74.6	53.02	50.93	45.12	tr|H0UXF3|H0UXF3_CAVPO	Uncharacterized protein OS=Cavia porcellus GN=PDE6A PE=4 SV=1	CAVPO	43
67	59.14	59.2	47.07	43.96	41.84	tr|H0VC90|H0VC90_CAVPO	Uncharacterized protein OS=Cavia porcellus GN=HSP90B1 PE=3 SV=1	CAVPO	33
68	59.12	59.2	50.1	46.69	38.33	tr|H0UYE7|H0UYE7_CAVPO	Alanine--tRNA ligase, cytoplasmic OS=Cavia porcellus GN=AARS PE=3 SV=1	CAVPO	31

69	59.12	59.2	50.1	46.69	38.33	tr|H0UYE7|H0UYE7_CAVPO	Alanine--tRNA ligase, cytoplasmic OS=Cavia porcellus GN=AARS PE=3 SV=1	CAVPO	31
70	59.04	59.1	90.83	81.66	77.51	tr|H0VKB0|H0VKB0_CAVPO	L-lactate dehydrogenase (Fragment) OS=Cavia porcellus GN=LDHA PE=3 SV=1	CAVPO	78
71	58.86	58.9	80	78.4	75.47	tr|H0VKP1|H0VKP1_CAVPO	Uncharacterized protein OS=Cavia porcellus GN=ACTG1 PE=3 SV=1	CAVPO	86
72	58.18	58.2	65.11	63.31	60.25	tr|H0UWR7|H0UWR7_CAVPO	T-complex protein 1 subunit alpha OS=Cavia porcellus GN=LOC100714101 PE=3 SV=1	CAVPO	34
73	57.72	57.9	28.84	22.46	16.12	tr|H0WA32|H0WA32_CAVPO	Uncharacterized protein OS=Cavia porcellus GN=TPR PE=4 SV=1	CAVPO	30
74	57.67	58.3	64.36	63.09	60.73	tr|H0VEJ5|H0VEJ5_CAVPO	Uncharacterized protein (Fragment) OS=Cavia porcellus GN=CCT8 PE=3 SV=1	CAVPO	34
75	57.34	68	52.41	45.12	42.19	tr|H0V733|H0V733_CAVPO	Phosphorylase OS=Cavia porcellus GN=PYGL PE=3 SV=1	CAVPO	37
76	57.19	57.4	49.8	42.65	42.51	tr|H0VEI7|H0VEI7_CAVPO	Uncharacterized protein (Fragment) OS=Cavia porcellus GN=Nsf PE=3 SV=1	CAVPO	36
77	57.16	57.2	43.55	43.55	42.49	tr|H0V3P6|H0V3P6_CAVPO	Uncharacterized protein (Fragment) OS=Cavia porcellus GN=NCAM1 PE=4 SV=1	CAVPO	42
78	56.97	61.3	57.38	53.18	49.46	tr|H0V8F4|H0V8F4_CAVPO	Uncharacterized protein (Fragment) OS=Cavia porcellus GN=HSPA4L PE=3 SV=1	CAVPO	37
79	56.36	56.5	43.09	37.31	33.57	tr|H0VM20|H0VM20_CAVPO	Uncharacterized protein OS=Cavia porcellus GN=DCTN1 PE=4 SV=1	CAVPO	32
80	56.33	56.4	43.2	40.91	37.8	tr|H0V1E2|H0V1E2_CAVPO	Uncharacterized protein OS=Cavia porcellus GN=GANAB PE=4 SV=1	CAVPO	32
81	56.18	56.2	51.52	48.23	46.59	tr|H0UW67|H0UW67_CAVPO	6-phosphofructokinase OS=Cavia porcellus GN=PFKP PE=3 SV=1	CAVPO	34
82	56.09	56.6	58.41	56.56	55.08	tr|H0UXK6|H0UXK6_CAVPO	Uncharacterized protein OS=Cavia porcellus GN=CCT5 PE=3 SV=1	CAVPO	31

83	55.95	66.5	44.74	40.09	38.77	tr|H0V335|H0V335_CAVPO	Uncharacterized protein OS=Cavia porcellus GN=EPB41L3 PE=4 SV=1	CAVPO	38
84	55.83	55.9	66.33	65.49	55.11	tr|H0UT42|H0UT42_CAVPO	Uncharacterized protein (Fragment) OS=Cavia porcellus GN=TKT PE=4 SV=1	CAVPO	38
85	55.36	57.4	65.61	54.99	54.99	tr|H0VNL4|H0VNL4_CAVPO	Uncharacterized protein (Fragment) OS=Cavia porcellus GN=LMNB1 PE=3 SV=1	CAVPO	41
86	55.12	55.3	34.67	32.46	27.46	tr|H0VTK1|H0VTK1_CAVPO	Calcium-transporting ATPase OS=Cavia porcellus GN=ATP2B1 PE=3 SV=1	CAVPO	28
87	55.06	55.1	67.33	65.54	65.54	tr|H0VBS1|H0VBS1_CAVPO	Protein disulfide-isomerase OS=Cavia porcellus GN=PDIA3 PE=3 SV=1	CAVPO	35
88	54.8	54.9	56.99	53.41	51.97	tr|H0VCM3|H0VCM3_CAVPO	Glucose-6-phosphate isomerase OS=Cavia porcellus GN=GPI PE=3 SV=1	CAVPO	59
89	54.63	54.7	66.17	62.8	59.25	tr|H0VMF2|H0VMF2_CAVPO	Uncharacterized protein (Fragment) OS=Cavia porcellus GN=CCT2 PE=3 SV=1	CAVPO	28
90	54.33	54.4	47.74	46.5	45.13	tr|H0VK01|H0VK01_CAVPO	Uncharacterized protein (Fragment) OS=Cavia porcellus GN=DDX17 PE=3 SV=1	CAVPO	33
91	53.78	53.8	43.11	38.81	32.45	tr|H0V0P6|H0V0P6_CAVPO	Uncharacterized protein OS=Cavia porcellus GN=OGDH PE=4 SV=1	CAVPO	29
92	53.3	53.7	26.67	21.93	16.97	tr|H0VB36|H0VB36_CAVPO	Uncharacterized protein (Fragment) OS=Cavia porcellus GN=MYO5A PE=4 SV=1	CAVPO	27
93	52.77	52.8	64.22	58.76	57.44	tr|H0VZD9|H0VZD9_CAVPO	ATP synthase subunit beta OS=Cavia porcellus GN=ATP5B PE=3 SV=1	CAVPO	58
94	52.24	52.3	67.78	65.03	60.51	tr|H0VL04|H0VL04_CAVPO	Protein disulfide-isomerase OS=Cavia porcellus GN=P4HB PE=3 SV=1	CAVPO	31
95	52.13	52.2	36.91	34.44	25.41	tr|H0VEF4|H0VEF4_CAVPO	Uncharacterized protein (Fragment) OS=Cavia porcellus GN=SF3B1 PE=4 SV=1	CAVPO	28
96	51.73	51.7	47.79	46.69	45.72	tr|H0V5Q8|H0V5Q8_CAVPO	Uncharacterized protein OS=Cavia porcellus GN=NDUFS1 PE=4 SV=1	CAVPO	28

97	51.61	54	31.68	24.42	16.63	tr|H0W917|H0W917_CAVPO	Uncharacterized protein (Fragment) OS=Cavia porcellus GN=MAP1A PE=4 SV=1	CAVPO	25
98	51.42	51.4	38.85	28.44	27.58	tr|H0V943|H0V943_CAVPO	Uncharacterized protein OS=Cavia porcellus GN=MYO6 PE=4 SV=1	CAVPO	30
99	50.52	50.6	78.82	77.35	74.41	tr|A9LS48|A9LS48_CAVPO	Beta-transducin OS=Cavia porcellus GN=Gnb1 PE=2 SV=1	CAVPO	71
100	50.41	50.4	40.7	37.96	33.46	tr|H0VD65|H0VD65_CAVPO	Uncharacterized protein OS=Cavia porcellus GN=CNTN1 PE=4 SV=1	CAVPO	27
101	49.77	49.8	68.4	68.2	55.4	tr|H0V3J6|H0V3J6_CAVPO	Uncharacterized protein OS=Cavia porcellus GN=DARS PE=3 SV=1	CAVPO	25
102	49.69	49.7	40.45	34.64	34.64	tr|H0VPE3|H0VPE3_CAVPO	Uncharacterized protein (Fragment) OS=Cavia porcellus GN=ILF3 PE=4 SV=1	CAVPO	28
103	49.64	49.7	35.74	31.04	25.2	tr|H0VEX7|H0VEX7_CAVPO	Structural maintenance of chromosomes protein OS=Cavia porcellus GN=SMC1A PE=3 SV=1	CAVPO	26
104	49.6	49.7	41.08	32.46	25.88	tr|H0VDK8|H0VDK8_CAVPO	Uncharacterized protein OS=Cavia porcellus GN=SMC3 PE=4 SV=1	CAVPO	25
105	49.54	49.6	53.45	53.09	50.36	tr|H0VM26|H0VM26_CAVPO	ATP synthase subunit alpha OS=Cavia porcellus GN=ATP5A1 PE=3 SV=1	CAVPO	38
106	49.51	50.1	41.39	40.36	39.57	tr|H0V0D8|H0V0D8_CAVPO	Uncharacterized protein OS=Cavia porcellus GN=PDCD6IP PE=4 SV=1	CAVPO	26
107	49.31	62.3	33.12	28.05	19.1	tr|H0UY16|H0UY16_CAVPO	Uncharacterized protein (Fragment) OS=Cavia porcellus GN=MYH14 PE=4 SV=1	CAVPO	31
108	49.3	49.3	61.21	61.03	59.61	tr|H0VCN9|H0VCN9_CAVPO	Uncharacterized protein (Fragment) OS=Cavia porcellus PE=4 SV=1	CAVPO	32
109	49.16	56.3	63.99	59.62	57.87	tr|H0VF18|H0VF18_CAVPO	Uncharacterized protein OS=Cavia porcellus GN=CRMP1 PE=4 SV=1	CAVPO	42
110	48.73	49	60.92	55.6	51.15	tr|H0W1Z7|H0W1Z7_CAVPO	Uncharacterized protein OS=Cavia porcellus	CAVPO	33

							GN=HSPD1 PE=3 SV=1		
111	48.57	80.9	49.61	46.19	44.63	tr|B3SI06|B3SI06_CAVPO	Sodium/potassium-ATPase alpha-subunit isoform 1 splice-variant a OS=Cavia porcellus GN=atp1a1 PE=2 SV=2	CAVPO	53
112	48.44	48.6	31.9	28.03	20	tr|H0UTE6|H0UTE6_CAVPO	Uncharacterized protein OS=Cavia porcellus GN=LRPPRC PE=4 SV=1	CAVPO	25
113	48.3	48.3	73.12	67.55	66.1	tr|H0VCS7|H0VCS7_CAVPO	Aspartate aminotransferase OS=Cavia porcellus GN=GOT1 PE=3 SV=1	CAVPO	30
114	48.21	56.2	30.48	21.56	15.43	tr|H0W0A9|H0W0A9_CAVPO	Uncharacterized protein (Fragment) OS=Cavia porcellus GN=SPTB PE=4 SV=1	CAVPO	28
115	48.08	52.4	52.5	46.68	39.7	tr|H0V9N5|H0V9N5_CAVPO	Uncharacterized protein OS=Cavia porcellus GN=EPB41 PE=4 SV=1	CAVPO	28
116	48.01	48.1	44.23	36.51	33.8	tr|H0V7T7|H0V7T7_CAVPO	Uncharacterized protein OS=Cavia porcellus GN=HYOU1 PE=3 SV=1	CAVPO	28
117	47.93	48	53.59	49.56	47.99	tr|H0V7E1|H0V7E1_CAVPO	Inosine-5'-monophosphate dehydrogenase OS=Cavia porcellus GN=IMPDH1 PE=3 SV=1	CAVPO	28
118	47.89	47.9	54.99	52.45	49.58	tr|H0VSR9|H0VSR9_CAVPO	Uncharacterized protein (Fragment) OS=Cavia porcellus PE=4 SV=1	CAVPO	31
119	47.87	49.7	76.61	66.14	63	tr|H0VS82|H0VS82_CAVPO	Uncharacterized protein OS=Cavia porcellus GN=DPYSL4 PE=4 SV=1	CAVPO	36
120	47.81	47.9	37.98	30.66	27.76	tr|H0V2B7|H0V2B7_CAVPO	Uncharacterized protein OS=Cavia porcellus GN=GTF2I PE=4 SV=1	CAVPO	25
121	47.53	47.6	55.24	50.23	43.82	tr|H0VFG8|H0VFG8_CAVPO	Uncharacterized protein (Fragment) OS=Cavia porcellus GN=DDX1 PE=4 SV=1	CAVPO	25
122	47.41	48.5	43.03	35.51	29.18	tr|H0VTU7|H0VTU7_CAVPO	Uncharacterized protein OS=Cavia porcellus GN=Parp1 PE=4 SV=1	CAVPO	25
123	47.22	47.3	44.04	35.74	33.66	tr|H0VBZ6|H0VBZ6_CAVPO	Uncharacterized protein (Fragment) OS=Cavia porcellus GN=KIF5B PE=4 SV=1	CAVPO	25

124	46.92	47	64.77	64.58	58.12	tr|H0UWY1|H0UWY1_CAVPO	Uncharacterized protein OS=Cavia porcellus GN=ATP6V1B2 PE=3 SV=1	CAVPO	28
125	46.4	46.4	70.5	68.82	68.59	tr|H0UUR0|H0UUR0_CAVPO	Uncharacterized protein OS=Cavia porcellus GN=CALR PE=3 SV=1	CAVPO	32
126	46.16	46.2	59.11	58.21	55.89	tr|H0VBV7|H0VBV7_CAVPO	Uncharacterized protein OS=Cavia porcellus GN=DPYSL5 PE=4 SV=1	CAVPO	28
127	46.04	46.1	22.06	16.12	12.66	tr|H0VF88|H0VF88_CAVPO	Uncharacterized protein OS=Cavia porcellus GN=SRRM2 PE=4 SV=1	CAVPO	24
128	45.85	45.9	51.39	46.21	43.99	tr|H0UZM9|H0UZM9_CAVPO	Uncharacterized protein (Fragment) OS=Cavia porcellus GN=STIP1 PE=4 SV=1	CAVPO	29
129	45.68	45.7	31.21	31.21	27.97	tr|H0VSJ5|H0VSJ5_CAVPO	Uncharacterized protein (Fragment) OS=Cavia porcellus GN=SLC12A5 PE=4 SV=1	CAVPO	23
130	45.34	45.4	44.58	44.58	40.42	tr|H0VRX2|H0VRX2_CAVPO	Uncharacterized protein (Fragment) OS=Cavia porcellus GN=SLC25A12 PE=3 SV=1	CAVPO	25
131	45.25	53.4	44.42	38.02	33.49	tr|H0V2T6|H0V2T6_CAVPO	Uncharacterized protein (Fragment) OS=Cavia porcellus GN=PDE6C PE=4 SV=1	CAVPO	31
132	45.07	45.1	30.68	24.91	24.91	tr|H0W5M5|H0W5M5_CAVPO	Uncharacterized protein OS=Cavia porcellus GN=ACIN1 PE=4 SV=1	CAVPO	26
133	45.03	45.1	47.46	44.03	40.92	tr|H0VF40|H0VF40_CAVPO	Uncharacterized protein OS=Cavia porcellus GN=Hnrnpr PE=4 SV=1	CAVPO	25
134	44.92	44.9	77.19	74.74	70.88	tr|H0VL82|H0VL82_CAVPO	Triosephosphate isomerase OS=Cavia porcellus GN=TPI1 PE=3 SV=1	CAVPO	75
135	44.85	44.9	43.66	40.08	38.28	tr|H0UZN6|H0UZN6_CAVPO	Uncharacterized protein (Fragment) OS=Cavia porcellus GN=CTNNB1 PE=4 SV=1	CAVPO	23
136	44.61	44.6	51.24	45.39	44.33	tr|H0VNV0|H0VNV0_CAVPO	Uncharacterized protein OS=Cavia porcellus GN=GRK1 PE=3 SV=1	CAVPO	25
137	44.5	44.8	35.13	30.15	22.88	tr|H0VS53|H0VS53_CAVPO	Coatomer subunit alpha OS=Cavia porcellus GN=COPA PE=3 SV=1	CAVPO	22

138	43.43	43.4	58.57	57.66	52.35	tr|H0W6C9|H0W6C9_CAVPO	Uncharacterized protein OS=Cavia porcellus GN=Fam169a PE=4 SV=1	CAVPO	41
139	43.21	43.2	69.68	69.44	68.06	tr|H0VE02|H0VE02_CAVPO	Aspartate aminotransferase OS=Cavia porcellus GN=GOT2 PE=3 SV=1	CAVPO	39
140	43.15	43.2	50.88	45.58	41.74	tr|H0UYD9|H0UYD9_CAVPO	Uncharacterized protein OS=Cavia porcellus GN=HSPA9 PE=3 SV=1	CAVPO	35
141	42.77	42.8	59.74	56.39	45.21	tr|H0V4S0|H0V4S0_CAVPO	Uncharacterized protein OS=Cavia porcellus GN=KHSRP PE=4 SV=1	CAVPO	30
142	42.35	42.4	52.41	49.31	48.28	tr|H0VZZ5|H0VZZ5_CAVPO	Uncharacterized protein OS=Cavia porcellus GN=PGM1 PE=3 SV=1	CAVPO	27
143	42.13	42.2	40.38	36.6	27.17	tr|H0V7U8|H0V7U8_CAVPO	Uncharacterized protein OS=Cavia porcellus GN=DHX15 PE=4 SV=1	CAVPO	21
144	42.09	42.1	54.5	50.08	47.3	tr|H0VMU9|H0VMU9_CAVPO	Leukotriene A(4) hydrolase OS=Cavia porcellus GN=Lta4h PE=3 SV=1	CAVPO	25
145	41.93	42	55.29	53.62	52.32	tr|H0W4A6|H0W4A6_CAVPO	Uncharacterized protein (Fragment) OS=Cavia porcellus GN=CCT7 PE=3 SV=1	CAVPO	25
146	41.88	41.9	44.41	38.51	38.51	tr|H0V2B9|H0V2B9_CAVPO	Protein disulfide-isomerase A4 OS=Cavia porcellus GN=PDIA4 PE=3 SV=1	CAVPO	25
147	41.83	41.9	34.98	32.45	29.45	tr|H0VJ49|H0VJ49_CAVPO	Uncharacterized protein OS=Cavia porcellus GN=ATP6V0A1 PE=4 SV=1	CAVPO	23
148	41.66	54.3	66.08	65.2	63.09	tr|H0W639|H0W639_CAVPO	Uncharacterized protein OS=Cavia porcellus GN=DPYSL2 PE=4 SV=1	CAVPO	53
149	41.62	41.7	55.85	50.25	47.28	tr|H0V2D5|H0V2D5_CAVPO	Dolichyl-diphosphooligosaccharide--protein glycosyltransferase subunit 1 OS=Cavia porcellus GN=RPN1 PE=3 SV=1	CAVPO	25
150	41.49	41.6	46.59	40.4	27.49	tr|H0VSV2|H0VSV2_CAVPO	Coatomer subunit beta OS=Cavia porcellus GN=COPB1 PE=3 SV=1	CAVPO	20
151	41.21	41.3	39.38	36.87	30.59	tr|H0V830|H0V830_CAVPO	Uncharacterized protein OS=Cavia porcellus	CAVPO	21

							GN=KPNB1 PE=4 SV=1		
152	41.18	41.3	48.29	41.21	40.16	tr|H0V6P1|H0V6P1_CAVPO	Uncharacterized protein OS=Cavia porcellus GN=HADHA PE=3 SV=1	CAVPO	22
153	41.18	60.7	87.09	85.44	81.04	tr|H0VA39|H0VA39_CAVPO	Uncharacterized protein OS=Cavia porcellus GN=ALDOA PE=3 SV=1	CAVPO	85
154	41.16	41.6	36.71	28.24	23.69	tr|H0VRS6|H0VRS6_CAVPO	Uncharacterized protein OS=Cavia porcellus PE=4 SV=1	CAVPO	23
155	40.85	40.9	53.69	52.96	51.48	tr|H0V7W3|H0V7W3_CAVPO	Uncharacterized protein OS=Cavia porcellus GN=EIF4A3 PE=3 SV=1	CAVPO	24
156	40.59	40.7	51.94	46.83	39.44	tr|H0UUL3|H0UUL3_CAVPO	Uncharacterized protein (Fragment) OS=Cavia porcellus GN=HNRNPUL2 PE=4 SV=1	CAVPO	22
157	40.43	40.5	76.68	71.85	68.9	tr|H0VEQ9|H0VEQ9_CAVPO	Glutamine synthetase OS=Cavia porcellus GN=GLUL PE=3 SV=1	CAVPO	68
158	40.38	40.5	36.08	28.28	25.18	tr|H0VY86|H0VY86_CAVPO	Uncharacterized protein OS=Cavia porcellus GN=UPF1 PE=4 SV=1	CAVPO	23
159	40.28	54.8	45.26	44.06	35.01	tr|H0VE10|H0VE10_CAVPO	Uncharacterized protein OS=Cavia porcellus GN=HK2 PE=3 SV=1	CAVPO	28
160	40.24	40.3	74	72	68.29	tr|A9LS49|A9LS49_CAVPO	Alpha-transducin OS=Cavia porcellus GN=Gnat1 PE=2 SV=1	CAVPO	50
161	40.23	40.3	35.83	29.9	29.56	tr|H0VE17|H0VE17_CAVPO	Uncharacterized protein (Fragment) OS=Cavia porcellus GN=PSMD2 PE=4 SV=1	CAVPO	20
162	40.08	40.2	31.82	27.27	22.81	tr|H0UU89|H0UU89_CAVPO	Uncharacterized protein OS=Cavia porcellus GN=SF3B3 PE=4 SV=1	CAVPO	20
163	40.07	40.3	57.25	54.24	53.11	tr|H0V927|H0V927_CAVPO	Uncharacterized protein OS=Cavia porcellus GN=CCT6A PE=3 SV=1	CAVPO	25
164	39.76	39.9	46.68	44.55	43.84	tr|H0UXN2|H0UXN2_CAVPO	Uncharacterized protein OS=Cavia porcellus GN=SYT1 PE=4 SV=1	CAVPO	22

165	39.72	39.8	59.31	59.31	56.28	tr|H0V2B3|H0V2B3_CAVPO	Elongation factor 1-alpha OS=Cavia porcellus GN=EEF1A1 PE=3 SV=1	CAVPO	29
166	39.67	43.7	54.17	51.95	45.64	tr|H0UXQ5|H0UXQ5_CAVPO	T-complex protein 1 subunit delta OS=Cavia porcellus GN=CCT4 PE=3 SV=1	CAVPO	24
167	39.47	50.6	52.85	49.31	49.31	tr|H0WBX1|H0WBX1_CAVPO	Uncharacterized protein (Fragment) OS=Cavia porcellus GN=LOC100717205 PE=3 SV=1	CAVPO	35
168	39.42	39.5	62.11	61.26	59.37	tr|H0V3G6|H0V3G6_CAVPO	Uncharacterized protein OS=Cavia porcellus GN=Cndp2 PE=4 SV=1	CAVPO	22
169	39.38	41.5	54.33	48.69	47.08	tr|H0W9M5|H0W9M5_CAVPO	Uncharacterized protein OS=Cavia porcellus GN=Nefl PE=3 SV=1	CAVPO	27
170	39.05	39.1	46.91	46.68	46.45	tr|H0WBQ8|H0WBQ8_CAVPO	Uncharacterized protein OS=Cavia porcellus GN=EEF1G PE=4 SV=1	CAVPO	24
171	38.97	39.1	33.64	28.57	23.78	tr|H0VJP8|H0VJP8_CAVPO	Uncharacterized protein OS=Cavia porcellus GN=AP3B2 PE=4 SV=1	CAVPO	22
172	38.89	39.5	68.2	58.6	55	tr|H0UTG9|H0UTG9_CAVPO	Uncharacterized protein OS=Cavia porcellus GN=ABAT PE=3 SV=1	CAVPO	23
173	38.86	39	40.83	36.56	29.65	tr|H0V1N2|H0V1N2_CAVPO	Uncharacterized protein (Fragment) OS=Cavia porcellus GN=VPS35 PE=4 SV=1	CAVPO	20
174	38.72	40.9	36.23	32.71	29.19	tr|H0VBK1|H0VBK1_CAVPO	Uncharacterized protein OS=Cavia porcellus GN=Hnrnpul1 PE=4 SV=1	CAVPO	21
175	38.67	78.2	64.8	62.79	59.48	tr|H0V1W9|H0V1W9_CAVPO	Uncharacterized protein OS=Cavia porcellus GN=HSP90AB1 PE=3 SV=1	CAVPO	55
176	38.53	40.1	66.45	61.79	58.14	tr|H0VE35|H0VE35_CAVPO	Uncharacterized protein (Fragment) OS=Cavia porcellus GN=SLC25A5 PE=3 SV=1	CAVPO	36
177	38.49	39	41.31	32.88	27.47	tr|H0VI49|H0VI49_CAVPO	Uncharacterized protein OS=Cavia porcellus GN=SART3 PE=4 SV=1	CAVPO	21
178	38.43	38.5	42.82	40.79	32.66	tr|H0VL76|H0VL76_CAVPO	Ubiquitin carboxyl-terminal hydrolase OS=Cavia porcellus GN=USP5 PE=3 SV=1	CAVPO	22

179	38.35	44.1	43.71	38.31	32.9	tr|H0VC58|H0VC58_CAVPO	6-phosphofructokinase OS=Cavia porcellus GN=PFKM PE=3 SV=1	CAVPO	23
180	38.28	40	65.49	58.41	58.41	tr|H0VH39|H0VH39_CAVPO	Elongation factor Tu OS=Cavia porcellus GN=TUFM PE=3 SV=1	CAVPO	21
181	38.26	38.3	49.91	46.04	44.75	tr|H0V4U2|H0V4U2_CAVPO	Glutamate dehydrogenase OS=Cavia porcellus PE=3 SV=1	CAVPO	24
182	38.24	38.3	23.48	17.98	12.22	tr|H0VCI4|H0VCI4_CAVPO	Uncharacterized protein (Fragment) OS=Cavia porcellus GN=CHD4 PE=4 SV=1	CAVPO	18
183	38.06	38.3	41.77	35.8	24.49	tr|H0VH65|H0VH65_CAVPO	Uncharacterized protein OS=Cavia porcellus GN=EFTUD2 PE=4 SV=1	CAVPO	18
184	37.91	38	59.02	56.12	55.01	tr|H0UWB2|H0UWB2_CAVPO	Uncharacterized protein OS=Cavia porcellus GN=HNRNPH1 PE=4 SV=1	CAVPO	25
185	37.77	56	75.28	74.83	70.56	tr|H0VNS2|H0VNS2_CAVPO	Uncharacterized protein OS=Cavia porcellus GN=GDI2 PE=4 SV=1	CAVPO	35
186	37.66	37.8	41.32	26.89	20.28	tr|H0V7M6|H0V7M6_CAVPO	Uncharacterized protein OS=Cavia porcellus GN=SUGP2 PE=4 SV=1	CAVPO	18
187	37.43	37.5	58.45	57	51.93	tr|H0VP28|H0VP28_CAVPO	Isocitrate dehydrogenase [NADP] OS=Cavia porcellus GN=IDH1 PE=3 SV=1	NADP	19
188	37.39	39.2	12.6	10.14	4.543	tr|H0V4J4|H0V4J4_CAVPO	Uncharacterized protein OS=Cavia porcellus GN=HUWE1 PE=4 SV=1	CAVPO	17
189	37.34	37.4	46.14	44.55	42.97	tr|H0VR32|H0VR32_CAVPO	tRNA-splicing ligase RtcB homolog OS=Cavia porcellus GN=RTCB PE=3 SV=1	CAVPO	20
190	37.32	37.4	54.35	46.74	39.49	tr|H0VUK3|H0VUK3_CAVPO	Uncharacterized protein OS=Cavia porcellus GN=MPP2 PE=4 SV=1	CAVPO	23
191	37.31	37.3	29.75	29.75	25.93	tr|H0UWU9|H0UWU9_CAVPO	Uncharacterized protein (Fragment) OS=Cavia porcellus GN=SNAP91 PE=4 SV=1	CAVPO	22
192	37.27	37.4	61.72	54.95	47.19	tr|Q8R4Z8|Q8R4Z8_CAVPO	WD repeat1 protein OS=Cavia porcellus PE=2 SV=1	CAVPO	19

193	37.18	37.2	49.83	44.58	41.96	tr|H0VMJ6|H0VMJ6_CAVPO	Uncharacterized protein (Fragment) OS=Cavia porcellus GN=PABPC3 PE=4 SV=1	CAVPO	24
194	36.98	42.7	56.35	51.8	46.76	tr|H0UX23|H0UX23_CAVPO	Uncharacterized protein OS=Cavia porcellus GN=Ckmt1a PE=3 SV=1	CAVPO	41
195	36.72	36.7	63.57	60.63	60.63	tr|H0VSD7|H0VSD7_CAVPO	Uncharacterized protein (Fragment) OS=Cavia porcellus GN=SFPQ PE=4 SV=1	CAVPO	25
196	36.67	36.8	54.4	47.2	47.2	tr|H0WCA5|H0WCA5_CAVPO	Uncharacterized protein (Fragment) OS=Cavia porcellus GN=CKAP4 PE=4 SV=1	CAVPO	21
197	36.64	36.7	54.94	50.84	43.86	tr|H0V0M5|H0V0M5_CAVPO	Uncharacterized protein OS=Cavia porcellus GN=Ssb PE=4 SV=1	CAVPO	23
198	36.6	37.1	52.84	38.12	37.06	tr|H0VF09|H0VF09_CAVPO	Uncharacterized protein OS=Cavia porcellus GN=YARS PE=4 SV=1	CAVPO	19
199	36.54	36.6	55.53	55.29	53.18	tr|H0UYB6|H0UYB6_CAVPO	Uncharacterized protein OS=Cavia porcellus GN=DDX39B PE=4 SV=1	CAVPO	21
200	36.45	49.9	34.69	23.21	16.68	tr|H0UTQ3|H0UTQ3_CAVPO	Uncharacterized protein OS=Cavia porcellus GN=MYH9 PE=4 SV=1	CAVPO	27
201	36.1	36.1	56.33	56.33	49.56	tr|H0UYL6|H0UYL6_CAVPO	Uncharacterized protein OS=Cavia porcellus GN=FKBP4 PE=4 SV=1	CAVPO	19
202	35.88	35.9	66.04	60.62	59.17	tr|H0V2S5|H0V2S5_CAVPO	Uncharacterized protein OS=Cavia porcellus GN=UQCRC1 PE=3 SV=1	CAVPO	31
203	35.88	35.9	80.28	77.51	77.51	tr|H0VQ28|H0VQ28_CAVPO	Uncharacterized protein OS=Cavia porcellus GN=PPA1 PE=4 SV=1	CAVPO	20
204	35.83	35.9	65.75	63.39	61.02	tr|H0V8Q6|H0V8Q6_CAVPO	Uncharacterized protein OS=Cavia porcellus GN=PGAM1 PE=3 SV=1	CAVPO	46
205	35.49	35.5	82.95	80.18	79.72	tr|H0VAX0|H0VAX0_CAVPO	Uncharacterized protein OS=Cavia porcellus GN=LOC100731896 PE=3 SV=1	CAVPO	31
206	35.48	35.5	30.17	26.44	26.44	tr|H0UVA7|H0UVA7_CAVPO	Uncharacterized protein OS=Cavia porcellus GN=NCL PE=4 SV=1	CAVPO	20

207	35.45	50.7	54.34	49.04	45.34	tr|H0V9E4|H0V9E4_CAVPO	Uncharacterized protein (Fragment) OS=Cavia porcellus GN=DDX5 PE=3 SV=1	CAVPO	30
208	35.23	35.3	43.67	39.44	33.56	tr|H0VI64|H0VI64_CAVPO	Uncharacterized protein OS=Cavia porcellus GN=CCAR2 PE=4 SV=1	CAVPO	19
209	35.22	35.3	31.12	27.62	23.11	tr|H0UY46|H0UY46_CAVPO	Uncharacterized protein OS=Cavia porcellus GN=NNT PE=4 SV=1	CAVPO	19
210	35.2	35.3	32.26	28.76	19.54	tr|H0UZS5|H0UZS5_CAVPO	Ubiquitin carboxyl-terminal hydrolase (Fragment) OS=Cavia porcellus GN=USP7 PE=3 SV=1	CAVPO	16
211	35.03	35	58.33	55.65	55.65	tr|H0UTZ2|H0UTZ2_CAVPO	Histone H2A OS=Cavia porcellus GN=H2AFY PE=3 SV=1	CAVPO	21
212	35.03	35.1	50.6	47.4	45.8	tr|H0VBD5|H0VBD5_CAVPO	Uncharacterized protein (Fragment) OS=Cavia porcellus GN=FSCN1 PE=4 SV=1	CAVPO	22
213	34.95	35	69.21	60.59	57.88	tr|H0VJ13|H0VJ13_CAVPO	Uncharacterized protein OS=Cavia porcellus GN=PSMC5 PE=3 SV=1	CAVPO	19
214	34.95	35	45.38	42.69	38.08	tr|H0VT52|H0VT52_CAVPO	Uncharacterized protein OS=Cavia porcellus GN=ALDH9A1 PE=3 SV=1	CAVPO	21
215	34.8	34.8	80.68	78.31	72.88	tr|H0VHA7|H0VHA7_CAVPO	Uncharacterized protein OS=Cavia porcellus GN=NAPA PE=4 SV=1	CAVPO	18
216	34.75	34.8	22.17	16.38	14.11	tr|H0VJA5|H0VJA5_CAVPO	Uncharacterized protein OS=Cavia porcellus GN=UGGT1 PE=4 SV=1	CAVPO	17
217	34.68	34.8	45.63	40.29	38.2	tr|H0WCI8|H0WCI8_CAVPO	Uncharacterized protein OS=Cavia porcellus GN=DLG4 PE=4 SV=1	CAVPO	21
218	34.66	34.8	32.61	30.01	22.01	tr|H0W9B7|H0W9B7_CAVPO	Lon protease homolog, mitochondrial OS=Cavia porcellus GN=LONP1 PE=3 SV=1	CAVPO	18
219	34.51	42.6	48.07	42.23	34.79	tr|H0WBB5|H0WBB5_CAVPO	Uncharacterized protein (Fragment) OS=Cavia porcellus GN=PFKL PE=3 SV=1	CAVPO	25
220	34.5	34.7	46.85	41.18	31.99	tr|H0VC11|H0VC11_CAVPO	Uncharacterized protein OS=Cavia porcellus	CAVPO	19

							GN=SF3B2 PE=4 SV=1		
221	34.4	34.4	40.75	34.99	31.77	tr|H0VLA9|H0VLA9_CAVPO	Uncharacterized protein (Fragment) OS=Cavia porcellus GN=IMMT PE=4 SV=1	CAVPO	17
222	34.39	34.4	35.89	33.73	25.21	tr|H0V673|H0V673_CAVPO	Uncharacterized protein (Fragment) OS=Cavia porcellus GN=Trim28 PE=4 SV=1	CAVPO	18
223	34.37	34.4	34.73	28.9	24.84	tr|H0V7D2|H0V7D2_CAVPO	Uncharacterized protein OS=Cavia porcellus GN=SND1 PE=4 SV=1	CAVPO	19
224	34.33	38.5	62.87	60.18	54.49	tr|H0V9E8|H0V9E8_CAVPO	L-lactate dehydrogenase OS=Cavia porcellus GN=LDHB PE=3 SV=1	CAVPO	33
225	34.32	34.4	58.82	48.53	48.53	tr|H0VM96|H0VM96_CAVPO	Uncharacterized protein (Fragment) OS=Cavia porcellus GN=ATIC PE=4 SV=1	CAVPO	19
226	34.31	34.7	40.04	30.92	23.39	tr|H0VML7|H0VML7_CAVPO	Uncharacterized protein OS=Cavia porcellus GN=IARS2 PE=3 SV=1	CAVPO	17
227	34.28	34.3	79.8	79.8	78.79	tr|H0VWR9|H0VWR9_CAVPO	Uncharacterized protein OS=Cavia porcellus GN=RCVRN PE=4 SV=1	CAVPO	36
228	34.2	34.2	49.38	46.6	45.06	tr|H0V2Q9|H0V2Q9_CAVPO	Uncharacterized protein OS=Cavia porcellus PE=4 SV=1	CAVPO	27
229	34.16	34.2	53.58	53.24	52.05	tr|H0VAL1|H0VAL1_CAVPO	Uncharacterized protein OS=Cavia porcellus GN=HNRNPL PE=4 SV=1	CAVPO	34
230	34.15	34.2	43.75	41.44	41.44	tr|H0VRQ8|H0VRQ8_CAVPO	Adenosylhomocysteinase (Fragment) OS=Cavia porcellus GN=AHCY PE=3 SV=1	CAVPO	18
231	33.87	38.1	42.69	38.97	34.67	tr|H0WCD0|H0WCD0_CAVPO	Uncharacterized protein OS=Cavia porcellus GN=NEFM PE=3 SV=1	CAVPO	24
232	33.83	36	55.16	54.73	46.81	tr|H0VGH4|H0VGH4_CAVPO	Uncharacterized protein OS=Cavia porcellus GN=Nono PE=4 SV=1	CAVPO	21
233	33.76	33.8	72.94	69.02	68.63	tr|H0VBD1|H0VBD1_CAVPO	Uncharacterized protein (Fragment) OS=Cavia porcellus GN=YWHAE PE=3 SV=1	CAVPO	30

234	33.74	33.9	44.85	40.15	32.5	tr|H0V014|H0V014_CAVPO	Uncharacterized protein OS=Cavia porcellus GN=HSPA12A PE=4 SV=1	CAVPO	18
235	33.68	33.8	50.65	48.05	39.9	tr|H0VIT7|H0VIT7_CAVPO	Uncharacterized protein (Fragment) OS=Cavia porcellus GN=TOMM70A PE=4 SV=1	CAVPO	17
236	33.63	33.7	70.71	67.16	65.09	tr|H0W7Y9|H0W7Y9_CAVPO	Malate dehydrogenase OS=Cavia porcellus GN=Mdh2 PE=3 SV=1	CAVPO	27
237	33.37	33.4	51.81	49.4	49.4	tr|H0VLM1|H0VLM1_CAVPO	Uncharacterized protein (Fragment) OS=Cavia porcellus GN=SEPT7 PE=3 SV=1	CAVPO	20
238	33.31	34.4	35.53	34.43	32.1	tr|H0V0P2|H0V0P2_CAVPO	Uncharacterized protein OS=Cavia porcellus GN=ADD1 PE=4 SV=1	CAVPO	21
239	33.31	33.5	27.09	23.38	21.01	tr|H0V5Q7|H0V5Q7_CAVPO	Uncharacterized protein OS=Cavia porcellus GN=Cse1l PE=4 SV=1	CAVPO	20
240	33.26	33.4	51.5	43.78	43.78	tr|H0VS46|H0VS46_CAVPO	Citrate synthase (Fragment) OS=Cavia porcellus GN=CS PE=3 SV=1	CAVPO	20
241	33.19	33.3	51.73	48.96	46.88	tr|H0VS89|H0VS89_CAVPO	Uncharacterized protein (Fragment) OS=Cavia porcellus GN=SUCLA2 PE=3 SV=1	CAVPO	19
242	33.08	33.2	38.7	30.03	23.17	tr|H0V1M9|H0V1M9_CAVPO	Uncharacterized protein OS=Cavia porcellus GN=ACO1 PE=4 SV=1	CAVPO	16
243	33.08	35.2	43.3	32.82	28.69	tr|H0V2V2|H0V2V2_CAVPO	Uncharacterized protein (Fragment) OS=Cavia porcellus GN=EZR PE=4 SV=1	CAVPO	20
244	32.77	32.8	43.12	35.82	30.22	tr|H0VMZ4|H0VMZ4_CAVPO	Uncharacterized protein OS=Cavia porcellus GN=FARSB PE=4 SV=1	CAVPO	16
245	32.75	32.8	25.29	23.13	20.16	tr|H0V2V7|H0V2V7_CAVPO	Uncharacterized protein (Fragment) OS=Cavia porcellus GN=AP3D1 PE=4 SV=1	CAVPO	18
246	32.67	32.8	46.82	46.25	43.63	tr|H0VBP7|H0VBP7_CAVPO	Uncharacterized protein OS=Cavia porcellus GN=EHD1 PE=4 SV=1	CAVPO	19
247	32.57	33.1	18.42	8.175	4.206	tr|H0VB93|H0VB93_CAVPO	Uncharacterized protein OS=Cavia porcellus GN=PLEC PE=4 SV=1	CAVPO	16

248	32.53	32.6	29.62	26.39	17.46	tr|H0V5Q1|H0V5Q1_CAVPO	Uncharacterized protein (Fragment) OS=Cavia porcellus GN=AKAP12 PE=4 SV=1	CAVPO	19
249	32.53	32.6	22.2	19.01	14.7	tr|H0VC79|H0VC79_CAVPO	Uncharacterized protein OS=Cavia porcellus GN=CYFIP2 PE=4 SV=1	CAVPO	16
250	31.94	32	45.1	33.56	27.52	tr|H0VSZ2|H0VSZ2_CAVPO	Uncharacterized protein (Fragment) OS=Cavia porcellus GN=DNM1L PE=3 SV=1	CAVPO	16
251	31.89	31.9	44.39	43.46	40.19	tr|H0WAS6|H0WAS6_CAVPO	Uncharacterized protein OS=Cavia porcellus GN=LOC100718808 PE=4 SV=1	CAVPO	24
252	31.83	31.8	56.35	56.35	52.79	tr|H0VS10|H0VS10_CAVPO	Uncharacterized protein OS=Cavia porcellus GN=Pa2g4 PE=4 SV=1	CAVPO	16
253	31.81	33.1	53.21	47.39	45.98	tr|H0W278|H0W278_CAVPO	Uncharacterized protein (Fragment) OS=Cavia porcellus GN=LOC100728538 PE=3 SV=1	CAVPO	18
254	31.58	31.6	60.51	57.96	54.14	tr|H0VT90|H0VT90_CAVPO	Uncharacterized protein (Fragment) OS=Cavia porcellus GN=GNAO1 PE=4 SV=1	CAVPO	22
255	31.43	42.6	47.95	42.16	36.25	tr|H0VL02|H0VL02_CAVPO	Uncharacterized protein OS=Cavia porcellus GN=EPB41L1 PE=4 SV=1	CAVPO	23
256	31.42	31.5	32.64	24.84	17.44	tr|H0VIN3|H0VIN3_CAVPO	Uncharacterized protein OS=Cavia porcellus GN=PITPNM1 PE=4 SV=1	CAVPO	17
257	31.41	31.4	61.64	59.91	44.4	tr|H0V894|H0V894_CAVPO	Uncharacterized protein OS=Cavia porcellus GN=NDUFV1 PE=4 SV=1	CAVPO	16
258	31.41	31.5	43.9	42.37	33.9	tr|H0VNX9|H0VNX9_CAVPO	Uncharacterized protein (Fragment) OS=Cavia porcellus GN=PTPN11 PE=4 SV=1	CAVPO	20
259	31.35	31.4	67.08	65.84	53.09	tr|H0UT17|H0UT17_CAVPO	Uncharacterized protein OS=Cavia porcellus GN=PDC PE=4 SV=1	CAVPO	24
260	31.3	31.3	67.14	67.14	62.38	tr|H0W9D3|H0W9D3_CAVPO	Uncharacterized protein (Fragment) OS=Cavia porcellus GN=GSTP1 PE=3 SV=1	CAVPO	31
261	31.25	31.4	20.85	16.54	14.84	tr|H0VJV7|H0VJV7_CAVPO	Uncharacterized protein OS=Cavia porcellus GN=NFASC PE=4 SV=1	CAVPO	16

262	31.2	31.2	48.73	41.49	41.49	tr|H0VN04|H0VN04_CAVPO	Uncharacterized protein OS=Cavia porcellus GN=ALDH7A1 PE=3 SV=1	CAVPO	19
263	31.02	31.1	86.1	79.92	74.9	tr|H0VB71|H0VB71_CAVPO	Uncharacterized protein OS=Cavia porcellus GN=CA2 PE=4 SV=1	CAVPO	62
264	31.01	31.1	35.96	29.7	23.84	tr|H0VEC8|H0VEC8_CAVPO	Uncharacterized protein OS=Cavia porcellus GN=GPRIN1 PE=4 SV=1	CAVPO	16
265	30.86	30.9	44.99	39.89	39.89	tr|H0V4N2|H0V4N2_CAVPO	Uncharacterized protein OS=Cavia porcellus GN=PSIP1 PE=4 SV=1	CAVPO	22
266	30.84	30.9	54.2	46.56	37.98	tr|H0V2J8|H0V2J8_CAVPO	Uncharacterized protein OS=Cavia porcellus GN=PAK2 PE=4 SV=1	CAVPO	16
267	30.81	30.9	23.72	17.6	12.63	tr|H0VKV1|H0VKV1_CAVPO	Uncharacterized protein OS=Cavia porcellus GN=SYNJ1 PE=4 SV=1	CAVPO	16
268	30.73	30.8	72.33	71.84	68.45	tr|H0UZK2|H0UZK2_CAVPO	Synaptosomal-associated protein (Fragment) OS=Cavia porcellus GN=SNAP25 PE=3 SV=1	CAVPO	23
269	30.69	36.6	50.1	43.06	41.61	tr|H0V286|H0V286_CAVPO	Uncharacterized protein OS=Cavia porcellus GN=INA PE=3 SV=1	CAVPO	24
270	30.68	30.9	18.38	14.82	9.593	tr|H0VQH3|H0VQH3_CAVPO	Uncharacterized protein OS=Cavia porcellus GN=HCFC1 PE=4 SV=1	CAVPO	15
271	30.67	30.8	64.2	62.35	59.26	tr|H0VMU3|H0VMU3_CAVPO	Uncharacterized protein (Fragment) OS=Cavia porcellus GN=MAPK1 PE=4 SV=1	CAVPO	17
272	30.62	30.7	49	42.68	35.59	tr|H0VDG4|H0VDG4_CAVPO	Uncharacterized protein (Fragment) OS=Cavia porcellus GN=SDHA PE=4 SV=1	CAVPO	17
273	30.62	30.7	44.06	38.75	33.59	tr|H0VH45|H0VH45_CAVPO	Uncharacterized protein OS=Cavia porcellus GN=UBA2 PE=3 SV=1	CAVPO	16
274	30.6	30.6	61.35	57.84	52.7	tr|H0UVB7|H0UVB7_CAVPO	Uncharacterized protein OS=Cavia porcellus GN=PPID PE=4 SV=1	CAVPO	19
275	30.59	30.7	84.85	77.58	73.94	tr|H0UZ02|H0UZ02_CAVPO	Peptidyl-prolyl cis-trans isomerase OS=Cavia porcellus GN=PPIA PE=3 SV=1	CAVPO	49

276	30.5	30.6	31.19	29.2	21.13	tr|H0VNL9|H0VNL9_CAVPO	Uncharacterized protein OS=Cavia porcellus GN=CTNNA1 PE=4 SV=1	CAVPO	18
277	30.47	30.6	31.86	29.11	26.37	tr|H0UTX6|H0UTX6_CAVPO	Uncharacterized protein (Fragment) OS=Cavia porcellus GN=PSMD1 PE=4 SV=1	CAVPO	17
278	30.44	31	60.91	56.94	55.95	tr|H0WDA2|H0WDA2_CAVPO	Uncharacterized protein OS=Cavia porcellus GN=PRPF19 PE=4 SV=1	CAVPO	17
279	30.41	30.7	45.78	39.56	27.26	tr|H0V525|H0V525_CAVPO	Annexin (Fragment) OS=Cavia porcellus GN=ANXA6 PE=3 SV=1	CAVPO	13
280	30.25	30.4	50.85	42.77	31.06	tr|H0W8I2|H0W8I2_CAVPO	Uncharacterized protein (Fragment) OS=Cavia porcellus GN=RUVBL2 PE=4 SV=1	CAVPO	14
281	30.21	30.6	57.31	57.31	51.54	tr|H0UTU2|H0UTU2_CAVPO	Uncharacterized protein OS=Cavia porcellus GN=PSPC1 PE=4 SV=1	CAVPO	17
282	30.17	30.3	56.87	52.64	49.68	tr|H0UZD4|H0UZD4_CAVPO	Uncharacterized protein (Fragment) OS=Cavia porcellus GN=DNPEP PE=3 SV=1	CAVPO	19
283	30.16	30.4	61.26	56.21	43.16	tr|H0V5E7|H0V5E7_CAVPO	Uncharacterized protein OS=Cavia porcellus GN=HADHB PE=3 SV=1	CAVPO	16
284	30.14	30.2	59.91	51.25	48.52	tr|H0VBR1|H0VBR1_CAVPO	Uncharacterized protein (Fragment) OS=Cavia porcellus GN=PSMC3 PE=3 SV=1	CAVPO	18
285	30.13	30.2	56.97	54.73	51.24	tr|H0VMG4|H0VMG4_CAVPO	Uncharacterized protein OS=Cavia porcellus GN=DCTN2 PE=4 SV=1	CAVPO	15
286	30.05	30.1	46.77	44.4	42.46	tr|H0VLR4|H0VLR4_CAVPO	Uncharacterized protein (Fragment) OS=Cavia porcellus GN=MECP2 PE=4 SV=1	CAVPO	21
287	29.99	30.2	30.49	24.8	13.16	tr|H0VSS2|H0VSS2_CAVPO	Eukaryotic translation initiation factor 3 subunit A OS=Cavia porcellus GN=EIF3A PE=3 SV=1	CAVPO	12
288	29.95	30.1	31.67	28.33	17.96	tr|H0V416|H0V416_CAVPO	Uncharacterized protein (Fragment) OS=Cavia porcellus GN=IPO5 PE=4 SV=1	CAVPO	15
289	29.91	30.2	32.88	30.04	27.35	tr|H0VBE1|H0VBE1_CAVPO	Uncharacterized protein OS=Cavia porcellus	CAVPO	17

							GN=RBM14 PE=4 SV=1		
290	29.88	30	67.69	65.23	58.77	tr|H0VM25|H0VM25_CAVPO	Uncharacterized protein OS=Cavia porcellus GN=AKR1A1 PE=4 SV=1	CAVPO	15
291	29.8	43.8	73.73	71.66	70.05	tr|H0VDC5|H0VDC5_CAVPO	Uncharacterized protein OS=Cavia porcellus GN=ENO2 PE=3 SV=1	CAVPO	53
292	29.78	30	43.48	36.82	26.97	tr|H0V9Z5|H0V9Z5_CAVPO	Uncharacterized protein OS=Cavia porcellus GN=RARS PE=3 SV=1	CAVPO	15
293	29.7	30.1	84.76	80.95	80	tr|H0VJX4|H0VJX4_CAVPO	Uncharacterized protein (Fragment) OS=Cavia porcellus GN=HDGF PE=4 SV=1	CAVPO	23
294	29.69	29.8	65.3	63.41	58.04	tr|H0VF29|H0VF29_CAVPO	Uncharacterized protein OS=Cavia porcellus GN=RLBP1 PE=4 SV=1	CAVPO	20
295	29.63	29.7	65.7	62.79	56.98	tr|H0VCZ6|H0VCZ6_CAVPO	Uncharacterized protein (Fragment) OS=Cavia porcellus GN=GNB2L1 PE=4 SV=1	CAVPO	18
296	29.39	29.5	25.7	20.79	20.09	tr|H0UWX2|H0UWX2_CAVPO	Uncharacterized protein OS=Cavia porcellus GN=SRRT PE=4 SV=1	CAVPO	16
297	29.24	51.1	38.34	35.07	29.55	tr|H0V4P1|H0V4P1_CAVPO	Uncharacterized protein OS=Cavia porcellus GN=AP2A1 PE=4 SV=1	CAVPO	27
298	29.22	29.4	62.33	52.91	47.98	tr|H0W4K9|H0W4K9_CAVPO	Uncharacterized protein OS=Cavia porcellus GN=SCLY PE=4 SV=1	CAVPO	17
299	29.02	29.1	58.19	49.9	37.96	tr|H0VLV4|H0VLV4_CAVPO	Uncharacterized protein OS=Cavia porcellus GN=LAP3 PE=3 SV=1	CAVPO	17
300	29	29.1	43.39	39.37	39.37	tr|A4L9L3|A4L9L3_CAVPO	Rhodopsin OS=Cavia porcellus GN=Rho PE=2 SV=1	CAVPO	65
301	28.98	31.2	43.79	41.57	36.83	tr|H0W7J5|H0W7J5_CAVPO	Uncharacterized protein OS=Cavia porcellus GN=FUBP1 PE=4 SV=1	CAVPO	19
302	28.95	29	51.3	49.74	47.14	tr|H0V665|H0V665_CAVPO	Uncharacterized protein (Fragment) OS=Cavia porcellus GN=CADM1 PE=4 SV=1	CAVPO	25

303	28.91	29.1	28.17	20.43	17.22	tr|H0WC63|H0WC63_CAVPO	Uncharacterized protein (Fragment) OS=Cavia porcellus GN=TJP2 PE=4 SV=1	CAVPO	15
304	28.9	29	34.58	30.94	22.64	tr|H0VKL0|H0VKL0_CAVPO	Uncharacterized protein OS=Cavia porcellus GN=USO1 PE=4 SV=1	CAVPO	14
305	28.88	28.9	63.38	63.1	60.85	tr|H0V4H8|H0V4H8_CAVPO	Uncharacterized protein OS=Cavia porcellus GN=Hnrnpk PE=4 SV=1	CAVPO	27
306	28.82	28.9	50.79	46.86	38.22	tr|H0VFT3|H0VFT3_CAVPO	Uncharacterized protein OS=Cavia porcellus GN=ATP6V1C1 PE=4 SV=1	CAVPO	15
307	28.75	28.8	69.38	62.08	55.62	tr|H0VNW7|H0VNW7_CAVPO	Uncharacterized protein OS=Cavia porcellus GN=PCBP1 PE=4 SV=1	CAVPO	19
308	28.71	28.8	78.41	70.04	70.04	tr|H0VIE5|H0VIE5_CAVPO	Uncharacterized protein (Fragment) OS=Cavia porcellus GN=PRDX6 PE=4 SV=1	CAVPO	19
309	28.71	32.6	48.02	36.59	32.32	tr|H0VP03|H0VP03_CAVPO	Uncharacterized protein (Fragment) OS=Cavia porcellus GN=DDX3X PE=3 SV=1	CAVPO	19
310	28.65	28.7	91.98	91.98	89.84	tr|H0W1E7|H0W1E7_CAVPO	Uncharacterized protein OS=Cavia porcellus GN=PEBP1 PE=4 SV=1	CAVPO	21
311	28.63	64.2	79.4	76.62	73.84	tr|H0UYF4|H0UYF4_CAVPO	Uncharacterized protein (Fragment) OS=Cavia porcellus GN=TUBB3 PE=3 SV=1	CAVPO	90
312	28.6	28.6	74.91	73.5	70.67	tr|H0W405|H0W405_CAVPO	Uncharacterized protein OS=Cavia porcellus GN=Vdac1 PE=4 SV=1	CAVPO	27
313	28.56	28.7	24.36	21.61	16.64	tr|H0VQW6|H0VQW6_CAVPO	Uncharacterized protein (Fragment) OS=Cavia porcellus GN=GLG1 PE=4 SV=1	CAVPO	15
314	28.55	28.6	54.58	51.86	51.86	tr|H0UWA3|H0UWA3_CAVPO	Uncharacterized protein OS=Cavia porcellus GN=LOC100713856 PE=4 SV=1	CAVPO	22
315	28.5	28.6	20.82	17.35	10.2	tr|H0WB17|H0WB17_CAVPO	Uncharacterized protein OS=Cavia porcellus GN=CPNE1 PE=4 SV=1	CAVPO	14
316	28.41	28.6	25.33	17.25	13.33	tr|H0VHJ2|H0VHJ2_CAVPO	Uncharacterized protein (Fragment) OS=Cavia porcellus GN=KIF21A PE=3 SV=1	CAVPO	17

317	28.36	33.4	44.85	44.85	42.16	tr|H0VIL8|H0VIL8_CAVPO	Uncharacterized protein OS=Cavia porcellus GN=EIF4A2 PE=3 SV=1	CAVPO	19
318	28.31	28.5	39.22	33.99	27.94	tr|H0VS65|H0VS65_CAVPO	Uncharacterized protein (Fragment) OS=Cavia porcellus GN=GUCY1B3 PE=3 SV=1	CAVPO	15
319	28.23	28.3	51.75	51.75	44.96	tr|H0VEU3|H0VEU3_CAVPO	Uncharacterized protein OS=Cavia porcellus GN=RUVBL1 PE=4 SV=1	CAVPO	16
320	28.22	28.4	49.41	46.85	36.81	tr|H0UUQ7|H0UUQ7_CAVPO	Uncharacterized protein OS=Cavia porcellus GN=FARSA PE=3 SV=1	CAVPO	15
321	28.15	28.3	41.83	34.77	26.41	tr|H0W6X4|H0W6X4_CAVPO	Uncharacterized protein OS=Cavia porcellus GN=SEC23A PE=4 SV=1	CAVPO	17
322	28.13	28.2	48.79	43.27	41.06	tr|H0V8C7|H0V8C7_CAVPO	Uncharacterized protein OS=Cavia porcellus GN=UQCRC2 PE=3 SV=1	CAVPO	17
323	28.12	28.3	60	58.44	41.11	tr|H0VCQ1|H0VCQ1_CAVPO	Uncharacterized protein OS=Cavia porcellus GN=LANCL2 PE=4 SV=1	CAVPO	18
324	28.11	28.2	42.86	41.24	35.02	tr|H0UV63|H0UV63_CAVPO	Uncharacterized protein OS=Cavia porcellus GN=CMAS PE=4 SV=1	CAVPO	14
325	28.1	28.1	59.94	58.68	58.68	tr|H0UVQ5|H0UVQ5_CAVPO	Uncharacterized protein (Fragment) OS=Cavia porcellus GN=PRKAR2A PE=4 SV=1	CAVPO	16
326	28.05	28.3	26.28	23.62	15.85	tr|H0VU24|H0VU24_CAVPO	Uncharacterized protein OS=Cavia porcellus GN=THRAP3 PE=4 SV=1	CAVPO	14
327	28.01	28.3	50.63	41.59	23.37	tr|H0VM53|H0VM53_CAVPO	Uncharacterized protein (Fragment) OS=Cavia porcellus GN=ACSL6 PE=4 SV=1	CAVPO	14
328	27.95	30.1	51.99	48.01	45.26	tr|H0UXA8|H0UXA8_CAVPO	Transaldolase OS=Cavia porcellus GN=TALDO1 PE=3 SV=1	CAVPO	19
329	27.91	28.1	25.07	23.45	22.64	tr|H0VGG4|H0VGG4_CAVPO	Uncharacterized protein OS=Cavia porcellus GN=SV2A PE=4 SV=1	CAVPO	18
330	27.89	28	49	37.03	25.56	tr|H0UV42|H0UV42_CAVPO	Uncharacterized protein OS=Cavia porcellus GN=CDC5L PE=4 SV=1	CAVPO	14

331	27.88	28	34.88	27.15	18.21	tr|H0VRK1|H0VRK1_CAVPO	Uncharacterized protein (Fragment) OS=Cavia porcellus GN=COPB2 PE=4 SV=1	CAVPO	14
332	27.66	27.7	50.69	49.31	49.31	tr|H0UXR9|H0UXR9_CAVPO	Uncharacterized protein OS=Cavia porcellus GN=STX1B PE=3 SV=1	CAVPO	17
333	27.64	28.2	23.23	16.97	9.804	tr|H0V2Q4|H0V2Q4_CAVPO	Uncharacterized protein (Fragment) OS=Cavia porcellus GN=MAP3K5 PE=4 SV=1	CAVPO	13
334	27.63	28	18.78	15.71	14.1	tr|H0VQQ2|H0VQQ2_CAVPO	Uncharacterized protein OS=Cavia porcellus GN=CNGB1 PE=4 SV=1	CAVPO	20
335	27.54	27.7	52.12	48.43	39.96	tr|H0VLY5|H0VLY5_CAVPO	Uncharacterized protein (Fragment) OS=Cavia porcellus GN=GLS PE=3 SV=1	CAVPO	20
336	27.47	32	57.11	52.53	48.43	tr|H0W0E9|H0W0E9_CAVPO	Uncharacterized protein (Fragment) OS=Cavia porcellus GN=HNRNPF PE=4 SV=1	CAVPO	19
337	27.44	27.5	52.96	52.66	50.59	tr|H0VT79|H0VT79_CAVPO	Uncharacterized protein (Fragment) OS=Cavia porcellus GN=SH3GL2 PE=4 SV=1	CAVPO	19
338	27.36	27.5	39.05	34.69	31.16	tr|O70529|O70529_CAVPO	Mutifunctional protein2 OS=Cavia porcellus PE=2 SV=1	CAVPO	14
339	27.27	27.4	36.7	32.39	25.26	tr|H0VEQ3|H0VEQ3_CAVPO	Uncharacterized protein (Fragment) OS=Cavia porcellus GN=PADI2 PE=4 SV=1	CAVPO	15
340	27.17	27.5	31.95	28.07	20.18	tr|H0VJD7|H0VJD7_CAVPO	Uncharacterized protein (Fragment) OS=Cavia porcellus GN=AMPD2 PE=4 SV=1	CAVPO	15
341	27.17	27.3	34.85	32.48	26.28	tr|H0VN93|H0VN93_CAVPO	Uncharacterized protein (Fragment) OS=Cavia porcellus GN=NARS PE=3 SV=1	CAVPO	13
342	27.12	40.3	45.45	41.73	33.38	tr|H0VN69|H0VN69_CAVPO	Uncharacterized protein (Fragment) OS=Cavia porcellus GN=SLC25A13 PE=3 SV=1	CAVPO	21
343	27.12	27.2	31.69	24.37	22.73	tr|H0W4K7|H0W4K7_CAVPO	Uncharacterized protein OS=Cavia porcellus GN=SAFB PE=4 SV=1	CAVPO	15
344	27.06	27.2	50.55	47.63	38.32	tr|H0VH80|H0VH80_CAVPO	Uncharacterized protein (Fragment) OS=Cavia porcellus GN=MYEF2 PE=4 SV=1	CAVPO	15

345	26.91	27	40.76	36.01	27.31	tr|H0UUH1|H0UUH1_CAVPO	Uncharacterized protein OS=Cavia porcellus GN=DPP3 PE=4 SV=1	CAVPO	14
346	26.87	26.9	53.8	51.58	49.68	tr|H0UVV9|H0UVV9_CAVPO	Uncharacterized protein OS=Cavia porcellus GN=AKR1B1 PE=4 SV=1	CAVPO	15
347	26.82	27	60.51	56.92	50.26	tr|H0VE97|H0VE97_CAVPO	Pyruvate dehydrogenase E1 component subunit alpha OS=Cavia porcellus GN=Pdha1 PE=3 SV=1	CAVPO	16
348	26.74	26.9	34.26	28.19	22.63	tr|H0VLN9|H0VLN9_CAVPO	Eukaryotic translation initiation factor 3 subunit B OS=Cavia porcellus GN=EIF3B PE=3 SV=1	CAVPO	16
349	26.67	26.8	41.46	41.46	37.25	tr|H0VF70|H0VF70_CAVPO	Uncharacterized protein OS=Cavia porcellus GN=CCBL2 PE=4 SV=1	CAVPO	14
350	26.66	26.7	40.26	32.18	27.23	tr|H0UX41|H0UX41_CAVPO	Uncharacterized protein (Fragment) OS=Cavia porcellus GN=RAP1GDS1 PE=4 SV=1	CAVPO	14
351	26.65	26.8	23.13	17.04	14.4	tr|H0V1J8|H0V1J8_CAVPO	Uncharacterized protein OS=Cavia porcellus GN=SYNM PE=3 SV=1	CAVPO	13
352	26.65	26.8	31.85	24.79	21.06	tr|H0V562|H0V562_CAVPO	Uncharacterized protein OS=Cavia porcellus GN=CTNND1 PE=4 SV=1	CAVPO	15
353	26.65	26.7	43.14	31.81	27.42	tr|H0VHG4|H0VHG4_CAVPO	Uncharacterized protein OS=Cavia porcellus GN=SCP2 PE=4 SV=1	CAVPO	13
354	26.61	27.1	38.1	35.02	28.11	tr|H0UYX2|H0UYX2_CAVPO	Uncharacterized protein OS=Cavia porcellus GN=RNPEP PE=4 SV=1	CAVPO	15
355	26.53	26.6	21.71	19.3	14.81	tr|H0VKW4|H0VKW4_CAVPO	Uncharacterized protein (Fragment) OS=Cavia porcellus GN=UBE2O PE=4 SV=1	CAVPO	14
356	26.51	26.6	43.14	42.14	28.57	tr|H0V7W2|H0V7W2_CAVPO	Uncharacterized protein (Fragment) OS=Cavia porcellus GN=C2CD2L PE=4 SV=1	CAVPO	13
357	26.46	26.6	31.82	28.03	21.02	tr|H0VW42|H0VW42_CAVPO	Uncharacterized protein (Fragment) OS=Cavia porcellus GN=THOP1 PE=3 SV=1	CAVPO	15

358	26.41	26.5	31.2	25.19	14.44	tr|H0VJ01|H0VJ01_CAVPO	Uncharacterized protein OS=Cavia porcellus GN=DDX46 PE=3 SV=1	CAVPO	12
359	26.26	26.3	67.01	59.38	59.38	tr|H0UUF6|H0UUF6_CAVPO	Uncharacterized protein (Fragment) OS=Cavia porcellus GN=PNP PE=4 SV=1	CAVPO	15
360	26.24	30.7	50	40.37	35.74	tr|H0VTM9|H0VTM9_CAVPO	Uncharacterized protein OS=Cavia porcellus GN=MPP6 PE=4 SV=1	CAVPO	19
361	26.23	26.3	64.78	61.01	59.12	tr|H0VR33|H0VR33_CAVPO	Uncharacterized protein OS=Cavia porcellus GN=APEX1 PE=4 SV=1	CAVPO	17
362	26.23	26.3	25.58	19.12	14.9	tr|H0VSS1|H0VSS1_CAVPO	Uncharacterized protein OS=Cavia porcellus GN=CLASP2 PE=4 SV=1	CAVPO	14
363	26.16	26.2	56.44	54.1	52.69	tr|H0VC99|H0VC99_CAVPO	Uncharacterized protein (Fragment) OS=Cavia porcellus GN=CTBP2 PE=3 SV=1	CAVPO	17
364	26.16	26.3	39.09	33.04	23.46	tr|H0VRD6|H0VRD6_CAVPO	Uncharacterized protein OS=Cavia porcellus GN=SF3A1 PE=4 SV=1	CAVPO	14
365	26.09	26.2	50.76	50.54	41.04	tr|H0VLJ1|H0VLJ1_CAVPO	Uncharacterized protein OS=Cavia porcellus GN=NDUFS2 PE=3 SV=1	CAVPO	17
366	26.09	26.2	67.31	67.31	63.46	tr|H0W865|H0W865_CAVPO	Histone H4 (Fragment) OS=Cavia porcellus GN=LOC100736110 PE=3 SV=1	CAVPO	38
367	26	32.4	76.61	76.61	76.61	tr|Q91Y83|Q91Y83_CAVPO	Glutathione S-transferase subunit gYc OS=Cavia porcellus GN=Gstm2 PE=2 SV=1	CAVPO	22
368	25.97	26.1	44.2	35.45	30.68	tr|H0UXV8|H0UXV8_CAVPO	Uncharacterized protein (Fragment) OS=Cavia porcellus GN=TTLL12 PE=4 SV=1	CAVPO	17
369	25.97	26.2	41.06	31.92	23.94	tr|H0VFT4|H0VFT4_CAVPO	Uncharacterized protein OS=Cavia porcellus GN=QARS PE=3 SV=1	CAVPO	15
370	25.97	26	54.19	47.6	47.6	tr|H0VHL7|H0VHL7_CAVPO	Malate dehydrogenase (Fragment) OS=Cavia porcellus GN=MDH1 PE=3 SV=1	CAVPO	26
371	25.97	26	53.17	53.17	44.08	tr|H0VMZ8|H0VMZ8_CAVPO	Uncharacterized protein OS=Cavia porcellus GN=SYN1 PE=4 SV=1	CAVPO	18

372	25.89	25.9	51.8	46.4	46.4	tr|H0VKG9|H0VKG9_CAVPO	Uncharacterized protein OS=Cavia porcellus GN=PACSIN1 PE=4 SV=1	CAVPO	15
373	25.88	26.1	14.72	10.67	8.611	tr|H0VGT5|H0VGT5_CAVPO	Uncharacterized protein OS=Cavia porcellus GN=KIF1A PE=3 SV=1	CAVPO	14
374	25.83	27.3	29.12	19.98	13.23	tr|H0UY50|H0UY50_CAVPO	Uncharacterized protein OS=Cavia porcellus GN=SMARCA5 PE=4 SV=1	CAVPO	12
375	25.76	27.3	63.61	56.46	56.46	tr|H0UZA0|H0UZA0_CAVPO	Uncharacterized protein OS=Cavia porcellus GN=VDAC2 PE=4 SV=1	CAVPO	20
376	25.69	25.9	34.64	25.65	20.31	tr|H0V5Z7|H0V5Z7_CAVPO	Uncharacterized protein (Fragment) OS=Cavia porcellus GN=CUL3 PE=3 SV=1	CAVPO	14
377	25.66	25.8	44.65	34.9	31.71	tr|H0V8G4|H0V8G4_CAVPO	Uncharacterized protein OS=Cavia porcellus GN=PHGDH PE=3 SV=1	CAVPO	14
378	25.65	25.7	77.78	73.91	71.01	tr|H0VTS0|H0VTS0_CAVPO	Uncharacterized protein OS=Cavia porcellus GN=RAB7A PE=3 SV=1	CAVPO	15
379	25.6	25.8	51.9	40.28	37.68	tr|H0V2G6|H0V2G6_CAVPO	Uncharacterized protein OS=Cavia porcellus GN=CAMK2D PE=4 SV=1	CAVPO	17
380	25.57	25.7	48.59	46.29	36.32	tr|H0VKT5|H0VKT5_CAVPO	Uncharacterized protein OS=Cavia porcellus GN=RBMX PE=4 SV=1	CAVPO	14
381	25.52	25.7	21.17	17.74	12.22	tr|H0VEQ7|H0VEQ7_CAVPO	Uncharacterized protein OS=Cavia porcellus GN=TBCD PE=4 SV=1	CAVPO	11
382	25.49	25.6	56.58	53.33	33.69	tr|H0VJK1|H0VJK1_CAVPO	Uncharacterized protein (Fragment) OS=Cavia porcellus GN=TMPO PE=4 SV=1	CAVPO	13
383	25.49	25.6	56.58	53.33	33.69	tr|H0VJK1|H0VJK1_CAVPO	Uncharacterized protein (Fragment) OS=Cavia porcellus GN=TMPO PE=4 SV=1	CAVPO	13
384	25.45	25.7	29.53	22.54	18.12	tr|H0VJ05|H0VJ05_CAVPO	Transcription elongation factor SPT5 OS=Cavia porcellus GN=SUPT5H PE=3 SV=1	CAVPO	14
385	25.43	27.8	57.04	46.24	41.08	tr|H0VKA3|H0VKA3_CAVPO	Uncharacterized protein OS=Cavia porcellus GN=SEPT6 PE=3 SV=1	CAVPO	15

386	25.31	29.5	60.14	58.78	53.38	tr|H0VWR2|H0VWR2_CAVPO	Uncharacterized protein OS=Cavia porcellus PE=4 SV=1	CAVPO	35
387	25.2	25.2	51.27	49.01	45.07	tr|H0UUJ4|H0UUJ4_CAVPO	Uncharacterized protein OS=Cavia porcellus GN=GNAZ PE=4 SV=1	CAVPO	12
388	25.16	25.2	61.22	57.41	57.41	tr|H0UUB7|H0UUB7_CAVPO	Uncharacterized protein OS=Cavia porcellus GN=CALB1 PE=4 SV=1	CAVPO	17
389	25.12	25.2	43.35	36.24	36.24	tr|H0VLH9|H0VLH9_CAVPO	Uncharacterized protein (Fragment) OS=Cavia porcellus GN=AP2M1 PE=4 SV=1	CAVPO	14
390	25.11	25.1	33.99	33.6	33.6	tr|H0UZ00|H0UZ00_CAVPO	Uncharacterized protein OS=Cavia porcellus GN=PCYOX1 PE=4 SV=1	CAVPO	17
391	25.09	25.2	29.62	24.76	18.86	tr|H0V163|H0V163_CAVPO	Uncharacterized protein OS=Cavia porcellus GN=ANK2 PE=4 SV=1	CAVPO	14
392	25.03	25.1	31.55	25.12	24.02	tr|H0VQ41|H0VQ41_CAVPO	Protein arginine N-methyltransferase 5 OS=Cavia porcellus GN=PRMT5 PE=3 SV=1	CAVPO	13
393	24.99	25	49.86	47.09	45.43	tr|H0V393|H0V393_CAVPO	Uncharacterized protein OS=Cavia porcellus GN=ADK PE=4 SV=1	CAVPO	15
394	24.95	25.7	56	46.13	41.6	tr|H0VT29|H0VT29_CAVPO	Uncharacterized protein (Fragment) OS=Cavia porcellus GN=IDH3B PE=3 SV=1	CAVPO	14
395	24.94	25.1	26.28	24.06	16.35	tr|H0UW16|H0UW16_CAVPO	Uncharacterized protein (Fragment) OS=Cavia porcellus GN=FAM120A PE=4 SV=1	CAVPO	15
396	24.94	29.3	69.39	69.39	68.98	tr|H0VW15|H0VW15_CAVPO	Uncharacterized protein OS=Cavia porcellus GN=YWHAZ PE=3 SV=1	CAVPO	31
397	24.82	27.2	28.63	22.42	19.22	tr|H0VBE8|H0VBE8_CAVPO	Uncharacterized protein OS=Cavia porcellus GN=NEFH PE=3 SV=1	CAVPO	21
398	24.76	24.8	75.88	71.36	70.85	tr|H0W0H7|H0W0H7_CAVPO	Uncharacterized protein OS=Cavia porcellus GN=TAGLN3 PE=4 SV=1	CAVPO	17
399	24.7	24.9	18.87	17.64	11.65	tr|H0VBA1|H0VBA1_CAVPO	Uncharacterized protein OS=Cavia porcellus GN=SEC31A PE=4 SV=1	CAVPO	13

400	24.68	24.7	61.44	47.46	47.46	tr|H0VJX7|H0VJX7_CAVPO	Uncharacterized protein OS=Cavia porcellus GN=SRSF1 PE=4 SV=1	CAVPO	14
401	24.67	24.7	43.89	35.74	35.74	tr|H0V7V6|H0V7V6_CAVPO	Uncharacterized protein (Fragment) OS=Cavia porcellus GN=AMPH PE=4 SV=1	CAVPO	14
402	24.65	24.7	55.99	50.14	42.34	tr|H0V8F0|H0V8F0_CAVPO	Uncharacterized protein OS=Cavia porcellus GN=PDHB PE=4 SV=1	CAVPO	17
403	24.56	24.7	52.84	47.16	36.49	tr|H0VFF3|H0VFF3_CAVPO	Uncharacterized protein OS=Cavia porcellus GN=PSMD11 PE=4 SV=1	CAVPO	12
404	24.54	24.7	35.94	31.45	24.93	tr|H0VIA6|H0VIA6_CAVPO	Uncharacterized protein OS=Cavia porcellus GN=GUCY1A3 PE=3 SV=1	CAVPO	13
405	24.52	25.8	55.85	54.63	41.46	tr|H0VRI4|H0VRI4_CAVPO	Platelet-activating factor acetylhydrolase IB subunit alpha OS=Cavia porcellus GN=PAFAH1B1 PE=3 SV=1	CAVPO	12
406	24.51	24.6	48.16	48.16	43.48	tr|H0W5T5|H0W5T5_CAVPO	Uncharacterized protein OS=Cavia porcellus GN=PHB2 PE=4 SV=1	CAVPO	12
407	24.48	24.6	44.28	39.4	38.09	tr|H0USZ7|H0USZ7_CAVPO	Uncharacterized protein OS=Cavia porcellus GN=ALDH6A1 PE=3 SV=1	CAVPO	16
408	24.45	24.5	65.3	58.74	58.74	tr|H0UWH8|H0UWH8_CAVPO	Uncharacterized protein OS=Cavia porcellus GN=IDH3A PE=3 SV=1	CAVPO	22
409	24.45	24.6	48.46	39.96	35.26	tr|H0VRF5|H0VRF5_CAVPO	Uncharacterized protein OS=Cavia porcellus GN=PUF60 PE=4 SV=1	CAVPO	13
410	24.32	24.4	39.92	36.9	27.42	tr|H0V1B4|H0V1B4_CAVPO	Uncharacterized protein (Fragment) OS=Cavia porcellus GN=TOM1L2 PE=4 SV=1	CAVPO	13
411	24.27	24.3	34.84	31.12	31.12	tr|H0VYB6|H0VYB6_CAVPO	Uncharacterized protein OS=Cavia porcellus GN=AGPAT3 PE=4 SV=1	CAVPO	14
412	24.15	24.2	69.31	68.81	68.32	tr|H0VQC2|H0VQC2_CAVPO	Uncharacterized protein (Fragment) OS=Cavia porcellus GN=PRDX1 PE=4 SV=1	CAVPO	21
413	24.14	24.8	43.97	41.38	40.52	tr|H0VKU4|H0VKU4_CAVPO	Uncharacterized protein OS=Cavia porcellus	CAVPO	17

							GN=ATP6V1H PE=4 SV=1		
414	24.12	28.2	50.54	50.54	46.77	tr|H0V7J4|H0V7J4_CAVPO	Histone H2A OS=Cavia porcellus GN=H2AFY2 PE=3 SV=1	CAVPO	16
415	24.04	24.1	55.32	48.14	46.01	tr|H0VIE4|H0VIE4_CAVPO	Uncharacterized protein OS=Cavia porcellus GN=ACTR1A PE=3 SV=1	CAVPO	13
416	24.02	24	27.24	25	22.15	tr|H0UUD0|H0UUD0_CAVPO	Uncharacterized protein OS=Cavia porcellus GN=SLC2A1 PE=3 SV=1	CAVPO	24
417	23.97	24	60	55.28	50	tr|H0V7F8|H0V7F8_CAVPO	Uncharacterized protein OS=Cavia porcellus GN=PPP1R7 PE=4 SV=1	CAVPO	14
418	23.95	26	16.17	15.04	13.44	tr|H0VE70|H0VE70_CAVPO	Uncharacterized protein OS=Cavia porcellus GN=GNAS PE=4 SV=1	CAVPO	16
419	23.91	26.2	30.5	24.45	20.14	tr|H0VA45|H0VA45_CAVPO	Uncharacterized protein OS=Cavia porcellus GN=HSPH1 PE=3 SV=1	CAVPO	15
420	23.88	24	57.82	52.79	50.28	tr|H0VPB0|H0VPB0_CAVPO	Uncharacterized protein OS=Cavia porcellus GN=RBM4B PE=4 SV=1	CAVPO	13
421	23.87	26.1	36.12	32.29	28.05	tr|H0V4E3|H0V4E3_CAVPO	Uncharacterized protein OS=Cavia porcellus GN=TRAP1 PE=3 SV=1	CAVPO	14
422	23.84	23.9	33.18	31.61	27.7	tr|H0VJU8|H0VJU8_CAVPO	Acetyltransferase component of pyruvate dehydrogenase complex OS=Cavia porcellus GN=DLAT PE=3 SV=1	CAVPO	15
423	23.83	23.9	35.84	25.78	24.36	tr|H0VT81|H0VT81_CAVPO	Uncharacterized protein OS=Cavia porcellus GN=ADD3 PE=4 SV=1	CAVPO	14
424	23.82	23.9	53.3	50.82	46.7	tr|H0W6E0|H0W6E0_CAVPO	Uncharacterized protein (Fragment) OS=Cavia porcellus GN=VAT1 PE=4 SV=1	CAVPO	15
425	23.7	23.8	49.42	49.42	49.42	tr|H0WC55|H0WC55_CAVPO	Uncharacterized protein OS=Cavia porcellus GN=PRPH2 PE=4 SV=1	CAVPO	19
426	23.69	23.7	44.47	42.75	42.75	tr|H0VCH6|H0VCH6_CAVPO	Uncharacterized protein OS=Cavia porcellus GN=CTSD PE=3 SV=1	CAVPO	15

427	23.69	23.7	68.81	68.81	63.86	tr|H0VUX6|H0VUX6_CAVPO	Uncharacterized protein (Fragment) OS=Cavia porcellus GN=GUK1 PE=4 SV=1	CAVPO	15
428	23.66	23.7	48.37	41.3	41.3	tr|H0UU43|H0UU43_CAVPO	Uncharacterized protein (Fragment) OS=Cavia porcellus GN=ADH5 PE=3 SV=1	CAVPO	16
429	23.66	23.8	69.59	61.15	61.15	tr|H0V3Y5|H0V3Y5_CAVPO	Uncharacterized protein (Fragment) OS=Cavia porcellus GN=LOC100723257 PE=3 SV=1	CAVPO	14
430	23.63	23.7	38.58	37.07	30.39	tr|H0V674|H0V674_CAVPO	Adenylyl cyclase-associated protein OS=Cavia porcellus GN=CAP1 PE=3 SV=1	CAVPO	12
431	23.61	23.7	36.96	36.96	35.02	tr|H0V8Z1|H0V8Z1_CAVPO	Uncharacterized protein OS=Cavia porcellus GN=SCRN1 PE=4 SV=1	CAVPO	15
432	23.51	23.6	30.22	28.71	25.54	tr|H0V6J1|H0V6J1_CAVPO	Uncharacterized protein (Fragment) OS=Cavia porcellus GN=EIF3L PE=3 SV=1	CAVPO	13
433	23.49	23.6	54.63	51.42	41.4	tr|H0V1M7|H0V1M7_CAVPO	Uncharacterized protein (Fragment) OS=Cavia porcellus GN=PTBP2 PE=4 SV=1	CAVPO	14
434	23.46	23.5	48.92	43.61	39.04	tr|H0VBM4|H0VBM4_CAVPO	Uncharacterized protein OS=Cavia porcellus GN=APMAP PE=4 SV=1	CAVPO	14
435	23.45	23.5	59.19	54.86	51.62	tr|H0V269|H0V269_CAVPO	Uncharacterized protein OS=Cavia porcellus GN=NSFL1C PE=4 SV=1	CAVPO	14
436	23.41	23.4	56.18	54.31	46.82	tr|H0VSV5|H0VSV5_CAVPO	Proteasome subunit alpha type (Fragment) OS=Cavia porcellus GN=Psma1 PE=3 SV=1	CAVPO	12
437	23.41	29.7	72.94	68.53	68.53	tr|H0W2W3|H0W2W3_CAVPO	Uncharacterized protein OS=Cavia porcellus GN=GNB3 PE=4 SV=1	CAVPO	21
438	23.4	26.1	67.25	63.76	60.7	tr|H0VEL1|H0VEL1_CAVPO	Uncharacterized protein (Fragment) OS=Cavia porcellus GN=GSTM3 PE=3 SV=1	CAVPO	16
439	23.36	23.4	65.61	65.08	65.08	tr|H0V8R3|H0V8R3_CAVPO	Uncharacterized protein OS=Cavia porcellus GN=PARK7 PE=4 SV=1	CAVPO	19
440	23.32	23.4	39.44	30.85	20.14	tr|H0VLA8|H0VLA8_CAVPO	Uncharacterized protein OS=Cavia porcellus GN=PREP PE=4 SV=1	CAVPO	12

441	23.32	23.7	38.84	38.66	31.94	tr|H0VNC0|H0VNC0_CAVPO	Uncharacterized protein (Fragment) OS=Cavia porcellus GN=GRK7 PE=3 SV=1	CAVPO	13
442	23.31	24.3	55.27	51.41	40.62	tr|H0WAK4|H0WAK4_CAVPO	Uncharacterized protein OS=Cavia porcellus GN=LOC101788057 PE=3 SV=1	CAVPO	12
443	23.27	25.3	34.56	30.87	30.87	tr|H0VFS3|H0VFS3_CAVPO	Inosine-5'-monophosphate dehydrogenase (Fragment) OS=Cavia porcellus GN=IMPDH2 PE=3 SV=1	CAVPO	14
444	23.24	23.5	51.45	45.41	38.89	tr|H0VAM2|H0VAM2_CAVPO	Uncharacterized protein (Fragment) OS=Cavia porcellus GN=VAT1L PE=4 SV=1	CAVPO	13
445	23.23	23.3	67.07	65.45	52.44	tr|H0V034|H0V034_CAVPO	Proteasome subunit alpha type OS=Cavia porcellus GN=PSMA6 PE=3 SV=1	CAVPO	13
446	23.19	23.4	20.11	12.13	6.984	tr|H0V983|H0V983_CAVPO	Uncharacterized protein OS=Cavia porcellus GN=CKAP5 PE=4 SV=1	CAVPO	12
447	23.18	23.4	44.47	38.83	32.1	tr|H0VDD0|H0VDD0_CAVPO	Uncharacterized protein (Fragment) OS=Cavia porcellus GN=RNH1 PE=4 SV=1	CAVPO	14
448	23.07	23.2	74.32	74.32	74.32	tr|H0VKV0|H0VKV0_CAVPO	Uncharacterized protein (Fragment) OS=Cavia porcellus GN=Calm1 PE=4 SV=1	CAVPO	33
449	23	25.4	48.01	45.8	29.87	tr|H0VCH1|H0VCH1_CAVPO	Isocitrate dehydrogenase [NADP] OS=Cavia porcellus GN=IDH2 PE=3 SV=1	NADP	14
450	22.99	23.1	46.4	32.36	21.23	tr|H0VLR8|H0VLR8_CAVPO	Malic enzyme OS=Cavia porcellus GN=ME2 PE=3 SV=1	CAVPO	11
451	22.97	23	36.18	36.18	36.18	tr|H0VD81|H0VD81_CAVPO	Uncharacterized protein OS=Cavia porcellus GN=Anp32a PE=4 SV=1	CAVPO	21
452	22.93	23	65.74	65.12	63.58	tr|B5ABI3|B5ABI3_CAVPO	Uncharacterized protein OS=Cavia porcellus GN=YB1 PE=2 SV=1	CAVPO	15
453	22.87	22.9	51.12	50.37	45.66	tr|H0UUS6|H0UUS6_CAVPO	Uncharacterized protein (Fragment) OS=Cavia porcellus GN=ACTR3 PE=3 SV=1	CAVPO	12
454	22.84	22.9	52.35	45.13	42.6	tr|H0W9R6|H0W9R6_CAVPO	Uncharacterized protein OS=Cavia porcellus	CAVPO	12

							GN=IMPA1 PE=4 SV=1		
455	22.81	22.9	51.45	47.91	42.44	tr|H0V615|H0V615_CAVPO	Uncharacterized protein OS=Cavia porcellus GN=NAPG PE=4 SV=1	CAVPO	12
456	22.78	22.8	45.15	38.5	36.57	tr|H0VJK3|H0VJK3_CAVPO	Uncharacterized protein OS=Cavia porcellus GN=SLC25A3 PE=3 SV=1	CAVPO	20
457	22.77	22.9	31.62	20.29	17	tr|H0VA86|H0VA86_CAVPO	Uncharacterized protein OS=Cavia porcellus GN=CUL4A PE=3 SV=1	CAVPO	11
458	22.73	22.8	33.93	27.78	27.78	tr|H0VKG3|H0VKG3_CAVPO	Uncharacterized protein OS=Cavia porcellus GN=API5 PE=4 SV=1	CAVPO	12
459	22.73	22.8	22.45	18.3	16.28	tr|H0VP57|H0VP57_CAVPO	Uncharacterized protein OS=Cavia porcellus GN=IPO7 PE=4 SV=1	CAVPO	12
460	22.63	22.7	54.46	54.46	51.49	tr|H0VZ24|H0VZ24_CAVPO	6-phosphogluconate dehydrogenase, decarboxylating (Fragment) OS=Cavia porcellus GN=PGD PE=3 SV=1	CAVPO	13
461	22.62	23.1	56.19	53.65	42.86	tr|H0V0K2|H0V0K2_CAVPO	Uncharacterized protein OS=Cavia porcellus GN=EIF2S1 PE=4 SV=1	CAVPO	12
462	22.52	22.6	34.83	29.96	23.03	tr|H0VSM1|H0VSM1_CAVPO	Uncharacterized protein OS=Cavia porcellus GN=PSMD3 PE=4 SV=1	CAVPO	10
463	22.45	22.6	47.56	37.04	29.82	tr|H0V7A0|H0V7A0_CAVPO	Uncharacterized protein OS=Cavia porcellus GN=SMU1 PE=4 SV=1	CAVPO	13
464	22.36	22.4	54.04	54.04	51.55	tr|H0V9K1|H0V9K1_CAVPO	Uncharacterized protein (Fragment) OS=Cavia porcellus GN=PDXK PE=4 SV=1	CAVPO	13
465	22.35	56.6	46.16	40.51	35.5	tr|H0V545|H0V545_CAVPO	Uncharacterized protein OS=Cavia porcellus GN=AP1B1 PE=4 SV=1	CAVPO	30
466	22.31	22.4	51.5	45.96	34.64	tr|H0VW72|H0VW72_CAVPO	Uncharacterized protein OS=Cavia porcellus GN=PSMC2 PE=3 SV=1	CAVPO	13
467	22.3	22.3	40.67	40.67	31.91	tr|H0VHQ8|H0VHQ8_CAVPO	Uncharacterized protein (Fragment) OS=Cavia porcellus GN=PDIA6 PE=3 SV=1	CAVPO	12

468	22.3	22.4	31.57	26.29	22.14	tr|H0VWP6|H0VWP6_CAVPO	Uncharacterized protein (Fragment) OS=Cavia porcellus GN=CAPN2 PE=4 SV=1	CAVPO	11
469	22.29	22.5	23.74	17.04	14.78	tr|H0V2G0|H0V2G0_CAVPO	Uncharacterized protein OS=Cavia porcellus GN=CCAR1 PE=4 SV=1	CAVPO	14
470	22.22	22.3	31.53	28.83	28.83	tr|H0W767|H0W767_CAVPO	Uncharacterized protein (Fragment) OS=Cavia porcellus GN=RANBP3 PE=4 SV=1	CAVPO	13
471	22.18	22.2	43.11	37.11	37.11	tr|H0V542|H0V542_CAVPO	Uncharacterized protein (Fragment) OS=Cavia porcellus GN=Gsr PE=4 SV=1	CAVPO	12
472	22.16	22.3	33.83	28.8	23.04	tr|H0VMW5|H0VMW5_CAVPO	Uncharacterized protein OS=Cavia porcellus GN=RGS9 PE=4 SV=1	CAVPO	15
473	22.13	22.3	72.02	67.49	52.67	tr|H0V3N5|H0V3N5_CAVPO	Uncharacterized protein OS=Cavia porcellus GN=Rps3 PE=3 SV=1	CAVPO	11
474	22.08	22.2	47.59	44.48	43.91	tr|H0W951|H0W951_CAVPO	Uncharacterized protein OS=Cavia porcellus GN=PRMT1 PE=4 SV=1	CAVPO	13
475	22.04	23.2	50.64	46.8	36.83	tr|H0V659|H0V659_CAVPO	Uncharacterized protein OS=Cavia porcellus GN=Csnk2a1 PE=4 SV=1	CAVPO	12
476	22.03	22	70.83	65.83	65.83	tr|H0VZK8|H0VZK8_CAVPO	Uncharacterized protein (Fragment) OS=Cavia porcellus GN=DDAH1 PE=4 SV=1	CAVPO	12
477	22	22	54.05	51.13	51.13	tr|H0V4U3|H0V4U3_CAVPO	Serine/threonine-protein phosphatase OS=Cavia porcellus GN=PPP2CA PE=3 SV=1	CAVPO	13
478	22	22.1	43.55	39.52	36.69	tr|H0W1D6|H0W1D6_CAVPO	Uncharacterized protein OS=Cavia porcellus GN=TPM3 PE=3 SV=1	CAVPO	12
479	21.94	22	63.21	43.63	37.74	tr|Q8K1W3|Q8K1W3_CAVPO	Interphotoreceptor retinoid binding protein (Fragment) OS=Cavia porcellus GN=irbp PE=4 SV=1	CAVPO	12
480	21.86	21.9	63.76	63.76	63.3	tr|H0UYR4|H0UYR4_CAVPO	Uncharacterized protein (Fragment) OS=Cavia porcellus GN=HPRT1 PE=4 SV=1	CAVPO	12
481	21.84	22	30.98	25.84	19.26	tr|H0VKJ2|H0VKJ2_CAVPO	Lysine--tRNA ligase OS=Cavia porcellus	CAVPO	11

							GN=KARS PE=3 SV=1		
482	21.82	21.9	47.42	41.8	33.03	tr|H0VA43|H0VA43_CAVPO	Uncharacterized protein (Fragment) OS=Cavia porcellus GN=ERO1L PE=4 SV=1	CAVPO	12
483	21.8	22.5	49.38	41.94	33.26	tr|H0V3H0|H0V3H0_CAVPO	Serine hydroxymethyltransferase OS=Cavia porcellus GN=SHMT1 PE=1 SV=1	CAVPO	12
484	21.8	21.9	31.36	31.36	28.14	tr|H0VU30|H0VU30_CAVPO	Uncharacterized protein OS=Cavia porcellus GN=NT5C2 PE=3 SV=1	CAVPO	13
485	21.79	22.2	57.64	53.38	34.84	tr|H0VEM1|H0VEM1_CAVPO	Uncharacterized protein OS=Cavia porcellus GN=LANCL1 PE=4 SV=1	CAVPO	16
486	21.77	21.9	34.33	24.84	18.35	tr|H0W9W4|H0W9W4_CAVPO	Uncharacterized protein (Fragment) OS=Cavia porcellus GN=RRBP1 PE=4 SV=1	CAVPO	13
487	21.7	21.8	36.76	24.87	18.86	tr|H0V5X7|H0V5X7_CAVPO	Uncharacterized protein OS=Cavia porcellus GN=DDX42 PE=3 SV=1	CAVPO	12
488	21.69	21.7	31.49	31.23	30.98	tr|H0VNZ8|H0VNZ8_CAVPO	Uncharacterized protein OS=Cavia porcellus GN=NPTN PE=4 SV=1	CAVPO	13
489	21.67	22	73.16	65.81	50	tr|H0UTX5|H0UTX5_CAVPO	Uncharacterized protein OS=Cavia porcellus GN=PHB PE=4 SV=1	CAVPO	12
490	21.67	21.7	41.87	34.52	32.94	tr|H0VQT4|H0VQT4_CAVPO	Uncharacterized protein (Fragment) OS=Cavia porcellus GN=HDAC2 PE=4 SV=1	CAVPO	13
491	21.62	21.8	49.65	45.41	42.35	tr|H0VT57|H0VT57_CAVPO	Uncharacterized protein OS=Cavia porcellus GN=Rbbp4 PE=4 SV=1	CAVPO	15
492	21.6	21.8	20.02	15.64	10.51	tr|H0V477|H0V477_CAVPO	Uncharacterized protein (Fragment) OS=Cavia porcellus GN=Nomo1 PE=4 SV=1	CAVPO	10
493	21.57	21.6	74.7	65.06	65.06	tr|H0VBH0|H0VBH0_CAVPO	Uncharacterized protein (Fragment) OS=Cavia porcellus GN=CFL1 PE=4 SV=1	CAVPO	12
494	21.42	21.5	32.41	28.01	26.71	tr|H0VP14|H0VP14_CAVPO	Uncharacterized protein OS=Cavia porcellus GN=AIFM1 PE=4 SV=1	CAVPO	12

495	21.41	21.6	28.84	24.43	21.02	tr|H0VB69|H0VB69_CAVPO	Mitochondrial Rho GTPase OS=Cavia porcellus GN=RHOT1 PE=3 SV=1	CAVPO	12
496	21.36	21.4	53.85	47.93	47.93	tr|H0W737|H0W737_CAVPO	Uncharacterized protein OS=Cavia porcellus GN=COX4I1 PE=4 SV=1	CAVPO	14
497	21.35	21.4	34.69	33.39	29.7	tr|H0V1J1|H0V1J1_CAVPO	Uncharacterized protein OS=Cavia porcellus GN=TERF2 PE=4 SV=1	CAVPO	12
498	21.35	21.4	43.19	35.73	30.85	tr|H0V759|H0V759_CAVPO	Uncharacterized protein OS=Cavia porcellus GN=PSMD6 PE=4 SV=1	CAVPO	11
499	21.33	21.4	15.98	14.83	12.16	tr|H0V7Q6|H0V7Q6_CAVPO	Uncharacterized protein OS=Cavia porcellus GN=NRCAM PE=4 SV=1	CAVPO	10
500	21.31	21.5	46.77	38.75	23.87	tr|H0W849|H0W849_CAVPO	Uncharacterized protein (Fragment) OS=Cavia porcellus GN=ARCN1 PE=4 SV=1	CAVPO	9
501	21.26	21.4	22.35	18.16	12.68	tr|H0V0S3|H0V0S3_CAVPO	Uncharacterized protein OS=Cavia porcellus GN=SEC24C PE=4 SV=1	CAVPO	10
502	21.23	21.3	49.04	41.4	41.4	tr|H0V4G6|H0V4G6_CAVPO	Uncharacterized protein OS=Cavia porcellus GN=SLC25A11 PE=3 SV=1	CAVPO	12
503	21.2	21.3	18.7	14.52	11.7	tr|H0VFP3|H0VFP3_CAVPO	Uncharacterized protein (Fragment) OS=Cavia porcellus GN=XPO5 PE=4 SV=1	CAVPO	12
504	21.18	21.3	71.68	69.58	63.64	tr|Q07E10|Q07E10_CAVPO	Capping protein (Actin filament) muscle Z-line, alpha 2 OS=Cavia porcellus GN=CAPZA2 PE=4 SV=1	CAVPO	12
505	21.17	21.2	24.01	21.01	19.24	tr|H0VPQ2|H0VPQ2_CAVPO	Uncharacterized protein OS=Cavia porcellus GN=LSS PE=4 SV=1	CAVPO	11
506	21.16	21.2	46.48	44.79	40	tr|H0VG68|H0VG68_CAVPO	Uncharacterized protein OS=Cavia porcellus GN=NDUFA10 PE=4 SV=1	CAVPO	10
507	21.16	21.2	35.97	32.91	32.91	tr|H0VKB7|H0VKB7_CAVPO	Uncharacterized protein OS=Cavia porcellus GN=Luc7l2 PE=4 SV=1	CAVPO	11
508	21.14	21.2	20.26	14.92	11.46	tr|H0VD57|H0VD57_CAVPO	Uncharacterized protein OS=Cavia porcellus	CAVPO	12

							GN=NUP155 PE=4 SV=1		
509	21.07	21.1	50	50	46.75	tr|H0V181|H0V181_CAVPO	Uncharacterized protein OS=Cavia porcellus GN=BPNT1 PE=4 SV=1	CAVPO	11
510	21.03	23.4	35.09	31.32	20.94	tr|H0VJK0|H0VJK0_CAVPO	Adenosylhomocysteinase OS=Cavia porcellus GN=AHCYL1 PE=3 SV=1	CAVPO	11
511	20.97	23	51.66	51.29	46.13	tr|H0VGS0|H0VGS0_CAVPO	Uncharacterized protein OS=Cavia porcellus GN=CALB2 PE=4 SV=1	CAVPO	20
512	20.95	21.1	45.29	34.86	29.26	tr|H0UZY7|H0UZY7_CAVPO	Uncharacterized protein OS=Cavia porcellus GN=MAP2K1 PE=4 SV=1	CAVPO	13
513	20.93	21	57.06	52.35	45.15	tr|H0VPA8|H0VPA8_CAVPO	Uncharacterized protein (Fragment) OS=Cavia porcellus GN=SEPT2 PE=3 SV=1	CAVPO	13
514	20.92	21	44.21	38.14	28.27	tr|H0VLR7|H0VLR7_CAVPO	Catalase OS=Cavia porcellus GN=Mip PE=3 SV=1	CAVPO	12
515	20.91	21	39.71	37.8	32.3	tr|H0W5L8|H0W5L8_CAVPO	Uncharacterized protein OS=Cavia porcellus GN=SERPINH1 PE=3 SV=1	CAVPO	12
516	20.9	20.9	32.37	32.37	32.37	tr|H0V2J0|H0V2J0_CAVPO	Uncharacterized protein (Fragment) OS=Cavia porcellus GN=NCEH1 PE=4 SV=1	CAVPO	11
517	20.84	20.9	36.38	34.32	31.12	tr|H0VFI7|H0VFI7_CAVPO	Serine/threonine-protein phosphatase (Fragment) OS=Cavia porcellus GN=PPP3CA PE=3 SV=1	CAVPO	13
518	20.77	20.8	70.83	65.1	65.1	tr|H0V534|H0V534_CAVPO	Uncharacterized protein (Fragment) OS=Cavia porcellus GN=AK1 PE=3 SV=1	CAVPO	11
519	20.71	20.8	47.15	47.15	47.15	tr|H0VH33|H0VH33_CAVPO	Uncharacterized protein (Fragment) OS=Cavia porcellus GN=ERP29 PE=4 SV=1	CAVPO	12
520	20.69	20.9	24.84	12.82	11.39	tr|H0V7G7|H0V7G7_CAVPO	Uncharacterized protein OS=Cavia porcellus GN=HDLBP PE=4 SV=1	CAVPO	11
521	20.69	20.8	49.59	39.84	39.84	tr|H0VCJ0|H0VCJ0_CAVPO	Uncharacterized protein OS=Cavia porcellus GN=BCAP31 PE=4 SV=1	CAVPO	12

522	20.68	20.8	36.53	35.13	28.54	tr|H0VCU8|H0VCU8_CAVPO	Uncharacterized protein OS=Cavia porcellus GN=SF3A3 PE=4 SV=1	CAVPO	13
523	20.65	30.8	32.54	30.74	28.35	tr|H0V4A5|H0V4A5_CAVPO	Uncharacterized protein (Fragment) OS=Cavia porcellus GN=LOC100724384 PE=4 SV=1	CAVPO	19
524	20.64	21.7	27.2	21.54	19.93	tr|H0UXL7|H0UXL7_CAVPO	Uncharacterized protein OS=Cavia porcellus GN=BAG6 PE=4 SV=1	CAVPO	13
525	20.63	20.8	22.68	19.95	13.3	tr|H0V7I9|H0V7I9_CAVPO	Uncharacterized protein (Fragment) OS=Cavia porcellus GN=PC PE=3 SV=1	CAVPO	10
526	20.59	20.8	31.22	25.34	18.55	tr|H0V7G5|H0V7G5_CAVPO	Uncharacterized protein (Fragment) OS=Cavia porcellus GN=GBE1 PE=4 SV=1	CAVPO	12
527	20.57	20.7	30	24.86	16.62	tr|H0V4I3|H0V4I3_CAVPO	Uncharacterized protein OS=Cavia porcellus GN=DCLK1 PE=4 SV=1	CAVPO	12
528	20.5	20.6	49.53	47.35	42.99	tr|H0UWE9|H0UWE9_CAVPO	Uncharacterized protein OS=Cavia porcellus GN=SLC25A22 PE=3 SV=1	CAVPO	12
529	20.5	20.5	55.99	55.99	55.99	tr|H0VP58|H0VP58_CAVPO	Uncharacterized protein OS=Cavia porcellus GN=COPE PE=4 SV=1	CAVPO	12
530	20.48	21.6	25.75	18.35	11.26	tr|H0UUX4|H0UUX4_CAVPO	Uncharacterized protein OS=Cavia porcellus GN=VARS PE=3 SV=1	CAVPO	11
531	20.48	20.5	80.47	72.56	66.98	tr|H0V8C8|H0V8C8_CAVPO	Uncharacterized protein OS=Cavia porcellus GN=RAB14 PE=3 SV=1	CAVPO	14
532	20.47	22.5	64.16	55.2	53.18	tr|H0V7P5|H0V7P5_CAVPO	Uncharacterized protein OS=Cavia porcellus GN=HNRNPH3 PE=4 SV=1	CAVPO	13
533	20.44	20.5	29.7	28.04	26.75	tr|H0V5J4|H0V5J4_CAVPO	Uncharacterized protein (Fragment) OS=Cavia porcellus GN=IGF2BP2 PE=4 SV=1	CAVPO	11
534	20.43	20.6	21.05	14.86	9.677	tr|H0VL70|H0VL70_CAVPO	Uncharacterized protein (Fragment) OS=Cavia porcellus GN=LARS PE=3 SV=1	CAVPO	9
535	20.41	20.5	39.27	33.2	28.54	tr|H0VN88|H0VN88_CAVPO	Uncharacterized protein OS=Cavia porcellus GN=GPHN PE=4 SV=1	CAVPO	11

536	20.39	20.6	7.271	2.698	1.772	tr|H0VC35|H0VC35_CAVPO	Uncharacterized protein (Fragment) OS=Cavia porcellus GN=SYNE1 PE=4 SV=1	CAVPO	11
537	20.36	20.5	33.83	32.15	28.97	tr|H0UUF3|H0UUF3_CAVPO	Uncharacterized protein OS=Cavia porcellus GN=TROVE2 PE=4 SV=1	CAVPO	12
538	20.36	20.5	32.21	28.46	26.88	tr|Q99NA8|Q99NA8_CAVPO	Putative monoamine oxidase A (Fragment) OS=Cavia porcellus GN=MAO-A PE=2 SV=1	CAVPO	12
539	20.35	20.5	36.89	31.77	23.45	tr|H0V874|H0V874_CAVPO	Uncharacterized protein OS=Cavia porcellus GN=RUFY3 PE=4 SV=1	CAVPO	11
540	20.35	20.4	32.58	25.44	20.56	tr|H0VMS7|H0VMS7_CAVPO	Microtubule-associated protein (Fragment) OS=Cavia porcellus GN=MAPT PE=3 SV=1	CAVPO	12
541	20.29	20.5	37.82	32.63	23.21	tr|H0W345|H0W345_CAVPO	Uncharacterized protein OS=Cavia porcellus GN=KLC1 PE=4 SV=1	CAVPO	13
542	20.23	20.3	54.13	54.13	54.13	tr|H0W9A0|H0W9A0_CAVPO	Uncharacterized protein OS=Cavia porcellus GN=RAB11B PE=3 SV=1	CAVPO	11
543	20.19	20.2	74.45	72.99	69.34	tr|H0UWI7|H0UWI7_CAVPO	Uncharacterized protein OS=Cavia porcellus GN=CRABP1 PE=3 SV=1	CAVPO	12
544	20.17	20.2	34.84	26.97	24.61	tr|H0VCE7|H0VCE7_CAVPO	Uncharacterized protein (Fragment) OS=Cavia porcellus GN=UGP2 PE=4 SV=1	CAVPO	10
545	20.15	20.3	19.62	14.99	10.72	tr|H0VK83|H0VK83_CAVPO	Uncharacterized protein (Fragment) OS=Cavia porcellus GN=NCKAP1 PE=4 SV=1	CAVPO	10
546	20.14	20.2	21.58	19.09	19.09	tr|H0W523|H0W523_CAVPO	Uncharacterized protein OS=Cavia porcellus GN=SLC16A1 PE=4 SV=1	CAVPO	19
547	20.09	20.2	33.07	28.84	23.82	tr|H0VRW8|H0VRW8_CAVPO	Uncharacterized protein OS=Cavia porcellus GN=DYNC1I2 PE=4 SV=1	CAVPO	10
548	20.08	20.2	48.22	45.27	45.27	tr|H0VA29|H0VA29_CAVPO	Delta-aminolevulinic acid dehydratase (Fragment) OS=Cavia porcellus GN=ALAD PE=3 SV=1	CAVPO	13
549	20.07	20.1	56.97	56.97	50.82	tr|H0UVY4|H0UVY4_CAVPO	Uncharacterized protein OS=Cavia porcellus	CAVPO	10

							GN=C14orf166 PE=4 SV=1		
550	20	20	53.37	53.37	53.37	tr|H0UYP7|H0UYP7_CAVPO	Uncharacterized protein OS=Cavia porcellus GN=HPCA PE=4 SV=1	CAVPO	10
551	20	20	66.03	66.03	66.03	tr|H0V278|H0V278_CAVPO	Uncharacterized protein OS=Cavia porcellus GN=ATP5H PE=4 SV=1	CAVPO	12
552	19.9	20	28.44	23.67	14	tr|H0VMF1|H0VMF1_CAVPO	Uncharacterized protein OS=Cavia porcellus GN=MARS PE=3 SV=1	CAVPO	10
553	19.9	20	45.7	43.32	36.8	tr|H0W1P5|H0W1P5_CAVPO	Uncharacterized protein (Fragment) OS=Cavia porcellus GN=PRKACA PE=4 SV=1	CAVPO	11
554	19.88	19.9	43.52	37.72	31.91	tr|H0VN31|H0VN31_CAVPO	Uncharacterized protein OS=Cavia porcellus GN=SLC3A2 PE=4 SV=1	CAVPO	13
555	19.84	19.9	50.48	46.03	45.4	tr|H0VGC8|H0VGC8_CAVPO	Uncharacterized protein OS=Cavia porcellus GN=Calu PE=4 SV=1	CAVPO	12
556	19.84	19.9	33.97	30.53	24.81	tr|H0VJK8|H0VJK8_CAVPO	Uncharacterized protein OS=Cavia porcellus GN=DYNC1LI1 PE=4 SV=1	CAVPO	10
557	19.84	20	53.96	37.13	34.16	tr|H0W511|H0W511_CAVPO	Uncharacterized protein OS=Cavia porcellus GN=COPS4 PE=4 SV=1	CAVPO	10
558	19.82	19.9	56.2	45.74	45.74	tr|H0VCY8|H0VCY8_CAVPO	Uncharacterized protein (Fragment) OS=Cavia porcellus GN=Rpsa PE=3 SV=1	CAVPO	12
559	19.77	19.9	25.98	19.08	15.75	tr|H0VX70|H0VX70_CAVPO	Coatomer subunit gamma OS=Cavia porcellus GN=COPG1 PE=3 SV=1	CAVPO	11
560	19.73	52.3	50.78	41.93	33.97	tr|H0UVG6|H0UVG6_CAVPO	Uncharacterized protein OS=Cavia porcellus GN=ACTN1 PE=4 SV=1	CAVPO	26
561	19.73	19.8	42.34	42.34	32.31	tr|H0UVW8|H0UVW8_CAVPO	Uncharacterized protein OS=Cavia porcellus GN=NANS PE=4 SV=1	CAVPO	13
562	19.7	21.9	28.45	28.45	20.17	tr|H0V628|H0V628_CAVPO	Uncharacterized protein OS=Cavia porcellus GN=ADD2 PE=4 SV=1	CAVPO	11

563	19.69	19.8	30.7	28.19	22.8	tr|H0VA38|H0VA38_CAVPO	Uncharacterized protein OS=Cavia porcellus GN=NT5DC2 PE=3 SV=1	CAVPO	10
564	19.67	19.8	36.06	29.82	26.12	tr|H0VXY7|H0VXY7_CAVPO	Uncharacterized protein (Fragment) OS=Cavia porcellus GN=PRKCSH PE=4 SV=1	CAVPO	10
565	19.63	19.7	72.78	65.82	64.56	tr|H0W0N2|H0W0N2_CAVPO	Uncharacterized protein OS=Cavia porcellus GN=CPLX3 PE=4 SV=1	CAVPO	11
566	19.59	19.6	32.04	29.18	27.35	tr|H0VUD6|H0VUD6_CAVPO	Microtubule-associated protein (Fragment) OS=Cavia porcellus GN=MAP4 PE=3 SV=1	CAVPO	11
567	19.57	19.6	54.46	52.92	51.08	tr|H0UV39|H0UV39_CAVPO	Uncharacterized protein (Fragment) OS=Cavia porcellus GN=SFXN3 PE=4 SV=1	CAVPO	11
568	19.54	19.6	50.59	41.57	41.57	tr|H0VB82|H0VB82_CAVPO	Uncharacterized protein OS=Cavia porcellus GN=ETFB PE=4 SV=1	CAVPO	10
569	19.51	19.9	43.64	41.95	26.27	tr|H0VJ25|H0VJ25_CAVPO	mRNA cap guanine-N7 methyltransferase OS=Cavia porcellus GN=RNMT PE=3 SV=1	CAVPO	10
570	19.5	19.6	39.5	37.7	30.93	tr|H0V0W1|H0V0W1_CAVPO	Uncharacterized protein OS=Cavia porcellus GN=COPS2 PE=4 SV=1	CAVPO	11
571	19.5	19.6	42.95	41.03	38.14	tr|H0VTB9|H0VTB9_CAVPO	Uncharacterized protein OS=Cavia porcellus GN=HSD17B12 PE=3 SV=1	CAVPO	10
572	19.48	30.6	40.23	24.87	18.7	tr|H0W2U4|H0W2U4_CAVPO	Uncharacterized protein OS=Cavia porcellus GN=KIF5C PE=4 SV=1	CAVPO	15
573	19.46	19.5	47.81	43.96	41.13	tr|H0V6E6|H0V6E6_CAVPO	Uncharacterized protein (Fragment) OS=Cavia porcellus GN=ILF2 PE=4 SV=1	CAVPO	10
574	19.43	19.6	56.5	53.25	46.75	tr|H0UTH1|H0UTH1_CAVPO	Phosphomannomutase OS=Cavia porcellus GN=PMM2 PE=3 SV=1	CAVPO	12
575	19.43	25.5	55.93	55.93	55.93	tr|H0VIX2|H0VIX2_CAVPO	Uncharacterized protein OS=Cavia porcellus GN=YWHAQ PE=3 SV=1	CAVPO	19
576	19.38	19.6	35.12	30.62	28.2	tr|H0VFU0|H0VFU0_CAVPO	Uncharacterized protein (Fragment) OS=Cavia porcellus GN=DAK PE=4 SV=1	CAVPO	10

577	19.37	22.5	28.27	21.5	20.68	tr|H0VHX8|H0VHX8_CAVPO	Uncharacterized protein (Fragment) OS=Cavia porcellus GN=DLG2 PE=4 SV=1	CAVPO	13
578	19.36	19.5	52.13	49.65	42.2	tr|H0VIE3|H0VIE3_CAVPO	Uncharacterized protein OS=Cavia porcellus GN=ESD PE=4 SV=1	CAVPO	12
579	19.3	19.3	45.3	45.3	45.3	tr|H0VX59|H0VX59_CAVPO	Uncharacterized protein OS=Cavia porcellus GN=ROM1 PE=4 SV=1	CAVPO	16
580	19.29	19.5	23.81	15.87	11.11	tr|H0VQH0|H0VQH0_CAVPO	Uncharacterized protein OS=Cavia porcellus GN=MYBBP1A PE=4 SV=1	CAVPO	10
581	19.28	19.4	47.33	47.33	34.33	tr|H0V6J2|H0V6J2_CAVPO	Uncharacterized protein OS=Cavia porcellus GN=TCEA1 PE=4 SV=1	CAVPO	11
582	19.26	19.4	35.12	30.77	21.4	tr|H0VT25|H0VT25_CAVPO	Uncharacterized protein (Fragment) OS=Cavia porcellus GN=Nop56 PE=4 SV=1	CAVPO	10
583	19.19	19.3	65.31	52.03	40.22	tr|H0VL43|H0VL43_CAVPO	Uncharacterized protein (Fragment) OS=Cavia porcellus GN=PITPNB PE=4 SV=1	CAVPO	11
584	19.18	19.5	60.54	60.15	53.26	tr|H0VEZ1|H0VEZ1_CAVPO	Uncharacterized protein OS=Cavia porcellus GN=HSD17B10 PE=3 SV=1	CAVPO	10
585	19.18	19.3	53.45	49.45	46.18	tr|H0VLY8|H0VLY8_CAVPO	Uncharacterized protein OS=Cavia porcellus GN=PROSC PE=1 SV=1	CAVPO	11
586	19.18	19.3	38.07	34.26	33.5	tr|H0VVN0|H0VVN0_CAVPO	Uncharacterized protein OS=Cavia porcellus GN=ACTR2 PE=3 SV=1	CAVPO	11
587	19.09	19.2	35.88	31.85	28.66	tr|H0UUE7|H0UUE7_CAVPO	Uncharacterized protein OS=Cavia porcellus GN=Eif2s3 PE=4 SV=1	CAVPO	10
588	19.09	19.3	69.49	56.99	51.1	tr|H0UUT5|H0UUT5_CAVPO	Uncharacterized protein (Fragment) OS=Cavia porcellus GN=Mtap PE=3 SV=1	CAVPO	10
589	19.09	19.2	58.97	55.31	55.31	tr|H0VJ50|H0VJ50_CAVPO	Uncharacterized protein OS=Cavia porcellus GN=C21orf33 PE=4 SV=1	CAVPO	12
590	19.08	19.2	45.85	34.97	32.9	tr|H0VDC6|H0VDC6_CAVPO	Uncharacterized protein (Fragment) OS=Cavia porcellus GN=HIBCH PE=4 SV=1	CAVPO	10

591	19.04	19.3	24.55	19.55	17.61	tr|H0VKR9|H0VKR9_CAVPO	Uncharacterized protein OS=Cavia porcellus GN=WDR44 PE=4 SV=1	CAVPO	12
592	19	19.1	55.82	51	47.39	tr|H0UUQ6|H0UUQ6_CAVPO	Uncharacterized protein OS=Cavia porcellus GN=PSME1 PE=4 SV=1	CAVPO	11
593	18.98	19.1	29.92	26.06	26.06	tr|H0VEH8|H0VEH8_CAVPO	Uncharacterized protein (Fragment) OS=Cavia porcellus GN=GSPT1 PE=4 SV=1	CAVPO	11
594	18.97	19.1	52.89	52.89	50.22	tr|H0VR64|H0VR64_CAVPO	Uncharacterized protein OS=Cavia porcellus GN=EEF1B2 PE=3 SV=1	CAVPO	13
595	18.95	19.1	31.09	24.18	20.56	tr|Q6WDN9|Q6WDN9_CAVPO	Preproalbumin OS=Cavia porcellus GN=Alb PE=2 SV=1	CAVPO	10
596	18.94	19	32.14	32.14	22.22	tr|H0W9N1|H0W9N1_CAVPO	Uncharacterized protein OS=Cavia porcellus GN=PSMD5 PE=4 SV=1	CAVPO	11
597	18.85	18.9	51.89	46.21	43.18	tr|H0VTN3|H0VTN3_CAVPO	40S ribosomal protein S3a OS=Cavia porcellus GN=RPS3A PE=3 SV=1	CAVPO	11
598	18.83	19	44.55	39	31.05	tr|H0VAG4|H0VAG4_CAVPO	Uncharacterized protein OS=Cavia porcellus GN=HNRNPLL PE=4 SV=1	CAVPO	12
599	18.83	18.9	49.55	46.59	41.25	tr|H0VQU5|H0VQU5_CAVPO	Uncharacterized protein OS=Cavia porcellus GN=DCPS PE=4 SV=1	CAVPO	11
600	18.77	18.9	58.24	48.28	48.28	tr|H0W5S7|H0W5S7_CAVPO	Proteasome subunit alpha type (Fragment) OS=Cavia porcellus GN=PSMA4 PE=3 SV=1	CAVPO	11
601	18.73	18.9	33.71	32.12	28.7	tr|H0VGN9|H0VGN9_CAVPO	Uncharacterized protein OS=Cavia porcellus GN=OAT PE=3 SV=1	CAVPO	10
602	18.7	18.8	40.39	36.72	31.1	tr|H0VQ97|H0VQ97_CAVPO	Uncharacterized protein OS=Cavia porcellus GN=RMDN3 PE=4 SV=1	CAVPO	9
603	18.68	18.7	29.7	27.79	27.79	tr|H0UY79|H0UY79_CAVPO	Uncharacterized protein OS=Cavia porcellus GN=St13 PE=4 SV=1	CAVPO	12
604	18.68	18.8	10.49	6.444	5.108	tr|H0VJM9|H0VJM9_CAVPO	Uncharacterized protein OS=Cavia porcellus GN=MTOR PE=4 SV=1	CAVPO	10

605	18.67	23.9	54.17	50.83	50.83	tr|H0UYE6|H0UYE6_CAVPO	Uncharacterized protein (Fragment) OS=Cavia porcellus GN=PRKAR2B PE=4 SV=1	CAVPO	15
606	18.65	18.7	32.27	31.63	27.8	tr|H0UTM4|H0UTM4_CAVPO	Uncharacterized protein OS=Cavia porcellus GN=RALY PE=4 SV=1	CAVPO	10
607	18.64	18.7	53.99	43.56	38.96	tr|H0VM56|H0VM56_CAVPO	Uncharacterized protein OS=Cavia porcellus GN=RPRD1B PE=4 SV=1	CAVPO	12
608	18.62	18.7	21.79	16.09	14.08	tr|H0VD03|H0VD03_CAVPO	Uncharacterized protein (Fragment) OS=Cavia porcellus GN=DNAJC6 PE=4 SV=1	CAVPO	11
609	18.58	18.8	32.81	29.66	25.17	tr|H0V7E3|H0V7E3_CAVPO	Eukaryotic translation initiation factor 3 subunit E OS=Cavia porcellus GN=EIF3E PE=3 SV=1	CAVPO	12
610	18.57	35.6	64.58	61.77	60.04	tr|H0W617|H0W617_CAVPO	Elongation factor 1-alpha OS=Cavia porcellus GN=EEF1A2 PE=3 SV=1	CAVPO	22
611	18.56	18.6	34.77	27.9	26.13	tr|H0V559|H0V559_CAVPO	Dihydrolipoyl dehydrogenase OS=Cavia porcellus GN=DLD PE=3 SV=1	CAVPO	10
612	18.56	30.5	71.19	67.51	65.82	tr|H0VJC9|H0VJC9_CAVPO	Uncharacterized protein OS=Cavia porcellus GN=GNAT2 PE=4 SV=1	CAVPO	24
613	18.52	18.7	55.79	53.96	43.29	tr|H0VKD4|H0VKD4_CAVPO	Uncharacterized protein OS=Cavia porcellus GN=BUB3 PE=4 SV=1	CAVPO	13
614	18.51	18.6	39.42	30.69	28.84	tr|B6V685|B6V685_CAVPO	Serpin B6 OS=Cavia porcellus PE=2 SV=1	CAVPO	10
615	18.5	20.6	60.07	54.51	54.17	tr|H0UX52|H0UX52_CAVPO	Uncharacterized protein (Fragment) OS=Cavia porcellus GN=DDAH2 PE=4 SV=1	CAVPO	11
616	18.5	20.7	41.87	37.19	37.19	tr|H0VC62|H0VC62_CAVPO	Uncharacterized protein OS=Cavia porcellus GN=ERP44 PE=4 SV=1	CAVPO	11
617	18.47	18.5	44.04	39.9	33.16	tr|H0V0B4|H0V0B4_CAVPO	Branched-chain-amino-acid aminotransferase (Fragment) OS=Cavia porcellus GN=BCAT1 PE=1 SV=1	CAVPO	12

618	18.46	18.5	55.19	55.19	52.28	tr|H0VP94|H0VP94_CAVPO	Proteasome subunit alpha type OS=Cavia porcellus GN=PSMA5 PE=3 SV=1	CAVPO	14
619	18.42	18.7	61.57	50.46	46.3	tr|H0UTI8|H0UTI8_CAVPO	Peptidyl-prolyl cis-trans isomerase OS=Cavia porcellus GN=PPIB PE=3 SV=1	CAVPO	11
620	18.41	18.6	16.26	11.96	6.854	tr|H0V9V6|H0V9V6_CAVPO	Uncharacterized protein OS=Cavia porcellus GN=EIF4G1 PE=4 SV=1	CAVPO	9
621	18.4	18.5	40.79	34.66	34.66	tr|H0VJN8|H0VJN8_CAVPO	Uncharacterized protein OS=Cavia porcellus GN=Rps2 PE=3 SV=1	CAVPO	10
622	18.39	18.4	41.92	38.38	35.86	tr|H0VNY0|H0VNY0_CAVPO	Uncharacterized protein OS=Cavia porcellus GN=GNB5 PE=4 SV=1	CAVPO	11
623	18.35	18.4	34.81	27.81	25.23	tr|H0V8L1|H0V8L1_CAVPO	Uncharacterized protein (Fragment) OS=Cavia porcellus GN=Aarsd1 PE=4 SV=1	CAVPO	10
624	18.33	20.7	61.98	51.71	49.43	tr|H0W454|H0W454_CAVPO	Uncharacterized protein OS=Cavia porcellus GN=NAPB PE=4 SV=1	CAVPO	10
625	18.3	18.3	53.61	45.88	43.81	tr|H0W577|H0W577_CAVPO	Uncharacterized protein OS=Cavia porcellus GN=BSG PE=4 SV=1	CAVPO	25
626	18.27	20.1	17.4	8.342	4.274	tr|H0UTT9|H0UTT9_CAVPO	Uncharacterized protein OS=Cavia porcellus GN=RANBP2 PE=4 SV=1	CAVPO	11
627	18.27	18.3	53.58	45.28	45.28	tr|H0W701|H0W701_CAVPO	Uncharacterized protein (Fragment) OS=Cavia porcellus GN=PURA PE=4 SV=1	CAVPO	10
628	18.26	18.4	57.03	53.61	51.33	tr|H0VGC1|H0VGC1_CAVPO	Uncharacterized protein (Fragment) OS=Cavia porcellus GN=FBL PE=3 SV=1	CAVPO	11
629	18.25	18.4	32.22	24.56	20.24	tr|H0VE29|H0VE29_CAVPO	Uncharacterized protein OS=Cavia porcellus GN=HARS PE=3 SV=1	CAVPO	9
630	18.21	18.3	50.29	46.29	45.71	tr|H0UWB0|H0UWB0_CAVPO	Uncharacterized protein (Fragment) OS=Cavia porcellus GN=GYG1 PE=4 SV=1	CAVPO	17
631	18.2	18.3	52.25	41.38	31.83	tr|H0VGD3|H0VGD3_CAVPO	Uncharacterized protein OS=Cavia porcellus GN=Ndufa9 PE=4 SV=1	CAVPO	13

632	18.15	20.3	71.14	71.14	70.65	tr|H0VS12|H0VS12_CAVPO	Uncharacterized protein OS=Cavia porcellus GN=RAB1B PE=3 SV=1	CAVPO	14
633	18.1	18.2	42.64	39.02	32.04	tr|H0VH01|H0VH01_CAVPO	Uncharacterized protein (Fragment) OS=Cavia porcellus GN=SEPT5 PE=3 SV=1	CAVPO	11
634	18.08	18.1	33.59	33.59	33.59	tr|H0VQ68|H0VQ68_CAVPO	Uncharacterized protein OS=Cavia porcellus GN=ATP5F1 PE=4 SV=1	CAVPO	12
635	18.04	18.1	71.98	70.88	68.13	tr|H0VR44|H0VR44_CAVPO	Uncharacterized protein (Fragment) OS=Cavia porcellus GN=ARL3 PE=3 SV=1	CAVPO	12
636	18.03	18.2	43.54	35.11	34.55	tr|H0WBZ0|H0WBZ0_CAVPO	Uncharacterized protein (Fragment) OS=Cavia porcellus GN=SAE1 PE=4 SV=1	CAVPO	11
637	18.02	18.1	45.4	45.09	40.8	tr|H0V3Q0|H0V3Q0_CAVPO	Uncharacterized protein (Fragment) OS=Cavia porcellus GN=RCN1 PE=4 SV=1	CAVPO	11
638	18	18	37.37	37.15	37.15	tr|H0UWJ4|H0UWJ4_CAVPO	Uncharacterized protein OS=Cavia porcellus GN=U2AF2 PE=4 SV=1	CAVPO	9
639	18	20	53.51	53.51	53.51	tr|H0W678|H0W678_CAVPO	Uncharacterized protein (Fragment) OS=Cavia porcellus GN=Rab3a PE=3 SV=1	CAVPO	14
640	17.98	18.1	60.15	53.51	43.54	tr|H0WBB9|H0WBB9_CAVPO	Uncharacterized protein OS=Cavia porcellus GN=OTUB1 PE=4 SV=1	CAVPO	10
641	17.96	18.4	36.56	36.29	33.6	tr|H0VFN3|H0VFN3_CAVPO	Uncharacterized protein (Fragment) OS=Cavia porcellus GN=CADM2 PE=4 SV=1	CAVPO	10
642	17.94	18	74.45	70.93	61.67	tr|H0VJK5|H0VJK5_CAVPO	Uncharacterized protein OS=Cavia porcellus GN=PCMT1 PE=4 SV=1	CAVPO	9
643	17.93	18.9	33.79	31.03	25.19	tr|H0UT24|H0UT24_CAVPO	Uncharacterized protein OS=Cavia porcellus GN=EEF1D PE=3 SV=1	CAVPO	11
644	17.92	18	75.56	75.56	75.56	tr|A9QUC5|A9QUC5_CAVPO	E-FABP OS=Cavia porcellus GN=FABP5 PE=2 SV=1	CAVPO	10
645	17.92	18	52.66	52.66	52.66	tr|H0UY27|H0UY27_CAVPO	Uncharacterized protein OS=Cavia porcellus GN=RAB18 PE=3 SV=1	CAVPO	10

646	17.89	19.9	50.18	50.18	50.18	tr|H0VBZ7|H0VBZ7_CAVPO	Uncharacterized protein (Fragment) OS=Cavia porcellus PE=4 SV=1	CAVPO	10
647	17.89	18	29.72	25.16	22.8	tr|H0VNA7|H0VNA7_CAVPO	Uncharacterized protein OS=Cavia porcellus GN=RAD21 PE=4 SV=1	CAVPO	11
648	17.87	17.9	45.44	32.7	26.75	tr|H0W8J0|H0W8J0_CAVPO	Uncharacterized protein (Fragment) OS=Cavia porcellus GN=SELENBP1 PE=4 SV=1	CAVPO	10
649	17.86	17.9	13.23	10.08	10.08	tr|H0VC49|H0VC49_CAVPO	Uncharacterized protein OS=Cavia porcellus GN=IMPG2 PE=4 SV=1	CAVPO	13
650	17.85	18	49.89	37.31	26.05	tr|H0VEL8|H0VEL8_CAVPO	Uncharacterized protein OS=Cavia porcellus GN=NUCB1 PE=4 SV=1	CAVPO	8
651	17.8	17.9	37.7	33.23	30.35	tr|H0WB79|H0WB79_CAVPO	Uncharacterized protein OS=Cavia porcellus GN=HNRNPAB PE=4 SV=1	CAVPO	11
652	17.78	17.8	62.57	62.03	60.43	tr|H0UVU1|H0UVU1_CAVPO	Uncharacterized protein (Fragment) OS=Cavia porcellus GN=UCHL1 PE=4 SV=1	CAVPO	13
653	17.78	17.9	20.88	18.24	15.22	tr|H0VB03|H0VB03_CAVPO	Uncharacterized protein (Fragment) OS=Cavia porcellus GN=PROM1 PE=4 SV=1	CAVPO	9
654	17.76	17.8	49.14	46.55	41.81	tr|H0V816|H0V816_CAVPO	Uncharacterized protein OS=Cavia porcellus GN=FAM213A PE=4 SV=1	CAVPO	9
655	17.75	17.9	34.47	34.47	28.18	tr|H0V435|H0V435_CAVPO	Uncharacterized protein OS=Cavia porcellus GN=UBQLN1 PE=4 SV=1	CAVPO	12
656	17.72	17.8	34.61	31.5	28.64	tr|H0UVQ0|H0UVQ0_CAVPO	Uncharacterized protein OS=Cavia porcellus GN=PREB PE=4 SV=1	CAVPO	9
657	17.72	17.9	15.42	8.227	6.764	tr|H0VRW6|H0VRW6_CAVPO	DNA-directed RNA polymerase OS=Cavia porcellus GN=Polr2a PE=3 SV=1	CAVPO	9
658	17.65	17.8	38.95	35.08	32.04	tr|H0V5J3|H0V5J3_CAVPO	Uncharacterized protein (Fragment) OS=Cavia porcellus GN=PIP4K2B PE=4 SV=1	CAVPO	10
659	17.64	17.8	39.89	37.61	37.61	tr|H0VHZ4|H0VHZ4_CAVPO	Uncharacterized protein OS=Cavia porcellus GN=ATP6V0D1 PE=4 SV=1	CAVPO	10

660	17.63	17.7	56.37	56.37	52.87	tr|H0W2M4|H0W2M4_CAVPO	Uncharacterized protein OS=Cavia porcellus GN=CRYM PE=4 SV=1	CAVPO	20
661	17.63	17.8	65.36	65.36	65.36	tr|Q99NI2|Q99NI2_CAVPO	Nucleoside diphosphate kinase A OS=Cavia porcellus GN=NDPK-A PE=2 SV=1	CAVPO	12
662	17.61	17.7	21.11	18.69	17.12	tr|H0VWT8|H0VWT8_CAVPO	Uncharacterized protein (Fragment) OS=Cavia porcellus GN=RAP1GAP PE=4 SV=1	CAVPO	10
663	17.6	17.7	58.21	58.21	40.06	tr|H0W3Y8|H0W3Y8_CAVPO	ATP-dependent (S)-NAD(P)H-hydrate dehydratase OS=Cavia porcellus GN=CARKD PE=3 SV=1	CAVPO	10
664	17.58	17.8	31.14	25.88	24.12	tr|H0V279|H0V279_CAVPO	Uncharacterized protein OS=Cavia porcellus GN=PSMD12 PE=4 SV=1	CAVPO	10
665	17.58	17.7	43.64	42.16	36.23	tr|H0V2R8|H0V2R8_CAVPO	Uncharacterized protein OS=Cavia porcellus GN=Wars PE=3 SV=1	CAVPO	12
666	17.57	17.7	42.14	40.13	38.13	tr|H0W149|H0W149_CAVPO	Uncharacterized protein OS=Cavia porcellus GN=Rplp0 PE=3 SV=1	CAVPO	10
667	17.55	19.2	34.59	31.76	28.71	tr|H0VHL2|H0VHL2_CAVPO	Uncharacterized protein (Fragment) OS=Cavia porcellus GN=PAICS PE=4 SV=1	CAVPO	11
668	17.55	17.9	38.77	38.53	32.39	tr|H0VHT6|H0VHT6_CAVPO	Mannose-6-phosphate isomerase (Fragment) OS=Cavia porcellus GN=MPI PE=3 SV=1	CAVPO	11
669	17.53	17.6	50.89	50.89	47.32	tr|H0VA50|H0VA50_CAVPO	Uncharacterized protein (Fragment) OS=Cavia porcellus GN=RS1 PE=4 SV=1	CAVPO	12
670	17.48	17.7	45.53	43.58	31.56	tr|H0V190|H0V190_CAVPO	Uncharacterized protein (Fragment) OS=Cavia porcellus GN=PPME1 PE=4 SV=1	CAVPO	9
671	17.48	17.6	21.26	19.2	19.2	tr|H0VUV9|H0VUV9_CAVPO	Uncharacterized protein OS=Cavia porcellus GN=NCDN PE=4 SV=1	CAVPO	12
672	17.44	17.6	22.88	18.59	17.34	tr|H0V1I4|H0V1I4_CAVPO	Uncharacterized protein OS=Cavia porcellus GN=BBS2 PE=4 SV=1	CAVPO	10
673	17.41	17.5	46.46	44.31	41.54	tr|H0V853|H0V853_CAVPO	Uncharacterized protein (Fragment) OS=Cavia	CAVPO	11

							porcellus GN=AKR7A3 PE=4 SV=1		
674	17.39	26.5	58.71	57.64	51.47	tr|H0UV47|H0UV47_CAVPO	Uncharacterized protein (Fragment) OS=Cavia porcellus GN=PCBP2 PE=4 SV=1	CAVPO	17
675	17.37	17.5	62.62	62.62	50.97	tr|H0UW23|H0UW23_CAVPO	Uncharacterized protein (Fragment) OS=Cavia porcellus GN=LIN7A PE=4 SV=1	CAVPO	10
676	17.37	17.5	47.15	44.11	34.98	tr|H0W953|H0W953_CAVPO	40S ribosomal protein S4 OS=Cavia porcellus GN=RPS4X PE=3 SV=1	CAVPO	9
677	17.35	17.6	76	62.4	62.4	tr|H0VUU3|H0VUU3_CAVPO	Histone H2B (Fragment) OS=Cavia porcellus GN=HIST1H2BM PE=3 SV=1	CAVPO	37
678	17.3	18.7	40.87	40.56	37.77	tr|H0V629|H0V629_CAVPO	Serine/threonine-protein phosphatase OS=Cavia porcellus GN=PPP1CC PE=3 SV=1	CAVPO	10
679	17.28	17.4	43.17	43.17	40.06	tr|H0VR31|H0VR31_CAVPO	Probable tRNA N6-adenosine threonylcarbamoyltransferase OS=Cavia porcellus GN=OSGEP PE=3 SV=1	CAVPO	9
680	17.27	21.6	44.63	34.11	32	tr|H0V9M9|H0V9M9_CAVPO	Uncharacterized protein (Fragment) OS=Cavia porcellus GN=SEPT8 PE=3 SV=1	CAVPO	12
681	17.27	17.8	18.77	12.89	8.711	tr|H0VR73|H0VR73_CAVPO	Uncharacterized protein (Fragment) OS=Cavia porcellus GN=EEA1 PE=4 SV=1	CAVPO	11
682	17.26	17.4	43.09	36.97	36.7	tr|H0V556|H0V556_CAVPO	Uncharacterized protein OS=Cavia porcellus GN=DEK PE=4 SV=1	CAVPO	11
683	17.23	19.5	57.78	57.78	57.78	tr|H0V2K4|H0V2K4_CAVPO	Uncharacterized protein OS=Cavia porcellus GN=PRDX4 PE=4 SV=1	CAVPO	13
684	17.21	17.4	48.5	40.53	40.53	tr|H0VYZ5|H0VYZ5_CAVPO	Uncharacterized protein OS=Cavia porcellus GN=CYB5R3 PE=3 SV=1	CAVPO	11
685	17.2	17.3	34.22	31.31	25	tr|H0W6Z1|H0W6Z1_CAVPO	Uncharacterized protein OS=Cavia porcellus GN=KIAA0513 PE=4 SV=1	CAVPO	10
686	17.19	17.3	16.01	13.11	12.99	tr|H0UXZ0|H0UXZ0_CAVPO	Uncharacterized protein OS=Cavia porcellus GN=PIP5K1A PE=4 SV=1	CAVPO	12

687	17.19	17.4	24.79	16.48	15.37	tr|H0VQ21|H0VQ21_CAVPO	Uncharacterized protein (Fragment) OS=Cavia porcellus GN=TARS PE=3 SV=1	CAVPO	11
688	17.13	17.4	37.67	35	33.33	tr|H0UXT3|H0UXT3_CAVPO	Uncharacterized protein OS=Cavia porcellus GN=ARPC2 PE=4 SV=1	CAVPO	11
689	17.13	17.3	50	42.01	42.01	tr|H0V811|H0V811_CAVPO	Uncharacterized protein OS=Cavia porcellus GN=AHSA1 PE=4 SV=1	CAVPO	11
690	17.12	17.2	66.89	58.28	58.28	tr|H0VS22|H0VS22_CAVPO	Uncharacterized protein (Fragment) OS=Cavia porcellus GN=MYL6 PE=4 SV=1	CAVPO	9
691	17.11	17.6	17.53	10.82	8.247	tr|H0VAH4|H0VAH4_CAVPO	Uncharacterized protein OS=Cavia porcellus GN=TNR PE=4 SV=1	CAVPO	9
692	17.1	17.5	11.14	7.625	3.092	tr|H0VEU6|H0VEU6_CAVPO	Uncharacterized protein (Fragment) OS=Cavia porcellus GN=DMXL2 PE=4 SV=1	CAVPO	7
693	17.1	17.5	21.62	13.41	8.333	tr|H0VQ01|H0VQ01_CAVPO	Uncharacterized protein (Fragment) OS=Cavia porcellus GN=IQGAP1 PE=4 SV=1	CAVPO	9
694	17.08	17.1	60.69	60.69	60.69	tr|H0VQN1|H0VQN1_CAVPO	Uncharacterized protein (Fragment) OS=Cavia porcellus GN=RAB2A PE=3 SV=1	CAVPO	13
695	17.05	17.1	56.34	56.34	56.34	tr|H0UUI9|H0UUI9_CAVPO	Uncharacterized protein OS=Cavia porcellus GN=Atp5o PE=3 SV=1	CAVPO	13
696	17.04	27	54.2	37.83	33.41	tr|H0UUU6|H0UUU6_CAVPO	Uncharacterized protein (Fragment) OS=Cavia porcellus GN=DNM3 PE=3 SV=1	CAVPO	14
697	17.03	17.1	59.92	50.61	42.91	tr|H0VQE7|H0VQE7_CAVPO	Uncharacterized protein OS=Cavia porcellus GN=PSMA7 PE=4 SV=1	CAVPO	9
698	17.03	17.3	34.49	30.59	22.78	tr|H0W752|H0W752_CAVPO	Uncharacterized protein (Fragment) OS=Cavia porcellus GN=LOC100720949 PE=3 SV=1	CAVPO	9
699	17.02	17.2	58.76	55.47	45.99	tr|H0UT25|H0UT25_CAVPO	Uncharacterized protein OS=Cavia porcellus GN=PYCRL PE=4 SV=1	CAVPO	10
700	16.96	18.5	49.62	41.31	41.31	tr|H0UU01|H0UU01_CAVPO	Uncharacterized protein OS=Cavia porcellus GN=DNAJA1 PE=3 SV=1	CAVPO	13

701	16.93	17.1	35.56	26.46	26.46	tr|H0UV40|H0UV40_CAVPO	Ubiquitin carboxyl-terminal hydrolase (Fragment) OS=Cavia porcellus GN=USP14 PE=3 SV=1	CAVPO	11
702	16.91	17	73.77	65.57	58.47	tr|H0V7C2|H0V7C2_CAVPO	Uncharacterized protein OS=Cavia porcellus GN=ARF5 PE=3 SV=1	CAVPO	11
703	16.88	17.1	49.39	41.77	38.11	tr|H0VGU4|H0VGU4_CAVPO	Uncharacterized protein OS=Cavia porcellus GN=NUDC PE=4 SV=1	CAVPO	13
704	16.85	17	41.01	33.6	28.31	tr|H0V834|H0V834_CAVPO	Uncharacterized protein (Fragment) OS=Cavia porcellus GN=ACAD8 PE=3 SV=1	CAVPO	9
705	16.83	17	24.04	16.98	10.36	tr|H0VDQ2|H0VDQ2_CAVPO	Uncharacterized protein (Fragment) OS=Cavia porcellus GN=EIF4G2 PE=4 SV=1	CAVPO	8
706	16.83	17.3	16.85	13.75	10.55	tr|H0VMS1|H0VMS1_CAVPO	Uncharacterized protein OS=Cavia porcellus GN=CP PE=4 SV=1	CAVPO	8
707	16.82	16.9	50.57	44.53	39.25	tr|H0VQ50|H0VQ50_CAVPO	Proteasome subunit beta type (Fragment) OS=Cavia porcellus GN=PSMB5 PE=3 SV=1	CAVPO	9
708	16.79	16.9	62.5	62.5	62.5	tr|H0W7U7|H0W7U7_CAVPO	Uncharacterized protein OS=Cavia porcellus GN=HSPB1 PE=3 SV=1	CAVPO	12
709	16.78	17.2	21.47	17.85	10.95	tr|H0VAC1|H0VAC1_CAVPO	Eukaryotic translation initiation factor 3 subunit C OS=Cavia porcellus GN=Eif3c PE=3 SV=1	CAVPO	8
710	16.74	16.8	45.88	45.1	45.1	tr|H0V721|H0V721_CAVPO	Proteasome subunit alpha type OS=Cavia porcellus GN=PSMA3 PE=3 SV=1	CAVPO	11
711	16.72	19.1	31.7	21.24	16.56	tr|H0VBJ0|H0VBJ0_CAVPO	Uncharacterized protein OS=Cavia porcellus GN=BCLAF1 PE=4 SV=1	CAVPO	12
712	16.71	16.9	37.5	32.89	27.19	tr|H0UU39|H0UU39_CAVPO	Uncharacterized protein OS=Cavia porcellus GN=FKBP5 PE=4 SV=1	CAVPO	9
713	16.7	16.8	40.07	26.78	25.28	tr|H0W6B9|H0W6B9_CAVPO	Uncharacterized protein OS=Cavia porcellus GN=TULP1 PE=4 SV=1	CAVPO	11

714	16.67	18	36.69	30.8	23	tr|H0VCS0|H0VCS0_CAVPO	Uncharacterized protein OS=Cavia porcellus GN=SLC32A1 PE=4 SV=1	CAVPO	10
715	16.65	17.8	18.15	13.87	11.78	tr|H0VLZ0|H0VLZ0_CAVPO	Signal transducer and activator of transcription OS=Cavia porcellus GN=STAT1 PE=3 SV=1	CAVPO	12
716	16.64	23.7	48.56	46.26	42.24	tr|H0W3C3|H0W3C3_CAVPO	Uncharacterized protein (Fragment) OS=Cavia porcellus GN=CTBP1 PE=3 SV=1	CAVPO	14
717	16.63	16.8	14.64	12.16	7.522	tr|H0VBV1|H0VBV1_CAVPO	Uncharacterized protein OS=Cavia porcellus GN=WDR7 PE=4 SV=1	CAVPO	8
718	16.62	16.7	43.79	38.64	33.96	tr|H0VSG3|H0VSG3_CAVPO	Uncharacterized protein OS=Cavia porcellus GN=ACAT1 PE=3 SV=1	CAVPO	9
719	16.6	16.8	38.73	35.95	33.67	tr|H0UWF4|H0UWF4_CAVPO	S-adenosylmethionine synthase OS=Cavia porcellus GN=MAT2A PE=3 SV=1	CAVPO	9
720	16.6	16.7	49.12	40.71	40.71	tr|H0VWF1|H0VWF1_CAVPO	Uncharacterized protein OS=Cavia porcellus GN=ATP6V1E1 PE=3 SV=1	CAVPO	12
721	16.58	16.8	24.59	18.33	18.33	tr|H0UTF8|H0UTF8_CAVPO	Signal recognition particle subunit SRP72 OS=Cavia porcellus GN=SRP72 PE=3 SV=1	CAVPO	10
722	16.58	17.3	27.43	16.51	9.049	tr|H0UZY0|H0UZY0_CAVPO	Uncharacterized protein (Fragment) OS=Cavia porcellus GN=TCERG1 PE=4 SV=1	CAVPO	9
723	16.56	16.9	29.69	28.21	21.12	tr|H0V790|H0V790_CAVPO	NADPH--cytochrome P450 reductase OS=Cavia porcellus GN=Por PE=3 SV=1	CAVPO	10
724	16.54	16.7	25.23	20.63	17.5	tr|H0VNU3|H0VNU3_CAVPO	Uncharacterized protein OS=Cavia porcellus PE=3 SV=1	CAVPO	9
725	16.47	16.7	30.79	23.34	18.96	tr|H0VS40|H0VS40_CAVPO	Uncharacterized protein (Fragment) OS=Cavia porcellus GN=PRKCA PE=4 SV=1	CAVPO	8
726	16.45	22.5	48.34	47.87	45.02	tr|H0V150|H0V150_CAVPO	Uncharacterized protein OS=Cavia porcellus GN=HMGB2 PE=4 SV=1	CAVPO	21
727	16.41	20.8	30.98	26.53	22.45	tr|H0W9H4|H0W9H4_CAVPO	Uncharacterized protein (Fragment) OS=Cavia	CAVPO	11

							porcellus GN=BIN1 PE=4 SV=1		
728	16.39	16.5	45.74	41.13	37.59	tr|H0V183|H0V183_CAVPO	Uncharacterized protein OS=Cavia porcellus GN=PSMD13 PE=4 SV=1	CAVPO	9
729	16.39	16.4	50.88	41.81	34.5	tr|H0VL96|H0VL96_CAVPO	Uncharacterized protein (Fragment) OS=Cavia porcellus GN=MSI1 PE=4 SV=1	CAVPO	9
730	16.37	83.4	83.15	80.45	80.22	tr|H0VMG5|H0VMG5_CAVPO	Uncharacterized protein OS=Cavia porcellus GN=TUBB2B PE=3 SV=1	CAVPO	132
731	16.34	22.6	76.61	69.27	56.88	tr|H0VQH5|H0VQH5_CAVPO	Uncharacterized protein OS=Cavia porcellus GN=GSTM2 PE=3 SV=1	CAVPO	16
732	16.33	16.4	57.89	57.89	53.29	tr|H0VCN8|H0VCN8_CAVPO	Uncharacterized protein (Fragment) OS=Cavia porcellus GN=LOC100727315 PE=3 SV=1	CAVPO	10
733	16.31	16.6	28.19	28.19	19.24	tr|H0V329|H0V329_CAVPO	Uncharacterized protein (Fragment) OS=Cavia porcellus GN=RANGAP1 PE=4 SV=1	CAVPO	10
734	16.27	16.4	48.84	46.84	43.85	tr|H0VPV4|H0VPV4_CAVPO	Uncharacterized protein OS=Cavia porcellus GN=CAPZB PE=4 SV=1	CAVPO	15
735	16.22	16.4	45.77	44.28	38.81	tr|H0UWR3|H0UWR3_CAVPO	Uncharacterized protein (Fragment) OS=Cavia porcellus GN=ACAT2 PE=3 SV=1	CAVPO	10
736	16.21	16.2	48.6	48.6	45.79	tr|H0VTZ4|H0VTZ4_CAVPO	Uncharacterized protein (Fragment) OS=Cavia porcellus GN=SEC13 PE=4 SV=1	CAVPO	10
737	16.2	16.4	29.37	18.03	14.34	tr|H0VJ48|H0VJ48_CAVPO	Uncharacterized protein OS=Cavia porcellus GN=APEH PE=4 SV=1	CAVPO	10
738	16.19	16.2	37.92	34.83	31.74	tr|H0VKJ1|H0VKJ1_CAVPO	Uncharacterized protein OS=Cavia porcellus GN=STOML2 PE=4 SV=1	CAVPO	9
739	16.19	16.2	20.85	17.49	16.62	tr|H0WC86|H0WC86_CAVPO	Uncharacterized protein OS=Cavia porcellus GN=CNGA1 PE=4 SV=1	CAVPO	9
740	16.17	16.5	60.22	59.68	56.99	tr|H0W6D7|H0W6D7_CAVPO	Uncharacterized protein OS=Cavia porcellus GN=ARL8B PE=3 SV=1	CAVPO	10

741	16.15	16.3	39.11	29.38	20.82	tr|H0VTZ8|H0VTZ8_CAVPO	Uncharacterized protein OS=Cavia porcellus GN=SARS PE=3 SV=1	CAVPO	8
742	16.14	16.3	51.96	40.22	31.56	tr|H0UUR5|H0UUR5_CAVPO	Uncharacterized protein OS=Cavia porcellus GN=SLC9A3R1 PE=4 SV=1	CAVPO	8
743	16.13	16.2	41.41	34.8	33.48	tr|H0VEC4|H0VEC4_CAVPO	Uncharacterized protein OS=Cavia porcellus GN=DLST PE=3 SV=1	CAVPO	10
744	16.13	16.2	75.32	67.53	63.64	tr|H0VFM6|H0VFM6_CAVPO	Uncharacterized protein OS=Cavia porcellus GN=EIF5AL1 PE=4 SV=1	CAVPO	9
745	16.11	16.1	49.52	49.05	49.05	tr|H0VRR4|H0VRR4_CAVPO	Uncharacterized protein (Fragment) OS=Cavia porcellus GN=CMPK1 PE=3 SV=1	CAVPO	9
746	16.08	16.1	64.4	53.4	53.4	tr|H0WBZ2|H0WBZ2_CAVPO	Uncharacterized protein OS=Cavia porcellus GN=Cdc42 PE=3 SV=1	CAVPO	10
747	15.98	16.2	26.67	15.77	11.15	tr|H0VJ64|H0VJ64_CAVPO	Uncharacterized protein OS=Cavia porcellus GN=CUL5 PE=3 SV=1	CAVPO	9
748	15.92	16.1	15.49	11.38	6.886	tr|H0V0I8|H0V0I8_CAVPO	Uncharacterized protein OS=Cavia porcellus GN=PFAS PE=4 SV=1	CAVPO	7
749	15.91	16	55.36	47.06	40.48	tr|H0VF98|H0VF98_CAVPO	Uncharacterized protein OS=Cavia porcellus GN=TXNL1 PE=4 SV=1	CAVPO	8
750	15.91	16	24.7	24.7	24.7	tr|H0VJL3|H0VJL3_CAVPO	Uncharacterized protein OS=Cavia porcellus GN=TARDBP PE=4 SV=1	CAVPO	8
751	15.9	16	54.83	48.97	45.86	tr|H0VCV4|H0VCV4_CAVPO	Uncharacterized protein (Fragment) OS=Cavia porcellus GN=NTM PE=4 SV=1	CAVPO	9
752	15.87	16	37.33	26.43	26.43	tr|H0V0Q6|H0V0Q6_CAVPO	Uncharacterized protein OS=Cavia porcellus GN=Sugt1 PE=4 SV=1	CAVPO	8
753	15.87	16	43.15	37.5	37.5	tr|H0VPC7|H0VPC7_CAVPO	Uncharacterized protein OS=Cavia porcellus GN=ALYREF PE=4 SV=1	CAVPO	8
754	15.85	16	51.41	51.41	41.37	tr|H0VCG2|H0VCG2_CAVPO	Uncharacterized protein (Fragment) OS=Cavia porcellus GN=NDUFV2 PE=4 SV=1	CAVPO	10

755	15.84	15.9	47.35	44.88	44.88	tr|H0VNA2|H0VNA2_CAVPO	Uncharacterized protein (Fragment) OS=Cavia porcellus GN=CYC1 PE=4 SV=1	CAVPO	10
756	15.82	16	29.4	26.08	18.94	tr|H0VI33|H0VI33_CAVPO	Uncharacterized protein OS=Cavia porcellus GN=PPP2R5D PE=4 SV=1	CAVPO	9
757	15.81	15.9	20.58	16.25	14.8	tr|H0UWZ5|H0UWZ5_CAVPO	Uncharacterized protein (Fragment) OS=Cavia porcellus GN=Psap PE=4 SV=1	CAVPO	10
758	15.77	16	47.16	42.79	28.38	tr|H0V3S4|H0V3S4_CAVPO	Uncharacterized protein OS=Cavia porcellus GN=AAK1 PE=4 SV=1	CAVPO	8
759	15.73	15.8	70.56	66.67	66.67	tr|H0VG07|H0VG07_CAVPO	Uncharacterized protein (Fragment) OS=Cavia porcellus GN=GLO1 PE=4 SV=1	CAVPO	16
760	15.66	15.9	18.5	16.84	10.38	tr|H0VAD0|H0VAD0_CAVPO	Uncharacterized protein (Fragment) OS=Cavia porcellus GN=GARS PE=3 SV=1	CAVPO	7
761	15.64	15.8	32.29	24.34	21.93	tr|H0V9P2|H0V9P2_CAVPO	Uncharacterized protein OS=Cavia porcellus GN=SRSF4 PE=4 SV=1	CAVPO	10
762	15.62	15.8	50.38	50.13	38.93	tr|H0V8U6|H0V8U6_CAVPO	Uncharacterized protein OS=Cavia porcellus GN=LOC100727448 PE=3 SV=1	CAVPO	8
763	15.57	15.6	37.24	37.24	31.51	tr|H0UUI2|H0UUI2_CAVPO	Uncharacterized protein OS=Cavia porcellus GN=Map4 PE=4 SV=1	CAVPO	9
764	15.53	18	73.87	61.81	57.79	tr|H0W316|H0W316_CAVPO	Uncharacterized protein (Fragment) OS=Cavia porcellus GN=PRDX2 PE=4 SV=1	CAVPO	17
765	15.5	15.6	53.73	53.73	41.42	tr|H0W6T7|H0W6T7_CAVPO	Uncharacterized protein OS=Cavia porcellus GN=Mapre1 PE=4 SV=1	CAVPO	8
766	15.49	15.5	40.48	38.44	38.44	tr|H0VIS2|H0VIS2_CAVPO	Uncharacterized protein OS=Cavia porcellus GN=SDR39U1 PE=4 SV=1	CAVPO	10
767	15.48	15.7	27.11	17.09	11.84	tr|H0VCD3|H0VCD3_CAVPO	Uncharacterized protein OS=Cavia porcellus GN=NUP93 PE=4 SV=1	CAVPO	8
768	15.44	15.7	20.72	13.92	10.41	tr|H0VAB1|H0VAB1_CAVPO	Uncharacterized protein OS=Cavia porcellus GN=PRPF6 PE=4 SV=1	CAVPO	9

769	15.44	15.5	38.3	27.75	24.77	tr|H0VCC9|H0VCC9_CAVPO	Uncharacterized protein OS=Cavia porcellus GN=PDK1 PE=4 SV=1	CAVPO	9
770	15.42	15.4	35.29	34.9	34.9	tr|H0V664|H0V664_CAVPO	Uncharacterized protein OS=Cavia porcellus GN=SNRPA1 PE=4 SV=1	CAVPO	8
771	15.42	15.5	17.67	14.59	14.59	tr|H0VBF0|H0VBF0_CAVPO	Uncharacterized protein OS=Cavia porcellus GN=SV2B PE=4 SV=1	CAVPO	11
772	15.42	15.6	51.82	51.21	34.85	tr|H0VYI8|H0VYI8_CAVPO	Uncharacterized protein OS=Cavia porcellus GN=AIP PE=4 SV=1	CAVPO	10
773	15.41	15.4	49.04	45.19	45.19	tr|H0W399|H0W399_CAVPO	Uncharacterized protein OS=Cavia porcellus GN=THEM6 PE=4 SV=1	CAVPO	9
774	15.38	15.5	21.36	14.29	12.82	tr|H0VLR0|H0VLR0_CAVPO	Uncharacterized protein (Fragment) OS=Cavia porcellus GN=NCBP1 PE=4 SV=1	CAVPO	9
775	15.37	15.4	39.49	33.12	30.89	tr|H0UUD1|H0UUD1_CAVPO	Uncharacterized protein OS=Cavia porcellus GN=SGTA PE=4 SV=1	CAVPO	8
776	15.35	25.5	40.75	39.25	29.17	tr|H0W1F5|H0W1F5_CAVPO	Uncharacterized protein OS=Cavia porcellus GN=CAMK2B PE=4 SV=1	CAVPO	16
777	15.32	15.8	46.65	40.5	36.31	tr|H0V391|H0V391_CAVPO	Uncharacterized protein OS=Cavia porcellus GN=SNRNP40 PE=4 SV=1	CAVPO	10
778	15.32	15.4	41.73	29.86	29.86	tr|H0VH31|H0VH31_CAVPO	ATP synthase subunit gamma (Fragment) OS=Cavia porcellus GN=ATP5C1 PE=3 SV=1	CAVPO	8
779	15.3	15.5	49.13	44.19	41.57	tr|H0V804|H0V804_CAVPO	Aldose 1-epimerase OS=Cavia porcellus GN=GALM PE=3 SV=1	CAVPO	13
780	15.3	15.4	36.46	36.46	36.46	tr|H0VHX0|H0VHX0_CAVPO	Uncharacterized protein (Fragment) OS=Cavia porcellus GN=SNRPA PE=4 SV=1	CAVPO	10
781	15.29	15.4	29.63	29.63	27.51	tr|H0VAE5|H0VAE5_CAVPO	Uncharacterized protein OS=Cavia porcellus GN=CDC37 PE=4 SV=1	CAVPO	9
782	15.28	15.3	47.83	42.61	42.17	tr|H0VLZ7|H0VLZ7_CAVPO	Uncharacterized protein (Fragment) OS=Cavia porcellus GN=CACYBP PE=4 SV=1	CAVPO	8

783	15.26	15.4	38.12	34.9	23.02	tr|H0VDK5|H0VDK5_CAVPO	Uncharacterized protein (Fragment) OS=Cavia porcellus GN=Nr2e3 PE=3 SV=1	CAVPO	8
784	15.24	27.8	39.05	29.6	22.94	tr|H0V9B7|H0V9B7_CAVPO	Uncharacterized protein (Fragment) OS=Cavia porcellus GN=MSN PE=4 SV=1	CAVPO	14
785	15.21	15.3	53.94	52.7	52.7	tr|H0W7R3|H0W7R3_CAVPO	Uncharacterized protein OS=Cavia porcellus GN=CLIC1 PE=4 SV=1	CAVPO	9
786	15.2	15.3	21.68	21.33	18.88	tr|H0V4I6|H0V4I6_CAVPO	Uncharacterized protein OS=Cavia porcellus GN=STX3 PE=3 SV=1	CAVPO	9
787	15.18	26.8	54.11	47.21	47.21	tr|H0V0F7|H0V0F7_CAVPO	Uncharacterized protein (Fragment) OS=Cavia porcellus GN=MAPK3 PE=4 SV=1	CAVPO	14
788	15.15	15.3	33.33	23.12	12.16	tr|H0V7U5|H0V7U5_CAVPO	Uncharacterized protein OS=Cavia porcellus GN=XPNPEP1 PE=3 SV=1	CAVPO	7
789	15.12	15.5	24.31	17.93	13.84	tr|H0VNC7|H0VNC7_CAVPO	Uncharacterized protein OS=Cavia porcellus GN=GRIPAP1 PE=4 SV=1	CAVPO	10
790	15.07	15.1	29.41	25.41	23.53	tr|H0W0W7|H0W0W7_CAVPO	Uncharacterized protein OS=Cavia porcellus GN=FAM98B PE=4 SV=1	CAVPO	8
791	15.07	15.2	33.42	32.64	32.64	tr|H0WDP3|H0WDP3_CAVPO	Uncharacterized protein (Fragment) OS=Cavia porcellus GN=ZNF385A PE=4 SV=1	CAVPO	8
792	15.06	22.8	63.82	60.16	56.1	tr|H0VHQ9|H0VHQ9_CAVPO	Uncharacterized protein OS=Cavia porcellus GN=YWHAH PE=3 SV=1	CAVPO	17
793	15.06	15.2	45.81	36.53	32.63	tr|H0VK04|H0VK04_CAVPO	Uncharacterized protein OS=Cavia porcellus GN=COPS5 PE=4 SV=1	CAVPO	9
794	14.97	16.2	26.2	20.54	17.14	tr|H0VIY6|H0VIY6_CAVPO	Uncharacterized protein OS=Cavia porcellus GN=LETM1 PE=4 SV=1	CAVPO	8
795	14.96	15	47.86	47.86	44.75	tr|H0VA51|H0VA51_CAVPO	Uncharacterized protein OS=Cavia porcellus GN=PRDX3 PE=4 SV=1	CAVPO	12
796	14.94	15.1	52.41	46.08	36.45	tr|H0W608|H0W608_CAVPO	Uncharacterized protein OS=Cavia porcellus GN=CHORDC1 PE=4 SV=1	CAVPO	9

797	14.92	15	40.6	38.59	33.89	tr|H0UUD5|H0UUD5_CAVPO	Uncharacterized protein OS=Cavia porcellus GN=ISOC1 PE=4 SV=1	CAVPO	7
798	14.86	15	17.79	17.66	13.43	tr|H0V956|H0V956_CAVPO	Uncharacterized protein (Fragment) OS=Cavia porcellus GN=IMPG1 PE=4 SV=1	CAVPO	9
799	14.81	15.2	9.263	5.811	3.368	tr|H0VMT0|H0VMT0_CAVPO	Uncharacterized protein OS=Cavia porcellus GN=CNOT1 PE=4 SV=1	CAVPO	7
800	14.79	14.9	35.12	23.7	17.63	tr|H0VU80|H0VU80_CAVPO	Uncharacterized protein OS=Cavia porcellus GN=RPH3A PE=4 SV=1	CAVPO	8
801	14.78	15.1	26.73	15.22	11.39	tr|H0VGT0|H0VGT0_CAVPO	Uncharacterized protein (Fragment) OS=Cavia porcellus GN=EPS15L1 PE=4 SV=1	CAVPO	8
802	14.78	14.8	23.4	17.88	15.56	tr|H0VLB8|H0VLB8_CAVPO	Uncharacterized protein OS=Cavia porcellus GN=CDH2 PE=3 SV=1	CAVPO	12
803	14.77	14.9	40.33	31.65	19.71	tr|H0VJJ1|H0VJJ1_CAVPO	Uncharacterized protein OS=Cavia porcellus GN=GLYR1 PE=4 SV=1	CAVPO	8
804	14.66	14.8	45.79	41.43	29.6	tr|H0UT26|H0UT26_CAVPO	Uncharacterized protein OS=Cavia porcellus GN=TSTA3 PE=3 SV=1	CAVPO	8
805	14.63	14.7	25.96	25.96	25.96	tr|H0WE19|H0WE19_CAVPO	Uncharacterized protein OS=Cavia porcellus GN=KHDRBS1 PE=4 SV=1	CAVPO	9
806	14.62	14.7	35.92	33.62	33.62	tr|H0VCT5|H0VCT5_CAVPO	Uncharacterized protein OS=Cavia porcellus GN=GALE PE=3 SV=1	CAVPO	9
807	14.6	14.8	14.92	8.305	5.085	tr|H0VI14|H0VI14_CAVPO	Uncharacterized protein (Fragment) OS=Cavia porcellus GN=TJP1 PE=4 SV=1	CAVPO	8
808	14.57	14.7	24.58	22.36	14.72	tr|H0V2Q8|H0V2Q8_CAVPO	Uncharacterized protein OS=Cavia porcellus GN=ACSL3 PE=4 SV=1	CAVPO	7
809	14.57	14.6	42.01	42.01	32.29	tr|H0V8G5|H0V8G5_CAVPO	Uncharacterized protein (Fragment) OS=Cavia porcellus GN=AIMP1 PE=4 SV=1	CAVPO	8
810	14.56	14.6	26.89	24.44	24.44	tr|H0VVP2|H0VVP2_CAVPO	Clusterin (Fragment) OS=Cavia porcellus GN=CLU PE=3 SV=1	CAVPO	8

811	14.55	14.7	12.31	9.9	8.153	tr|H0V362|H0V362_CAVPO	Uncharacterized protein OS=Cavia porcellus GN=SLC4A7 PE=4 SV=1	CAVPO	8
812	14.54	30.7	45.98	45.98	43.22	tr|H0V478|H0V478_CAVPO	Uncharacterized protein (Fragment) OS=Cavia porcellus GN=EIF4A1 PE=3 SV=1	CAVPO	17
813	14.52	14.6	25.54	22.66	22.66	tr|H0UY85|H0UY85_CAVPO	Uncharacterized protein OS=Cavia porcellus GN=GPM6A PE=4 SV=1	CAVPO	10
814	14.51	14.7	31.72	28.57	20.8	tr|H0UXI4|H0UXI4_CAVPO	Uncharacterized protein (Fragment) OS=Cavia porcellus GN=RABEP2 PE=4 SV=1	CAVPO	8
815	14.51	14.6	29.2	27.43	25.37	tr|H0V7B3|H0V7B3_CAVPO	Uncharacterized protein OS=Cavia porcellus GN=ERLIN2 PE=4 SV=1	CAVPO	7
816	14.5	14.6	35.83	32.25	31.27	tr|H0UZG2|H0UZG2_CAVPO	Uncharacterized protein (Fragment) OS=Cavia porcellus PE=4 SV=1	CAVPO	10
817	14.49	14.6	49.83	47.39	31.71	tr|H0UZ78|H0UZ78_CAVPO	Uncharacterized protein OS=Cavia porcellus GN=GNPDA1 PE=3 SV=1	CAVPO	9
818	14.49	16.6	33.26	30.37	24.17	tr|H0VH83|H0VH83_CAVPO	Uncharacterized protein OS=Cavia porcellus GN=Ddx6 PE=3 SV=1	CAVPO	8
819	14.48	15.2	30.1	25.11	13.27	tr|H0VWN4|H0VWN4_CAVPO	Uncharacterized protein (Fragment) OS=Cavia porcellus GN=SART1 PE=4 SV=1	CAVPO	7
820	14.46	14.7	18.94	15.51	8.826	tr|H0VHF0|H0VHF0_CAVPO	Uncharacterized protein OS=Cavia porcellus GN=NRD1 PE=3 SV=1	CAVPO	9
821	14.45	14.6	36.85	21.57	19.78	tr|H0VUU6|H0VUU6_CAVPO	Serine/threonine-protein phosphatase 2A 55 kDa regulatory subunit B (Fragment) OS=Cavia porcellus GN=PPP2R2A PE=3 SV=1	CAVPO	8
822	14.44	14.5	30.06	30.06	26.59	tr|H0VGD6|H0VGD6_CAVPO	Uncharacterized protein OS=Cavia porcellus GN=Opn1sw PE=3 SV=1	CAVPO	10
823	14.43	14.5	50.39	50.39	50.39	tr|H0V5T6|H0V5T6_CAVPO	Uncharacterized protein OS=Cavia porcellus GN=SUB1 PE=4 SV=1	CAVPO	9
824	14.43	14.5	35.42	32.44	27.98	tr|H0V826|H0V826_CAVPO	Uncharacterized protein OS=Cavia porcellus	CAVPO	8

							GN=VPS26B PE=4 SV=1		
825	14.42	14.5	27.87	21.35	20	tr|H0UZD0|H0UZD0_CAVPO	Dolichyl-diphosphooligosaccharide--protein glycosyltransferase 48 kDa subunit (Fragment) OS=Cavia porcellus GN=DDOST PE=3 SV=1	CAVPO	8
826	14.4	14.6	16.16	11.47	7.01	tr|H0UW78|H0UW78_CAVPO	Uncharacterized protein OS=Cavia porcellus GN=SUPT6H PE=4 SV=1	CAVPO	8
827	14.4	14.5	83.9	83.9	77.97	tr|H0W7H4|H0W7H4_CAVPO	Uncharacterized protein (Fragment) OS=Cavia porcellus GN=TCEB2 PE=4 SV=1	CAVPO	8
828	14.38	15.8	36.81	36.81	34.89	tr|H0VP33|H0VP33_CAVPO	Uncharacterized protein (Fragment) OS=Cavia porcellus GN=PTPLAD1 PE=4 SV=1	CAVPO	13
829	14.37	14.4	39.38	37.5	30.94	tr|H0UUU1|H0UUU1_CAVPO	Uncharacterized protein (Fragment) OS=Cavia porcellus GN=RDH12 PE=3 SV=1	CAVPO	7
830	14.34	14.5	37.55	31.74	27.8	tr|H0VKK5|H0VKK5_CAVPO	Uncharacterized protein OS=Cavia porcellus GN=G3BP2 PE=4 SV=1	CAVPO	11
831	14.34	14.4	34.12	28.95	17.13	tr|H0W7X2|H0W7X2_CAVPO	Uncharacterized protein (Fragment) OS=Cavia porcellus GN=DBN1 PE=4 SV=1	CAVPO	7
832	14.3	14.4	47.46	31.07	31.07	tr|H0W4Y1|H0W4Y1_CAVPO	Uncharacterized protein OS=Cavia porcellus GN=Thumpd1 PE=4 SV=1	CAVPO	8
833	14.28	14.6	41.5	31.07	24.03	tr|H0UTF0|H0UTF0_CAVPO	Uncharacterized protein (Fragment) OS=Cavia porcellus GN=ARRB1 PE=4 SV=1	CAVPO	8
834	14.28	14.7	14.81	14.68	12.86	tr|H0VQB7|H0VQB7_CAVPO	Uncharacterized protein (Fragment) OS=Cavia porcellus GN=Hcn1 PE=4 SV=1	CAVPO	8
835	14.28	14.4	48.09	47.66	45.11	tr|H0W0X2|H0W0X2_CAVPO	Uncharacterized protein OS=Cavia porcellus GN=RGS9BP PE=4 SV=1	CAVPO	8
836	14.27	14.3	14.95	14.8	13.9	tr|H0W436|H0W436_CAVPO	Uncharacterized protein OS=Cavia porcellus GN=TMEM201 PE=4 SV=1	CAVPO	10
837	14.24	14.4	21.43	15.28	12.57	tr|H0W379|H0W379_CAVPO	Uncharacterized protein OS=Cavia porcellus GN=Ubap2l PE=4 SV=1	CAVPO	7

838	14.22	14.4	23.41	17.33	13.49	tr|H0VDQ5|H0VDQ5_CAVPO	Uncharacterized protein OS=Cavia porcellus GN=UBTF PE=4 SV=1	CAVPO	9
839	14.21	16.5	43.4	40.88	34.91	tr|H0V2N6|H0V2N6_CAVPO	Uncharacterized protein (Fragment) OS=Cavia porcellus GN=GNAQ PE=4 SV=1	CAVPO	9
840	14.21	14.2	53.7	53.24	46.76	tr|H0V568|H0V568_CAVPO	Uncharacterized protein OS=Cavia porcellus GN=RAB5A PE=3 SV=1	CAVPO	7
841	14.21	14.3	31.09	23.64	18	tr|H0V5R5|H0V5R5_CAVPO	Uncharacterized protein OS=Cavia porcellus GN=Cttn PE=4 SV=1	CAVPO	7
842	14.21	14.3	59.11	48.44	43.11	tr|H0VYK1|H0VYK1_CAVPO	Uncharacterized protein OS=Cavia porcellus GN=BCAS2 PE=4 SV=1	CAVPO	8
843	14.2	14.3	23.22	23.22	19.19	tr|H0UTI6|H0UTI6_CAVPO	Uncharacterized protein OS=Cavia porcellus GN=SNX1 PE=4 SV=1	CAVPO	8
844	14.17	14.3	24.35	21.41	17.32	tr|H0V5U4|H0V5U4_CAVPO	Uncharacterized protein (Fragment) OS=Cavia porcellus GN=Eif4b PE=4 SV=1	CAVPO	10
845	14.14	14.2	39.02	30.23	27.65	tr|H0VX68|H0VX68_CAVPO	Uncharacterized protein OS=Cavia porcellus GN=NAP1L4 PE=3 SV=1	CAVPO	10
846	14.13	14.3	31.74	28.77	21.92	tr|H0VH03|H0VH03_CAVPO	Uncharacterized protein (Fragment) OS=Cavia porcellus GN=TMX3 PE=4 SV=1	CAVPO	8
847	14.12	14.4	63.91	60	57.83	tr|H0VL12|H0VL12_CAVPO	Uncharacterized protein OS=Cavia porcellus GN=UCHL3 PE=4 SV=1	CAVPO	10
848	14.12	15.4	32.2	27.43	17.89	tr|H0VY56|H0VY56_CAVPO	Uncharacterized protein OS=Cavia porcellus GN=LOC100713505 PE=4 SV=1	CAVPO	10
849	14.09	22.4	61.38	60.98	60.98	tr|H0WBK0|H0WBK0_CAVPO	Uncharacterized protein OS=Cavia porcellus GN=YWHAB PE=3 SV=1	CAVPO	23
850	14.05	14.4	62.43	52.91	48.15	tr|H0VNW9|H0VNW9_CAVPO	Uncharacterized protein OS=Cavia porcellus GN=HEBP1 PE=4 SV=1	CAVPO	8
851	14.04	14	60.47	60.47	53.49	tr|H0V2V6|H0V2V6_CAVPO	Galectin OS=Cavia porcellus GN=LGALSL PE=4 SV=1	CAVPO	8

852	14.04	14	23.62	21.77	21.77	tr|H0VA62|H0VA62_CAVPO	Uncharacterized protein OS=Cavia porcellus GN=SLC1A3 PE=4 SV=1	CAVPO	14
853	14.02	14	28.33	24.91	24.91	tr|H0UXF8|H0UXF8_CAVPO	Uncharacterized protein OS=Cavia porcellus GN=ELMOD2 PE=4 SV=1	CAVPO	8
854	14.02	14.1	40	31.65	25.82	tr|H0W1G1|H0W1G1_CAVPO	Uncharacterized protein OS=Cavia porcellus GN=TERF2IP PE=4 SV=1	CAVPO	8
855	14	14	53.38	53.38	53.38	tr|H0UTX4|H0UTX4_CAVPO	40S ribosomal protein S12 OS=Cavia porcellus GN=Rps12 PE=3 SV=1	CAVPO	8
856	14	14	32	30.4	30.4	tr|H0VJ66|H0VJ66_CAVPO	Uncharacterized protein OS=Cavia porcellus GN=NDRG3 PE=4 SV=1	CAVPO	7
857	13.97	14.5	19.25	15.34	9.311	tr|H0UWK4|H0UWK4_CAVPO	Uncharacterized protein OS=Cavia porcellus GN=Smchd1 PE=4 SV=1	CAVPO	9
858	13.96	14	28.8	28.53	25.27	tr|H0V755|H0V755_CAVPO	Uncharacterized protein (Fragment) OS=Cavia porcellus GN=ADPRHL2 PE=4 SV=1	CAVPO	7
859	13.96	14.1	13.37	10.59	6.441	tr|H0VB24|H0VB24_CAVPO	Uncharacterized protein (Fragment) OS=Cavia porcellus PE=4 SV=1	CAVPO	7
860	13.95	14.1	24.47	21.73	21.73	tr|H0VMP9|H0VMP9_CAVPO	Uncharacterized protein (Fragment) OS=Cavia porcellus GN=SCARB2 PE=4 SV=1	CAVPO	8
861	13.92	14.1	25.77	23.77	19.78	tr|H0VRZ2|H0VRZ2_CAVPO	Uncharacterized protein OS=Cavia porcellus GN=METTL16 PE=4 SV=1	CAVPO	8
862	13.91	14	22.81	17.89	16.49	tr|H0UYT2|H0UYT2_CAVPO	Uncharacterized protein OS=Cavia porcellus GN=VPS45 PE=4 SV=1	CAVPO	9
863	13.89	13.9	41.38	33.45	28.97	tr|H0UZW4|H0UZW4_CAVPO	Uncharacterized protein (Fragment) OS=Cavia porcellus GN=FHL1 PE=4 SV=1	CAVPO	9
864	13.88	14.2	18.86	14.68	9.539	tr|H0VMZ6|H0VMZ6_CAVPO	Uncharacterized protein OS=Cavia porcellus GN=MTHFD1 PE=3 SV=1	CAVPO	7
865	13.87	14	34.03	22.96	20.46	tr|H0VE77|H0VE77_CAVPO	Uncharacterized protein OS=Cavia porcellus GN=PPM1B PE=3 SV=1	CAVPO	9

866	13.83	50.5	25.38	18.93	13.13	tr|H0UUY7|H0UUY7_CAVPO	Uncharacterized protein OS=Cavia porcellus GN=MAP1A PE=4 SV=1	CAVPO	25
867	13.83	14.1	22.75	10.83	8.28	tr|H0UXI3|H0UXI3_CAVPO	Uncharacterized protein OS=Cavia porcellus GN=SRGAP3 PE=4 SV=1	CAVPO	7
868	13.83	13.9	28.04	26.74	22.61	tr|H0V4J7|H0V4J7_CAVPO	Uncharacterized protein (Fragment) OS=Cavia porcellus GN=BLMH PE=4 SV=1	CAVPO	9
869	13.82	14.2	40.06	36.69	30.53	tr|H0VN79|H0VN79_CAVPO	Uncharacterized protein (Fragment) OS=Cavia porcellus GN=Sept3 PE=3 SV=1	CAVPO	10
870	13.8	13.8	50.69	43.78	43.78	tr|H0VZ77|H0VZ77_CAVPO	Uncharacterized protein OS=Cavia porcellus GN=PRDX5 PE=4 SV=1	CAVPO	8
871	13.78	13.9	32.84	29.1	29.1	tr|H0VLK8|H0VLK8_CAVPO	Uncharacterized protein OS=Cavia porcellus GN=Rad23b PE=4 SV=1	CAVPO	7
872	13.72	13.8	32.69	31.04	31.04	tr|H0VI56|H0VI56_CAVPO	Uncharacterized protein OS=Cavia porcellus GN=NDRG1 PE=4 SV=1	CAVPO	7
873	13.72	13.8	44.16	43.29	39.39	tr|H0WD22|H0WD22_CAVPO	Uncharacterized protein (Fragment) OS=Cavia porcellus GN=Vapb PE=4 SV=1	CAVPO	7
874	13.71	13.8	40.75	36.64	34.25	tr|H0V324|H0V324_CAVPO	Uncharacterized protein OS=Cavia porcellus GN=CDK5 PE=4 SV=1	CAVPO	8
875	13.7	13.7	34.11	32.11	26.09	tr|H0VBK0|H0VBK0_CAVPO	Uncharacterized protein (Fragment) OS=Cavia porcellus GN=LZTFL1 PE=4 SV=1	CAVPO	8
876	13.68	13.8	49.74	48.72	45.64	tr|H0VL90|H0VL90_CAVPO	Uncharacterized protein OS=Cavia porcellus GN=PGRMC1 PE=4 SV=1	CAVPO	10
877	13.68	13.9	41.67	32.07	28.79	tr|H0VUU0|H0VUU0_CAVPO	Obg-like ATPase 1 OS=Cavia porcellus GN=Ola1 PE=3 SV=1	CAVPO	10
878	13.67	13.8	36.13	33.53	33.53	tr|H0UZW0|H0UZW0_CAVPO	Uncharacterized protein OS=Cavia porcellus GN=SUCLG1 PE=3 SV=1	CAVPO	10
879	13.66	13.7	49.02	49.02	48.53	tr|H0UX57|H0UX57_CAVPO	Rho GDP-dissociation inhibitor 1 OS=Cavia porcellus GN=ARHGDIA PE=4 SV=1	CAVPO	9

880	13.63	13.8	21.48	19.47	12.21	tr|H0W7I3|H0W7I3_CAVPO	Uncharacterized protein (Fragment) OS=Cavia porcellus GN=CACNA2D2 PE=4 SV=1	CAVPO	7
881	13.62	17.6	7.792	3.043	1.706	tr|H0V4N0|H0V4N0_CAVPO	Uncharacterized protein (Fragment) OS=Cavia porcellus GN=DST PE=4 SV=1	CAVPO	10
882	13.61	14	25.06	19.86	12.29	tr|H0VJH3|H0VJH3_CAVPO	Uncharacterized protein OS=Cavia porcellus GN=AGO1 PE=4 SV=1	CAVPO	8
883	13.61	13.7	21.96	17.97	17.97	tr|H0VST5|H0VST5_CAVPO	Uncharacterized protein OS=Cavia porcellus GN=CPSF6 PE=4 SV=1	CAVPO	9
884	13.59	13.8	16.35	9.03	7.383	tr|H0WCJ1|H0WCJ1_CAVPO	Uncharacterized protein OS=Cavia porcellus GN=PTPN23 PE=4 SV=1	CAVPO	8
885	13.57	13.7	47.37	47.37	47.37	tr|H0V472|H0V472_CAVPO	Uncharacterized protein OS=Cavia porcellus GN=U2AF1 PE=4 SV=1	CAVPO	11
886	13.56	13.7	21.2	10.95	8.722	tr|H0V6M3|H0V6M3_CAVPO	Uncharacterized protein (Fragment) OS=Cavia porcellus GN=OPA1 PE=3 SV=1	CAVPO	8
887	13.56	13.6	42.01	41.64	33.83	tr|H0VK78|H0VK78_CAVPO	Uncharacterized protein (Fragment) OS=Cavia porcellus GN=ELAVL1 PE=4 SV=1	CAVPO	9
888	13.51	13.6	57.66	54.95	49.55	tr|H0VKS2|H0VKS2_CAVPO	Cytochrome b-c1 complex subunit 7 OS=Cavia porcellus GN=UQCRB PE=3 SV=1	CAVPO	7
889	13.49	13.5	28.48	27.51	24.92	tr|H0V2N0|H0V2N0_CAVPO	Uncharacterized protein OS=Cavia porcellus GN=Atp1b1 PE=3 SV=1	CAVPO	7
890	13.47	13.5	64.11	55.02	55.02	tr|H0VJX3|H0VJX3_CAVPO	Uncharacterized protein (Fragment) OS=Cavia porcellus GN=Sarnp PE=4 SV=1	CAVPO	7
891	13.45	13.5	46.72	46.31	39.75	tr|H0VC50|H0VC50_CAVPO	Uncharacterized protein (Fragment) OS=Cavia porcellus GN=QDPR PE=4 SV=1	CAVPO	8
892	13.43	14.4	14.17	12.33	7.084	tr|H0V3B0|H0V3B0_CAVPO	Uncharacterized protein (Fragment) OS=Cavia porcellus GN=XPO7 PE=4 SV=1	CAVPO	8
893	13.41	13.6	23.25	18.03	15.54	tr|H0UYN9|H0UYN9_CAVPO	Uncharacterized protein OS=Cavia porcellus GN=RBM25 PE=4 SV=1	CAVPO	9

894	13.41	13.5	53.42	52.99	52.99	tr|H0VN17|H0VN17_CAVPO	Proteasome subunit alpha type OS=Cavia porcellus GN=Psma2 PE=3 SV=1	CAVPO	11
895	13.39	15.6	53.96	53.96	50	tr|H0VI61|H0VI61_CAVPO	Uncharacterized protein OS=Cavia porcellus GN=LOC100724499 PE=4 SV=1	CAVPO	9
896	13.38	13.5	49.74	46.03	46.03	tr|H0VHH8|H0VHH8_CAVPO	Uncharacterized protein OS=Cavia porcellus GN=KRAS PE=4 SV=1	CAVPO	8
897	13.34	13.5	32.34	30.03	27.39	tr|H0V8T5|H0V8T5_CAVPO	Uncharacterized protein (Fragment) OS=Cavia porcellus GN=CRK PE=4 SV=1	CAVPO	7
898	13.34	13.5	59.07	52.33	47.15	tr|H0VQ69|H0VQ69_CAVPO	Uncharacterized protein OS=Cavia porcellus GN=RHOA PE=3 SV=1	CAVPO	10
899	13.33	13.4	44.9	38.1	31.63	tr|H0VFX8|H0VFX8_CAVPO	Uncharacterized protein OS=Cavia porcellus GN=LRRC59 PE=4 SV=1	CAVPO	7
900	13.29	13.4	59.34	51.1	51.1	tr|H0UU46|H0UU46_CAVPO	Uncharacterized protein (Fragment) OS=Cavia porcellus GN=Vps29 PE=4 SV=1	CAVPO	7
901	13.28	13.3	49.5	49.5	46.5	tr|H0VU00|H0VU00_CAVPO	Uncharacterized protein OS=Cavia porcellus GN=GUCA1A PE=4 SV=1	CAVPO	8
902	13.27	13.3	59.8	59.8	59.8	tr|H0VUC2|H0VUC2_CAVPO	Uncharacterized protein (Fragment) OS=Cavia porcellus GN=HSPE1 PE=3 SV=1	CAVPO	7
903	13.26	13.4	81.15	80.33	80.33	tr|H0WCV4|H0WCV4_CAVPO	Uncharacterized protein (Fragment) OS=Cavia porcellus GN=PCP2 PE=4 SV=1	CAVPO	10
904	13.25	13.4	34.75	29.08	24.35	tr|H0VZ95|H0VZ95_CAVPO	Uncharacterized protein (Fragment) OS=Cavia porcellus GN=ACADSB PE=3 SV=1	CAVPO	7
905	13.24	13.5	30.4	24.07	12.35	tr|H0VQM0|H0VQM0_CAVPO	Uncharacterized protein OS=Cavia porcellus GN=MPP4 PE=4 SV=1	CAVPO	7
906	13.19	13.2	33.77	23.82	23.82	tr|H0VMM4|H0VMM4_CAVPO	Uncharacterized protein OS=Cavia porcellus GN=ACOT7 PE=4 SV=1	CAVPO	8
907	13.17	13.3	54.23	54.23	54.23	tr|H0VP39|H0VP39_CAVPO	Uncharacterized protein OS=Cavia porcellus GN=COTL1 PE=4 SV=1	CAVPO	8

908	13.16	13.4	19.46	19.29	16.61	tr|H0V8K5|H0V8K5_CAVPO	Uncharacterized protein OS=Cavia porcellus GN=SLC17A7 PE=4 SV=1	CAVPO	7
909	13.15	13.2	36	35.56	35.56	tr|H0V3F6|H0V3F6_CAVPO	Uncharacterized protein (Fragment) OS=Cavia porcellus GN=TMED10 PE=3 SV=1	CAVPO	7
910	13.14	13.3	44.05	44.05	39.21	tr|H0V1I1|H0V1I1_CAVPO	Uncharacterized protein OS=Cavia porcellus GN=NUDT21 PE=4 SV=1	CAVPO	8
911	13.13	13.4	37.54	29.77	29.77	tr|H0VGU6|H0VGU6_CAVPO	Uncharacterized protein OS=Cavia porcellus GN=FN3KRP PE=4 SV=1	CAVPO	8
912	13.13	13.2	39.89	39.89	33.33	tr|H0W8M2|H0W8M2_CAVPO	Uncharacterized protein OS=Cavia porcellus GN=CBX3 PE=4 SV=1	CAVPO	7
913	13.12	13.2	43.98	37.05	37.05	tr|H0VQC8|H0VQC8_CAVPO	Uncharacterized protein (Fragment) OS=Cavia porcellus GN=ASRGL1 PE=1 SV=1	CAVPO	8
914	13.11	13.3	27.73	20.6	12.52	tr|H0UTP3|H0UTP3_CAVPO	Uncharacterized protein (Fragment) OS=Cavia porcellus GN=GUCY1A2 PE=3 SV=1	CAVPO	6
915	13.1	15.4	52.61	48.34	45.5	tr|H0VCP3|H0VCP3_CAVPO	Uncharacterized protein OS=Cavia porcellus GN=Rac1 PE=3 SV=1	CAVPO	9
916	13.08	13.3	30.2	16.87	13.68	tr|H0VGH8|H0VGH8_CAVPO	Uncharacterized protein OS=Cavia porcellus GN=CTNNBL1 PE=4 SV=1	CAVPO	8
917	13.08	13.1	24.18	22.44	20.5	tr|H0VKC2|H0VKC2_CAVPO	Uncharacterized protein (Fragment) OS=Cavia porcellus GN=TXNRD1 PE=3 SV=1	CAVPO	7
918	13.08	13.2	34.85	32.58	32.58	tr|H0VKQ8|H0VKQ8_CAVPO	Uncharacterized protein (Fragment) OS=Cavia porcellus GN=ECHS1 PE=3 SV=1	CAVPO	7
919	13.06	20.8	38.36	33.96	28.72	tr|H0VUZ3|H0VUZ3_CAVPO	Uncharacterized protein OS=Cavia porcellus GN=CTNNA2 PE=4 SV=1	CAVPO	13
920	13.03	13.1	49.02	37.58	26.47	tr|H0V5Z0|H0V5Z0_CAVPO	Uncharacterized protein (Fragment) OS=Cavia porcellus GN=ABHD10 PE=4 SV=1	CAVPO	7
921	13.02	13.2	79.73	64.86	55.41	tr|H0VCC3|H0VCC3_CAVPO	Guanine nucleotide-binding protein subunit gamma OS=Cavia porcellus GN=GNGT1 PE=3	CAVPO	10

							SV=1		
922	13	13.2	29.69	26.82	18.01	tr|H0V9V3|H0V9V3_CAVPO	Uncharacterized protein (Fragment) OS=Cavia porcellus GN=ZNF326 PE=4 SV=1	CAVPO	10
923	12.99	13.1	41.28	39.24	24.71	tr|H0V9B5|H0V9B5_CAVPO	Uncharacterized protein OS=Cavia porcellus GN=CDC42EP4 PE=4 SV=1	CAVPO	6
924	12.98	13.1	60.21	47.64	42.93	tr|H0UUN1|H0UUN1_CAVPO	Uncharacterized protein OS=Cavia porcellus GN=VSNL1 PE=4 SV=1	CAVPO	7
925	12.98	13	32.39	29.25	29.25	tr|H0VU35|H0VU35_CAVPO	Uncharacterized protein OS=Cavia porcellus GN=PRPS1 PE=4 SV=1	CAVPO	7
926	12.95	13.1	26.72	22.5	14.84	tr|H0VI00|H0VI00_CAVPO	Uncharacterized protein OS=Cavia porcellus GN=PCK2 PE=3 SV=1	CAVPO	6
927	12.94	13.5	11.94	4.146	2.034	tr|H0V437|H0V437_CAVPO	Uncharacterized protein (Fragment) OS=Cavia porcellus GN=AKAP9 PE=4 SV=1	CAVPO	7
928	12.92	13	29.62	16.82	15.72	tr|H0VSJ7|H0VSJ7_CAVPO	Uncharacterized protein (Fragment) OS=Cavia porcellus GN=NCOA5 PE=4 SV=1	CAVPO	8
929	12.91	13	52.73	52.73	42.42	tr|H0V5S5|H0V5S5_CAVPO	Uncharacterized protein (Fragment) OS=Cavia porcellus GN=DSTN PE=4 SV=1	CAVPO	8
930	12.89	13	34.08	27.93	27.93	tr|H0UVC6|H0UVC6_CAVPO	Uncharacterized protein OS=Cavia porcellus GN=DNAJB11 PE=4 SV=1	CAVPO	7
931	12.86	13	35.38	35.1	26.74	tr|H0V290|H0V290_CAVPO	Uncharacterized protein (Fragment) OS=Cavia porcellus GN=LMAN2 PE=4 SV=1	CAVPO	8
932	12.83	13.1	25.62	20.22	13.99	tr|H0W1W7|H0W1W7_CAVPO	Uncharacterized protein OS=Cavia porcellus GN=SARM1 PE=4 SV=1	CAVPO	8
933	12.82	13.1	26.46	26.46	18.97	tr|H0UY99|H0UY99_CAVPO	Uncharacterized protein (Fragment) OS=Cavia porcellus GN=RCC2 PE=4 SV=1	CAVPO	8
934	12.81	12.9	39.05	38.69	35.04	tr|H0VN38|H0VN38_CAVPO	Uncharacterized protein OS=Cavia porcellus GN=STX12 PE=3 SV=1	CAVPO	8

935	12.8	13.1	26.82	21.36	19.32	tr|H0W1R5|H0W1R5_CAVPO	Uncharacterized protein OS=Cavia porcellus GN=SNRNP70 PE=4 SV=1	CAVPO	8
936	12.79	12.9	31.83	31.83	31.51	tr|H0V8S4|H0V8S4_CAVPO	Uncharacterized protein (Fragment) OS=Cavia porcellus GN=CNN3 PE=4 SV=1	CAVPO	7
937	12.78	13.2	28.16	27.91	21.36	tr|H0V901|H0V901_CAVPO	Uncharacterized protein OS=Cavia porcellus GN=DNAJA2 PE=3 SV=1	CAVPO	7
938	12.74	12.8	29.24	25.85	23.76	tr|H0VBP6|H0VBP6_CAVPO	Uncharacterized protein OS=Cavia porcellus GN=HDHD2 PE=4 SV=1	CAVPO	6
939	12.73	25.2	37.01	27.94	27.94	tr|H0VLE0|H0VLE0_CAVPO	Uncharacterized protein OS=Cavia porcellus GN=SYNCRIP PE=4 SV=1	CAVPO	13
940	12.71	12.8	35.5	35.5	27.36	tr|H0V679|H0V679_CAVPO	Uncharacterized protein (Fragment) OS=Cavia porcellus GN=PPT1 PE=4 SV=1	CAVPO	8
941	12.68	12.8	15.54	8.163	7.3	tr|H0V6V0|H0V6V0_CAVPO	Uncharacterized protein OS=Cavia porcellus GN=SIN3A PE=4 SV=1	CAVPO	8
942	12.67	12.8	40.62	35.38	30.46	tr|H0VKG2|H0VKG2_CAVPO	Uncharacterized protein (Fragment) OS=Cavia porcellus GN=EIF3I PE=3 SV=1	CAVPO	9
943	12.65	13	18.34	12.43	6.437	tr|H0V4K2|H0V4K2_CAVPO	Uncharacterized protein OS=Cavia porcellus GN=VCL PE=4 SV=1	CAVPO	6
944	12.65	12.7	32.06	26.65	21.64	tr|H0W2I6|H0W2I6_CAVPO	Serine/threonine-protein phosphatase OS=Cavia porcellus GN=PPP5C PE=3 SV=1	CAVPO	8
945	12.64	12.8	18.84	16.43	12.46	tr|H0VEI9|H0VEI9_CAVPO	Uncharacterized protein (Fragment) OS=Cavia porcellus GN=KIF2A PE=4 SV=1	CAVPO	7
946	12.61	13.1	25.71	19.28	13.01	tr|H0V9L6|H0V9L6_CAVPO	Uncharacterized protein (Fragment) OS=Cavia porcellus GN=SCFD1 PE=4 SV=1	CAVPO	6
947	12.61	12.7	37.65	37.65	30.2	tr|Q0QEZ1|Q0QEZ1_CAVPO	Succinate dehydrogenase complex subunit B (Fragment) OS=Cavia porcellus GN=SDHB PE=2 SV=1	CAVPO	7
948	12.6	12.7	29.69	29.69	29.69	tr|H0V3E6|H0V3E6_CAVPO	Uncharacterized protein (Fragment) OS=Cavia	CAVPO	8

							porcellus GN=GBAS PE=4 SV=1		
949	12.58	12.7	17.19	15.44	10.53	tr|H0VAW7|H0VAW7_CAVPO	Uncharacterized protein (Fragment) OS=Cavia porcellus GN=SLC1A2 PE=4 SV=1	CAVPO	8
950	12.57	12.7	86.96	86.96	83.48	tr|H0VNX3|H0VNX3_CAVPO	Uncharacterized protein OS=Cavia porcellus GN=Mif PE=4 SV=1	CAVPO	19
951	12.56	12.7	56.25	52.34	52.34	tr|H0UTQ8|H0UTQ8_CAVPO	Uncharacterized protein OS=Cavia porcellus GN=Uba52 PE=4 SV=1	CAVPO	15
952	12.56	12.6	48.71	48.71	47.41	tr|H0V323|H0V323_CAVPO	Uncharacterized protein (Fragment) OS=Cavia porcellus GN=PAFAH1B3 PE=4 SV=1	CAVPO	7
953	12.53	12.8	23.46	11.61	9.674	tr|H0VI01|H0VI01_CAVPO	Uncharacterized protein OS=Cavia porcellus GN=AP1G1 PE=4 SV=1	CAVPO	7
954	12.52	12.8	33.42	30.45	20.79	tr|H0UX08|H0UX08_CAVPO	Uncharacterized protein (Fragment) OS=Cavia porcellus GN=ARPC1A PE=4 SV=1	CAVPO	8
955	12.52	12.6	36.33	33.71	31.09	tr|H0V742|H0V742_CAVPO	Uncharacterized protein (Fragment) OS=Cavia porcellus GN=NDUFS3 PE=3 SV=1	CAVPO	7
956	12.51	12.6	35.42	33.54	25.08	tr|H0V0I3|H0V0I3_CAVPO	Annexin (Fragment) OS=Cavia porcellus GN=ANXA5 PE=3 SV=1	CAVPO	7
957	12.51	14.3	56.52	49.8	37.94	tr|H0V268|H0V268_CAVPO	Uncharacterized protein OS=Cavia porcellus GN=CLIC4 PE=4 SV=1	CAVPO	10
958	12.5	13.2	14.9	9.785	6.079	tr|H0VCB3|H0VCB3_CAVPO	Uncharacterized protein (Fragment) OS=Cavia porcellus GN=RAB3GAP2 PE=4 SV=1	CAVPO	7
959	12.5	12.7	28.46	21.05	16.57	tr|H0VIU6|H0VIU6_CAVPO	Uncharacterized protein OS=Cavia porcellus GN=PPM1H PE=4 SV=1	CAVPO	7
960	12.48	12.7	36.21	31.03	18.23	tr|H0UX53|H0UX53_CAVPO	Uncharacterized protein OS=Cavia porcellus GN=PCYT2 PE=4 SV=1	CAVPO	7
961	12.47	12.7	22.06	18.48	12.61	tr|H0VAT4|H0VAT4_CAVPO	Uncharacterized protein OS=Cavia porcellus GN=APPL1 PE=4 SV=1	CAVPO	7

962	12.46	12.6	41.8	36.07	33.61	tr|H0VJ37|H0VJ37_CAVPO	Uncharacterized protein (Fragment) OS=Cavia porcellus GN=LMAN1 PE=4 SV=1	CAVPO	13
963	12.46	12.8	26.27	22.73	13.98	tr|H0VJC0|H0VJC0_CAVPO	Uncharacterized protein OS=Cavia porcellus GN=CPT2 PE=3 SV=1	CAVPO	7
964	12.45	12.5	32.81	29.69	25.62	tr|H0VVS6|H0VVS6_CAVPO	Uncharacterized protein (Fragment) OS=Cavia porcellus GN=PGP PE=4 SV=1	CAVPO	6
965	12.43	12.5	47.72	43.57	41.08	tr|H0UVM8|H0UVM8_CAVPO	Proteasome subunit beta type OS=Cavia porcellus GN=PSMB1 PE=3 SV=1	CAVPO	7
966	12.42	12.6	44.05	44.05	38.33	tr|H0V6U7|H0V6U7_CAVPO	Uncharacterized protein OS=Cavia porcellus GN=SPAG7 PE=4 SV=1	CAVPO	7
967	12.39	12.5	8.524	6.346	5.295	tr|H0UXB9|H0UXB9_CAVPO	Uncharacterized protein OS=Cavia porcellus GN=PRRC2C PE=4 SV=1	CAVPO	8
968	12.38	12.5	36.87	18.84	15.83	tr|H0VJC1|H0VJC1_CAVPO	Uncharacterized protein OS=Cavia porcellus GN=PRPF31 PE=4 SV=1	CAVPO	6
969	12.38	12.5	36.06	36.06	32.96	tr|H0VZD0|H0VZD0_CAVPO	Uncharacterized protein OS=Cavia porcellus GN=G6PC2 PE=4 SV=1	CAVPO	11
970	12.37	12.8	32.02	28.48	16.42	tr|H0VPJ5|H0VPJ5_CAVPO	Aldehyde dehydrogenase OS=Cavia porcellus GN=ALDH3A2 PE=3 SV=1	CAVPO	7
971	12.37	12.5	61.86	61.86	55.93	tr|H0W8B0|H0W8B0_CAVPO	Uncharacterized protein (Fragment) OS=Cavia porcellus GN=Snrpd2 PE=4 SV=1	CAVPO	9
972	12.36	12.4	30.39	26.47	22.55	tr|H0W3C2|H0W3C2_CAVPO	Aminoacylase-1 OS=Cavia porcellus GN=ACY1 PE=3 SV=1	CAVPO	7
973	12.35	12.4	20	18.92	16.77	tr|H0VX80|H0VX80_CAVPO	Uncharacterized protein OS=Cavia porcellus GN=SLC16A3 PE=4 SV=1	CAVPO	8
974	12.33	12.6	38.76	30.86	23.21	tr|H0V217|H0V217_CAVPO	Uncharacterized protein OS=Cavia porcellus GN=PSMC4 PE=3 SV=1	CAVPO	7
975	12.33	12.4	52.59	35.34	28.02	tr|H0VT21|H0VT21_CAVPO	Small nuclear ribonucleoprotein-associated protein OS=Cavia porcellus PE=3 SV=1	CAVPO	8

976	12.31	12.6	24.06	14.23	9.702	tr|H0V9U8|H0V9U8_CAVPO	Uncharacterized protein OS=Cavia porcellus GN=PANK4 PE=4 SV=1	CAVPO	6
977	12.3	12.7	29.04	29.04	29.04	tr|H0V063|H0V063_CAVPO	Uncharacterized protein OS=Cavia porcellus GN=CRKL PE=4 SV=1	CAVPO	8
978	12.28	23.3	66.67	62.83	49.85	tr|H0V1D4|H0V1D4_CAVPO	Uncharacterized protein OS=Cavia porcellus GN=PCBP3 PE=4 SV=1	CAVPO	14
979	12.28	12.5	36.46	29.64	23.03	tr|H0V4W8|H0V4W8_CAVPO	Uncharacterized protein OS=Cavia porcellus GN=SAMM50 PE=4 SV=1	CAVPO	7
980	12.23	14.4	43.82	43.46	32.51	tr|H0VH21|H0VH21_CAVPO	Uncharacterized protein OS=Cavia porcellus GN=VDAC3 PE=4 SV=1	CAVPO	9
981	12.22	14.4	20.57	18.53	18.33	tr|H0VIV2|H0VIV2_CAVPO	Uncharacterized protein (Fragment) OS=Cavia porcellus GN=Slc2a3 PE=3 SV=1	CAVPO	8
982	12.18	12.4	37.55	37.55	28.82	tr|H0V6B5|H0V6B5_CAVPO	Uncharacterized protein OS=Cavia porcellus GN=CLTB PE=4 SV=1	CAVPO	9
983	12.16	12.3	23.24	20.23	18.56	tr|H0W2A0|H0W2A0_CAVPO	Transporter OS=Cavia porcellus GN=SLC6A1 PE=3 SV=1	CAVPO	11
984	12.13	12.2	20.92	19.08	18.9	tr|H0V2W8|H0V2W8_CAVPO	Uncharacterized protein (Fragment) OS=Cavia porcellus GN=SLC1A5 PE=4 SV=1	CAVPO	10
985	12.12	12.2	34.11	31.4	31.4	tr|H0VQL2|H0VQL2_CAVPO	Uncharacterized protein (Fragment) OS=Cavia porcellus GN=ATP1B2 PE=3 SV=1	CAVPO	11
986	12.11	12.3	22.71	18.19	15.51	tr|H0UU14|H0UU14_CAVPO	Uncharacterized protein OS=Cavia porcellus GN=SSRP1 PE=4 SV=1	CAVPO	8
987	12.1	12.2	18.01	18.01	16.86	tr|H0VU17|H0VU17_CAVPO	Uncharacterized protein (Fragment) OS=Cavia porcellus GN=CAPRIN1 PE=4 SV=1	CAVPO	9
988	12.09	12.1	65.15	52.53	52.53	tr|H0V9U0|H0V9U0_CAVPO	Uncharacterized protein OS=Cavia porcellus GN=CDKN1B PE=4 SV=1	CAVPO	7
989	12.09	12.2	63.45	48.97	48.97	tr|H0VQZ0|H0VQZ0_CAVPO	Uncharacterized protein OS=Cavia porcellus GN=UBE2V2 PE=4 SV=1	CAVPO	11

990	12.07	12.1	59.18	54.42	54.42	tr|H0UWE7|H0UWE7_CAVPO	Uncharacterized protein OS=Cavia porcellus GN=LOC100720944 PE=3 SV=1	CAVPO	7
991	12.06	12.1	56.25	47.16	47.16	tr|H0VJN3|H0VJN3_CAVPO	Uncharacterized protein OS=Cavia porcellus GN=NDUFB10 PE=4 SV=1	CAVPO	6
992	12.05	14.3	51.02	37.25	26.64	tr|H0UYJ4|H0UYJ4_CAVPO	Uncharacterized protein (Fragment) OS=Cavia porcellus GN=PSMC1 PE=3 SV=1	CAVPO	8
993	12.04	12.1	21.62	15.58	15.58	tr|H0VES1|H0VES1_CAVPO	Uncharacterized protein (Fragment) OS=Cavia porcellus GN=Ewsr1 PE=4 SV=1	CAVPO	10
994	12.03	12.1	56.22	42.49	42.49	tr|H0VLQ7|H0VLQ7_CAVPO	Uncharacterized protein OS=Cavia porcellus GN=MSRA PE=3 SV=1	CAVPO	6
995	12.02	12.2	27.33	25.06	19.13	tr|H0VMU7|H0VMU7_CAVPO	Uncharacterized protein OS=Cavia porcellus GN=ARHGAP1 PE=4 SV=1	CAVPO	7
996	12	12	31.25	31.25	31.25	tr|H0VFW6|H0VFW6_CAVPO	Uncharacterized protein (Fragment) OS=Cavia porcellus GN=LASP1 PE=4 SV=1	CAVPO	8
997	11.98	12.3	27.45	14.67	9.511	tr|H0V2Y9|H0V2Y9_CAVPO	Uncharacterized protein OS=Cavia porcellus GN=GYS1 PE=4 SV=1	CAVPO	6
998	11.98	12.1	15.91	10.83	10.06	tr|H0V481|H0V481_CAVPO	Uncharacterized protein (Fragment) OS=Cavia porcellus GN=SORBS2 PE=4 SV=1	CAVPO	7
999	11.96	12.2	31.65	23.84	20.25	tr|H0V6Y2|H0V6Y2_CAVPO	Uncharacterized protein OS=Cavia porcellus GN=ATXN10 PE=4 SV=1	CAVPO	7
1000	11.95	12	42.41	42.41	32.28	tr|H0VGV8|H0VGV8_CAVPO	Uncharacterized protein OS=Cavia porcellus GN=UBE2I PE=3 SV=1	CAVPO	6
1001	11.92	15.1	61.97	56.69	50.7	tr|H0VR94|H0VR94_CAVPO	Uncharacterized protein (Fragment) OS=Cavia porcellus GN=CAPZA1 PE=4 SV=1	CAVPO	8
1002	11.91	12	53.57	52.55	48.98	tr|H0V261|H0V261_CAVPO	Uncharacterized protein (Fragment) OS=Cavia porcellus GN=TXNDC12 PE=4 SV=1	CAVPO	6
1003	11.9	12	23.55	20.34	17.77	tr|H0VSX5|H0VSX5_CAVPO	Uncharacterized protein OS=Cavia porcellus GN=PPP2R5E PE=4 SV=1	CAVPO	9

1004	11.87	11.9	46.88	32.14	28.13	tr|H0UW80|H0UW80_CAVPO	Peptidyl-prolyl cis-trans isomerase OS=Cavia porcellus GN=FKBP3 PE=4 SV=1	CAVPO	6
1005	11.86	12	28.47	22.22	20.14	tr|H0VI51|H0VI51_CAVPO	Uncharacterized protein OS=Cavia porcellus GN=KIAA1715 PE=4 SV=1	CAVPO	6
1006	11.85	12.1	34.93	28.47	18.18	tr|H0VJC7|H0VJC7_CAVPO	Uncharacterized protein OS=Cavia porcellus GN=BZW2 PE=4 SV=1	CAVPO	7
1007	11.81	11.9	59.02	56.59	56.59	tr|H0UZS2|H0UZS2_CAVPO	Uncharacterized protein OS=Cavia porcellus GN=Ranbp1 PE=4 SV=1	CAVPO	8
1008	11.81	12	42.43	37.47	24.57	tr|H0VRE0|H0VRE0_CAVPO	Uncharacterized protein OS=Cavia porcellus GN=Sec14l2 PE=4 SV=1	CAVPO	7
1009	11.8	12	42.41	39.29	39.29	tr|H0V1S2|H0V1S2_CAVPO	Uncharacterized protein OS=Cavia porcellus GN=CHMP4B PE=4 SV=1	CAVPO	7
1010	11.8	12	35.56	27.92	24.58	tr|H0VGI9|H0VGI9_CAVPO	Uncharacterized protein OS=Cavia porcellus GN=SCRN3 PE=4 SV=1	CAVPO	7
1011	11.78	11.8	31.96	23.17	23.17	tr|H0WA47|H0WA47_CAVPO	Uncharacterized protein OS=Cavia porcellus GN=Dhrs7 PE=3 SV=1	CAVPO	6
1012	11.78	11.8	58.55	53.29	47.37	tr|Q53YS8|Q53YS8_CAVPO	Superoxide dismutase [Cu-Zn] (Fragment) OS=Cavia porcellus GN=SOD1 PE=2 SV=1	Cu-Zn	12
1013	11.77	11.9	42.13	37.01	27.17	tr|H0VLP7|H0VLP7_CAVPO	Uncharacterized protein OS=Cavia porcellus GN=PSME3 PE=4 SV=1	CAVPO	6
1014	11.76	11.9	24.8	24.64	15.46	tr|H0UWQ1|H0UWQ1_CAVPO	Uncharacterized protein OS=Cavia porcellus GN=IRGQ PE=4 SV=1	CAVPO	6
1015	11.76	11.9	24.27	24.04	24.04	tr|H0W5Z3|H0W5Z3_CAVPO	Uncharacterized protein OS=Cavia porcellus GN=NDUFV3 PE=4 SV=1	CAVPO	8
1016	11.75	11.8	44.74	38.82	38.82	tr|H0W478|H0W478_CAVPO	Uncharacterized protein (Fragment) OS=Cavia porcellus GN=RPS18 PE=3 SV=1	CAVPO	8
1017	11.74	11.9	28.18	22.31	13.03	tr|H0VUX9|H0VUX9_CAVPO	Uncharacterized protein OS=Cavia porcellus GN=LOC100713897 PE=4 SV=1	CAVPO	6

1018	11.74	11.8	52.07	51.61	51.61	tr|H0W2B1|H0W2B1_CAVPO	Superoxide dismutase (Fragment) OS=Cavia porcellus GN=SOD2 PE=3 SV=1	CAVPO	11
1019	11.73	11.8	46.86	32.57	29.14	tr|H0UVL7|H0UVL7_CAVPO	Uncharacterized protein OS=Cavia porcellus GN=STRAP PE=4 SV=1	CAVPO	7
1020	11.71	11.8	46.51	45.58	42.79	tr|H0W9T2|H0W9T2_CAVPO	Uncharacterized protein OS=Cavia porcellus GN=LOC100727822 PE=4 SV=1	CAVPO	8
1021	11.7	20.6	43.24	41.16	32.64	tr|H0VAT3|H0VAT3_CAVPO	Histone deacetylase OS=Cavia porcellus GN=HDAC1 PE=3 SV=1	CAVPO	12
1022	11.69	11.9	12.63	8.37	6.167	tr|H0V7U3|H0V7U3_CAVPO	Ubiquitin carboxyl-terminal hydrolase (Fragment) OS=Cavia porcellus GN=USP47 PE=3 SV=1	CAVPO	6
1023	11.68	11.9	20.79	20.32	11.75	tr|H0VDW5|H0VDW5_CAVPO	Signal recognition particle subunit SRP68 OS=Cavia porcellus GN=SRP68 PE=3 SV=1	CAVPO	6
1024	11.67	13.1	62.67	62	61.33	tr|H0WA91|H0WA91_CAVPO	Uncharacterized protein OS=Cavia porcellus GN=COX5A PE=4 SV=1	CAVPO	10
1025	11.66	23.9	34.65	24.64	22.22	tr|H0VHU1|H0VHU1_CAVPO	Uncharacterized protein OS=Cavia porcellus GN=DLG1 PE=4 SV=1	CAVPO	16
1026	11.66	11.9	29.64	22.31	9.581	tr|H0VQP4|H0VQP4_CAVPO	Uncharacterized protein OS=Cavia porcellus GN=MTA2 PE=4 SV=1	CAVPO	7
1027	11.65	11.9	57.89	54.39	51.46	tr|H0V3N6|H0V3N6_CAVPO	Uncharacterized protein OS=Cavia porcellus GN=LOC100714659 PE=4 SV=1	CAVPO	7
1028	11.65	12	46.2	42.69	26.02	tr|H0WAR7|H0WAR7_CAVPO	Uncharacterized protein OS=Cavia porcellus GN=WDR77 PE=4 SV=1	CAVPO	6
1029	11.64	11.7	48.61	48.21	38.25	tr|H0VZF2|H0VZF2_CAVPO	Uncharacterized protein OS=Cavia porcellus GN=PGLS PE=4 SV=1	CAVPO	7
1030	11.61	11.8	27.64	24.39	15.45	tr|H0V646|H0V646_CAVPO	Uncharacterized protein (Fragment) OS=Cavia porcellus GN=ADSL PE=4 SV=1	CAVPO	6
1031	11.61	11.7	79.29	52.86	52.86	tr|H0VN18|H0VN18_CAVPO	Profilin OS=Cavia porcellus GN=PFN1 PE=3	CAVPO	7

							SV=1		
1032	11.58	11.7	25.37	25.37	19.45	tr|H0UY00|H0UY00_CAVPO	Uncharacterized protein (Fragment) OS=Cavia porcellus GN=ENDOD1 PE=4 SV=1	CAVPO	6
1033	11.57	12.2	33.94	29.07	13.62	tr|H0UX84|H0UX84_CAVPO	Uncharacterized protein (Fragment) OS=Cavia porcellus GN=GPS1 PE=4 SV=1	CAVPO	5
1034	11.57	11.7	49.74	28.57	21.68	tr|H0V5Q3|H0V5Q3_CAVPO	Uncharacterized protein OS=Cavia porcellus GN=SEPHS1 PE=4 SV=1	CAVPO	6
1035	11.57	11.8	38.19	38.19	33.33	tr|H0VK45|H0VK45_CAVPO	Uncharacterized protein OS=Cavia porcellus GN=SNX12 PE=4 SV=1	CAVPO	7
1036	11.55	11.7	25.3	21.15	21.15	tr|H0VYL1|H0VYL1_CAVPO	Uncharacterized protein OS=Cavia porcellus GN=NUP62 PE=4 SV=1	CAVPO	7
1037	11.52	11.6	50.48	46.19	46.19	tr|H0V0R2|H0V0R2_CAVPO	Uncharacterized protein OS=Cavia porcellus GN=ABHD14B PE=4 SV=1	CAVPO	7
1038	11.51	11.7	44.54	29.89	27.87	tr|H0WA55|H0WA55_CAVPO	Uncharacterized protein OS=Cavia porcellus GN=TBCC PE=4 SV=1	CAVPO	7
1039	11.49	11.6	61.44	50.85	47.03	tr|H0W641|H0W641_CAVPO	Uncharacterized protein OS=Cavia porcellus GN=LOC100732059 PE=3 SV=1	CAVPO	10
1040	11.48	18.7	42.86	35.99	28.94	tr|H0V888|H0V888_CAVPO	Uncharacterized protein (Fragment) OS=Cavia porcellus GN=CAMK2A PE=4 SV=1	CAVPO	14
1041	11.48	11.6	30.84	22.75	18.86	tr|H0VAZ0|H0VAZ0_CAVPO	Zinc finger Ran-binding domain-containing protein 2 OS=Cavia porcellus GN=ZRANB2 PE=3 SV=1	CAVPO	7
1042	11.45	13.3	38.81	36.57	36.57	tr|H0VPW7|H0VPW7_CAVPO	Uncharacterized protein OS=Cavia porcellus GN=ANP32E PE=4 SV=1	CAVPO	11
1043	11.44	11.9	6.314	3.886	2.105	tr|H0VJ41|H0VJ41_CAVPO	Uncharacterized protein (Fragment) OS=Cavia porcellus GN=BSN PE=4 SV=1	CAVPO	6
1044	11.43	12.2	18.97	13.11	9.953	tr|H0V141|H0V141_CAVPO	Uncharacterized protein (Fragment) OS=Cavia porcellus GN=TNPO1 PE=4 SV=1	CAVPO	8

1045	11.43	15.6	32.53	29.76	24.22	tr|H0VTS7|H0VTS7_CAVPO	Uncharacterized protein (Fragment) OS=Cavia porcellus GN=HNRNPDL PE=4 SV=1	CAVPO	10
1046	11.42	12.8	41.62	38.38	28.65	tr|H0VPG3|H0VPG3_CAVPO	Phosphoserine aminotransferase OS=Cavia porcellus GN=Psat1 PE=3 SV=1	CAVPO	9
1047	11.42	11.5	29.55	24.72	21.31	tr|H0VT69|H0VT69_CAVPO	Eukaryotic translation initiation factor 3 subunit H OS=Cavia porcellus GN=EIF3H PE=3 SV=1	CAVPO	6
1048	11.4	11.5	35.1	26.43	21.78	tr|H0V880|H0V880_CAVPO	Uncharacterized protein (Fragment) OS=Cavia porcellus GN=G3bp1 PE=4 SV=1	CAVPO	10
1049	11.4	11.7	31.05	29.34	25.36	tr|H0VLT4|H0VLT4_CAVPO	Uncharacterized protein OS=Cavia porcellus GN=TMOD2 PE=4 SV=1	CAVPO	7
1050	11.4	11.7	17.35	14.78	11.18	tr|H0W8M9|H0W8M9_CAVPO	Uncharacterized protein OS=Cavia porcellus GN=HGS PE=4 SV=1	CAVPO	9
1051	11.38	11.5	31.61	30.97	30.97	tr|H0V643|H0V643_CAVPO	Uncharacterized protein OS=Cavia porcellus GN=FAM162A PE=4 SV=1	CAVPO	6
1052	11.36	11.4	29.22	17.81	17.81	tr|H0VG23|H0VG23_CAVPO	Uncharacterized protein OS=Cavia porcellus GN=RCC1 PE=4 SV=1	CAVPO	6
1053	11.35	11.5	22.24	18.82	16.35	tr|H0VLQ6|H0VLQ6_CAVPO	Uncharacterized protein OS=Cavia porcellus GN=CELF2 PE=4 SV=1	CAVPO	7
1054	11.35	11.4	38.42	38.42	33.16	tr|H0W3F0|H0W3F0_CAVPO	Uncharacterized protein OS=Cavia porcellus GN=CYGB PE=3 SV=1	CAVPO	6
1055	11.34	11.4	67.52	58.6	58.6	tr|H0W575|H0W575_CAVPO	Uncharacterized protein OS=Cavia porcellus GN=PIN1 PE=4 SV=1	CAVPO	8
1056	11.33	11.8	21.89	16.74	9.263	tr|H0VH29|H0VH29_CAVPO	Uncharacterized protein (Fragment) OS=Cavia porcellus GN=ATXN2L PE=4 SV=1	CAVPO	7
1057	11.32	11.5	38.38	33.84	29.29	tr|H0UT84|H0UT84_CAVPO	Uncharacterized protein (Fragment) OS=Cavia porcellus GN=NUDT5 PE=3 SV=1	CAVPO	6
1058	11.3	11.6	23.45	18.83	15.28	tr|H0UVE1|H0UVE1_CAVPO	Nicalin OS=Cavia porcellus GN=NCLN PE=3	CAVPO	8

							SV=1		
1059	11.28	28.3	52.62	49.66	43.74	tr|H0W2C0|H0W2C0_CAVPO	Uncharacterized protein OS=Cavia porcellus GN=HNRNPH2 PE=4 SV=1	CAVPO	19
1060	11.28	11.3	32.39	23.94	23.94	tr|H0WBQ6|H0WBQ6_CAVPO	Uncharacterized protein OS=Cavia porcellus GN=HIST1H1C PE=3 SV=1	CAVPO	7
1061	11.26	13.4	31.87	28.71	22.14	tr|H0UTC1|H0UTC1_CAVPO	Uncharacterized protein (Fragment) OS=Cavia porcellus GN=SMARCE1 PE=4 SV=1	CAVPO	7
1062	11.24	11.4	54.14	54.14	54.14	tr|H0VL42|H0VL42_CAVPO	Uncharacterized protein OS=Cavia porcellus GN=FABP3 PE=3 SV=1	CAVPO	7
1063	11.24	11.4	33.33	29.66	27.22	tr|H0VLJ5|H0VLJ5_CAVPO	Uncharacterized protein (Fragment) OS=Cavia porcellus GN=EIF3F PE=3 SV=1	CAVPO	7
1064	11.23	13.6	31.29	31.29	18.62	tr|H0VNJ9|H0VNJ9_CAVPO	Uncharacterized protein OS=Cavia porcellus GN=Aldh2 PE=3 SV=1	CAVPO	9
1065	11.2	11.4	24.47	24.47	22.63	tr|H0VEL9|H0VEL9_CAVPO	Uncharacterized protein OS=Cavia porcellus GN=AMACR PE=4 SV=1	CAVPO	8
1066	11.2	11.3	31.74	26.65	24.85	tr|H0VRU8|H0VRU8_CAVPO	Uncharacterized protein OS=Cavia porcellus GN=WDR5 PE=4 SV=1	CAVPO	6
1067	11.19	11.2	35.4	34.07	34.07	tr|H0W9K3|H0W9K3_CAVPO	Uncharacterized protein (Fragment) OS=Cavia porcellus GN=TMED7 PE=3 SV=1	CAVPO	7
1068	11.18	11.4	33.02	23.77	23.77	tr|H0VLQ5|H0VLQ5_CAVPO	Uncharacterized protein (Fragment) OS=Cavia porcellus GN=LCMT1 PE=4 SV=1	CAVPO	7
1069	11.17	11.4	24.96	16.59	14.33	tr|H0VGU7|H0VGU7_CAVPO	Uncharacterized protein OS=Cavia porcellus GN=FXR1 PE=4 SV=1	CAVPO	6
1070	11.15	11.4	42.86	29.5	27.64	tr|H0VGM4|H0VGM4_CAVPO	Uncharacterized protein OS=Cavia porcellus GN=SFXN1 PE=4 SV=1	CAVPO	7
1071	11.15	11.5	14.47	7.294	5.882	tr|H0W9Y0|H0W9Y0_CAVPO	Uncharacterized protein (Fragment) OS=Cavia porcellus GN=YLPM1 PE=4 SV=1	CAVPO	7

1072	11.12	11.4	13.74	7.971	2.804	tr|H0VS44|H0VS44_CAVPO	Uncharacterized protein (Fragment) OS=Cavia porcellus GN=ARFGEF1 PE=4 SV=1	CAVPO	5
1073	11.11	11.3	28.15	26.27	21.45	tr|H0UZW6|H0UZW6_CAVPO	Uncharacterized protein (Fragment) OS=Cavia porcellus GN=CAMK1D PE=4 SV=1	CAVPO	6
1074	11.1	11.2	33.18	27.61	19.49	tr|H0V8A6|H0V8A6_CAVPO	Uncharacterized protein OS=Cavia porcellus GN=DBNL PE=4 SV=1	CAVPO	6
1075	11.08	13.6	37.69	23.09	17.43	tr|H0VYG3|H0VYG3_CAVPO	Uncharacterized protein (Fragment) OS=Cavia porcellus GN=LMNA PE=3 SV=1	CAVPO	7
1076	11.07	13.8	46.62	46.26	28.11	tr|H0V4P0|H0V4P0_CAVPO	Uncharacterized protein OS=Cavia porcellus GN=MAPRE3 PE=4 SV=1	CAVPO	9
1077	11.06	11.5	17.54	15.1	10.48	tr|H0VRP0|H0VRP0_CAVPO	Uncharacterized protein (Fragment) OS=Cavia porcellus GN=SEC24A PE=4 SV=1	CAVPO	6
1078	11.03	13.2	25.77	18.85	9.744	tr|H0W3J4|H0W3J4_CAVPO	Uncharacterized protein OS=Cavia porcellus GN=OXR1 PE=4 SV=1	CAVPO	7
1079	11.02	11.3	24.34	23.11	18.61	tr|H0VI81|H0VI81_CAVPO	Uncharacterized protein OS=Cavia porcellus GN=UBXN7 PE=4 SV=1	CAVPO	7
1080	11	33.4	26.73	22.2	16.78	tr|H0V4N9|H0V4N9_CAVPO	Calcium-transporting ATPase OS=Cavia porcellus GN=ATP2B3 PE=3 SV=1	CAVPO	18
1081	10.99	11.2	17.31	13.31	7.235	tr|H0VEN5|H0VEN5_CAVPO	Uncharacterized protein OS=Cavia porcellus GN=CDK11B PE=4 SV=1	CAVPO	5
1082	10.92	11	37.4	23.58	18.7	tr|H0VI68|H0VI68_CAVPO	Uncharacterized protein OS=Cavia porcellus GN=PRPSAP2 PE=4 SV=1	CAVPO	5
1083	10.92	11.2	37.34	32.74	21.23	tr|H0VKG4|H0VKG4_CAVPO	Uncharacterized protein OS=Cavia porcellus GN=TSG101 PE=4 SV=1	CAVPO	6
1084	10.91	11.2	23.89	14.03	8.723	tr|H0UTC6|H0UTC6_CAVPO	Uncharacterized protein OS=Cavia porcellus GN=ALDH18A1 PE=4 SV=1	CAVPO	6
1085	10.9	11.2	17.83	11.17	9.481	tr|H0UYT0|H0UYT0_CAVPO	Uncharacterized protein OS=Cavia porcellus GN=SUPT16H PE=4 SV=1	CAVPO	8

1086	10.9	11	34.78	27.64	25.16	tr|H0VQV1|H0VQV1_CAVPO	Uncharacterized protein (Fragment) OS=Cavia porcellus GN=RCN2 PE=4 SV=1	CAVPO	8
1087	10.88	11.1	19.71	9.38	5.336	tr|H0VU32|H0VU32_CAVPO	Uncharacterized protein (Fragment) OS=Cavia porcellus GN=ITGA2 PE=3 SV=1	CAVPO	6
1088	10.87	13.3	39.9	39.42	35.1	tr|H0UYZ9|H0UYZ9_CAVPO	Uncharacterized protein OS=Cavia porcellus GN=RAB6C PE=3 SV=1	CAVPO	9
1089	10.87	11	33.68	30.56	27.08	tr|H0VTV5|H0VTV5_CAVPO	Uncharacterized protein (Fragment) OS=Cavia porcellus GN=LSAMP PE=4 SV=1	CAVPO	8
1090	10.86	10.9	76.52	76.52	69.57	tr|H0V6G7|H0V6G7_CAVPO	Uncharacterized protein OS=Cavia porcellus GN=Rplp2 PE=3 SV=1	CAVPO	6
1091	10.84	11	27.71	23.29	19.28	tr|H0VTA5|H0VTA5_CAVPO	Uncharacterized protein (Fragment) OS=Cavia porcellus GN=DYNC1LI2 PE=4 SV=1	CAVPO	6
1092	10.82	10.9	22.4	18.67	18.67	tr|H0V2E0|H0V2E0_CAVPO	Uncharacterized protein (Fragment) OS=Cavia porcellus GN=SH3GLB2 PE=4 SV=1	CAVPO	7
1093	10.82	11	36.31	31.85	31.21	tr|H0W551|H0W551_CAVPO	Uncharacterized protein (Fragment) OS=Cavia porcellus GN=RPS19 PE=4 SV=1	CAVPO	6
1094	10.81	10.9	53.16	41.14	41.14	tr|H0V264|H0V264_CAVPO	Uncharacterized protein OS=Cavia porcellus GN=BTF3L4 PE=4 SV=1	CAVPO	6
1095	10.81	11	35.56	32.59	22.59	tr|H0V363|H0V363_CAVPO	Uncharacterized protein OS=Cavia porcellus GN=COMT PE=4 SV=1	CAVPO	6
1096	10.8	10.9	49.32	32.19	23.63	tr|H0UUH8|H0UUH8_CAVPO	Uncharacterized protein (Fragment) OS=Cavia porcellus GN=Rpl5 PE=3 SV=1	CAVPO	5
1097	10.8	11.4	23.17	20.68	18.18	tr|H0VAR5|H0VAR5_CAVPO	Uncharacterized protein OS=Cavia porcellus GN=KRT1 PE=3 SV=1	CAVPO	11
1098	10.79	10.9	20.27	14.7	11.49	tr|H0WCN4|H0WCN4_CAVPO	Uncharacterized protein OS=Cavia porcellus GN=IGSF8 PE=4 SV=1	CAVPO	5
1099	10.77	11	16.07	11.9	10.83	tr|H0VUZ6|H0VUZ6_CAVPO	Uncharacterized protein OS=Cavia porcellus GN=MAP6 PE=4 SV=1	CAVPO	7

1100	10.76	10.9	19.01	17.84	10.8	tr|H0W3W0|H0W3W0_CAVPO	Uncharacterized protein OS=Cavia porcellus GN=KCNB1 PE=4 SV=1	CAVPO	6
1101	10.73	10.8	22.9	21.14	14.48	tr|H0W3S4|H0W3S4_CAVPO	Uncharacterized protein (Fragment) OS=Cavia porcellus PE=4 SV=1	CAVPO	6
1102	10.7	13.2	14.88	12.24	5.194	tr|H0V8S9|H0V8S9_CAVPO	Phosphoinositide phospholipase C (Fragment) OS=Cavia porcellus GN=PLCB1 PE=4 SV=1	CAVPO	5
1103	10.66	10.7	49.32	38.36	38.36	tr|H0VHP7|H0VHP7_CAVPO	Uncharacterized protein (Fragment) OS=Cavia porcellus GN=Csnk2b PE=4 SV=1	CAVPO	7
1104	10.66	10.7	27.04	18.02	18.02	tr|H0VSX9|H0VSX9_CAVPO	Uncharacterized protein OS=Cavia porcellus GN=CSTF2 PE=4 SV=1	CAVPO	6
1105	10.66	10.7	23.1	17.52	11.78	tr|H0W6N5|H0W6N5_CAVPO	Uncharacterized protein (Fragment) OS=Cavia porcellus GN=SF1 PE=4 SV=1	CAVPO	5
1106	10.64	10.7	49.29	48.57	47.14	tr|H0VPZ2|H0VPZ2_CAVPO	Uncharacterized protein OS=Cavia porcellus GN=SNCA PE=4 SV=1	CAVPO	6
1107	10.63	10.7	26.75	26.75	21.93	tr|H0VA74|H0VA74_CAVPO	Uncharacterized protein OS=Cavia porcellus GN=TSN PE=4 SV=1	CAVPO	5
1108	10.63	10.9	31.9	11.33	6.901	tr|H0VDS9|H0VDS9_CAVPO	Uncharacterized protein OS=Cavia porcellus GN=TOP1 PE=4 SV=1	CAVPO	5
1109	10.62	10.8	36.07	29.03	11.73	tr|H0VIW0|H0VIW0_CAVPO	Uncharacterized protein OS=Cavia porcellus GN=CAB39 PE=4 SV=1	CAVPO	4
1110	10.61	10.7	33.14	30.29	23.14	tr|H0W311|H0W311_CAVPO	Uncharacterized protein OS=Cavia porcellus GN=Ptgr2 PE=4 SV=1	CAVPO	5
1111	10.58	10.8	37.66	33.67	28.18	tr|H0VRS1|H0VRS1_CAVPO	Uncharacterized protein OS=Cavia porcellus GN=TFG PE=4 SV=1	CAVPO	8
1112	10.58	10.7	15.42	12.11	9.51	tr|H0W4A1|H0W4A1_CAVPO	Uncharacterized protein OS=Cavia porcellus GN=SLTM PE=4 SV=1	CAVPO	7
1113	10.55	10.6	62.21	62.21	62.21	tr|B0FJL6|B0FJL6_CAVPO	TPT1 OS=Cavia porcellus GN=TPT1 PE=2 SV=1	CAVPO	6

1114	10.54	10.7	14.9	10.17	10.17	tr|H0UUS2|H0UUS2_CAVPO	Uncharacterized protein OS=Cavia porcellus GN=EMC1 PE=4 SV=1	CAVPO	7
1115	10.54	10.8	33.95	32.41	32.41	tr|H0VXV1|H0VXV1_CAVPO	Uncharacterized protein (Fragment) OS=Cavia porcellus GN=COPS6 PE=4 SV=1	CAVPO	8
1116	10.53	10.7	29.75	23.27	17.23	tr|H0VUQ4|H0VUQ4_CAVPO	Uncharacterized protein OS=Cavia porcellus GN=BAG5 PE=4 SV=1	CAVPO	6
1117	10.5	10.6	42.94	41.1	26.38	tr|H0VC16|H0VC16_CAVPO	Uncharacterized protein (Fragment) OS=Cavia porcellus GN=PPP2R4 PE=4 SV=1	CAVPO	7
1118	10.5	10.7	33.24	33.24	27.35	tr|H0VDW9|H0VDW9_CAVPO	Uncharacterized protein OS=Cavia porcellus GN=CTSB PE=3 SV=1	CAVPO	7
1119	10.49	19.2	67.21	60.32	56.28	tr|H0VG97|H0VG97_CAVPO	Uncharacterized protein OS=Cavia porcellus GN=YWHAG PE=3 SV=1	CAVPO	14
1120	10.48	10.7	16.86	10.74	8.056	tr|H0UZQ1|H0UZQ1_CAVPO	Uncharacterized protein OS=Cavia porcellus GN=RBM10 PE=4 SV=1	CAVPO	7
1121	10.48	10.9	14.11	9.809	7.656	tr|H0VGW0|H0VGW0_CAVPO	Uncharacterized protein OS=Cavia porcellus GN=SYMPK PE=4 SV=1	CAVPO	7
1122	10.47	10.5	53.13	53.13	53.13	tr|H0UVI6|H0UVI6_CAVPO	Uncharacterized protein (Fragment) OS=Cavia porcellus GN=PFDN5 PE=3 SV=1	CAVPO	6
1123	10.46	10.6	56.45	56.45	40.86	tr|H0V587|H0V587_CAVPO	Uncharacterized protein OS=Cavia porcellus GN=ARL6 PE=3 SV=1	CAVPO	6
1124	10.44	10.5	28.26	17.94	17.94	tr|H0VEY6|H0VEY6_CAVPO	Uncharacterized protein OS=Cavia porcellus GN=ADRM1 PE=4 SV=1	CAVPO	7
1125	10.43	10.7	37.79	37.79	31.27	tr|H0V3T3|H0V3T3_CAVPO	Uncharacterized protein OS=Cavia porcellus GN=VTA1 PE=4 SV=1	CAVPO	8
1126	10.42	10.5	20.98	20.98	20.98	tr|H0UXN5|H0UXN5_CAVPO	Uncharacterized protein OS=Cavia porcellus GN=ECI1 PE=3 SV=1	CAVPO	6
1127	10.39	10.6	30.97	24.56	23.01	tr|H0W7C8|H0W7C8_CAVPO	Uncharacterized protein (Fragment) OS=Cavia porcellus GN=POLDIP3 PE=4 SV=1	CAVPO	6

1128	10.38	10.6	39.01	27.42	21.51	tr|H0V5X3|H0V5X3_CAVPO	Uncharacterized protein OS=Cavia porcellus GN=COPS3 PE=4 SV=1	CAVPO	8
1129	10.37	10.5	30.67	23.95	21.43	tr|H0VJZ6|H0VJZ6_CAVPO	Uncharacterized protein OS=Cavia porcellus GN=SRSF7 PE=4 SV=1	CAVPO	6
1130	10.36	10.4	27.04	27.04	19.74	tr|H0V052|H0V052_CAVPO	Uncharacterized protein OS=Cavia porcellus GN=NUCKS1 PE=4 SV=1	CAVPO	7
1131	10.33	10.4	41.9	32.38	23.17	tr|H0VQD1|H0VQD1_CAVPO	Uncharacterized protein OS=Cavia porcellus GN=C11orf54 PE=4 SV=1	CAVPO	5
1132	10.33	10.5	26.28	23.72	23.72	tr|H0VXK7|H0VXK7_CAVPO	Uncharacterized protein OS=Cavia porcellus GN=RDH8 PE=3 SV=1	CAVPO	6
1133	10.32	10.4	34.68	31.53	31.53	tr|H0VWY1|H0VWY1_CAVPO	Uncharacterized protein OS=Cavia porcellus GN=GSTA4 PE=4 SV=1	CAVPO	6
1134	10.31	10.4	62.8	62.8	39.61	tr|H0W3N2|H0W3N2_CAVPO	Uncharacterized protein (Fragment) OS=Cavia porcellus GN=LYPLA1 PE=4 SV=1	CAVPO	5
1135	10.3	10.4	69.05	66.67	61.9	tr|H0UTW1|H0UTW1_CAVPO	Uncharacterized protein (Fragment) OS=Cavia porcellus GN=DBI PE=4 SV=1	CAVPO	19
1136	10.3	10.4	51.88	43.75	38.75	tr|H0VLC3|H0VLC3_CAVPO	Uncharacterized protein (Fragment) OS=Cavia porcellus GN=EIF3J PE=4 SV=1	CAVPO	5
1137	10.3	10.5	31.97	27.6	22.13	tr|H0VRC7|H0VRC7_CAVPO	Uncharacterized protein (Fragment) OS=Cavia porcellus GN=TXNDC5 PE=3 SV=1	CAVPO	6
1138	10.29	10.3	37.96	31.39	27.74	tr|H0UYF3|H0UYF3_CAVPO	Uncharacterized protein (Fragment) OS=Cavia porcellus GN=NIT2 PE=4 SV=1	CAVPO	6
1139	10.26	10.3	47.22	36.11	32.22	tr|H0W7I1|H0W7I1_CAVPO	Uncharacterized protein OS=Cavia porcellus GN=APRT PE=3 SV=1	CAVPO	7
1140	10.25	10.5	20.42	17.26	15.16	tr|H0VJD3|H0VJD3_CAVPO	Uncharacterized protein OS=Cavia porcellus GN=ZMPSTE24 PE=4 SV=1	CAVPO	6
1141	10.24	10.3	14.83	12.45	11.05	tr|H0UVR3|H0UVR3_CAVPO	Uncharacterized protein OS=Cavia porcellus GN=BBS7 PE=4 SV=1	CAVPO	6

1142	10.23	10.3	34.83	34.83	28.36	tr|H0VY67|H0VY67_CAVPO	Proteasome subunit beta type OS=Cavia porcellus GN=PSMB2 PE=3 SV=1	CAVPO	7
1143	10.21	10.8	43.89	38.61	29.04	tr|H0VAK6|H0VAK6_CAVPO	Uncharacterized protein (Fragment) OS=Cavia porcellus GN=ECH1 PE=3 SV=1	CAVPO	7
1144	10.21	10.3	65.38	58.79	58.79	tr|H0VUE8|H0VUE8_CAVPO	Uncharacterized protein OS=Cavia porcellus GN=Dnajc5 PE=4 SV=1	CAVPO	6
1145	10.2	10.3	19.31	16.14	12.22	tr|H0UXE2|H0UXE2_CAVPO	Uncharacterized protein OS=Cavia porcellus GN=PIP5K1C PE=4 SV=1	CAVPO	5
1146	10.2	10.3	42.08	34.84	27.6	tr|H0V8M9|H0V8M9_CAVPO	Uncharacterized protein OS=Cavia porcellus GN=SRSF9 PE=4 SV=1	CAVPO	6
1147	10.18	10.6	23.03	20.29	10.95	tr|H0UZQ8|H0UZQ8_CAVPO	Uncharacterized protein OS=Cavia porcellus GN=KIAA1279 PE=4 SV=1	CAVPO	6
1148	10.18	10.3	29.02	28.74	26.44	tr|H0V7G6|H0V7G6_CAVPO	Uncharacterized protein OS=Cavia porcellus GN=NUDCD3 PE=4 SV=1	CAVPO	7
1149	10.17	10.5	30.04	18.98	11.06	tr|H0VJH6|H0VJH6_CAVPO	Uncharacterized protein OS=Cavia porcellus GN=GMPS PE=4 SV=1	CAVPO	7
1150	10.15	10.2	40.75	31.32	31.32	tr|H0VSU2|H0VSU2_CAVPO	Proteasome subunit beta type OS=Cavia porcellus GN=PSMB4 PE=3 SV=1	CAVPO	7
1151	10.13	10.2	25.09	16.96	13.43	tr|H0VHM8|H0VHM8_CAVPO	Uncharacterized protein (Fragment) OS=Cavia porcellus GN=FIP1L1 PE=4 SV=1	CAVPO	5
1152	10.11	10.1	61.9	61.9	61.9	tr|H0W051|H0W051_CAVPO	Uncharacterized protein OS=Cavia porcellus GN=SNCG PE=4 SV=1	CAVPO	6
1153	10.1	13.6	31.96	31.96	22.44	tr|H0W3J6|H0W3J6_CAVPO	Uncharacterized protein (Fragment) OS=Cavia porcellus GN=PTBP1 PE=4 SV=1	CAVPO	10
1154	10.08	10.2	18.55	17	11.75	tr|H0W1A8|H0W1A8_CAVPO	Transporter (Fragment) OS=Cavia porcellus GN=SLC6A9 PE=3 SV=1	CAVPO	6
1155	10.04	10.5	46	46	33.5	tr|H0VRV5|H0VRV5_CAVPO	Uncharacterized protein OS=Cavia porcellus GN=UBE2K PE=3 SV=1	CAVPO	6

1156	10.03	10	48.15	40.74	40.74	tr|H0V3R6|H0V3R6_CAVPO	ATP synthase-coupling factor 6, mitochondrial OS=Cavia porcellus GN=ATP5J PE=3 SV=1	CAVPO	6
1157	10.03	10	49.42	45.35	39.53	tr|H0VRA0|H0VRA0_CAVPO	Uncharacterized protein OS=Cavia porcellus GN=MYL12B PE=4 SV=1	CAVPO	6
1158	10.02	10	27.78	27.78	27.78	tr|H0UU66|H0UU66_CAVPO	Uncharacterized protein OS=Cavia porcellus GN=TRAPPC3 PE=4 SV=1	CAVPO	5
1159	10.02	10	14.86	12.74	12.74	tr|H0VPX4|H0VPX4_CAVPO	Uncharacterized protein OS=Cavia porcellus GN=MAPK9 PE=4 SV=1	CAVPO	5
1160	10.01	10.2	34.57	27.47	24.07	tr|H0UVV6|H0UVV6_CAVPO	Uncharacterized protein OS=Cavia porcellus GN=FAM49B PE=4 SV=1	CAVPO	6
1161	10	16.7	36.56	34.06	30.31	tr|H0V636|H0V636_CAVPO	Uncharacterized protein (Fragment) OS=Cavia porcellus GN=Gnai1 PE=4 SV=1	CAVPO	10
1162	10	10	12.55	12.55	12.55	tr|H0VTV4|H0VTV4_CAVPO	Uncharacterized protein OS=Cavia porcellus GN=Adpgk PE=4 SV=1	CAVPO	5
1163	10	10	44.62	44.62	44.62	tr|H0W528|H0W528_CAVPO	Uncharacterized protein OS=Cavia porcellus GN=PEA15 PE=4 SV=1	CAVPO	5
1164	9.98	10.1	58.06	58.06	46.24	tr|H0UYH3|H0UYH3_CAVPO	Uncharacterized protein OS=Cavia porcellus GN=RBBP9 PE=4 SV=1	CAVPO	5
1165	9.97	10	41.7	36.77	36.77	tr|H0V4H6|H0V4H6_CAVPO	Uncharacterized protein OS=Cavia porcellus GN=TPD52 PE=4 SV=1	CAVPO	5
1166	9.95	10.2	13.66	6.618	4.068	tr|H0VNK0|H0VNK0_CAVPO	Uncharacterized protein (Fragment) OS=Cavia porcellus GN=MADD PE=4 SV=1	CAVPO	5
1167	9.94	12.2	39.62	32.55	28.77	tr|H0VNV2|H0VNV2_CAVPO	Uncharacterized protein OS=Cavia porcellus GN=CLTA PE=4 SV=1	CAVPO	6
1168	9.92	12.1	51.9	51.27	46.84	tr|H0UYG9|H0UYG9_CAVPO	Uncharacterized protein (Fragment) OS=Cavia porcellus GN=RAB10 PE=3 SV=1	CAVPO	9
1169	9.91	10.4	24.34	21.54	14.04	tr|H0VD21|H0VD21_CAVPO	Uncharacterized protein OS=Cavia porcellus GN=SLC1A4 PE=4 SV=1	CAVPO	5

1170	9.91	10	18.49	11.76	8.824	tr|H0VJR1|H0VJR1_CAVPO	Uncharacterized protein OS=Cavia porcellus GN=Nrxn3 PE=4 SV=1	CAVPO	5
1171	9.89	9.95	37.55	28.52	28.52	tr|H0V6K5|H0V6K5_CAVPO	Uncharacterized protein OS=Cavia porcellus GN=SCGN PE=4 SV=1	CAVPO	6
1172	9.89	9.95	17.01	11.13	9.38	tr|H0VGH5|H0VGH5_CAVPO	Uncharacterized protein (Fragment) OS=Cavia porcellus GN=CLPTM1 PE=4 SV=1	CAVPO	5
1173	9.86	9.89	27.19	25.31	22.81	tr|H0VS03|H0VS03_CAVPO	Uncharacterized protein OS=Cavia porcellus GN=AIMP2 PE=4 SV=1	CAVPO	5
1174	9.85	10	25.59	19.43	19.43	tr|H0VTD9|H0VTD9_CAVPO	Uncharacterized protein OS=Cavia porcellus GN=AGK PE=4 SV=1	CAVPO	7
1175	9.82	9.93	55.49	53.05	53.05	tr|H0UTH4|H0UTH4_CAVPO	Uncharacterized protein OS=Cavia porcellus GN=CNRIP1 PE=4 SV=1	CAVPO	8
1176	9.82	9.95	38.22	25.57	21.26	tr|H0WBG7|H0WBG7_CAVPO	ATPase Asna1 OS=Cavia porcellus GN=ASNA1 PE=3 SV=1	CAVPO	6
1177	9.8	9.83	52.81	44.59	44.59	tr|H0VCT3|H0VCT3_CAVPO	Uncharacterized protein OS=Cavia porcellus GN=LYPLA2 PE=4 SV=1	CAVPO	5
1178	9.79	9.94	11.57	8.796	5.705	tr|H0VK90|H0VK90_CAVPO	Uncharacterized protein OS=Cavia porcellus GN=TPP2 PE=4 SV=1	CAVPO	5
1179	9.78	9.97	28.04	20.66	10.15	tr|H0UZ15|H0UZ15_CAVPO	Uncharacterized protein OS=Cavia porcellus GN=PGM3 PE=3 SV=1	CAVPO	5
1180	9.78	9.95	18.14	12.5	7.48	tr|H0VCU4|H0VCU4_CAVPO	Uncharacterized protein (Fragment) OS=Cavia porcellus GN=Rab3gap1 PE=4 SV=1	CAVPO	5
1181	9.78	9.95	44.77	44.77	44.77	tr|H0W703|H0W703_CAVPO	Uncharacterized protein OS=Cavia porcellus GN=NDUFA8 PE=4 SV=1	CAVPO	7
1182	9.77	9.83	15.47	12.91	8.859	tr|H0VAM0|H0VAM0_CAVPO	Uncharacterized protein OS=Cavia porcellus GN=GTPBP1 PE=4 SV=1	CAVPO	5
1183	9.77	9.8	26.33	26.33	26.33	tr|H0VDB7|H0VDB7_CAVPO	Uncharacterized protein OS=Cavia porcellus GN=SCAMP1 PE=4 SV=1	CAVPO	5

1184	9.77	9.8	30.03	24.92	24.92	tr|H0VHC4|H0VHC4_CAVPO	Uncharacterized protein OS=Cavia porcellus GN=CPPED1 PE=4 SV=1	CAVPO	5
1185	9.77	12.1	62.18	60.9	55.77	tr|H0VRA8|H0VRA8_CAVPO	Uncharacterized protein OS=Cavia porcellus GN=CPLX4 PE=4 SV=1	CAVPO	8
1186	9.75	9.85	33.51	33.51	33.51	tr|H0UWI3|H0UWI3_CAVPO	Uncharacterized protein (Fragment) OS=Cavia porcellus GN=GCA PE=4 SV=1	CAVPO	6
1187	9.74	9.9	24.29	13.94	12.05	tr|H0V786|H0V786_CAVPO	Uncharacterized protein (Fragment) OS=Cavia porcellus GN=LPCAT1 PE=4 SV=1	CAVPO	6
1188	9.73	9.85	60.74	60.74	40.74	tr|H0VU69|H0VU69_CAVPO	Uncharacterized protein OS=Cavia porcellus GN=RBP1 PE=3 SV=1	CAVPO	6
1189	9.72	9.81	20.68	12.27	11.13	tr|H0VCY2|H0VCY2_CAVPO	Uncharacterized protein (Fragment) OS=Cavia porcellus GN=MAP7D2 PE=4 SV=1	CAVPO	6
1190	9.71	10	27.99	17.36	10.5	tr|H0VJ90|H0VJ90_CAVPO	Uncharacterized protein (Fragment) OS=Cavia porcellus GN=NASP PE=4 SV=1	CAVPO	6
1191	9.69	9.88	37.38	31.63	22.04	tr|H0V1X9|H0V1X9_CAVPO	Uncharacterized protein OS=Cavia porcellus GN=Prkra PE=4 SV=1	CAVPO	5
1192	9.68	9.7	52.78	52.78	52.78	tr|H0V0E9|H0V0E9_CAVPO	Peptidyl-prolyl cis-trans isomerase OS=Cavia porcellus GN=FKBP1A PE=4 SV=1	CAVPO	6
1193	9.66	9.72	50.55	40.66	34.07	tr|H0WBZ3|H0WBZ3_CAVPO	Uncharacterized protein OS=Cavia porcellus GN=Cnpy2 PE=4 SV=1	CAVPO	5
1194	9.65	10.9	48.02	40.11	40.11	tr|H0VBU7|H0VBU7_CAVPO	Peptidyl-prolyl cis-trans isomerase OS=Cavia porcellus GN=PPIH PE=3 SV=1	CAVPO	6
1195	9.65	9.84	31.52	29.62	26.36	tr|H0VDH3|H0VDH3_CAVPO	Uncharacterized protein OS=Cavia porcellus GN=RAE1 PE=4 SV=1	CAVPO	8
1196	9.62	9.67	40.79	40.79	37.28	tr|H0W4F9|H0W4F9_CAVPO	Uncharacterized protein (Fragment) OS=Cavia porcellus GN=APOA1BP PE=3 SV=1	CAVPO	6
1197	9.61	25.8	36.81	26.73	19.29	tr|H0V0S9|H0V0S9_CAVPO	Uncharacterized protein (Fragment) OS=Cavia porcellus GN=RDX PE=4 SV=1	CAVPO	13

1198	9.6	9.79	18.55	14.18	9.201	tr|H0UVZ9|H0UVZ9_CAVPO	Uncharacterized protein (Fragment) OS=Cavia porcellus GN=SRPK2 PE=3 SV=1	CAVPO	5
1199	9.59	82.7	81.92	79.24	78.35	tr|H0V0E4|H0V0E4_CAVPO	Uncharacterized protein OS=Cavia porcellus GN=TUBB4A PE=3 SV=1	CAVPO	125
1200	9.59	9.62	25.31	25.31	25.31	tr|H0V4U0|H0V4U0_CAVPO	Uncharacterized protein OS=Cavia porcellus GN=Skp1 PE=4 SV=1	CAVPO	5
1201	9.58	30.8	58.39	56.38	48.66	tr|H0V528|H0V528_CAVPO	Uncharacterized protein OS=Cavia porcellus GN=SLC25A4 PE=3 SV=1	CAVPO	27
1202	9.58	9.69	12.25	10.05	8.592	tr|H0VAX6|H0VAX6_CAVPO	Transporter (Fragment) OS=Cavia porcellus GN=SLC6A11 PE=3 SV=1	CAVPO	5
1203	9.57	9.64	17.75	14.2	14.2	tr|H0V5J5|H0V5J5_CAVPO	Uncharacterized protein (Fragment) OS=Cavia porcellus GN=TBL1XR1 PE=4 SV=1	CAVPO	6
1204	9.57	9.72	27.16	18.53	18.53	tr|H0VYG0|H0VYG0_CAVPO	Uncharacterized protein OS=Cavia porcellus GN=HADH PE=4 SV=1	CAVPO	6
1205	9.54	9.73	37.65	37.65	23.48	tr|H0VDT7|H0VDT7_CAVPO	Uncharacterized protein OS=Cavia porcellus GN=Atp6v1d PE=4 SV=1	CAVPO	5
1206	9.53	9.64	29.6	21.97	21.97	tr|H0VZI9|H0VZI9_CAVPO	Uncharacterized protein OS=Cavia porcellus GN=HIST1H1B PE=3 SV=1	CAVPO	6
1207	9.52	9.69	14.97	13.05	11.71	tr|H0VI86|H0VI86_CAVPO	Uncharacterized protein OS=Cavia porcellus GN=SRC PE=4 SV=1	CAVPO	5
1208	9.5	9.87	14.69	10.11	5.453	tr|H0VPE8|H0VPE8_CAVPO	Uncharacterized protein OS=Cavia porcellus GN=IQSEC3 PE=4 SV=1	CAVPO	5
1209	9.49	9.54	67.92	67.92	50.94	tr|H0UUC3|H0UUC3_CAVPO	Uncharacterized protein (Fragment) OS=Cavia porcellus GN=CSTB PE=4 SV=1	CAVPO	5
1210	9.49	9.89	44.63	37.6	34.71	tr|H0UV88|H0UV88_CAVPO	Uncharacterized protein (Fragment) OS=Cavia porcellus GN=GSTO1 PE=3 SV=1	CAVPO	8
1211	9.49	9.57	41.7	39.01	33.63	tr|H0V025|H0V025_CAVPO	Uncharacterized protein (Fragment) OS=Cavia porcellus GN=APIP PE=3 SV=1	CAVPO	6

1212	9.47	9.52	28.94	28.65	26.65	tr|H0W7E8|H0W7E8_CAVPO	Uncharacterized protein OS=Cavia porcellus GN=SCAMP3 PE=4 SV=1	CAVPO	5
1213	9.44	10.4	23.23	18.36	14.38	tr|H0UZ21|H0UZ21_CAVPO	Malic enzyme (Fragment) OS=Cavia porcellus GN=ME1 PE=3 SV=1	CAVPO	7
1214	9.44	9.7	49.31	37.79	23.96	tr|H0V3J8|H0V3J8_CAVPO	Uncharacterized protein OS=Cavia porcellus GN=GRB2 PE=4 SV=1	CAVPO	5
1215	9.44	9.66	7.356	6.437	3.494	tr|H0VP27|H0VP27_CAVPO	Uncharacterized protein (Fragment) OS=Cavia porcellus GN=SEC16A PE=4 SV=1	CAVPO	6
1216	9.43	9.47	35.92	26.53	26.53	tr|H0VND2|H0VND2_CAVPO	Eukaryotic translation initiation factor 6 OS=Cavia porcellus GN=EIF6 PE=1 SV=1	CAVPO	6
1217	9.43	9.47	40.6	40.6	40.6	tr|H0VRQ6|H0VRQ6_CAVPO	Uncharacterized protein (Fragment) OS=Cavia porcellus GN=HRSP12 PE=4 SV=1	CAVPO	7
1218	9.42	9.59	41.97	41.97	31.75	tr|H0UXP3|H0UXP3_CAVPO	Cytochrome b-c1 complex subunit Rieske, mitochondrial OS=Cavia porcellus GN=UQCRFS1 PE=3 SV=1	CAVPO	7
1219	9.41	9.54	36.25	30.94	30.94	tr|H0V777|H0V777_CAVPO	Uncharacterized protein (Fragment) OS=Cavia porcellus GN=ETFA PE=4 SV=1	CAVPO	6
1220	9.4	9.51	30.27	27.89	26.71	tr|H0W0L4|H0W0L4_CAVPO	Uncharacterized protein OS=Cavia porcellus GN=CA14 PE=4 SV=1	CAVPO	7
1221	9.4	9.96	11.57	9.643	7.04	tr|H0W4F8|H0W4F8_CAVPO	Uncharacterized protein OS=Cavia porcellus GN=PITRM1 PE=3 SV=1	CAVPO	5
1222	9.39	9.52	22.73	17.46	17.46	tr|H0V019|H0V019_CAVPO	Uncharacterized protein (Fragment) OS=Cavia porcellus GN=HSDL2 PE=4 SV=1	CAVPO	5
1223	9.38	9.42	36.5	35.5	35.5	tr|H0VFT8|H0VFT8_CAVPO	Uncharacterized protein OS=Cavia porcellus GN=GUCA1B PE=4 SV=1	CAVPO	5
1224	9.36	9.4	33.7	28.89	28.89	tr|H0V7I1|H0V7I1_CAVPO	Uncharacterized protein (Fragment) OS=Cavia porcellus GN=TRA2B PE=4 SV=1	CAVPO	10
1225	9.36	9.4	42.86	42.86	42.86	tr|H0WA07|H0WA07_CAVPO	Thioredoxin OS=Cavia porcellus	CAVPO	5

							GN=LOC100729881 PE=3 SV=1		
1226	9.34	9.57	19.63	12.27	7.362	tr|H0VQL0|H0VQL0_CAVPO	Uncharacterized protein OS=Cavia porcellus GN=GTF3C4 PE=4 SV=1	CAVPO	5
1227	9.33	9.47	32.38	32.38	29.05	tr|H0UW56|H0UW56_CAVPO	Uncharacterized protein OS=Cavia porcellus GN=NDUFS8 PE=3 SV=1	CAVPO	5
1228	9.33	12.6	39.13	32.61	32.61	tr|H0VLL2|H0VLL2_CAVPO	Uncharacterized protein (Fragment) OS=Cavia porcellus GN=RPRD1A PE=4 SV=1	CAVPO	8
1229	9.31	9.48	26.15	26.15	26.15	tr|H0UYZ6|H0UYZ6_CAVPO	Uncharacterized protein OS=Cavia porcellus GN=NDRG2 PE=4 SV=1	CAVPO	6
1230	9.31	9.52	42.76	33.45	26.55	tr|H0VMZ7|H0VMZ7_CAVPO	Uncharacterized protein (Fragment) OS=Cavia porcellus GN=Tsnax PE=4 SV=1	CAVPO	6
1231	9.3	9.41	38.98	34.5	24.28	tr|H0UTR6|H0UTR6_CAVPO	Uncharacterized protein OS=Cavia porcellus GN=Fahd2a PE=4 SV=1	CAVPO	5
1232	9.29	9.42	39.25	27.73	24.92	tr|H0WDY5|H0WDY5_CAVPO	Uncharacterized protein (Fragment) OS=Cavia porcellus PE=4 SV=1	CAVPO	5
1233	9.28	9.4	42.64	39.59	32.49	tr|H0VP81|H0VP81_CAVPO	Uncharacterized protein OS=Cavia porcellus GN=Vbp1 PE=4 SV=1	CAVPO	6
1234	9.26	9.5	24.1	19.08	15.26	tr|H0VGQ4|H0VGQ4_CAVPO	Tubby-like protein (Fragment) OS=Cavia porcellus GN=TUB PE=3 SV=1	CAVPO	6
1235	9.26	9.36	41.57	36.7	27.72	tr|H0W0K4|H0W0K4_CAVPO	Uncharacterized protein (Fragment) OS=Cavia porcellus GN=SPR PE=4 SV=1	CAVPO	5
1236	9.22	9.3	25.56	21.55	17.79	tr|H0UT02|H0UT02_CAVPO	Uncharacterized protein OS=Cavia porcellus GN=BCL2L13 PE=4 SV=1	CAVPO	5
1237	9.21	9.52	27.11	13.8	6.716	tr|H0V0H4|H0V0H4_CAVPO	Uncharacterized protein OS=Cavia porcellus GN=DDX23 PE=3 SV=1	CAVPO	5
1238	9.21	9.29	21.41	19.46	16.55	tr|H0V6B8|H0V6B8_CAVPO	Uncharacterized protein (Fragment) OS=Cavia porcellus GN=WDFY1 PE=4 SV=1	CAVPO	5

1239	9.19	19.6	54.19	50.66	50.66	tr|H0UW38|H0UW38_CAVPO	Uncharacterized protein OS=Cavia porcellus GN=RAB3C PE=3 SV=1	CAVPO	14
1240	9.14	9.28	34.78	33.15	22.55	tr|H0VVH1|H0VVH1_CAVPO	Uncharacterized protein (Fragment) OS=Cavia porcellus GN=DDI2 PE=4 SV=1	CAVPO	7
1241	9.11	9.18	24.25	21.24	10.97	tr|H0VEC3|H0VEC3_CAVPO	Uncharacterized protein OS=Cavia porcellus GN=USP39 PE=4 SV=1	CAVPO	4
1242	9.11	9.26	28.67	24.67	24.67	tr|Q8VD40|Q8VD40_CAVPO	F-box protein OCP1 OS=Cavia porcellus GN=OCP1 PE=2 SV=1	CAVPO	6
1243	9.09	9.31	15.25	10.31	5.451	tr|H0VMR7|H0VMR7_CAVPO	DNA-directed RNA polymerase OS=Cavia porcellus GN=POLR2B PE=3 SV=1	CAVPO	4
1244	9.08	9.14	32.5	21.88	19.06	tr|H0VEG4|H0VEG4_CAVPO	Eukaryotic translation initiation factor 3 subunit G OS=Cavia porcellus GN=EIF3G PE=3 SV=1	CAVPO	5
1245	9.08	9.26	26.62	26.62	15.26	tr|H0VR10|H0VR10_CAVPO	Uncharacterized protein OS=Cavia porcellus GN=TECR PE=4 SV=1	CAVPO	6
1246	9.08	9.31	10.81	5.043	3.218	tr|H0WDG6|H0WDG6_CAVPO	Uncharacterized protein OS=Cavia porcellus GN=GTF3C1 PE=4 SV=1	CAVPO	5
1247	9.07	9.2	51.47	26.71	26.71	tr|H0VTD2|H0VTD2_CAVPO	Uncharacterized protein (Fragment) OS=Cavia porcellus GN=Eif2s2 PE=4 SV=1	CAVPO	7
1248	9.07	9.2	67.65	53.27	46.41	tr|H0W2Y9|H0W2Y9_CAVPO	Uncharacterized protein OS=Cavia porcellus GN=Pabpn1 PE=4 SV=1	CAVPO	6
1249	9.06	9.13	26.11	21.97	21.97	tr|H0V7Z3|H0V7Z3_CAVPO	Autophagy-related protein 3 OS=Cavia porcellus GN=ATG3 PE=3 SV=1	CAVPO	5
1250	9.06	9.44	35.57	24.11	20.95	tr|H0VRI7|H0VRI7_CAVPO	Uncharacterized protein OS=Cavia porcellus GN=DNAJC8 PE=4 SV=1	CAVPO	6
1251	9.05	9.15	60.67	58.43	58.43	tr|H0VA88|H0VA88_CAVPO	Uncharacterized protein OS=Cavia porcellus GN=BANF1 PE=4 SV=1	CAVPO	5
1252	9.04	9.24	22.49	17.09	10.8	tr|H0VMN5|H0VMN5_CAVPO	Uncharacterized protein OS=Cavia porcellus	CAVPO	6

							GN=PLAA PE=4 SV=1		
1253	9.03	11.2	54.78	54.78	47.83	tr|H0W7J9|H0W7J9_CAVPO	Uncharacterized protein OS=Cavia porcellus GN=RPL30 PE=3 SV=1	CAVPO	6
1254	9.02	10.7	32.32	25.8	14.81	tr|H0VH09|H0VH09_CAVPO	Uncharacterized protein OS=Cavia porcellus GN=HTATSF1 PE=4 SV=1	CAVPO	6
1255	9.01	9.1	30.75	27.81	19.79	tr|H0V1U1|H0V1U1_CAVPO	Eukaryotic translation initiation factor 3 subunit M OS=Cavia porcellus GN=EIF3M PE=3 SV=1	CAVPO	5
1256	9.01	9.37	10.01	6.098	2.33	tr|H0V3Q7|H0V3Q7_CAVPO	Uncharacterized protein OS=Cavia porcellus GN=NUP205 PE=4 SV=1	CAVPO	4
1257	9.01	9.23	29.19	19.28	12.97	tr|H0VE13|H0VE13_CAVPO	Uncharacterized protein OS=Cavia porcellus GN=IK PE=4 SV=1	CAVPO	5
1258	9	11.8	23.83	20.47	20.21	tr|H0W3W1|H0W3W1_CAVPO	Uncharacterized protein (Fragment) OS=Cavia porcellus GN=NAP1L1 PE=3 SV=1	CAVPO	7
1259	9	9.1	30.05	24.04	24.04	tr|H0W4U6|H0W4U6_CAVPO	Uncharacterized protein OS=Cavia porcellus GN=UBE2M PE=3 SV=1	CAVPO	5
1260	8.97	9.09	44.37	34.51	34.51	tr|H0W7J7|H0W7J7_CAVPO	Uncharacterized protein (Fragment) OS=Cavia porcellus GN=AP2S1 PE=4 SV=1	CAVPO	6
1261	8.96	9.09	27.96	19.73	15.09	tr|H0V7K9|H0V7K9_CAVPO	Uncharacterized protein OS=Cavia porcellus GN=MTDH PE=4 SV=1	CAVPO	6
1262	8.95	8.99	30.36	30.36	30.36	tr|H0UZP9|H0UZP9_CAVPO	Uncharacterized protein (Fragment) OS=Cavia porcellus GN=UNC119 PE=4 SV=1	CAVPO	6
1263	8.95	9.04	8.207	6.944	5.934	tr|H0VAV9|H0VAV9_CAVPO	Uncharacterized protein (Fragment) OS=Cavia porcellus GN=CD44 PE=4 SV=1	CAVPO	5
1264	8.94	8.97	36.41	36.41	36.41	tr|H0VPI1|H0VPI1_CAVPO	Uncharacterized protein (Fragment) OS=Cavia porcellus GN=Rpl12 PE=3 SV=1	CAVPO	5
1265	8.94	16.2	57.36	57.36	57.36	tr|H0VTH8|H0VTH8_CAVPO	Uncharacterized protein OS=Cavia porcellus GN=LIN7C PE=4 SV=1	CAVPO	10

1266	8.94	9	24.34	16.1	11.99	tr|H0VTZ0|H0VTZ0_CAVPO	Uncharacterized protein OS=Cavia porcellus GN=P4ha1 PE=4 SV=1	CAVPO	5
1267	8.92	8.98	24.53	24.29	12.26	tr|H0VNJ3|H0VNJ3_CAVPO	Uncharacterized protein OS=Cavia porcellus GN=PACSIN3 PE=4 SV=1	CAVPO	4
1268	8.91	9.05	21.12	18.65	13.53	tr|H0V2E7|H0V2E7_CAVPO	Uncharacterized protein OS=Cavia porcellus GN=PNN PE=4 SV=1	CAVPO	6
1269	8.89	9.01	34.1	31.03	25.29	tr|H0VE65|H0VE65_CAVPO	Proliferating cell nuclear antigen OS=Cavia porcellus GN=PCNA PE=3 SV=1	CAVPO	5
1270	8.87	14	34.89	31.65	31.65	tr|H0VD19|H0VD19_CAVPO	Uncharacterized protein (Fragment) OS=Cavia porcellus GN=MSI2 PE=4 SV=1	CAVPO	7
1271	8.87	11.2	18.92	13.01	10.81	tr|H0VP49|H0VP49_CAVPO	Uncharacterized protein (Fragment) OS=Cavia porcellus GN=PGM2 PE=3 SV=1	CAVPO	6
1272	8.87	12.7	20.12	16.56	14.24	tr|H0VWS3|H0VWS3_CAVPO	Uncharacterized protein OS=Cavia porcellus PE=4 SV=1	CAVPO	8
1273	8.86	71.3	84.82	84.82	84.6	tr|H0V3I8|H0V3I8_CAVPO	Uncharacterized protein (Fragment) OS=Cavia porcellus GN=TUBA4A PE=3 SV=1	CAVPO	120
1274	8.84	8.91	14.56	10.32	5.619	tr|H0V9X2|H0V9X2_CAVPO	Uncharacterized protein OS=Cavia porcellus GN=PPP6R3 PE=4 SV=1	CAVPO	4
1275	8.84	8.97	40	40	24.62	tr|H0VQK1|H0VQK1_CAVPO	Uncharacterized protein OS=Cavia porcellus GN=DPM1 PE=4 SV=1	CAVPO	5
1276	8.83	8.91	19.17	14.03	13.04	tr|H0V0Z5|H0V0Z5_CAVPO	Uncharacterized protein OS=Cavia porcellus GN=CAMKV PE=4 SV=1	CAVPO	6
1277	8.83	15.2	43.96	40.1	40.1	tr|H0VP79|H0VP79_CAVPO	Uncharacterized protein OS=Cavia porcellus GN=RAB8A PE=3 SV=1	CAVPO	10
1278	8.83	8.87	41.54	35.38	35.38	tr|H0W074|H0W074_CAVPO	Uncharacterized protein OS=Cavia porcellus GN=RPS15A PE=3 SV=1	CAVPO	5
1279	8.83	9.05	11.83	8.594	6.027	tr|H0W2J8|H0W2J8_CAVPO	Uncharacterized protein OS=Cavia porcellus GN=CHERP PE=4 SV=1	CAVPO	4

1280	8.77	8.8	49.49	49.49	49.49	tr|H0WDN4|H0WDN4_CAVPO	Acylphosphatase OS=Cavia porcellus GN=ACYP1 PE=3 SV=1	CAVPO	6
1281	8.75	8.81	64.6	63.72	63.72	tr|H0VQT8|H0VQT8_CAVPO	Uncharacterized protein OS=Cavia porcellus GN=Eif1 PE=4 SV=1	CAVPO	6
1282	8.75	8.89	13.95	10.68	7.715	tr|H0W7W7|H0W7W7_CAVPO	Uncharacterized protein (Fragment) OS=Cavia porcellus GN=MAP1S PE=4 SV=1	CAVPO	5
1283	8.73	8.85	30.77	27.97	14.22	tr|H0VUP6|H0VUP6_CAVPO	Uncharacterized protein OS=Cavia porcellus GN=ACTL6A PE=3 SV=1	CAVPO	4
1284	8.72	8.79	66.46	56.52	56.52	tr|H0W7Q3|H0W7Q3_CAVPO	Uncharacterized protein OS=Cavia porcellus GN=LAMTOR1 PE=4 SV=1	CAVPO	5
1285	8.71	8.8	55.93	50.85	50.85	tr|H0W335|H0W335_CAVPO	Uncharacterized protein OS=Cavia porcellus GN=C19orf70 PE=4 SV=1	CAVPO	5
1286	8.69	15.9	62.96	55.56	48.15	tr|H0W4H3|H0W4H3_CAVPO	Uncharacterized protein OS=Cavia porcellus GN=RAB2B PE=3 SV=1	CAVPO	10
1287	8.67	9.8	36.12	33.48	33.48	tr|Q9TEG8|Q9TEG8_CAVPO	Cytochrome c oxidase subunit 2 OS=Cavia porcellus GN=COII PE=3 SV=1	CAVPO	9
1288	8.65	8.8	21.7	15.73	12.48	tr|H0V723|H0V723_CAVPO	Uncharacterized protein OS=Cavia porcellus GN=COASY PE=3 SV=1	CAVPO	6
1289	8.64	8.75	23.64	23.64	16.31	tr|H0VD76|H0VD76_CAVPO	Ribosome biogenesis protein WDR12 OS=Cavia porcellus GN=Wdr12 PE=3 SV=1	CAVPO	5
1290	8.61	8.73	33.08	16.92	16.92	tr|H0UWR0|H0UWR0_CAVPO	Uncharacterized protein OS=Cavia porcellus GN=WTAP PE=4 SV=1	CAVPO	5
1291	8.61	8.73	26.63	19.2	19.2	tr|H0V3J1|H0V3J1_CAVPO	Uncharacterized protein OS=Cavia porcellus GN=DNAJB2 PE=4 SV=1	CAVPO	5
1292	8.61	8.86	51.3	51.3	47.4	tr|H0V6W8|H0V6W8_CAVPO	Uncharacterized protein OS=Cavia porcellus GN=UBE2L3 PE=3 SV=1	CAVPO	5
1293	8.61	8.7	28.62	26.26	21.89	tr|H0VJH4|H0VJH4_CAVPO	Uncharacterized protein OS=Cavia porcellus GN=EMC2 PE=4 SV=1	CAVPO	5

1294	8.59	8.69	11.84	8.635	5.71	tr|H0V9Q9|H0V9Q9_CAVPO	Uncharacterized protein (Fragment) OS=Cavia porcellus GN=STT3A PE=4 SV=1	CAVPO	5
1295	8.57	8.74	22.68	17.58	17.58	tr|H0VE80|H0VE80_CAVPO	Uncharacterized protein (Fragment) OS=Cavia porcellus GN=STAM PE=4 SV=1	CAVPO	6
1296	8.57	8.66	44.88	44.39	32.2	tr|H0W8S1|H0W8S1_CAVPO	Proteasome subunit beta type (Fragment) OS=Cavia porcellus GN=PSMB3 PE=3 SV=1	CAVPO	6
1297	8.56	8.6	14.61	9.608	6.766	tr|H0UVZ4|H0UVZ4_CAVPO	Uncharacterized protein OS=Cavia porcellus GN=SURF1 PE=4 SV=1	CAVPO	4
1298	8.56	8.6	46.72	38.52	22.13	tr|H0WCF4|H0WCF4_CAVPO	L-xylulose reductase OS=Cavia porcellus GN=DCXR PE=4 SV=1	CAVPO	4
1299	8.55	8.75	19.56	8.685	4.225	tr|H0UUT0|H0UUT0_CAVPO	Uncharacterized protein (Fragment) OS=Cavia porcellus GN=IARS PE=3 SV=1	CAVPO	4
1300	8.55	9	12.67	6.823	3.606	tr|H0VAA0|H0VAA0_CAVPO	Uncharacterized protein OS=Cavia porcellus GN=MYO18A PE=4 SV=1	CAVPO	5
1301	8.54	8.57	43.05	36.42	29.8	tr|H0W4J5|H0W4J5_CAVPO	Uncharacterized protein OS=Cavia porcellus GN=Rps14 PE=3 SV=1	CAVPO	4
1302	8.54	8.61	30.07	24.13	21.68	tr|H0W591|H0W591_CAVPO	Uncharacterized protein OS=Cavia porcellus GN=CRX PE=3 SV=1	CAVPO	6
1303	8.52	8.56	40.84	34.55	29.32	tr|H0UYK8|H0UYK8_CAVPO	Uncharacterized protein OS=Cavia porcellus GN=HPCAL4 PE=4 SV=1	CAVPO	5
1304	8.52	8.77	10.74	5.479	4.767	tr|H0VBY7|H0VBY7_CAVPO	Uncharacterized protein (Fragment) OS=Cavia porcellus GN=MLLT4 PE=4 SV=1	CAVPO	6
1305	8.51	8.62	50.28	50.28	40.88	tr|H0V5M0|H0V5M0_CAVPO	Malignant T-cell-amplified sequence OS=Cavia porcellus GN=MCTS1 PE=3 SV=1	CAVPO	5
1306	8.49	8.64	25.88	18.36	12.19	tr|H0V9Y4|H0V9Y4_CAVPO	Uncharacterized protein OS=Cavia porcellus GN=ABCE1 PE=3 SV=1	CAVPO	5
1307	8.49	8.66	45.45	39.39	24.24	tr|H0VG44|H0VG44_CAVPO	Uncharacterized protein OS=Cavia porcellus GN=SRI PE=4 SV=1	CAVPO	5

1308	8.47	8.63	36.71	21.84	21.52	tr|H0V379|H0V379_CAVPO	Uncharacterized protein OS=Cavia porcellus GN=PDLIM3 PE=4 SV=1	CAVPO	5
1309	8.47	10.6	41.3	41.3	41.3	tr|H0VBZ1|H0VBZ1_CAVPO	Uncharacterized protein OS=Cavia porcellus GN=RAP1B PE=4 SV=1	CAVPO	7
1310	8.47	8.69	18.74	13.77	4.589	tr|H0VRL3|H0VRL3_CAVPO	Uncharacterized protein OS=Cavia porcellus GN=OGT PE=4 SV=1	CAVPO	4
1311	8.47	8.58	51.22	51.22	35.77	tr|H0VUM8|H0VUM8_CAVPO	Uncharacterized protein (Fragment) OS=Cavia porcellus GN=PHPT1 PE=4 SV=1	CAVPO	5
1312	8.45	8.54	38.59	21.14	19.13	tr|H0V9M0|H0V9M0_CAVPO	Apolipoprotein E OS=Cavia porcellus GN=APOE PE=3 SV=1	CAVPO	5
1313	8.45	8.73	26.89	19.32	17.05	tr|H0VHL0|H0VHL0_CAVPO	Uncharacterized protein (Fragment) OS=Cavia porcellus GN=PRPF4 PE=4 SV=1	CAVPO	7
1314	8.44	8.57	29.66	29.66	29.66	tr|H0V9N7|H0V9N7_CAVPO	Uncharacterized protein OS=Cavia porcellus GN=PON2 PE=4 SV=1	CAVPO	6
1315	8.43	8.46	36.02	36.02	32.7	tr|H0VLX4|H0VLX4_CAVPO	Uncharacterized protein OS=Cavia porcellus GN=NACA2 PE=4 SV=1	CAVPO	5
1316	8.42	8.51	24.03	22.08	17.53	tr|H0VIP9|H0VIP9_CAVPO	Uncharacterized protein (Fragment) OS=Cavia porcellus GN=SDSL PE=4 SV=1	CAVPO	4
1317	8.41	8.62	22.57	15.7	10.76	tr|H0VJ80|H0VJ80_CAVPO	Diacylglycerol kinase OS=Cavia porcellus GN=DGKE PE=3 SV=1	CAVPO	7
1318	8.39	8.5	35.04	30.48	21.94	tr|H0W7N7|H0W7N7_CAVPO	Uncharacterized protein OS=Cavia porcellus GN=NEGR1 PE=4 SV=1	CAVPO	4
1319	8.38	8.92	19.72	15.99	11.19	tr|H0V6I2|H0V6I2_CAVPO	Uncharacterized protein OS=Cavia porcellus GN=Mccc2 PE=4 SV=1	CAVPO	5
1320	8.37	8.5	35.76	31.69	29.36	tr|H0UVT1|H0UVT1_CAVPO	Uncharacterized protein OS=Cavia porcellus GN=PVRL1 PE=4 SV=1	CAVPO	7
1321	8.36	8.52	29.68	25.81	19.03	tr|H0W6W3|H0W6W3_CAVPO	Anamorsin OS=Cavia porcellus GN=CIAPIN1 PE=3 SV=1	CAVPO	6

1322	8.34	9.52	41.04	35.07	35.07	tr|H0V710|H0V710_CAVPO	Uncharacterized protein OS=Cavia porcellus GN=SNCB PE=4 SV=1	CAVPO	13
1323	8.34	8.56	20.14	18.61	9.306	tr|H0VC67|H0VC67_CAVPO	Uncharacterized protein (Fragment) OS=Cavia porcellus GN=SACM1L PE=4 SV=1	CAVPO	5
1324	8.34	8.41	67.12	67.12	48.63	tr|H0VG92|H0VG92_CAVPO	Uncharacterized protein (Fragment) OS=Cavia porcellus GN=NDUFA12 PE=4 SV=1	CAVPO	5
1325	8.33	22.4	52.44	34.34	32.02	tr|H0VDQ3|H0VDQ3_CAVPO	Uncharacterized protein OS=Cavia porcellus GN=SEPT11 PE=3 SV=1	CAVPO	13
1326	8.32	8.49	11.27	9.419	4.665	tr|H0UXH0|H0UXH0_CAVPO	Uncharacterized protein OS=Cavia porcellus GN=MYO1B PE=4 SV=1	CAVPO	5
1327	8.32	8.35	36.52	26.97	23.03	tr|H0V1Z0|H0V1Z0_CAVPO	Uncharacterized protein (Fragment) OS=Cavia porcellus GN=ARPC3 PE=4 SV=1	CAVPO	5
1328	8.32	8.54	11.51	9.637	6.884	tr|H0VDM6|H0VDM6_CAVPO	Integrin beta (Fragment) OS=Cavia porcellus GN=ITGB1 PE=3 SV=1	CAVPO	5
1329	8.32	8.53	14.54	9.346	6.646	tr|H0VYB3|H0VYB3_CAVPO	Uncharacterized protein OS=Cavia porcellus GN=IQSEC1 PE=4 SV=1	CAVPO	5
1330	8.3	8.43	45	27.5	22.86	tr|H0VQ33|H0VQ33_CAVPO	Uncharacterized protein (Fragment) OS=Cavia porcellus GN=GTF2B PE=4 SV=1	CAVPO	5
1331	8.3	8.52	19.71	13.44	10.24	tr|H0VTQ5|H0VTQ5_CAVPO	Uncharacterized protein OS=Cavia porcellus GN=Srrm1 PE=4 SV=1	CAVPO	6
1332	8.29	8.4	21.41	13.58	8.355	tr|H0V2I5|H0V2I5_CAVPO	Uncharacterized protein (Fragment) OS=Cavia porcellus GN=PRKAR1A PE=4 SV=1	CAVPO	4
1333	8.29	8.53	18.32	18.32	8.972	tr|H0VSK1|H0VSK1_CAVPO	Uncharacterized protein OS=Cavia porcellus GN=PDE1B PE=4 SV=1	CAVPO	4
1334	8.27	8.29	56.69	56.69	50.39	tr|H0V125|H0V125_CAVPO	Uncharacterized protein OS=Cavia porcellus GN=NUTF2 PE=4 SV=1	CAVPO	5
1335	8.27	8.6	32.07	32.07	25.54	tr|H0VAS4|H0VAS4_CAVPO	Uncharacterized protein (Fragment) OS=Cavia porcellus GN=CBX5 PE=4 SV=1	CAVPO	6

1336	8.27	8.44	29.24	28.88	28.88	tr|H0VAZ7|H0VAZ7_CAVPO	Proteasome subunit beta type OS=Cavia porcellus GN=PSMB7 PE=3 SV=1	CAVPO	7
1337	8.26	8.28	27.68	27.68	27.68	tr|H0VJ27|H0VJ27_CAVPO	Uncharacterized protein OS=Cavia porcellus GN=Aqp1 PE=3 SV=1	CAVPO	6
1338	8.25	8.49	16.99	6.43	5.662	tr|H0VFF2|H0VFF2_CAVPO	Uncharacterized protein OS=Cavia porcellus GN=SKIV2L2 PE=4 SV=1	CAVPO	5
1339	8.24	8.37	9.894	8.14	4.195	tr|H0VFK8|H0VFK8_CAVPO	Uncharacterized protein (Fragment) OS=Cavia porcellus GN=SPHKAP PE=4 SV=1	CAVPO	4
1340	8.24	8.41	23.79	22.07	17.24	tr|H0VKV2|H0VKV2_CAVPO	Uncharacterized protein OS=Cavia porcellus GN=ERGIC1 PE=4 SV=1	CAVPO	5
1341	8.23	8.29	31.25	31.25	31.25	tr|H0W952|H0W952_CAVPO	Histone H2A OS=Cavia porcellus GN=H2AFZ PE=3 SV=1	CAVPO	7
1342	8.22	83.8	82.66	79.95	79.05	tr|H0VKM7|H0VKM7_CAVPO	Uncharacterized protein OS=Cavia porcellus GN=TUBB PE=3 SV=1	CAVPO	130
1343	8.21	8.41	42.11	22.01	18.42	tr|H0UYD8|H0UYD8_CAVPO	Uncharacterized protein OS=Cavia porcellus GN=AP3M2 PE=4 SV=1	CAVPO	5
1344	8.2	8.31	8.761	4.855	3.571	tr|H0VFV6|H0VFV6_CAVPO	Uncharacterized protein OS=Cavia porcellus GN=LAMB2 PE=4 SV=1	CAVPO	5
1345	8.2	8.43	56.68	56.68	36.9	tr|H0VYF1|H0VYF1_CAVPO	Uncharacterized protein OS=Cavia porcellus GN=Dhfr PE=3 SV=1	CAVPO	6
1346	8.19	8.21	8.809	4.818	4.129	tr|H0VH71|H0VH71_CAVPO	Uncharacterized protein (Fragment) OS=Cavia porcellus GN=RPRD2 PE=4 SV=1	CAVPO	4
1347	8.19	8.21	54.79	43.84	38.36	tr|H0VJC4|H0VJC4_CAVPO	Uncharacterized protein OS=Cavia porcellus GN=MAGOH PE=4 SV=1	CAVPO	4
1348	8.17	8.62	38.91	38.91	29.18	tr|H0UX22|H0UX22_CAVPO	Uncharacterized protein (Fragment) OS=Cavia porcellus GN=ELAVL3 PE=4 SV=1	CAVPO	8
1349	8.17	8.41	31.44	21.26	17.37	tr|H0WA39|H0WA39_CAVPO	Uncharacterized protein OS=Cavia porcellus GN=MAP2K6 PE=4 SV=1	CAVPO	5

1350	8.16	8.26	47.49	36.31	36.31	tr|H0V9U7|H0V9U7_CAVPO	Uncharacterized protein OS=Cavia porcellus GN=NDUFB9 PE=4 SV=1	CAVPO	9
1351	8.13	8.42	39.38	31.34	20.21	tr|H0UW14|H0UW14_CAVPO	Uncharacterized protein OS=Cavia porcellus GN=PACSIN2 PE=4 SV=1	CAVPO	8
1352	8.13	16.6	30.76	20.45	16.52	tr|H0VEW1|H0VEW1_CAVPO	Uncharacterized protein OS=Cavia porcellus GN=PABPC4 PE=4 SV=1	CAVPO	12
1353	8.12	8.19	32.85	25.91	19.71	tr|H0VWE9|H0VWE9_CAVPO	Uncharacterized protein OS=Cavia porcellus GN=CNPY3 PE=4 SV=1	CAVPO	4
1354	8.11	8.12	44.55	38.61	36.63	tr|H0UYB7|H0UYB7_CAVPO	Uncharacterized protein OS=Cavia porcellus GN=Hmgn1 PE=4 SV=1	CAVPO	10
1355	8.11	12.6	32.91	29.14	18.87	tr|H0V0V4|H0V0V4_CAVPO	Uncharacterized protein OS=Cavia porcellus GN=SEPT4 PE=3 SV=1	CAVPO	8
1356	8.1	8.11	15.35	15.35	13.54	tr|H0VEW2|H0VEW2_CAVPO	Uncharacterized protein (Fragment) OS=Cavia porcellus GN=SRSF11 PE=4 SV=1	CAVPO	4
1357	8.1	8.19	26.89	23.28	20.33	tr|H0W341|H0W341_CAVPO	Uncharacterized protein OS=Cavia porcellus GN=RNPS1 PE=4 SV=1	CAVPO	4
1358	8.09	8.2	19.04	13.04	10.34	tr|H0VA19|H0VA19_CAVPO	Reticulon (Fragment) OS=Cavia porcellus GN=RTN1 PE=4 SV=1	CAVPO	5
1359	8.09	8.33	12.96	8.83	6.237	tr|H0VK50|H0VK50_CAVPO	Uncharacterized protein OS=Cavia porcellus GN=CASKIN1 PE=4 SV=1	CAVPO	6
1360	8.07	8.2	11.41	11.41	11.41	tr|H0UUW7|H0UUW7_CAVPO	Uncharacterized protein (Fragment) OS=Cavia porcellus GN=Zfr PE=4 SV=1	CAVPO	5
1361	8.07	8.19	21.8	19.89	13	tr|H0VE88|H0VE88_CAVPO	Uncharacterized protein OS=Cavia porcellus GN=NPEPL1 PE=4 SV=1	CAVPO	4
1362	8.07	8.29	11.94	9.336	5.655	tr|H0VLV8|H0VLV8_CAVPO	Uncharacterized protein OS=Cavia porcellus GN=SLC4A10 PE=4 SV=1	CAVPO	5
1363	8.07	8.27	11.01	9.748	5.241	tr|H0VS78|H0VS78_CAVPO	Uncharacterized protein OS=Cavia porcellus GN=EML4 PE=4 SV=1	CAVPO	5

1364	8.07	8.08	28.96	28.96	23.98	tr|H0VX01|H0VX01_CAVPO	Uncharacterized protein OS=Cavia porcellus GN=RAB28 PE=4 SV=1	CAVPO	4
1365	8.06	8.17	35.24	35.24	18.57	tr|H0V831|H0V831_CAVPO	Uncharacterized protein (Fragment) OS=Cavia porcellus GN=THYN1 PE=4 SV=1	CAVPO	4
1366	8.06	8.06	25.76	18.94	18.94	tr|H0WAP2|H0WAP2_CAVPO	Uncharacterized protein OS=Cavia porcellus GN=COPS7B PE=4 SV=1	CAVPO	4
1367	8.05	8.25	20.91	17.94	12.72	tr|H0UVF4|H0UVF4_CAVPO	Uncharacterized protein OS=Cavia porcellus GN=NT5E PE=3 SV=1	CAVPO	5
1368	8.05	8.13	30.73	30.45	17.6	tr|H0VXH1|H0VXH1_CAVPO	Uncharacterized protein OS=Cavia porcellus GN=TOMM40 PE=4 SV=1	CAVPO	5
1369	8.04	8.14	13.36	10.33	8.54	tr|H0VQI6|H0VQI6_CAVPO	Uncharacterized protein OS=Cavia porcellus GN=NHLRC2 PE=4 SV=1	CAVPO	4
1370	8.03	8.03	37.14	30.71	30.71	tr|H0UU38|H0UU38_CAVPO	Profilin OS=Cavia porcellus GN=PFN2 PE=3 SV=1	CAVPO	7
1371	8.02	8.33	39.68	24.87	24.87	tr|H0UY97|H0UY97_CAVPO	Uncharacterized protein OS=Cavia porcellus GN=NDUFB5 PE=4 SV=1	CAVPO	5
1372	8.01	8.01	56.25	48.21	48.21	tr|H0V0G6|H0V0G6_CAVPO	Uncharacterized protein (Fragment) OS=Cavia porcellus GN=TCEB1 PE=4 SV=1	CAVPO	5
1373	8	10	36.76	32.43	32.43	tr|H0UTV1|H0UTV1_CAVPO	Uncharacterized protein (Fragment) OS=Cavia porcellus GN=RAB35 PE=3 SV=1	CAVPO	8
1374	8	8	38.46	38.46	38.46	tr|H0UUN4|H0UUN4_CAVPO	Uncharacterized protein (Fragment) OS=Cavia porcellus GN=ATP6V1G1 PE=4 SV=1	CAVPO	4
1375	8	8	20.08	20.08	16.32	tr|H0UW91|H0UW91_CAVPO	Proteasome subunit beta type OS=Cavia porcellus GN=Psmb6 PE=3 SV=1	CAVPO	5
1376	8	8	21.13	21.13	21.13	tr|H0V0G9|H0V0G9_CAVPO	Uncharacterized protein OS=Cavia porcellus GN=CDIPT PE=3 SV=1	CAVPO	4
1377	8	8	36.52	23.91	23.91	tr|H0V511|H0V511_CAVPO	Uncharacterized protein (Fragment) OS=Cavia porcellus GN=HDHD1 PE=4 SV=1	CAVPO	4

1378	8	14	47.22	38.89	38.89	tr|H0VIM0|H0VIM0_CAVPO	Uncharacterized protein OS=Cavia porcellus GN=RAB5C PE=3 SV=1	CAVPO	11
1379	8	8	71.43	71.43	71.43	tr|H0VKR7|H0VKR7_CAVPO	Uncharacterized protein OS=Cavia porcellus GN=HSBP1 PE=4 SV=1	CAVPO	5
1380	8	8.01	28.72	19.15	19.15	tr|H0VNF6|H0VNF6_CAVPO	Uncharacterized protein OS=Cavia porcellus GN=ARL6IP5 PE=4 SV=1	CAVPO	4
1381	8	17.2	26.44	25.96	18.27	tr|H0VNV3|H0VNV3_CAVPO	Uncharacterized protein OS=Cavia porcellus GN=UBQLN2 PE=4 SV=1	CAVPO	11
1382	8	8	42.02	34.45	34.45	tr|H0VYC9|H0VYC9_CAVPO	V-type proton ATPase subunit F OS=Cavia porcellus GN=ATP6V1F PE=3 SV=1	CAVPO	4
1383	8	14	42.79	42.33	42.33	tr|H0VYQ5|H0VYQ5_CAVPO	Uncharacterized protein OS=Cavia porcellus GN=RAB5B PE=3 SV=1	CAVPO	11
1384	8	8	16.08	16.08	13.05	tr|H0W3E8|H0W3E8_CAVPO	Uncharacterized protein OS=Cavia porcellus GN=SLC37A4 PE=4 SV=1	CAVPO	5
1385	7.98	8.14	11.95	11.5	8.623	tr|H0V2K6|H0V2K6_CAVPO	Uncharacterized protein OS=Cavia porcellus GN=GGT7 PE=4 SV=1	CAVPO	5
1386	7.97	8.14	44.96	44.19	44.19	tr|H0UZ85|H0UZ85_CAVPO	Uncharacterized protein OS=Cavia porcellus GN=COX5B PE=4 SV=1	CAVPO	5
1387	7.96	8.05	47.06	47.06	47.06	tr|H0UT30|H0UT30_CAVPO	Uncharacterized protein (Fragment) OS=Cavia porcellus GN=PTGES3 PE=4 SV=1	CAVPO	7
1388	7.94	8.04	42.96	42.96	42.22	tr|H0VFE1|H0VFE1_CAVPO	Uncharacterized protein OS=Cavia porcellus GN=CISD2 PE=4 SV=1	CAVPO	5
1389	7.93	8	34.75	32.05	18.53	tr|H0UTW8|H0UTW8_CAVPO	Uncharacterized protein OS=Cavia porcellus GN=BPGM PE=3 SV=1	CAVPO	4
1390	7.92	8.32	14.16	6.952	3.635	tr|H0V5J7|H0V5J7_CAVPO	Uncharacterized protein OS=Cavia porcellus GN=SMARCA2 PE=4 SV=1	CAVPO	5
1391	7.9	7.98	14.69	10.19	10.19	tr|H0UYS3|H0UYS3_CAVPO	Uncharacterized protein (Fragment) OS=Cavia porcellus GN=PAIP1 PE=4 SV=1	CAVPO	4

1392	7.9	10.6	15.08	11.9	6.689	tr|H0VJP1|H0VJP1_CAVPO	Uncharacterized protein OS=Cavia porcellus GN=U2SURP PE=4 SV=1	CAVPO	5
1393	7.88	7.94	46.94	46.94	37.76	tr|H0VSP9|H0VSP9_CAVPO	Uncharacterized protein (Fragment) OS=Cavia porcellus GN=COX6B1 PE=4 SV=1	CAVPO	4
1394	7.88	7.97	34.19	34.19	34.19	tr|H0W111|H0W111_CAVPO	Uncharacterized protein (Fragment) OS=Cavia porcellus PE=4 SV=1	CAVPO	9
1395	7.85	8.07	32.75	16.37	16.37	tr|H0V3U6|H0V3U6_CAVPO	Uncharacterized protein (Fragment) OS=Cavia porcellus GN=ACAA2 PE=3 SV=1	CAVPO	5
1396	7.85	7.98	24.25	21.26	20.66	tr|H0VLG8|H0VLG8_CAVPO	Uncharacterized protein (Fragment) OS=Cavia porcellus GN=NDRG4 PE=4 SV=1	CAVPO	5
1397	7.84	7.9	72.29	57.83	57.83	tr|H0UXF6|H0UXF6_CAVPO	40S ribosomal protein S21 OS=Cavia porcellus GN=RPS21 PE=3 SV=1	CAVPO	4
1398	7.84	14.5	47.1	43.12	43.12	tr|H0V5Y4|H0V5Y4_CAVPO	Uncharacterized protein OS=Cavia porcellus GN=GNPDA2 PE=3 SV=1	CAVPO	8
1399	7.83	7.9	33	26.33	20.33	tr|H0UVF1|H0UVF1_CAVPO	Uncharacterized protein OS=Cavia porcellus GN=ECHDC3 PE=4 SV=1	CAVPO	4
1400	7.83	7.93	44.77	29.07	29.07	tr|H0V3V4|H0V3V4_CAVPO	Uncharacterized protein (Fragment) OS=Cavia porcellus GN=RAB21 PE=4 SV=1	CAVPO	5
1401	7.82	9.23	15.6	12.84	11.21	tr|H0VKR3|H0VKR3_CAVPO	Uncharacterized protein (Fragment) OS=Cavia porcellus GN=CACNA2D1 PE=4 SV=1	CAVPO	8
1402	7.82	7.93	28.7	22.83	11.96	tr|H0VX18|H0VX18_CAVPO	Uncharacterized protein OS=Cavia porcellus GN=DCTN4 PE=4 SV=1	CAVPO	4
1403	7.82	7.87	28.04	26.46	23.28	tr|H0W5E6|H0W5E6_CAVPO	Uncharacterized protein OS=Cavia porcellus GN=PDCD6 PE=4 SV=1	CAVPO	4
1404	7.82	7.89	49.15	48.31	48.31	tr|H0WCU0|H0WCU0_CAVPO	Uncharacterized protein OS=Cavia porcellus GN=ATP6V1G2 PE=4 SV=1	CAVPO	4
1405	7.8	8.44	24.36	20.1	10.4	tr|H0V4T6|H0V4T6_CAVPO	Uncharacterized protein OS=Cavia porcellus GN=GART PE=3 SV=1	CAVPO	7

1406	7.8	7.86	57.3	57.3	57.3	tr|H0VST6|H0VST6_CAVPO	Uncharacterized protein OS=Cavia porcellus GN=DYNLL2 PE=4 SV=1	CAVPO	6
1407	7.78	7.87	34.96	32.52	28.86	tr|H0VIW3|H0VIW3_CAVPO	Uncharacterized protein OS=Cavia porcellus GN=IAH1 PE=4 SV=1	CAVPO	6
1408	7.77	7.89	23.37	23.37	13.03	tr|H0VM03|H0VM03_CAVPO	Uncharacterized protein OS=Cavia porcellus GN=WBP2 PE=4 SV=1	CAVPO	4
1409	7.77	7.95	20.47	10.68	8.902	tr|H0W6T1|H0W6T1_CAVPO	Uncharacterized protein OS=Cavia porcellus GN=Soga3 PE=4 SV=1	CAVPO	5
1410	7.76	7.86	53.6	39.2	39.2	tr|H0VBD9|H0VBD9_CAVPO	Uncharacterized protein OS=Cavia porcellus GN=LOC100716657 PE=4 SV=1	CAVPO	4
1411	7.75	8.09	26.73	26.07	17.82	tr|H0V7G4|H0V7G4_CAVPO	Uncharacterized protein OS=Cavia porcellus GN=MTCH2 PE=3 SV=1	CAVPO	4
1412	7.75	8.68	31.66	16.56	11.11	tr|H0WA52|H0WA52_CAVPO	Uncharacterized protein (Fragment) OS=Cavia porcellus PE=4 SV=1	CAVPO	4
1413	7.74	7.95	50.34	42.95	36.91	tr|H0VID0|H0VID0_CAVPO	Stathmin OS=Cavia porcellus GN=STMN1 PE=3 SV=1	CAVPO	9
1414	7.72	11.9	51.35	50.9	50.9	tr|H0WC22|H0WC22_CAVPO	Uncharacterized protein OS=Cavia porcellus GN=HNRNPA0 PE=4 SV=1	CAVPO	10
1415	7.71	7.81	44.88	44.88	44.88	tr|H0UTB0|H0UTB0_CAVPO	Uncharacterized protein OS=Cavia porcellus GN=PDCD5 PE=4 SV=1	CAVPO	5
1416	7.71	7.88	44.69	44.69	31.84	tr|H0W439|H0W439_CAVPO	Uncharacterized protein (Fragment) OS=Cavia porcellus GN=HIBADH PE=4 SV=1	CAVPO	4
1417	7.7	7.86	19.69	13.18	11.3	tr|H0V5U9|H0V5U9_CAVPO	Uncharacterized protein OS=Cavia porcellus GN=NUDCD1 PE=4 SV=1	CAVPO	5
1418	7.69	7.82	27.7	16.54	10.22	tr|H0VFN5|H0VFN5_CAVPO	Uncharacterized protein (Fragment) OS=Cavia porcellus GN=Bco2 PE=3 SV=1	CAVPO	4
1419	7.69	16.7	69.06	65.75	62.43	tr|H0WBX9|H0WBX9_CAVPO	Uncharacterized protein OS=Cavia porcellus GN=ARF1 PE=3 SV=1	CAVPO	12

1420	7.68	7.91	12.6	4.452	3.163	tr|H0UUI8|H0UUI8_CAVPO	Uncharacterized protein OS=Cavia porcellus GN=ITSN1 PE=4 SV=1	CAVPO	6
1421	7.68	8.04	39.25	32.26	27.42	tr|H0UWG1|H0UWG1_CAVPO	Uncharacterized protein OS=Cavia porcellus GN=DCTN3 PE=4 SV=1	CAVPO	6
1422	7.68	7.75	31.68	31.68	28.71	tr|H0VP56|H0VP56_CAVPO	Uncharacterized protein (Fragment) OS=Cavia porcellus GN=PAFAH1B2 PE=4 SV=1	CAVPO	5
1423	7.68	7.74	18.22	18.22	18.22	tr|H0VTW9|H0VTW9_CAVPO	Uncharacterized protein (Fragment) OS=Cavia porcellus GN=PLP1 PE=4 SV=1	CAVPO	4
1424	7.67	7.73	47.66	47.66	39.84	tr|H0VWD0|H0VWD0_CAVPO	Uncharacterized protein (Fragment) OS=Cavia porcellus GN=NHP2L1 PE=4 SV=1	CAVPO	6
1425	7.66	7.72	26.1	22.89	22.89	tr|H0VY54|H0VY54_CAVPO	Uncharacterized protein (Fragment) OS=Cavia porcellus GN=EIF4H PE=4 SV=1	CAVPO	5
1426	7.65	7.78	25.64	19.35	13.75	tr|H0W1C6|H0W1C6_CAVPO	Uncharacterized protein OS=Cavia porcellus GN=SCCPDH PE=4 SV=1	CAVPO	6
1427	7.63	7.83	27.43	22.15	13.92	tr|H0VTI7|H0VTI7_CAVPO	Methionine aminopeptidase 2 OS=Cavia porcellus GN=METAP2 PE=3 SV=1	CAVPO	4
1428	7.62	9.91	14.41	13.52	9.955	tr|H0UZF0|H0UZF0_CAVPO	Uncharacterized protein (Fragment) OS=Cavia porcellus GN=C2CD2 PE=4 SV=1	CAVPO	5
1429	7.62	7.68	37.5	37.5	37.5	tr|H0VLD8|H0VLD8_CAVPO	Uncharacterized protein OS=Cavia porcellus GN=CHCHD2 PE=4 SV=1	CAVPO	6
1430	7.61	7.91	11.28	5.367	5.367	tr|H0V265|H0V265_CAVPO	Uncharacterized protein (Fragment) OS=Cavia porcellus GN=NUP153 PE=4 SV=1	CAVPO	5
1431	7.6	7.66	24.54	24.54	24.54	tr|H0UWL2|H0UWL2_CAVPO	Uncharacterized protein (Fragment) OS=Cavia porcellus GN=GSTZ1 PE=3 SV=1	CAVPO	4
1432	7.6	32.8	26.38	20.85	20.05	tr|H0UY82|H0UY82_CAVPO	Uncharacterized protein OS=Cavia porcellus GN=DNM2 PE=3 SV=1	CAVPO	17
1433	7.59	7.85	10.74	10.74	2.625	tr|H0V5N6|H0V5N6_CAVPO	Uncharacterized protein OS=Cavia porcellus GN=L1CAM PE=4 SV=1	CAVPO	3

1434	7.59	7.76	20.46	20.46	20.46	tr|H0V7V7|H0V7V7_CAVPO	Uncharacterized protein OS=Cavia porcellus GN=DKK3 PE=4 SV=1	CAVPO	6
1435	7.59	10.1	27.59	18.63	12.5	tr|H0VGX4|H0VGX4_CAVPO	Uncharacterized protein (Fragment) OS=Cavia porcellus GN=BZW1 PE=4 SV=1	CAVPO	6
1436	7.57	7.94	35.76	35.1	30.46	tr|H0V2M1|H0V2M1_CAVPO	Uncharacterized protein (Fragment) OS=Cavia porcellus GN=GMFB PE=4 SV=1	CAVPO	4
1437	7.57	7.69	19.16	19.16	17.69	tr|H0VZ48|H0VZ48_CAVPO	Uncharacterized protein OS=Cavia porcellus GN=SERBP1 PE=4 SV=1	CAVPO	6
1438	7.53	7.72	16.76	11.81	8.791	tr|H0UVL9|H0UVL9_CAVPO	Uncharacterized protein OS=Cavia porcellus GN=GOLGA5 PE=4 SV=1	CAVPO	5
1439	7.52	7.58	35.8	26.85	22.22	tr|H0VT30|H0VT30_CAVPO	Uncharacterized protein (Fragment) OS=Cavia porcellus GN=NIT1 PE=4 SV=1	CAVPO	4
1440	7.52	16.4	47.5	40	31.5	tr|H0VU84|H0VU84_CAVPO	Uncharacterized protein OS=Cavia porcellus GN=MAP2K2 PE=4 SV=1	CAVPO	11
1441	7.5	7.67	39.17	39.17	39.17	tr|H0V5B6|H0V5B6_CAVPO	Uncharacterized protein OS=Cavia porcellus GN=EIF4E PE=3 SV=1	CAVPO	6
1442	7.5	7.71	47.98	42.42	42.42	tr|H0V7K0|H0V7K0_CAVPO	Uncharacterized protein OS=Cavia porcellus GN=Sar1a PE=3 SV=1	CAVPO	6
1443	7.5	7.67	35.96	35.96	35.96	tr|H0VHA1|H0VHA1_CAVPO	Uncharacterized protein OS=Cavia porcellus GN=PCNP PE=4 SV=1	CAVPO	5
1444	7.48	10.2	35.51	24.28	22.1	tr|H0WAN7|H0WAN7_CAVPO	Uncharacterized protein (Fragment) OS=Cavia porcellus GN=PURB PE=4 SV=1	CAVPO	8
1445	7.46	8.15	34.16	27.57	23.87	tr|H0UT76|H0UT76_CAVPO	Uncharacterized protein OS=Cavia porcellus GN=CAPNS1 PE=4 SV=1	CAVPO	6
1446	7.45	7.74	11.63	9.412	7.582	tr|H0VBT0|H0VBT0_CAVPO	Uncharacterized protein (Fragment) OS=Cavia porcellus GN=NSUN2 PE=4 SV=1	CAVPO	6
1447	7.45	7.63	30.3	16.76	11.41	tr|H0VFZ9|H0VFZ9_CAVPO	Uncharacterized protein OS=Cavia porcellus GN=TBC1D24 PE=4 SV=1	CAVPO	4

1448	7.44	7.54	39.36	39.36	24.1	tr|H0UZ10|H0UZ10_CAVPO	Uncharacterized protein OS=Cavia porcellus GN=EMD PE=4 SV=1	CAVPO	4
1449	7.44	9.26	40.37	27.52	18.35	tr|H0VR14|H0VR14_CAVPO	Uncharacterized protein (Fragment) OS=Cavia porcellus GN=VPS26A PE=4 SV=1	CAVPO	5
1450	7.43	7.63	52.76	47.24	47.24	tr|H0UTW5|H0UTW5_CAVPO	Uncharacterized protein OS=Cavia porcellus GN=HINT2 PE=4 SV=1	CAVPO	5
1451	7.42	9.72	45.25	29.61	27.37	tr|H0UUA3|H0UUA3_CAVPO	Uncharacterized protein OS=Cavia porcellus GN=HMGB3 PE=4 SV=1	CAVPO	6
1452	7.42	9.75	13.84	13.65	13.65	tr|H0VB23|H0VB23_CAVPO	Uncharacterized protein OS=Cavia porcellus GN=CELF1 PE=4 SV=1	CAVPO	6
1453	7.42	7.65	20.97	10.56	10.56	tr|H0VCA8|H0VCA8_CAVPO	Uncharacterized protein OS=Cavia porcellus GN=TM9SF2 PE=4 SV=1	CAVPO	5
1454	7.42	7.76	29.13	29.13	11.76	tr|H0VUT1|H0VUT1_CAVPO	Uncharacterized protein OS=Cavia porcellus GN=Sord PE=3 SV=1	CAVPO	3
1455	7.41	7.67	20.62	17.16	8.492	tr|H0UW47|H0UW47_CAVPO	Uncharacterized protein (Fragment) OS=Cavia porcellus GN=SCAI PE=4 SV=1	CAVPO	4
1456	7.41	7.49	12	5.767	5.767	tr|H0VRI2|H0VRI2_CAVPO	Uncharacterized protein (Fragment) OS=Cavia porcellus GN=GRAMD1B PE=4 SV=1	CAVPO	4
1457	7.39	7.58	33.89	33.89	25	tr|H0V7I2|H0V7I2_CAVPO	Uncharacterized protein OS=Cavia porcellus GN=SEH1L PE=4 SV=1	CAVPO	5
1458	7.39	7.56	24.76	16.04	16.04	tr|H0VBY6|H0VBY6_CAVPO	Uncharacterized protein OS=Cavia porcellus GN=SF3B4 PE=4 SV=1	CAVPO	5
1459	7.39	7.55	38.89	37.96	37.96	tr|H0W9E6|H0W9E6_CAVPO	Uncharacterized protein OS=Cavia porcellus GN=HMGA1 PE=4 SV=1	CAVPO	6
1460	7.38	10.9	29.8	26.65	17.77	tr|H0VCD1|H0VCD1_CAVPO	Uncharacterized protein (Fragment) OS=Cavia porcellus GN=CSNK2A2 PE=4 SV=1	CAVPO	7
1461	7.36	7.41	28.39	28.39	28.39	tr|H0VGF6|H0VGF6_CAVPO	Uncharacterized protein OS=Cavia porcellus GN=CD81 PE=4 SV=1	CAVPO	6

1462	7.36	7.52	56.36	48.18	48.18	tr|H0VKV6|H0VKV6_CAVPO	Uncharacterized protein (Fragment) OS=Cavia porcellus GN=TBCA PE=4 SV=1	CAVPO	5
1463	7.34	7.49	12.17	10.48	3.858	tr|H0V3X6|H0V3X6_CAVPO	Uncharacterized protein OS=Cavia porcellus GN=PRPF4B PE=4 SV=1	CAVPO	3
1464	7.34	7.53	47.62	42.86	42.86	tr|H0WCE5|H0WCE5_CAVPO	Uncharacterized protein OS=Cavia porcellus GN=ATP5D PE=3 SV=1	CAVPO	8
1465	7.33	7.48	11.54	8.115	6.444	tr|H0VLU1|H0VLU1_CAVPO	Uncharacterized protein (Fragment) OS=Cavia porcellus GN=ZC3H4 PE=4 SV=1	CAVPO	4
1466	7.3	7.39	11.89	8.223	6.902	tr|H0V3A8|H0V3A8_CAVPO	Uncharacterized protein OS=Cavia porcellus GN=NOLC1 PE=4 SV=1	CAVPO	4
1467	7.3	7.49	10.5	8.255	5.778	tr|H0VLK1|H0VLK1_CAVPO	Uncharacterized protein (Fragment) OS=Cavia porcellus GN=TMTC3 PE=4 SV=1	CAVPO	4
1468	7.3	7.76	9.034	5.1	2.574	tr|H0W5W2|H0W5W2_CAVPO	Uncharacterized protein (Fragment) OS=Cavia porcellus GN=PI4KA PE=4 SV=1	CAVPO	4
1469	7.29	7.4	34.23	27.31	18.85	tr|H0UVD4|H0UVD4_CAVPO	Uncharacterized protein (Fragment) OS=Cavia porcellus GN=AUH PE=3 SV=1	CAVPO	5
1470	7.28	7.34	20.49	15.03	15.03	tr|H0UZX7|H0UZX7_CAVPO	Uncharacterized protein (Fragment) OS=Cavia porcellus GN=ATAD1 PE=3 SV=1	CAVPO	4
1471	7.28	7.58	34.36	31.84	24.3	tr|H0V772|H0V772_CAVPO	Uncharacterized protein (Fragment) OS=Cavia porcellus GN=SMS PE=3 SV=1	CAVPO	6
1472	7.28	7.41	34.4	24	24	tr|H0VNE6|H0VNE6_CAVPO	Uncharacterized protein (Fragment) OS=Cavia porcellus GN=Rps25 PE=4 SV=1	CAVPO	4
1473	7.28	10.7	16.06	10.24	3.455	tr|H0VNU5|H0VNU5_CAVPO	Uncharacterized protein (Fragment) OS=Cavia porcellus GN=EIF4G3 PE=4 SV=1	CAVPO	5
1474	7.28	7.35	44.34	38.68	38.68	tr|H0VUH9|H0VUH9_CAVPO	Uncharacterized protein OS=Cavia porcellus GN=GLRX PE=4 SV=1	CAVPO	5
1475	7.28	7.44	46.43	38.69	30.95	tr|H0WDA3|H0WDA3_CAVPO	Uncharacterized protein (Fragment) OS=Cavia porcellus GN=ARPC4 PE=4 SV=1	CAVPO	4

1476	7.25	7.46	15.01	13.79	11.76	tr|H0V4N5|H0V4N5_CAVPO	Uncharacterized protein (Fragment) OS=Cavia porcellus GN=BBS4 PE=4 SV=1	CAVPO	5
1477	7.23	10.2	48.78	48.17	35.98	tr|H0V1J4|H0V1J4_CAVPO	Uncharacterized protein OS=Cavia porcellus GN=SRSF3 PE=4 SV=1	CAVPO	11
1478	7.22	7.36	34.76	30	30	tr|H0UWN2|H0UWN2_CAVPO	Uncharacterized protein OS=Cavia porcellus GN=COPS8 PE=4 SV=1	CAVPO	4
1479	7.22	7.28	19.65	18.16	14.68	tr|H0VYB4|H0VYB4_CAVPO	Uncharacterized protein OS=Cavia porcellus GN=DAZAP1 PE=4 SV=1	CAVPO	4
1480	7.21	7.38	30.94	25.9	14.03	tr|H0VTC7|H0VTC7_CAVPO	Uncharacterized protein OS=Cavia porcellus GN=TMX1 PE=4 SV=1	CAVPO	3
1481	7.2	7.39	35.33	19.94	7.692	tr|H0WDT4|H0WDT4_CAVPO	Uncharacterized protein (Fragment) OS=Cavia porcellus GN=PPP1R8 PE=4 SV=1	CAVPO	3
1482	7.19	7.38	9.923	7.186	4.705	tr|H0V898|H0V898_CAVPO	Uncharacterized protein (Fragment) OS=Cavia porcellus GN=ANKFY1 PE=4 SV=1	CAVPO	4
1483	7.19	7.39	65.67	51.49	47.76	tr|H0VX49|H0VX49_CAVPO	Uncharacterized protein OS=Cavia porcellus GN=CPLX2 PE=4 SV=1	CAVPO	4
1484	7.19	7.36	27.17	27.17	23.37	tr|H0W9Z7|H0W9Z7_CAVPO	Uncharacterized protein OS=Cavia porcellus GN=Arl2 PE=3 SV=1	CAVPO	4
1485	7.18	7.29	23.85	20.77	20.77	tr|H0V0A3|H0V0A3_CAVPO	Uncharacterized protein OS=Cavia porcellus GN=PNPO PE=3 SV=1	CAVPO	5
1486	7.18	7.37	14.31	14.31	12.72	tr|H0VIX4|H0VIX4_CAVPO	Uncharacterized protein OS=Cavia porcellus GN=Slc38a3 PE=4 SV=1	CAVPO	5
1487	7.17	9.4	20.8	20.8	12.39	tr|H0UXH8|H0UXH8_CAVPO	Mitochondrial import inner membrane translocase subunit TIM44 OS=Cavia porcellus GN=TIMM44 PE=3 SV=1	CAVPO	5
1488	7.16	7.36	8.895	5.634	2.743	tr|H0UZ91|H0UZ91_CAVPO	Uncharacterized protein OS=Cavia porcellus GN=TCOF1 PE=4 SV=1	CAVPO	4
1489	7.13	7.22	23.54	16.54	9.922	tr|H0V9N8|H0V9N8_CAVPO	Uncharacterized protein (Fragment) OS=Cavia	CAVPO	4

							porcellus GN=PMPCA PE=3 SV=1		
1490	7.12	7.17	47.41	47.41	47.41	tr|H0UY37|H0UY37_CAVPO	Uncharacterized protein OS=Cavia porcellus GN=NDUFA5 PE=4 SV=1	CAVPO	4
1491	7.12	7.72	48	41.14	32	tr|H0V5H7|H0V5H7_CAVPO	Uncharacterized protein OS=Cavia porcellus GN=LOC100727312 PE=4 SV=1	CAVPO	5
1492	7.1	9.31	31.43	25.97	16.88	tr|H0V3L6|H0V3L6_CAVPO	Uncharacterized protein OS=Cavia porcellus GN=PRPSAP1 PE=4 SV=1	CAVPO	6
1493	7.1	7.28	24.63	22.06	22.06	tr|H0VPX7|H0VPX7_CAVPO	ATP-dependent Clp protease proteolytic subunit OS=Cavia porcellus GN=CLPP PE=3 SV=1	CAVPO	4
1494	7.1	8.12	37.64	21.71	18.24	tr|H0VQQ9|H0VQQ9_CAVPO	Uncharacterized protein OS=Cavia porcellus GN=GSK3B PE=4 SV=1	CAVPO	5
1495	7.09	7.19	11.96	9.129	7.45	tr|H0VC02|H0VC02_CAVPO	Oxysterol-binding protein OS=Cavia porcellus GN=OSBPL1A PE=3 SV=1	CAVPO	5
1496	7.09	7.24	16.63	14.12	12.07	tr|H0VJC5|H0VJC5_CAVPO	Uncharacterized protein OS=Cavia porcellus GN=ANKMY2 PE=4 SV=1	CAVPO	5
1497	7.06	7.22	40.57	36	26.29	tr|A9LPA2|A9LPA2_CAVPO	AlphaB-crystallin OS=Cavia porcellus GN=Cryab PE=2 SV=1	CAVPO	4
1498	7.06	7.19	10.37	6.146	6.146	tr|H0UX04|H0UX04_CAVPO	Uncharacterized protein OS=Cavia porcellus GN=VAC14 PE=4 SV=1	CAVPO	4
1499	7.06	7.35	32.23	27.01	19.43	tr|H0W636|H0W636_CAVPO	60S ribosomal protein L13 OS=Cavia porcellus GN=RPL13 PE=3 SV=1	CAVPO	4
1500	7.04	7.13	16.18	14.97	11.23	tr|H0V3H4|H0V3H4_CAVPO	Uncharacterized protein OS=Cavia porcellus GN=PPM1E PE=3 SV=1	CAVPO	5
1501	7.03	7.09	13.17	7.302	7.302	tr|H0VT93|H0VT93_CAVPO	Uncharacterized protein OS=Cavia porcellus GN=GATAD2A PE=4 SV=1	CAVPO	4
1502	7.02	7.12	16.78	16.78	12.36	tr|H0V2I6|H0V2I6_CAVPO	Uncharacterized protein OS=Cavia porcellus GN=GORASP2 PE=4 SV=1	CAVPO	4

1503	7.02	7.12	37.44	37.44	37.44	tr|H0VFF0|H0VFF0_CAVPO	Uncharacterized protein OS=Cavia porcellus GN=RPS7 PE=4 SV=1	CAVPO	4
1504	7.01	7.23	20.29	8.592	6.563	tr|H0UXT4|H0UXT4_CAVPO	Uncharacterized protein (Fragment) OS=Cavia porcellus GN=NCAM2 PE=4 SV=1	CAVPO	4
1505	6.99	7.17	35.83	28.75	17.92	tr|H0V6N0|H0V6N0_CAVPO	Uncharacterized protein (Fragment) OS=Cavia porcellus GN=TMED4 PE=3 SV=1	CAVPO	5
1506	6.99	7.11	17.27	10.55	10.55	tr|H0VIH9|H0VIH9_CAVPO	Uncharacterized protein (Fragment) OS=Cavia porcellus GN=ACADM PE=3 SV=1	CAVPO	4
1507	6.98	8.94	11.64	10.57	6.654	tr|H0VNM7|H0VNM7_CAVPO	Uncharacterized protein (Fragment) OS=Cavia porcellus GN=LNPEP PE=4 SV=1	CAVPO	6
1508	6.97	7.06	26.65	21.56	18.56	tr|H0UXL3|H0UXL3_CAVPO	Uncharacterized protein OS=Cavia porcellus GN=MAT2B PE=4 SV=1	CAVPO	4
1509	6.97	7.02	19.9	16.5	16.5	tr|H0V2G1|H0V2G1_CAVPO	Uncharacterized protein OS=Cavia porcellus GN=RALB PE=4 SV=1	CAVPO	4
1510	6.96	7.04	12.64	9.195	6.404	tr|H0VD16|H0VD16_CAVPO	Uncharacterized protein OS=Cavia porcellus GN=EPN1 PE=4 SV=1	CAVPO	4
1511	6.95	7.28	24.42	19.19	14.39	tr|H0V2P8|H0V2P8_CAVPO	Uncharacterized protein (Fragment) OS=Cavia porcellus GN=MAP7 PE=4 SV=1	CAVPO	5
1512	6.94	7.2	21.49	19.63	11.94	tr|H0VCN1|H0VCN1_CAVPO	Putative GTP cyclohydrolase 1 type 2 OS=Cavia porcellus GN=NIF3L1 PE=3 SV=1	CAVPO	4
1513	6.94	7.01	14.5	4.22	4.22	tr|H0VG91|H0VG91_CAVPO	Uncharacterized protein (Fragment) OS=Cavia porcellus GN=SMARCC1 PE=4 SV=1	CAVPO	4
1514	6.93	7.07	32.06	31.3	30.53	tr|H0W4R6|H0W4R6_CAVPO	Uncharacterized protein OS=Cavia porcellus GN=PFDN6 PE=4 SV=1	CAVPO	4
1515	6.92	7.1	65.22	65.22	48.91	tr|H0V8K0|H0V8K0_CAVPO	Cytochrome b-c1 complex subunit 6 OS=Cavia porcellus GN=UQCRH PE=3 SV=1	CAVPO	4
1516	6.91	7.03	24.86	20.62	8.671	tr|H0VNJ0|H0VNJ0_CAVPO	Uncharacterized protein OS=Cavia porcellus GN=ARFGAP2 PE=4 SV=1	CAVPO	4

1517	6.91	7.07	21.83	13.71	11.42	tr|H0W1Y4|H0W1Y4_CAVPO	Uncharacterized protein OS=Cavia porcellus GN=PHAX PE=4 SV=1	CAVPO	4
1518	6.9	7	14.95	14.95	14.95	tr|H0V3D4|H0V3D4_CAVPO	Uncharacterized protein OS=Cavia porcellus GN=Rpl9 PE=4 SV=1	CAVPO	4
1519	6.9	6.98	21.07	18.54	18.54	tr|H0VW11|H0VW11_CAVPO	Uncharacterized protein (Fragment) OS=Cavia porcellus GN=TIMM50 PE=4 SV=1	CAVPO	5
1520	6.89	7.05	26.84	18.88	16.81	tr|H0VHQ2|H0VHQ2_CAVPO	Uncharacterized protein OS=Cavia porcellus GN=Igbp1 PE=4 SV=1	CAVPO	4
1521	6.86	6.99	46.27	46.27	35.82	tr|H0VG61|H0VG61_CAVPO	Uncharacterized protein OS=Cavia porcellus GN=GNG13 PE=4 SV=1	CAVPO	4
1522	6.86	7.03	26.93	21.87	18.4	tr|H0VX42|H0VX42_CAVPO	Uncharacterized protein OS=Cavia porcellus GN=C12orf10 PE=4 SV=1	CAVPO	6
1523	6.85	11.6	22.35	16.3	11.18	tr|H0VGV5|H0VGV5_CAVPO	Uncharacterized protein (Fragment) OS=Cavia porcellus GN=AGO2 PE=3 SV=1	CAVPO	8
1524	6.85	17.5	52.33	44.56	44.56	tr|H0W798|H0W798_CAVPO	Uncharacterized protein OS=Cavia porcellus GN=HPCAL1 PE=4 SV=1	CAVPO	9
1525	6.83	7.14	43.43	35.35	28.79	tr|H0V8T3|H0V8T3_CAVPO	Uncharacterized protein OS=Cavia porcellus GN=YKT6 PE=4 SV=1	CAVPO	5
1526	6.82	6.94	49.18	49.18	29.51	tr|H0WDZ7|H0WDZ7_CAVPO	Uncharacterized protein OS=Cavia porcellus GN=PFDN1 PE=4 SV=1	CAVPO	4
1527	6.81	6.9	12.79	9.39	4.46	tr|H0V6P7|H0V6P7_CAVPO	Uncharacterized protein (Fragment) OS=Cavia porcellus GN=UBE3A PE=4 SV=1	CAVPO	5
1528	6.81	6.9	19.3	13.09	5.369	tr|H0VRR0|H0VRR0_CAVPO	Uncharacterized protein OS=Cavia porcellus GN=PDLIM5 PE=4 SV=1	CAVPO	3
1529	6.81	6.87	36.18	36.18	35.68	tr|H0WAL4|H0WAL4_CAVPO	Uncharacterized protein OS=Cavia porcellus GN=MRPL12 PE=3 SV=1	CAVPO	4
1530	6.8	7.04	33.97	33.97	28.21	tr|H0VKJ3|H0VKJ3_CAVPO	Acyl carrier protein OS=Cavia porcellus GN=NDUFAB1 PE=3 SV=1	CAVPO	6

1531	6.8	6.98	15.54	13.99	11.14	tr|H0VSR5|H0VSR5_CAVPO	Uncharacterized protein OS=Cavia porcellus GN=SH3GLB1 PE=4 SV=1	CAVPO	4
1532	6.78	6.92	24.42	14.97	6.061	tr|H0VFI5|H0VFI5_CAVPO	Asparagine synthetase OS=Cavia porcellus GN=ASNS PE=4 SV=1	CAVPO	3
1533	6.78	6.91	29.06	29.06	25.66	tr|H0WAM6|H0WAM6_CAVPO	Uncharacterized protein OS=Cavia porcellus GN=PQBP1 PE=4 SV=1	CAVPO	4
1534	6.77	6.99	28.81	21.31	11.24	tr|H0V6G3|H0V6G3_CAVPO	Uncharacterized protein (Fragment) OS=Cavia porcellus GN=C6orf211 PE=4 SV=1	CAVPO	4
1535	6.77	7.08	10.49	6.936	3.633	tr|H0VE42|H0VE42_CAVPO	Uncharacterized protein (Fragment) OS=Cavia porcellus GN=SEC24B PE=4 SV=1	CAVPO	3
1536	6.76	6.86	26.19	16.96	16.96	tr|H0UVC9|H0UVC9_CAVPO	Uncharacterized protein OS=Cavia porcellus GN=CSNK1A1 PE=3 SV=1	CAVPO	4
1537	6.76	6.92	45.85	39.13	23.32	tr|H0V3S2|H0V3S2_CAVPO	Uncharacterized protein OS=Cavia porcellus GN=NFU1 PE=4 SV=1	CAVPO	4
1538	6.76	7.01	24.72	24.72	14.72	tr|H0W3V6|H0W3V6_CAVPO	Uncharacterized protein OS=Cavia porcellus GN=GMPPB PE=4 SV=1	CAVPO	4
1539	6.74	6.88	15.69	12.85	10.24	tr|H0VR15|H0VR15_CAVPO	Uncharacterized protein OS=Cavia porcellus GN=ZNF259 PE=4 SV=1	CAVPO	4
1540	6.71	7.03	17.98	16.53	10.11	tr|H0UV05|H0UV05_CAVPO	Uncharacterized protein OS=Cavia porcellus GN=CLINT1 PE=4 SV=1	CAVPO	5
1541	6.71	6.78	12.1	6.912	4.608	tr|H0VHK7|H0VHK7_CAVPO	Uncharacterized protein (Fragment) OS=Cavia porcellus GN=KDM1A PE=4 SV=1	CAVPO	3
1542	6.71	6.85	13.49	9.797	7.763	tr|H0VKE6|H0VKE6_CAVPO	Uncharacterized protein (Fragment) OS=Cavia porcellus GN=KPNA6 PE=4 SV=1	CAVPO	4
1543	6.7	13.8	13.47	8.588	6.831	tr|H0VJD9|H0VJD9_CAVPO	Uncharacterized protein OS=Cavia porcellus GN=CLASP1 PE=4 SV=1	CAVPO	8
1544	6.69	7.08	12.04	3.827	2.923	tr|H0VDU9|H0VDU9_CAVPO	Uncharacterized protein OS=Cavia porcellus GN=CLIP1 PE=4 SV=1	CAVPO	4

1545	6.69	6.84	27.02	27.02	19.57	tr|H0VIF0|H0VIF0_CAVPO	Uncharacterized protein OS=Cavia porcellus GN=PSMD7 PE=4 SV=1	CAVPO	4
1546	6.68	6.82	30.32	30.32	26.6	tr|H0UUK1|H0UUK1_CAVPO	40S ribosomal protein S8 (Fragment) OS=Cavia porcellus GN=RPS8 PE=3 SV=1	CAVPO	4
1547	6.68	6.89	18.31	9.606	3.202	tr|H0V0X2|H0V0X2_CAVPO	Uncharacterized protein OS=Cavia porcellus GN=PPFIA2 PE=4 SV=1	CAVPO	3
1548	6.64	6.83	11.21	9.032	4.788	tr|H0V1T6|H0V1T6_CAVPO	Uncharacterized protein (Fragment) OS=Cavia porcellus GN=GTF3C2 PE=4 SV=1	CAVPO	3
1549	6.64	6.8	16.72	8.065	8.065	tr|H0V949|H0V949_CAVPO	Uncharacterized protein OS=Cavia porcellus GN=HBS1L PE=4 SV=1	CAVPO	4
1550	6.64	6.82	19.78	19.33	9.888	tr|H0W6L0|H0W6L0_CAVPO	Phosphatidate cytidylyltransferase OS=Cavia porcellus GN=CDS2 PE=3 SV=1	CAVPO	3
1551	6.62	6.8	26.55	20.8	20.8	tr|H0UYQ9|H0UYQ9_CAVPO	Uncharacterized protein OS=Cavia porcellus GN=LOC100727246 PE=4 SV=1	CAVPO	4
1552	6.62	6.78	28.98	23.25	10.51	tr|H0VK07|H0VK07_CAVPO	Uncharacterized protein OS=Cavia porcellus GN=GDPD1 PE=4 SV=1	CAVPO	3
1553	6.6	6.74	10.53	8.062	4.161	tr|H0VKE8|H0VKE8_CAVPO	Uncharacterized protein (Fragment) OS=Cavia porcellus GN=ABLIM1 PE=4 SV=1	CAVPO	3
1554	6.6	6.75	51.56	31.11	19.11	tr|H0VX24|H0VX24_CAVPO	Uncharacterized protein (Fragment) OS=Cavia porcellus GN=AK4 PE=3 SV=1	CAVPO	4
1555	6.59	7.35	6.158	1.795	0.7143	tr|H0VBF5|H0VBF5_CAVPO	Uncharacterized protein OS=Cavia porcellus GN=UBR4 PE=4 SV=1	CAVPO	3
1556	6.59	6.63	61.97	60.56	50.7	tr|H0VCW1|H0VCW1_CAVPO	Uncharacterized protein OS=Cavia porcellus GN=Atp5i PE=4 SV=1	CAVPO	3
1557	6.59	6.89	27.59	24.56	16.71	tr|H0VHQ4|H0VHQ4_CAVPO	Uncharacterized protein OS=Cavia porcellus GN=CADM3 PE=4 SV=1	CAVPO	3
1558	6.57	6.87	13.06	8.36	5.494	tr|H0VSB1|H0VSB1_CAVPO	Uncharacterized protein (Fragment) OS=Cavia porcellus GN=CHL1 PE=4 SV=1	CAVPO	6

1559	6.55	6.88	19.26	10.96	5.738	tr|H0VMI5|H0VMI5_CAVPO	Uncharacterized protein OS=Cavia porcellus GN=RNF20 PE=4 SV=1	CAVPO	4
1560	6.53	6.63	35.47	31.98	31.98	tr|H0VKM6|H0VKM6_CAVPO	Uncharacterized protein (Fragment) OS=Cavia porcellus GN=Cetn2 PE=4 SV=1	CAVPO	4
1561	6.53	6.64	57.63	57.63	50	tr|H0VN10|H0VN10_CAVPO	Uncharacterized protein OS=Cavia porcellus GN=MTPN PE=4 SV=1	CAVPO	6
1562	6.53	6.63	20.97	20.97	20.97	tr|H0W9P3|H0W9P3_CAVPO	Uncharacterized protein (Fragment) OS=Cavia porcellus GN=BRI3BP PE=4 SV=1	CAVPO	4
1563	6.52	6.72	21.69	11.42	11.42	tr|H0VYK0|H0VYK0_CAVPO	Uncharacterized protein (Fragment) OS=Cavia porcellus GN=ESRRB PE=3 SV=1	CAVPO	4
1564	6.52	6.63	15.27	8.545	8.545	tr|H0WBM1|H0WBM1_CAVPO	Uncharacterized protein (Fragment) OS=Cavia porcellus GN=CPNE5 PE=4 SV=1	CAVPO	4
1565	6.51	6.61	72	72	72	tr|H0W5B9|H0W5B9_CAVPO	Guanine nucleotide-binding protein subunit gamma OS=Cavia porcellus GN=GNG3 PE=3 SV=1	CAVPO	4
1566	6.5	6.6	37.78	37.78	37.78	tr|H0V7E4|H0V7E4_CAVPO	Galectin (Fragment) OS=Cavia porcellus GN=LGALS1 PE=4 SV=1	CAVPO	5
1567	6.5	6.61	22.37	17.29	17.29	tr|H0VBM8|H0VBM8_CAVPO	Uncharacterized protein (Fragment) OS=Cavia porcellus GN=BLVRA PE=4 SV=1	CAVPO	4
1568	6.5	6.58	53.73	47.01	32.09	tr|H0VKP8|H0VKP8_CAVPO	Uncharacterized protein OS=Cavia porcellus GN=CYB5A PE=3 SV=1	CAVPO	3
1569	6.48	6.52	30.68	30.68	22.16	tr|H0VCR3|H0VCR3_CAVPO	Uncharacterized protein (Fragment) OS=Cavia porcellus GN=RPS10 PE=4 SV=1	CAVPO	3
1570	6.48	6.52	53.17	53.17	31.75	tr|H0VL48|H0VL48_CAVPO	Uncharacterized protein OS=Cavia porcellus GN=Snrpd3 PE=4 SV=1	CAVPO	4
1571	6.48	6.53	13.83	8.99	5.394	tr|H0VLH5|H0VLH5_CAVPO	Uncharacterized protein OS=Cavia porcellus GN=EIF2B5 PE=4 SV=1	CAVPO	3
1572	6.46	6.57	15.23	10.6	10.6	tr|H0UWN8|H0UWN8_CAVPO	Uncharacterized protein OS=Cavia porcellus	CAVPO	4

							GN=PRUNE PE=4 SV=1		
1573	6.46	8.73	59.26	55.56	42.59	tr|H0VUS4|H0VUS4_CAVPO	Uncharacterized protein (Fragment) OS=Cavia porcellus GN=PGRMC2 PE=4 SV=1	CAVPO	5
1574	6.46	7.6	30.8	28.03	17.65	tr|H0W7Q5|H0W7Q5_CAVPO	Uncharacterized protein OS=Cavia porcellus GN=NIPSNAP1 PE=4 SV=1	CAVPO	5
1575	6.45	6.48	17.59	13.89	8.796	tr|H0UW61|H0UW61_CAVPO	Uncharacterized protein (Fragment) OS=Cavia porcellus GN=RSRC2 PE=4 SV=1	CAVPO	3
1576	6.45	6.6	63.93	63.93	29.51	tr|H0V340|H0V340_CAVPO	Uncharacterized protein OS=Cavia porcellus GN=AAMDC PE=4 SV=1	CAVPO	3
1577	6.45	6.75	20.29	8.696	4.928	tr|H0V341|H0V341_CAVPO	Uncharacterized protein (Fragment) OS=Cavia porcellus GN=CRNKL1 PE=4 SV=1	CAVPO	3
1578	6.44	6.55	34.09	25.91	25.91	tr|H0UUX5|H0UUX5_CAVPO	Uncharacterized protein OS=Cavia porcellus GN=CHCHD3 PE=4 SV=1	CAVPO	4
1579	6.42	6.62	12.19	7.286	7.286	tr|H0V3L3|H0V3L3_CAVPO	Uncharacterized protein OS=Cavia porcellus GN=VWA5A PE=4 SV=1	CAVPO	4
1580	6.42	6.87	19.94	12.79	7.036	tr|H0VQQ0|H0VQQ0_CAVPO	Uncharacterized protein OS=Cavia porcellus GN=MSH2 PE=3 SV=1	CAVPO	6
1581	6.42	6.5	28.3	23.58	23.58	tr|Q811W5|Q811W5_CAVPO	Major prion protein (Fragment) OS=Cavia porcellus GN=PRNP PE=3 SV=1	CAVPO	4
1582	6.41	6.45	15.21	10.82	10.82	tr|H0VC37|H0VC37_CAVPO	Uncharacterized protein OS=Cavia porcellus GN=TIA1 PE=4 SV=1	CAVPO	4
1583	6.41	6.53	46.01	24.88	24.88	tr|H0W2H7|H0W2H7_CAVPO	Uncharacterized protein OS=Cavia porcellus GN=TPPP PE=4 SV=1	CAVPO	4
1584	6.4	6.43	16.73	11.74	9.431	tr|H0UVW2|H0UVW2_CAVPO	Uncharacterized protein OS=Cavia porcellus GN=AGFG1 PE=4 SV=1	CAVPO	3
1585	6.4	6.62	19.03	16.8	10.12	tr|H0WDI4|H0WDI4_CAVPO	Uncharacterized protein OS=Cavia porcellus GN=PCYOX1L PE=4 SV=1	CAVPO	4

1586	6.38	6.65	5.591	3.599	2.506	tr|H0UU69|H0UU69_CAVPO	Uncharacterized protein (Fragment) OS=Cavia porcellus GN=DIP2C PE=4 SV=1	CAVPO	4
1587	6.38	6.42	24.93	13.33	11.3	tr|H0V2K0|H0V2K0_CAVPO	Uncharacterized protein (Fragment) OS=Cavia porcellus GN=PHYHIPL PE=4 SV=1	CAVPO	3
1588	6.38	6.57	20.29	16.56	6.275	tr|H0VRK8|H0VRK8_CAVPO	Uncharacterized protein OS=Cavia porcellus GN=MUT PE=4 SV=1	CAVPO	3
1589	6.37	6.83	26.43	21.52	9.426	tr|H0VR62|H0VR62_CAVPO	Uncharacterized protein (Fragment) OS=Cavia porcellus GN=PEPD PE=3 SV=1	CAVPO	4
1590	6.37	6.47	49.25	35.07	35.07	tr|H0VR89|H0VR89_CAVPO	Uncharacterized protein (Fragment) OS=Cavia porcellus GN=NDUFA6 PE=4 SV=1	CAVPO	5
1591	6.36	6.51	18.09	14.76	7.9	tr|H0VM62|H0VM62_CAVPO	Uncharacterized protein (Fragment) OS=Cavia porcellus GN=WRNIP1 PE=4 SV=1	CAVPO	3
1592	6.35	6.47	10.8	5.78	4.907	tr|H0V610|H0V610_CAVPO	Uncharacterized protein (Fragment) OS=Cavia porcellus GN=ATP2C1 PE=4 SV=1	CAVPO	4
1593	6.35	8.24	8.293	4.581	1.633	tr|H0VEH4|H0VEH4_CAVPO	Uncharacterized protein (Fragment) OS=Cavia porcellus PE=4 SV=1	CAVPO	6
1594	6.35	6.38	22.78	18.92	18.92	tr|H0WD98|H0WD98_CAVPO	Uncharacterized protein (Fragment) OS=Cavia porcellus GN=TOLLIP PE=4 SV=1	CAVPO	4
1595	6.34	6.46	14.41	10.65	9.806	tr|H0VKJ8|H0VKJ8_CAVPO	Uncharacterized protein (Fragment) OS=Cavia porcellus GN=SGIP1 PE=4 SV=1	CAVPO	4
1596	6.33	6.54	66.95	57.63	34.75	tr|H0V3M5|H0V3M5_CAVPO	Uncharacterized protein OS=Cavia porcellus GN=LOC100726700 PE=4 SV=1	CAVPO	5
1597	6.3	6.44	32.59	32.59	32.59	tr|H0VRM1|H0VRM1_CAVPO	Uncharacterized protein (Fragment) OS=Cavia porcellus GN=LOC100731223 PE=3 SV=1	CAVPO	4
1598	6.29	6.55	9.984	6.084	4.056	tr|H0UW34|H0UW34_CAVPO	Uncharacterized protein (Fragment) OS=Cavia porcellus GN=ATXN2 PE=4 SV=1	CAVPO	3
1599	6.28	6.44	45.48	41.29	20.32	tr|H0W8Q2|H0W8Q2_CAVPO	NAD-dependent protein deacylase sirtuin-5, mitochondrial OS=Cavia porcellus GN=SIRT5	CAVPO	4

							PE=3 SV=1		
1600	6.27	6.56	17.99	13.39	7.95	tr|H0V5Q0|H0V5Q0_CAVPO	Uncharacterized protein (Fragment) OS=Cavia porcellus GN=CSTF3 PE=4 SV=1	CAVPO	3
1601	6.27	17.4	22.77	15.33	14.14	tr|H0VFG9|H0VFG9_CAVPO	Uncharacterized protein (Fragment) OS=Cavia porcellus GN=STRBP PE=4 SV=1	CAVPO	9
1602	6.26	6.37	22.24	12.87	11.03	tr|H0UYS6|H0UYS6_CAVPO	Eukaryotic translation initiation factor 3 subunit D OS=Cavia porcellus GN=EIF3D PE=3 SV=1	CAVPO	5
1603	6.26	6.53	30.27	22.33	20.6	tr|H0VFZ5|H0VFZ5_CAVPO	Uncharacterized protein (Fragment) OS=Cavia porcellus GN=RPL3 PE=3 SV=1	CAVPO	5
1604	6.26	6.38	47.67	47.67	38.37	tr|H0VPT6|H0VPT6_CAVPO	Uncharacterized protein (Fragment) OS=Cavia porcellus GN=SNRPF PE=4 SV=1	CAVPO	7
1605	6.25	6.34	13.87	13.87	13.87	tr|H0V235|H0V235_CAVPO	Uncharacterized protein (Fragment) OS=Cavia porcellus GN=CADM4 PE=4 SV=1	CAVPO	4
1606	6.24	6.35	39.22	27.78	13.4	tr|H0V0H2|H0V0H2_CAVPO	Uncharacterized protein (Fragment) OS=Cavia porcellus GN=CRYL1 PE=4 SV=1	CAVPO	3
1607	6.24	6.38	54.17	54.17	37.5	tr|H0V7X7|H0V7X7_CAVPO	Uncharacterized protein OS=Cavia porcellus GN=NAA38 PE=4 SV=1	CAVPO	5
1608	6.23	6.4	30.48	23.29	18.84	tr|H0VP73|H0VP73_CAVPO	Uncharacterized protein (Fragment) OS=Cavia porcellus GN=PGAM5 PE=4 SV=1	CAVPO	4
1609	6.23	6.6	10.04	8.185	3.772	tr|H0VW18|H0VW18_CAVPO	Uncharacterized protein OS=Cavia porcellus GN=EDC4 PE=4 SV=1	CAVPO	4
1610	6.22	6.37	7.971	7.005	7.005	tr|H0WAW2|H0WAW2_CAVPO	Uncharacterized protein OS=Cavia porcellus GN=ABCF1 PE=3 SV=1	CAVPO	5
1611	6.2	6.3	16.56	12.27	10.84	tr|H0VBK8|H0VBK8_CAVPO	Uncharacterized protein OS=Cavia porcellus GN=PMPCB PE=3 SV=1	CAVPO	4
1612	6.2	6.36	42.91	37.65	27.94	tr|H0VK62|H0VK62_CAVPO	Uncharacterized protein OS=Cavia porcellus GN=OCIAD1 PE=4 SV=1	CAVPO	4

1613	6.2	6.35	43.55	36.02	31.18	tr|H0W5U3|H0W5U3_CAVPO	Uncharacterized protein OS=Cavia porcellus GN=Ndufb8 PE=4 SV=1	CAVPO	4
1614	6.19	6.46	18.6	12.88	7.01	tr|H0UZ86|H0UZ86_CAVPO	Uncharacterized protein (Fragment) OS=Cavia porcellus GN=Acsl1 PE=4 SV=1	CAVPO	5
1615	6.18	6.61	38.1	24.13	17.78	tr|H0VH22|H0VH22_CAVPO	Uncharacterized protein OS=Cavia porcellus GN=WDR82 PE=4 SV=1	CAVPO	4
1616	6.17	6.34	16.22	11.64	11.64	tr|H0VQZ8|H0VQZ8_CAVPO	Uncharacterized protein OS=Cavia porcellus GN=ABI1 PE=4 SV=1	CAVPO	4
1617	6.17	17.1	73.91	67.7	58.39	tr|H0W2A1|H0W2A1_CAVPO	Nucleoside diphosphate kinase (Fragment) OS=Cavia porcellus GN=Nme1 PE=3 SV=1	CAVPO	11
1618	6.17	6.3	30.75	20.42	7.746	tr|H0W673|H0W673_CAVPO	Uncharacterized protein OS=Cavia porcellus GN=IVD PE=3 SV=1	CAVPO	3
1619	6.16	6.55	21.11	16.48	10.74	tr|H0VFB2|H0VFB2_CAVPO	Uncharacterized protein (Fragment) OS=Cavia porcellus GN=SLC27A1 PE=4 SV=1	CAVPO	5
1620	6.15	6.17	43.9	39.84	31.71	tr|H0UZK8|H0UZK8_CAVPO	Uncharacterized protein OS=Cavia porcellus GN=Txndc17 PE=4 SV=1	CAVPO	3
1621	6.15	6.17	41.44	41.44	41.44	tr|H0V7R6|H0V7R6_CAVPO	Cytochrome c oxidase subunit 6A, mitochondrial (Fragment) OS=Cavia porcellus GN=LOC100714805 PE=3 SV=1	CAVPO	4
1622	6.15	6.18	21.47	14.42	14.42	tr|H0V8Y9|H0V8Y9_CAVPO	Uncharacterized protein OS=Cavia porcellus GN=SYP PE=4 SV=1	CAVPO	6
1623	6.15	6.27	32.4	27.93	24.58	tr|H0W2P6|H0W2P6_CAVPO	Uncharacterized protein OS=Cavia porcellus GN=PTRH2 PE=4 SV=1	CAVPO	3
1624	6.14	6.29	19.02	14.88	8.286	tr|H0UVE3|H0UVE3_CAVPO	Uncharacterized protein (Fragment) OS=Cavia porcellus GN=CLCC1 PE=4 SV=1	CAVPO	3
1625	6.14	21.2	13.78	7.248	4.818	tr|H0VPB1|H0VPB1_CAVPO	Uncharacterized protein OS=Cavia porcellus GN=SPTBN2 PE=4 SV=1	CAVPO	11
1626	6.14	6.35	21.6	20.27	8.686	tr|H0W9Y5|H0W9Y5_CAVPO	Uncharacterized protein OS=Cavia porcellus	CAVPO	3

							GN=UBXN6 PE=4 SV=1		
1627	6.13	6.15	52.56	46.79	35.9	tr|H0V870|H0V870_CAVPO	Uncharacterized protein OS=Cavia porcellus GN=GAP43 PE=4 SV=1	CAVPO	3
1628	6.13	6.47	9.595	4.296	1.826	tr|H0VBT5|H0VBT5_CAVPO	Uncharacterized protein (Fragment) OS=Cavia porcellus GN=LRBA PE=4 SV=1	CAVPO	5
1629	6.13	6.26	28.13	21.02	17.9	tr|H0WCF1|H0WCF1_CAVPO	Uncharacterized protein OS=Cavia porcellus GN=PTRF PE=4 SV=1	CAVPO	7
1630	6.12	6.22	54.88	46.34	36.59	tr|H0UY09|H0UY09_CAVPO	Uncharacterized protein OS=Cavia porcellus GN=NDUFA4 PE=4 SV=1	CAVPO	3
1631	6.12	7.5	9.806	5.226	2.909	tr|H0V3B5|H0V3B5_CAVPO	Uncharacterized protein OS=Cavia porcellus GN=GBF1 PE=4 SV=1	CAVPO	5
1632	6.12	6.4	27.65	14.85	7.509	tr|H0VMM0|H0VMM0_CAVPO	Uncharacterized protein OS=Cavia porcellus GN=ACSF3 PE=4 SV=1	CAVPO	4
1633	6.12	6.28	35.29	24.79	24.79	tr|H0VU14|H0VU14_CAVPO	Uncharacterized protein (Fragment) OS=Cavia porcellus GN=SCAMP5 PE=4 SV=1	CAVPO	4
1634	6.11	6.2	24.9	20.82	13.88	tr|H0UT89|H0UT89_CAVPO	Uncharacterized protein OS=Cavia porcellus GN=TFAM PE=4 SV=1	CAVPO	3
1635	6.11	6.22	31.52	23.91	17.93	tr|H0VAC6|H0VAC6_CAVPO	Uncharacterized protein OS=Cavia porcellus GN=RHEB PE=4 SV=1	CAVPO	3
1636	6.11	6.13	49.35	29.87	29.87	tr|H0VPS7|H0VPS7_CAVPO	Uncharacterized protein OS=Cavia porcellus GN=PFDN2 PE=4 SV=1	CAVPO	5
1637	6.1	6.11	34.19	20.96	20.96	tr|H0VFR4|H0VFR4_CAVPO	Uncharacterized protein OS=Cavia porcellus GN=TIPRL PE=4 SV=1	CAVPO	5
1638	6.1	6.11	14.01	5.839	5.839	tr|H0VKK2|H0VKK2_CAVPO	Uncharacterized protein OS=Cavia porcellus GN=SAMD11 PE=4 SV=1	CAVPO	3
1639	6.1	6.24	30.38	27.43	20.25	tr|H0VP30|H0VP30_CAVPO	Uncharacterized protein (Fragment) OS=Cavia porcellus GN=NQO2 PE=4 SV=1	CAVPO	4

1640	6.08	10.5	22.16	15.8	13.68	tr|H0VG43|H0VG43_CAVPO	Uncharacterized protein OS=Cavia porcellus GN=SNX2 PE=4 SV=1	CAVPO	6
1641	6.08	6.3	15.45	15.45	15.45	tr|H0VLM9|H0VLM9_CAVPO	Uncharacterized protein OS=Cavia porcellus GN=TPP1 PE=4 SV=1	CAVPO	6
1642	6.07	6.17	4.674	3.635	3.635	tr|H0VFU2|H0VFU2_CAVPO	Ubiquitin carboxyl-terminal hydrolase OS=Cavia porcellus GN=USP19 PE=3 SV=1	CAVPO	4
1643	6.07	6.16	7.67	6.534	5.256	tr|H0W9U5|H0W9U5_CAVPO	Uncharacterized protein OS=Cavia porcellus GN=RNF214 PE=4 SV=1	CAVPO	3
1644	6.06	6.07	32.3	22.12	22.12	tr|H0VFF8|H0VFF8_CAVPO	Uncharacterized protein OS=Cavia porcellus GN=CHCHD6 PE=4 SV=1	CAVPO	3
1645	6.05	7.49	34.05	29.75	19.02	tr|H0V1Z1|H0V1Z1_CAVPO	Uncharacterized protein OS=Cavia porcellus GN=MAPRE2 PE=4 SV=1	CAVPO	5
1646	6.04	6.05	28.71	28.71	28.71	tr|H0V0V8|H0V0V8_CAVPO	Uncharacterized protein OS=Cavia porcellus GN=TMED2 PE=3 SV=1	CAVPO	4
1647	6.04	6.15	33.9	33.9	29.94	tr|H0W038|H0W038_CAVPO	Uncharacterized protein OS=Cavia porcellus GN=MXRA7 PE=4 SV=1	CAVPO	5
1648	6.03	6.16	18.15	13.31	7.863	tr|H0VHW8|H0VHW8_CAVPO	Uncharacterized protein OS=Cavia porcellus GN=WDR37 PE=4 SV=1	CAVPO	3
1649	6.02	6.03	27.08	21.53	21.53	tr|H0W934|H0W934_CAVPO	Uncharacterized protein OS=Cavia porcellus GN=LOC100731197 PE=3 SV=1	CAVPO	3
1650	6.01	6.01	20.89	20.89	20.89	tr|H0UZJ1|H0UZJ1_CAVPO	Uncharacterized protein OS=Cavia porcellus GN=ACP1 PE=4 SV=1	CAVPO	4
1651	6.01	6.01	25	20.61	20.61	tr|H0V5Y1|H0V5Y1_CAVPO	Uncharacterized protein (Fragment) OS=Cavia porcellus GN=FAM3C PE=4 SV=1	CAVPO	3
1652	6	6	8.462	8.462	8.462	tr|A9Z0V9|A9Z0V9_CAVPO	Carbonic anhydrase 3 OS=Cavia porcellus GN=CA3 PE=2 SV=1	CAVPO	3
1653	6	6	34.02	34.02	34.02	tr|H0UU57|H0UU57_CAVPO	Uncharacterized protein OS=Cavia porcellus GN=TIMM8A PE=4 SV=1	CAVPO	3

1654	6	6	42.22	42.22	42.22	tr|H0UUV1|H0UUV1_CAVPO	Uncharacterized protein (Fragment) OS=Cavia porcellus GN=HN1 PE=4 SV=1	CAVPO	3
1655	6	6	24.84	24.84	24.84	tr|H0UUY0|H0UUY0_CAVPO	Uncharacterized protein OS=Cavia porcellus GN=NUDCD2 PE=4 SV=1	CAVPO	3
1656	6	6	19.86	14.08	14.08	tr|H0V1F7|H0V1F7_CAVPO	Uncharacterized protein OS=Cavia porcellus GN=C1QBP PE=4 SV=1	CAVPO	3
1657	6	6	28.57	24.6	24.6	tr|H0V1V2|H0V1V2_CAVPO	Uncharacterized protein OS=Cavia porcellus GN=MPC2 PE=4 SV=1	CAVPO	3
1658	6	6	54.55	54.55	54.55	tr|H0V7L1|H0V7L1_CAVPO	Uncharacterized protein OS=Cavia porcellus GN=DPY30 PE=4 SV=1	CAVPO	3
1659	6	6.01	22.39	19.4	19.4	tr|H0VBW8|H0VBW8_CAVPO	Uncharacterized protein OS=Cavia porcellus GN=CHMP6 PE=4 SV=1	CAVPO	3
1660	6	6	33.81	33.81	33.81	tr|H0VEA9|H0VEA9_CAVPO	Uncharacterized protein OS=Cavia porcellus GN=RTCA PE=4 SV=1	CAVPO	3
1661	6	6.18	15.14	9.031	6.507	tr|H0VQ12|H0VQ12_CAVPO	Uncharacterized protein OS=Cavia porcellus GN=TFRC PE=4 SV=1	CAVPO	4
1662	6	6.05	11.98	7.385	7.385	tr|H0VRG5|H0VRG5_CAVPO	Uncharacterized protein OS=Cavia porcellus PE=4 SV=1	CAVPO	3
1663	6	6	32.5	32.5	32.5	tr|H0VT87|H0VT87_CAVPO	Uncharacterized protein OS=Cavia porcellus GN=LSM6 PE=4 SV=1	CAVPO	3
1664	6	6.01	39.02	39.02	39.02	tr|H0VV27|H0VV27_CAVPO	40S ribosomal protein S27 (Fragment) OS=Cavia porcellus GN=Rps27 PE=3 SV=1	CAVPO	4
1665	6	6	9.16	9.16	9.16	tr|H0VYG7|H0VYG7_CAVPO	Uncharacterized protein (Fragment) OS=Cavia porcellus GN=LAMP1 PE=4 SV=1	CAVPO	3
1666	6	8	22.54	21.97	21.97	tr|H0W3B9|H0W3B9_CAVPO	Uncharacterized protein OS=Cavia porcellus GN=HIST1H1E PE=3 SV=1	CAVPO	4
1667	6	6	41.61	41.61	31.06	tr|Q9WUR5|Q9WUR5_CAVPO	Thy-1 protein OS=Cavia porcellus GN=Thy-1 PE=2 SV=1	CAVPO	3

1668	5.99	6.04	11.79	6.534	4.784	tr|H0VT68|H0VT68_CAVPO	Uncharacterized protein OS=Cavia porcellus GN=FDPS PE=4 SV=1	CAVPO	3
1669	5.96	6.12	17.01	10.72	7.579	tr|H0VS64|H0VS64_CAVPO	Uncharacterized protein (Fragment) OS=Cavia porcellus GN=LRRC47 PE=4 SV=1	CAVPO	3
1670	5.94	6.98	35.32	24.11	12.65	tr|H0W2Z8|H0W2Z8_CAVPO	Uncharacterized protein (Fragment) OS=Cavia porcellus GN=CNP PE=4 SV=1	CAVPO	4
1671	5.93	6.15	19.77	19.48	19.48	tr|H0VH54|H0VH54_CAVPO	Uncharacterized protein (Fragment) OS=Cavia porcellus GN=DNAJB4 PE=4 SV=1	CAVPO	5
1672	5.92	6.09	19.86	6.575	5.616	tr|H0UT96|H0UT96_CAVPO	Uncharacterized protein (Fragment) OS=Cavia porcellus GN=HOOK1 PE=4 SV=1	CAVPO	3
1673	5.92	6.02	23.44	17.8	9.496	tr|H0VPY5|H0VPY5_CAVPO	Uncharacterized protein OS=Cavia porcellus GN=ERI3 PE=4 SV=1	CAVPO	3
1674	5.92	5.99	33.99	33.99	28.76	tr|H0VRN0|H0VRN0_CAVPO	Actin-related protein 2/3 complex subunit 5 OS=Cavia porcellus GN=ARPC5L PE=3 SV=1	CAVPO	4
1675	5.91	6	22.05	18.73	14.2	tr|H0VD47|H0VD47_CAVPO	Uncharacterized protein OS=Cavia porcellus GN=DFFA PE=4 SV=1	CAVPO	3
1676	5.91	8.24	14.29	14.29	12.24	tr|H0VEC9|H0VEC9_CAVPO	Uncharacterized protein (Fragment) OS=Cavia porcellus GN=PPP2R5A PE=4 SV=1	CAVPO	5
1677	5.91	5.98	28.92	28.92	24.7	tr|H0VGN5|H0VGN5_CAVPO	Peptidyl-prolyl cis-trans isomerase OS=Cavia porcellus GN=PPIL1 PE=3 SV=1	CAVPO	3
1678	5.89	5.97	50	50	50	tr|H0VVN9|H0VVN9_CAVPO	Uncharacterized protein (Fragment) OS=Cavia porcellus GN=ATOX1 PE=4 SV=1	CAVPO	3
1679	5.88	5.97	15.28	7.776	4.775	tr|H0VKX8|H0VKX8_CAVPO	Uncharacterized protein (Fragment) OS=Cavia porcellus GN=XRCC5 PE=4 SV=1	CAVPO	3
1680	5.87	5.95	26.67	22.96	22.96	tr|H0V582|H0V582_CAVPO	Uncharacterized protein OS=Cavia porcellus GN=LHPP PE=4 SV=1	CAVPO	4
1681	5.86	6.08	47.22	36.57	26.85	tr|H0V5S9|H0V5S9_CAVPO	Ribosomal protein (Fragment) OS=Cavia porcellus GN=RPL10A PE=3 SV=1	CAVPO	5

1682	5.85	5.94	27.31	19.68	13.25	tr|H0V1C8|H0V1C8_CAVPO	Uncharacterized protein (Fragment) OS=Cavia porcellus GN=LRRC57 PE=4 SV=1	CAVPO	3
1683	5.85	6.11	51.19	51.19	42.86	tr|H0V252|H0V252_CAVPO	Uncharacterized protein (Fragment) OS=Cavia porcellus GN=ACBD7 PE=4 SV=1	CAVPO	3
1684	5.85	5.92	24.12	24.12	18.59	tr|H0VAK4|H0VAK4_CAVPO	Uncharacterized protein OS=Cavia porcellus GN=DIRAS2 PE=4 SV=1	CAVPO	3
1685	5.85	9.4	26.27	21.2	16.14	tr|H0VY24|H0VY24_CAVPO	Uncharacterized protein OS=Cavia porcellus GN=Akr1b10 PE=4 SV=1	CAVPO	6
1686	5.84	6.01	20.59	12.74	8.917	tr|H0VFU5|H0VFU5_CAVPO	Uncharacterized protein OS=Cavia porcellus GN=CPSF7 PE=4 SV=1	CAVPO	3
1687	5.84	5.9	42.78	32.22	32.22	tr|H0W8T2|H0W8T2_CAVPO	Uncharacterized protein (Fragment) OS=Cavia porcellus GN=PDAP1 PE=4 SV=1	CAVPO	5
1688	5.83	6.02	32.35	19.41	11.65	tr|H0V1M8|H0V1M8_CAVPO	Uncharacterized protein OS=Cavia porcellus GN=ATL3 PE=4 SV=1	CAVPO	5
1689	5.83	6.03	12.46	9.044	5.973	tr|H0VXF7|H0VXF7_CAVPO	Uncharacterized protein OS=Cavia porcellus GN=SETD3 PE=4 SV=1	CAVPO	3
1690	5.82	6.18	12.11	8.993	6.954	tr|H0WBE6|H0WBE6_CAVPO	Uncharacterized protein OS=Cavia porcellus GN=MOGS PE=4 SV=1	CAVPO	4
1691	5.81	5.89	14.63	8.657	5.97	tr|H0VG58|H0VG58_CAVPO	Uncharacterized protein (Fragment) OS=Cavia porcellus GN=Wdr92 PE=4 SV=1	CAVPO	3
1692	5.81	5.91	41.22	34.46	34.46	tr|H0VT51|H0VT51_CAVPO	Uncharacterized protein (Fragment) OS=Cavia porcellus GN=MGST3 PE=4 SV=1	CAVPO	3
1693	5.79	6.1	23.11	12.13	10.53	tr|H0UYD7|H0UYD7_CAVPO	Uncharacterized protein OS=Cavia porcellus GN=ETF1 PE=4 SV=1	CAVPO	4
1694	5.79	5.85	34.08	28.49	22.91	tr|H0V8L2|H0V8L2_CAVPO	Uncharacterized protein (Fragment) OS=Cavia porcellus GN=PBDC1 PE=4 SV=1	CAVPO	3
1695	5.78	6.1	9.236	6.25	3.264	tr|H0VJ73|H0VJ73_CAVPO	Uncharacterized protein OS=Cavia porcellus GN=CPSF1 PE=4 SV=1	CAVPO	4

1696	5.77	6.31	18.78	17.09	10.55	tr|H0V2L6|H0V2L6_CAVPO	Uncharacterized protein OS=Cavia porcellus GN=GSS PE=4 SV=1	CAVPO	4
1697	5.77	5.82	31.01	31.01	31.01	tr|H0VK68|H0VK68_CAVPO	Uncharacterized protein OS=Cavia porcellus GN=ISCA1 PE=4 SV=1	CAVPO	3
1698	5.77	6.06	11.19	6.454	4.085	tr|H0VQP5|H0VQP5_CAVPO	Uncharacterized protein OS=Cavia porcellus GN=WDR11 PE=4 SV=1	CAVPO	3
1699	5.75	6	14.45	11.85	8.96	tr|H0V7S1|H0V7S1_CAVPO	Uncharacterized protein OS=Cavia porcellus GN=GHITM PE=4 SV=1	CAVPO	3
1700	5.75	5.96	22.43	15.33	13.27	tr|H0VS05|H0VS05_CAVPO	Uncharacterized protein (Fragment) OS=Cavia porcellus GN=AMER2 PE=4 SV=1	CAVPO	4
1701	5.74	5.8	28.32	28.32	28.32	tr|H0UUV7|H0UUV7_CAVPO	Dolichyl-diphosphooligosaccharide--protein glycosyltransferase subunit DAD1 OS=Cavia porcellus GN=DAD1 PE=3 SV=1	CAVPO	3
1702	5.74	6.01	14.18	9.929	3.445	tr|H0UY84|H0UY84_CAVPO	Uncharacterized protein (Fragment) OS=Cavia porcellus GN=Golga2 PE=4 SV=1	CAVPO	3
1703	5.73	5.94	15.53	13.27	5.502	tr|H0V3F8|H0V3F8_CAVPO	Uncharacterized protein OS=Cavia porcellus GN=ETFDH PE=4 SV=1	CAVPO	3
1704	5.72	5.8	20.21	11.45	7.156	tr|H0VAC3|H0VAC3_CAVPO	Uncharacterized protein OS=Cavia porcellus GN=DNAJC11 PE=4 SV=1	CAVPO	3
1705	5.72	5.93	68.29	68.29	68.29	tr|H0W408|H0W408_CAVPO	Uncharacterized protein OS=Cavia porcellus GN=UQCRQ PE=4 SV=1	CAVPO	7
1706	5.72	5.8	16.46	14.35	14.35	tr|H0WB85|H0WB85_CAVPO	Uncharacterized protein OS=Cavia porcellus GN=Srsf10 PE=4 SV=1	CAVPO	3
1707	5.71	5.93	28.57	24.88	16.59	tr|H0VQX8|H0VQX8_CAVPO	Uncharacterized protein (Fragment) OS=Cavia porcellus GN=Tpd52l2 PE=4 SV=1	CAVPO	3
1708	5.7	5.83	16.74	8.748	7.541	tr|H0UTL4|H0UTL4_CAVPO	Uncharacterized protein OS=Cavia porcellus GN=SPG20 PE=4 SV=1	CAVPO	3
1709	5.7	5.74	38	38	38	tr|H0UWE8|H0UWE8_CAVPO	Uncharacterized protein OS=Cavia porcellus	CAVPO	6

							GN=CEND1 PE=4 SV=1		
1710	5.7	5.89	23.57	14.26	7.034	tr|H0VDD6|H0VDD6_CAVPO	Uncharacterized protein OS=Cavia porcellus GN=OXSR1 PE=4 SV=1	CAVPO	3
1711	5.69	5.83	60.2	50	50	tr|H0VPK3|H0VPK3_CAVPO	Uncharacterized protein (Fragment) OS=Cavia porcellus GN=DYNLRB1 PE=4 SV=1	CAVPO	3
1712	5.69	5.97	20.96	16.17	14.12	tr|H0WCB3|H0WCB3_CAVPO	Uncharacterized protein OS=Cavia porcellus GN=GCDH PE=3 SV=1	CAVPO	4
1713	5.68	5.81	26.8	14.95	11.65	tr|H0V0Z1|H0V0Z1_CAVPO	Uncharacterized protein OS=Cavia porcellus GN=PLRG1 PE=4 SV=1	CAVPO	3
1714	5.68	5.75	10.34	6.466	6.466	tr|H0WBF9|H0WBF9_CAVPO	Uncharacterized protein OS=Cavia porcellus GN=LSM14A PE=4 SV=1	CAVPO	3
1715	5.67	5.77	54.9	54.9	49.02	tr|H0V8B7|H0V8B7_CAVPO	Uncharacterized protein OS=Cavia porcellus GN=LSM3 PE=4 SV=1	CAVPO	4
1716	5.67	5.75	63.36	54.96	41.98	tr|H0VR11|H0VR11_CAVPO	Uncharacterized protein OS=Cavia porcellus GN=NDUFB7 PE=4 SV=1	CAVPO	5
1717	5.66	12.3	44.59	34.39	28.34	tr|H0W492|H0W492_CAVPO	Uncharacterized protein (Fragment) OS=Cavia porcellus GN=GNA11 PE=4 SV=1	CAVPO	8
1718	5.65	5.91	27.42	19.86	16.08	tr|H0V1D7|H0V1D7_CAVPO	Uncharacterized protein OS=Cavia porcellus GN=AP1M1 PE=4 SV=1	CAVPO	5
1719	5.65	5.75	40.91	40.91	40.91	tr|H0W1J3|H0W1J3_CAVPO	Uncharacterized protein (Fragment) OS=Cavia porcellus GN=RBM8A PE=4 SV=1	CAVPO	4
1720	5.65	5.76	20.07	15.14	15.14	tr|H0W3P4|H0W3P4_CAVPO	Uncharacterized protein (Fragment) OS=Cavia porcellus GN=UBE2Z PE=4 SV=1	CAVPO	4
1721	5.63	5.7	27.18	20.58	20.58	tr|H0VC46|H0VC46_CAVPO	Uncharacterized protein (Fragment) OS=Cavia porcellus GN=RAD23A PE=4 SV=1	CAVPO	3
1722	5.63	7.81	34.83	22.47	22.47	tr|H0VHK3|H0VHK3_CAVPO	Uncharacterized protein (Fragment) OS=Cavia porcellus GN=TRA2A PE=4 SV=1	CAVPO	8

1723	5.63	5.78	40.49	36.81	19.63	tr|H0W525|H0W525_CAVPO	Uncharacterized protein (Fragment) OS=Cavia porcellus GN=NDUFA13 PE=4 SV=1	CAVPO	3
1724	5.62	5.8	8.663	7.533	6.026	tr|H0VG88|H0VG88_CAVPO	Uncharacterized protein (Fragment) OS=Cavia porcellus GN=CSPG5 PE=4 SV=1	CAVPO	3
1725	5.61	12.3	45.29	29.58	16.75	tr|H0UYM7|H0UYM7_CAVPO	Uncharacterized protein OS=Cavia porcellus GN=PPM1A PE=3 SV=1	CAVPO	7
1726	5.6	5.73	18.75	15.87	7.372	tr|H0VJB8|H0VJB8_CAVPO	Uncharacterized protein OS=Cavia porcellus GN=Papss1 PE=4 SV=1	CAVPO	3
1727	5.6	5.93	60.29	50	45.59	tr|H0VLQ4|H0VLQ4_CAVPO	Histone H3 OS=Cavia porcellus GN=LOC100735425 PE=3 SV=1	CAVPO	11
1728	5.59	5.87	15.24	15.24	9.959	tr|H0UVF0|H0UVF0_CAVPO	Uncharacterized protein OS=Cavia porcellus GN=Mlycd PE=4 SV=1	CAVPO	3
1729	5.58	5.79	16.45	14.04	14.04	tr|H0UYC3|H0UYC3_CAVPO	Adenylosuccinate synthetase (Fragment) OS=Cavia porcellus GN=ADSS PE=3 SV=1	CAVPO	4
1730	5.57	5.9	12.07	7.462	4.243	tr|H0V5D0|H0V5D0_CAVPO	Tyrosine-protein kinase receptor OS=Cavia porcellus GN=IGF1R PE=3 SV=1	CAVPO	5
1731	5.57	5.7	16.83	10	5.167	tr|H0V9F9|H0V9F9_CAVPO	Uncharacterized protein OS=Cavia porcellus GN=GLMN PE=4 SV=1	CAVPO	3
1732	5.57	5.63	16.81	13.11	13.11	tr|H0VBP5|H0VBP5_CAVPO	Uncharacterized protein OS=Cavia porcellus GN=EIF2B2 PE=3 SV=1	CAVPO	3
1733	5.56	5.65	31.73	24.5	16.87	tr|H0V6E1|H0V6E1_CAVPO	Uncharacterized protein OS=Cavia porcellus GN=CHTOP PE=4 SV=1	CAVPO	3
1734	5.56	5.78	15.02	10.41	8.362	tr|H0VP48|H0VP48_CAVPO	CTP synthase OS=Cavia porcellus GN=CTPS2 PE=3 SV=1	CAVPO	4
1735	5.55	5.91	31.48	19.5	7.799	tr|H0UZY8|H0UZY8_CAVPO	Uncharacterized protein (Fragment) OS=Cavia porcellus GN=RPL4 PE=4 SV=1	CAVPO	3
1736	5.54	5.61	29.96	24.05	14.35	tr|H0UT97|H0UT97_CAVPO	Uncharacterized protein OS=Cavia porcellus GN=THEM4 PE=4 SV=1	CAVPO	3

1737	5.54	5.75	34.19	21.61	16.45	tr|H0V6I5|H0V6I5_CAVPO	Uncharacterized protein OS=Cavia porcellus GN=PSMD14 PE=4 SV=1	CAVPO	5
1738	5.54	5.73	25.78	23.11	23.11	tr|H0VM51|H0VM51_CAVPO	Uncharacterized protein OS=Cavia porcellus GN=MOB4 PE=4 SV=1	CAVPO	4
1739	5.53	5.72	16.12	7.072	7.072	tr|H0VAW6|H0VAW6_CAVPO	Uncharacterized protein (Fragment) OS=Cavia porcellus GN=NPLOC4 PE=4 SV=1	CAVPO	4
1740	5.53	5.59	16.12	13.43	11.04	tr|H0VL10|H0VL10_CAVPO	Uncharacterized protein OS=Cavia porcellus GN=XRCC4 PE=4 SV=1	CAVPO	3
1741	5.52	5.73	10.36	10.36	6.554	tr|H0VH34|H0VH34_CAVPO	Coronin OS=Cavia porcellus GN=CORO1B PE=3 SV=1	CAVPO	3
1742	5.51	5.71	16.74	8.913	5.435	tr|H0UVE4|H0UVE4_CAVPO	Uncharacterized protein OS=Cavia porcellus GN=WDR47 PE=4 SV=1	CAVPO	3
1743	5.5	5.59	32.84	31.37	19.12	tr|H0WBB4|H0WBB4_CAVPO	Uncharacterized protein OS=Cavia porcellus GN=RPS5 PE=3 SV=1	CAVPO	3
1744	5.49	5.57	6.617	3.792	3.197	tr|H0V593|H0V593_CAVPO	Uncharacterized protein OS=Cavia porcellus GN=MAPK8IP3 PE=4 SV=1	CAVPO	3
1745	5.49	5.59	20.86	20.86	16.55	tr|H0W9Y6|H0W9Y6_CAVPO	Uncharacterized protein (Fragment) OS=Cavia porcellus GN=LSM4 PE=4 SV=1	CAVPO	3
1746	5.47	5.6	12.68	9.731	4.738	tr|H0VI82|H0VI82_CAVPO	Uncharacterized protein OS=Cavia porcellus GN=PNPT1 PE=4 SV=1	CAVPO	3
1747	5.47	5.72	11.6	7.797	4.533	tr|H0W1K1|H0W1K1_CAVPO	Uncharacterized protein OS=Cavia porcellus GN=ADNP PE=3 SV=1	CAVPO	4
1748	5.46	5.65	22.84	12.53	12.53	tr|H0V3D3|H0V3D3_CAVPO	Uncharacterized protein OS=Cavia porcellus GN=MAB21L1 PE=4 SV=1	CAVPO	4
1749	5.46	5.56	36.65	33.94	28.51	tr|H0V681|H0V681_CAVPO	Uncharacterized protein OS=Cavia porcellus GN=VPS28 PE=4 SV=1	CAVPO	6
1750	5.46	5.56	23.85	23.85	15.9	tr|H0V9N0|H0V9N0_CAVPO	Uncharacterized protein OS=Cavia porcellus GN=C12orf43 PE=4 SV=1	CAVPO	3

1751	5.46	5.55	17.71	14.39	14.39	tr|H0VPH5|H0VPH5_CAVPO	Uncharacterized protein OS=Cavia porcellus GN=PSMF1 PE=4 SV=1	CAVPO	3
1752	5.46	5.57	45	31.87	27.5	tr|H0WB05|H0WB05_CAVPO	Uncharacterized protein OS=Cavia porcellus PE=4 SV=1	CAVPO	3
1753	5.45	5.54	26.2	26.2	18.78	tr|H0V7D0|H0V7D0_CAVPO	Uncharacterized protein OS=Cavia porcellus GN=CWC15 PE=4 SV=1	CAVPO	4
1754	5.43	5.48	11.68	11.4	11.4	tr|H0UWC5|H0UWC5_CAVPO	Uncharacterized protein (Fragment) OS=Cavia porcellus GN=TWF1 PE=4 SV=1	CAVPO	4
1755	5.43	5.6	41.58	32.63	26.32	tr|H0V592|H0V592_CAVPO	Uncharacterized protein OS=Cavia porcellus GN=HN1L PE=4 SV=1	CAVPO	3
1756	5.43	6.23	10.29	6.327	3.211	tr|H0VB26|H0VB26_CAVPO	Uncharacterized protein OS=Cavia porcellus GN=HERC4 PE=4 SV=1	CAVPO	3
1757	5.42	25.9	42.62	38.17	36.53	tr|H0VCR5|H0VCR5_CAVPO	Uncharacterized protein OS=Cavia porcellus GN=DDX39A PE=4 SV=1	CAVPO	14
1758	5.41	5.59	19.55	12.18	9.615	tr|H0V4L8|H0V4L8_CAVPO	Uncharacterized protein OS=Cavia porcellus GN=PRPF38A PE=4 SV=1	CAVPO	3
1759	5.41	5.64	17.87	15.37	9.067	tr|H0V715|H0V715_CAVPO	Uncharacterized protein (Fragment) OS=Cavia porcellus GN=TRIM2 PE=4 SV=1	CAVPO	4
1760	5.41	5.71	11.15	5.874	2.825	tr|H0VLY1|H0VLY1_CAVPO	Uncharacterized protein (Fragment) OS=Cavia porcellus GN=ROCK2 PE=4 SV=1	CAVPO	3
1761	5.4	18.1	20.72	13.99	13.23	tr|H0VJ28|H0VJ28_CAVPO	Uncharacterized protein OS=Cavia porcellus GN=SAFB2 PE=4 SV=1	CAVPO	9
1762	5.4	5.51	18.44	9.886	9.886	tr|H0W984|H0W984_CAVPO	Uncharacterized protein OS=Cavia porcellus GN=LOC100727060 PE=3 SV=1	CAVPO	4
1763	5.38	5.56	27.56	27.56	26.92	tr|H0UW86|H0UW86_CAVPO	Uncharacterized protein OS=Cavia porcellus GN=RPL23A PE=3 SV=1	CAVPO	4
1764	5.38	5.59	6.141	4.87	2.065	tr|H0VDS4|H0VDS4_CAVPO	Uncharacterized protein OS=Cavia porcellus GN=NUP210 PE=4 SV=1	CAVPO	3

1765	5.38	5.63	13.43	8.277	3.125	tr|H0VWT1|H0VWT1_CAVPO	Uncharacterized protein (Fragment) OS=Cavia porcellus GN=SRCIN1 PE=4 SV=1	CAVPO	3
1766	5.38	5.58	42.77	28.32	19.08	tr|H0WD42|H0WD42_CAVPO	Uncharacterized protein (Fragment) OS=Cavia porcellus GN=UBL4A PE=4 SV=1	CAVPO	3
1767	5.37	5.8	13.66	9.247	7.849	tr|H0VGI1|H0VGI1_CAVPO	Ubiquitin carboxyl-terminal hydrolase (Fragment) OS=Cavia porcellus GN=USP4 PE=3 SV=1	CAVPO	6
1768	5.35	5.48	26.9	19.3	11.39	tr|H0UZZ9|H0UZZ9_CAVPO	Uncharacterized protein OS=Cavia porcellus GN=HMOX2 PE=4 SV=1	CAVPO	3
1769	5.35	5.68	18.01	11.14	4.922	tr|H0V2X9|H0V2X9_CAVPO	Uncharacterized protein OS=Cavia porcellus GN=CPT1A PE=3 SV=1	CAVPO	3
1770	5.35	5.54	30.17	10.83	8.511	tr|H0VHC9|H0VHC9_CAVPO	Uncharacterized protein (Fragment) OS=Cavia porcellus GN=SNW1 PE=4 SV=1	CAVPO	3
1771	5.35	5.49	17.72	13.29	9.072	tr|H0VL93|H0VL93_CAVPO	Uncharacterized protein OS=Cavia porcellus GN=KCTD8 PE=4 SV=1	CAVPO	3
1772	5.34	5.4	33.64	33.64	33.64	tr|Q60479|Q60479_CAVPO	Glutathione peroxidase (Fragment) OS=Cavia porcellus PE=2 SV=1	CAVPO	3
1773	5.33	5.4	17.89	17.89	17.37	tr|H0V247|H0V247_CAVPO	Uncharacterized protein OS=Cavia porcellus GN=LZIC PE=4 SV=1	CAVPO	3
1774	5.33	5.66	28.34	19.82	15.44	tr|H0V3R2|H0V3R2_CAVPO	Uncharacterized protein OS=Cavia porcellus GN=WIPI2 PE=4 SV=1	CAVPO	4
1775	5.33	5.6	13.48	7.961	1.746	tr|H0VBK5|H0VBK5_CAVPO	Uncharacterized protein OS=Cavia porcellus GN=FYCO1 PE=4 SV=1	CAVPO	3
1776	5.33	5.41	25.35	25.35	17.97	tr|H0VLX8|H0VLX8_CAVPO	GrpE protein homolog OS=Cavia porcellus GN=GRPEL1 PE=3 SV=1	CAVPO	3
1777	5.31	5.5	12.01	12.01	5.489	tr|H0VS51|H0VS51_CAVPO	Uncharacterized protein OS=Cavia porcellus GN=Dcaf8 PE=4 SV=1	CAVPO	3
1778	5.28	5.59	37.83	25.51	14.96	tr|H0VI70|H0VI70_CAVPO	Uncharacterized protein OS=Cavia porcellus	CAVPO	3

							GN=CCDC104 PE=4 SV=1		
1779	5.27	5.52	18.61	18.61	18.61	tr|H0UTH3|H0UTH3_CAVPO	Uncharacterized protein OS=Cavia porcellus GN=NECAP1 PE=4 SV=1	CAVPO	4
1780	5.25	5.37	36.82	29.71	12.13	tr|H0V0N8|H0V0N8_CAVPO	Adenylate kinase 2, mitochondrial OS=Cavia porcellus GN=AK2 PE=3 SV=1	CAVPO	2
1781	5.25	5.85	32.23	18.48	12.56	tr|H0W5F3|H0W5F3_CAVPO	Perilipin OS=Cavia porcellus GN=PLIN3 PE=3 SV=1	CAVPO	4
1782	5.24	5.29	7.519	7.519	7.519	tr|H0VAH5|H0VAH5_CAVPO	Uncharacterized protein OS=Cavia porcellus GN=RRAGC PE=4 SV=1	CAVPO	3
1783	5.23	5.33	25.84	22.47	22.47	tr|H0VY68|H0VY68_CAVPO	Uncharacterized protein OS=Cavia porcellus GN=PRAF2 PE=4 SV=1	CAVPO	3
1784	5.22	5.8	26.78	26.78	20.77	tr|H0VWB2|H0VWB2_CAVPO	Uncharacterized protein (Fragment) OS=Cavia porcellus GN=ARL1 PE=3 SV=1	CAVPO	4
1785	5.2	5.38	28.1	21.9	12.75	tr|H0W5X1|H0W5X1_CAVPO	Uncharacterized protein (Fragment) OS=Cavia porcellus GN=PPTC7 PE=4 SV=1	CAVPO	3
1786	5.19	5.25	9.031	6.404	6.404	tr|H0VKP2|H0VKP2_CAVPO	Uncharacterized protein (Fragment) OS=Cavia porcellus GN=Nlgn4x PE=4 SV=1	CAVPO	3
1787	5.18	13.7	8.579	5.549	4.054	tr|H0UTV0|H0UTV0_CAVPO	Uncharacterized protein OS=Cavia porcellus GN=TLN1 PE=4 SV=1	CAVPO	8
1788	5.18	5.25	31.21	30.64	30.64	tr|H0V8V2|H0V8V2_CAVPO	Uncharacterized protein OS=Cavia porcellus GN=SSR4 PE=4 SV=1	CAVPO	4
1789	5.17	5.37	42.03	42.03	42.03	tr|H0WB58|H0WB58_CAVPO	Guanine nucleotide-binding protein subunit gamma OS=Cavia porcellus GN=GNGT2 PE=3 SV=1	CAVPO	6
1790	5.16	5.25	20.36	14.59	9.726	tr|H0UWH9|H0UWH9_CAVPO	Uncharacterized protein OS=Cavia porcellus GN=RNF2 PE=4 SV=1	CAVPO	3
1791	5.16	7.54	7.036	3.247	1.178	tr|H0V9L0|H0V9L0_CAVPO	Uncharacterized protein OS=Cavia porcellus GN=HTT PE=4 SV=1	CAVPO	3

1792	5.15	5.27	33.77	29.22	25.32	tr|H0VBH5|H0VBH5_CAVPO	Actin-related protein 2/3 complex subunit 5 OS=Cavia porcellus GN=ARPC5 PE=3 SV=1	CAVPO	3
1793	5.15	5.21	29.59	24.49	24.49	tr|H0VDN3|H0VDN3_CAVPO	Uncharacterized protein OS=Cavia porcellus GN=RER1 PE=4 SV=1	CAVPO	3
1794	5.15	5.23	44.44	34.26	34.26	tr|H0VMN3|H0VMN3_CAVPO	Uncharacterized protein OS=Cavia porcellus GN=CISD1 PE=4 SV=1	CAVPO	3
1795	5.15	5.37	38.51	22.99	22.99	tr|H0VUA5|H0VUA5_CAVPO	Uncharacterized protein OS=Cavia porcellus GN=Eef1e1 PE=4 SV=1	CAVPO	4
1796	5.15	5.2	11.01	11.01	11.01	tr|H0WDV6|H0WDV6_CAVPO	Uncharacterized protein OS=Cavia porcellus GN=Ube2j1 PE=4 SV=1	CAVPO	3
1797	5.13	5.22	11.17	8.576	5.178	tr|H0V731|H0V731_CAVPO	Uncharacterized protein OS=Cavia porcellus GN=NXF1 PE=4 SV=1	CAVPO	3
1798	5.13	5.92	31.27	22.08	13.9	tr|H0VRF9|H0VRF9_CAVPO	Uncharacterized protein OS=Cavia porcellus GN=Pcbp4 PE=4 SV=1	CAVPO	4
1799	5.12	5.38	12.18	8.576	8.576	tr|H0UYA3|H0UYA3_CAVPO	Uncharacterized protein (Fragment) OS=Cavia porcellus GN=TM9SF3 PE=4 SV=1	CAVPO	4
1800	5.11	5.63	17.07	10.98	6.739	tr|H0VHV2|H0VHV2_CAVPO	Uncharacterized protein OS=Cavia porcellus GN=CASK PE=4 SV=1	CAVPO	5
1801	5.11	5.2	6.263	3.771	1.886	tr|H0VY94|H0VY94_CAVPO	Uncharacterized protein OS=Cavia porcellus GN=COL18A1 PE=4 SV=1	CAVPO	3
1802	5.11	5.24	12.06	12.06	12.06	tr|H0W479|H0W479_CAVPO	Uncharacterized protein OS=Cavia porcellus GN=RGR PE=4 SV=1	CAVPO	3
1803	5.1	5.79	18.57	14.14	7.384	tr|H0UW00|H0UW00_CAVPO	Coronin OS=Cavia porcellus GN=CORO1C PE=3 SV=1	CAVPO	4
1804	5.1	5.31	9.78	7.579	4.89	tr|H0UZW3|H0UZW3_CAVPO	Uncharacterized protein OS=Cavia porcellus GN=PHC2 PE=4 SV=1	CAVPO	3
1805	5.1	5.17	18.81	12.62	9.406	tr|H0VFK7|H0VFK7_CAVPO	Uncharacterized protein (Fragment) OS=Cavia porcellus GN=ACADL PE=3 SV=1	CAVPO	3

1806	5.1	5.16	21.08	21.08	17.04	tr|H0W4B4|H0W4B4_CAVPO	Uncharacterized protein OS=Cavia porcellus GN=Psmd9 PE=4 SV=1	CAVPO	3
1807	5.09	5.25	29.22	28.31	28.31	tr|H0V0Y0|H0V0Y0_CAVPO	Uncharacterized protein OS=Cavia porcellus GN=CHMP5 PE=4 SV=1	CAVPO	5
1808	5.09	5.25	50	48.68	48.68	tr|H0VR28|H0VR28_CAVPO	Uncharacterized protein (Fragment) OS=Cavia porcellus GN=COX6C PE=4 SV=1	CAVPO	5
1809	5.08	5.13	25.21	25.21	25.21	tr|H0VJS8|H0VJS8_CAVPO	Uncharacterized protein (Fragment) OS=Cavia porcellus GN=RPS20 PE=3 SV=1	CAVPO	3
1810	5.07	5.18	22.41	18.46	7.884	tr|H0UUW0|H0UUW0_CAVPO	Glycylpeptide N-tetradecanoyltransferase OS=Cavia porcellus GN=NMT1 PE=3 SV=1	CAVPO	3
1811	5.06	5.26	10.51	6.226	3.502	tr|H0V3K9|H0V3K9_CAVPO	Uncharacterized protein OS=Cavia porcellus GN=LLGL2 PE=4 SV=1	CAVPO	3
1812	5.04	5.11	21.1	17.43	11.62	tr|H0VK95|H0VK95_CAVPO	Nucleoporin NUP53 OS=Cavia porcellus GN=NUP35 PE=3 SV=1	CAVPO	3
1813	5.03	5.41	25.29	22.41	22.41	tr|H0V931|H0V931_CAVPO	Uncharacterized protein OS=Cavia porcellus GN=UBE2E3 PE=3 SV=1	CAVPO	4
1814	5.02	5.33	23.48	14.6	9.982	tr|H0VB21|H0VB21_CAVPO	Uncharacterized protein OS=Cavia porcellus GN=SNX27 PE=4 SV=1	CAVPO	4
1815	5.02	5.09	86.67	86.67	86.67	tr|H0VUI8|H0VUI8_CAVPO	Uncharacterized protein (Fragment) OS=Cavia porcellus GN=HINT1 PE=4 SV=1	CAVPO	4
1816	5.01	5.35	7.818	4.351	3.216	tr|H0VRE8|H0VRE8_CAVPO	Uncharacterized protein OS=Cavia porcellus GN=SCRIB PE=4 SV=1	CAVPO	4
1817	5.01	5.11	14.15	14.15	10.38	tr|H0W7P0|H0W7P0_CAVPO	Uncharacterized protein OS=Cavia porcellus GN=RDH5 PE=3 SV=1	CAVPO	3
1818	5	5.29	11.95	6.501	4.876	tr|H0UX90|H0UX90_CAVPO	Uncharacterized protein OS=Cavia porcellus GN=UBA6 PE=4 SV=1	CAVPO	6
1819	5	5.1	10.42	6.713	6.713	tr|H0V8Q2|H0V8Q2_CAVPO	Uncharacterized protein (Fragment) OS=Cavia porcellus GN=KRT14 PE=3 SV=1	CAVPO	3

1820	5	5.29	15.56	6.358	5.385	tr|H0VJG7|H0VJG7_CAVPO	Uncharacterized protein OS=Cavia porcellus GN=PDS5A PE=4 SV=1	CAVPO	5
1821	5	18.7	50.53	40.43	33.78	tr|H0VSV0|H0VSV0_CAVPO	Uncharacterized protein OS=Cavia porcellus GN=ACTR1B PE=3 SV=1	CAVPO	11
1822	4.99	5.07	38.37	29.65	25	tr|H0UVK3|H0UVK3_CAVPO	Uncharacterized protein OS=Cavia porcellus GN=POLR2G PE=4 SV=1	CAVPO	3
1823	4.99	5.1	15.2	7.281	7.281	tr|H0V3B3|H0V3B3_CAVPO	Uncharacterized protein OS=Cavia porcellus GN=IGSF21 PE=4 SV=1	CAVPO	3
1824	4.99	5.56	12.94	11.06	9.186	tr|H0V446|H0V446_CAVPO	Uncharacterized protein (Fragment) OS=Cavia porcellus GN=SEC61A1 PE=3 SV=1	CAVPO	4
1825	4.97	5.06	22.71	20.35	12.39	tr|H0V7P8|H0V7P8_CAVPO	Annexin OS=Cavia porcellus GN=ANXA2 PE=3 SV=1	CAVPO	3
1826	4.97	5.25	14.6	12.13	5.569	tr|H0W8V2|H0W8V2_CAVPO	Uncharacterized protein OS=Cavia porcellus GN=IWS1 PE=4 SV=1	CAVPO	3
1827	4.96	5.1	21.78	11.94	7.494	tr|H0V866|H0V866_CAVPO	Uncharacterized protein (Fragment) OS=Cavia porcellus GN=STK24 PE=4 SV=1	CAVPO	3
1828	4.96	5.13	30.52	29.87	29.87	tr|H0VAU2|H0VAU2_CAVPO	Uncharacterized protein OS=Cavia porcellus GN=KCTD12 PE=4 SV=1	CAVPO	4
1829	4.95	5.02	29.09	25.82	22.18	tr|H0UTM8|H0UTM8_CAVPO	Uncharacterized protein OS=Cavia porcellus GN=COPS7A PE=4 SV=1	CAVPO	4
1830	4.95	5.01	13.23	10.58	10.58	tr|H0VI39|H0VI39_CAVPO	Uncharacterized protein OS=Cavia porcellus GN=REEP5 PE=4 SV=1	CAVPO	3
1831	4.95	5.04	25.49	15.2	12.25	tr|H0VZ51|H0VZ51_CAVPO	Uncharacterized protein OS=Cavia porcellus GN=RPL13A PE=3 SV=1	CAVPO	3
1832	4.95	5.14	39.57	24.06	12.83	tr|H0W154|H0W154_CAVPO	Uncharacterized protein OS=Cavia porcellus GN=C17orf62 PE=4 SV=1	CAVPO	3
1833	4.94	5.01	27.04	27.04	20.75	tr|H0UZQ0|H0UZQ0_CAVPO	Uncharacterized protein (Fragment) OS=Cavia porcellus GN=NDUFB11 PE=4 SV=1	CAVPO	3

1834	4.94	5.35	16.96	10.24	7.93	tr|H0V7T5|H0V7T5_CAVPO	Uncharacterized protein (Fragment) OS=Cavia porcellus GN=ALDH1L1 PE=3 SV=1	CAVPO	5
1835	4.93	5.01	12.58	10.41	7.809	tr|H0V7U7|H0V7U7_CAVPO	Uncharacterized protein OS=Cavia porcellus GN=RGS7 PE=4 SV=1	CAVPO	3
1836	4.93	4.99	27.23	24.11	24.11	tr|H0W756|H0W756_CAVPO	Uncharacterized protein (Fragment) OS=Cavia porcellus GN=FAHD1 PE=4 SV=1	CAVPO	3
1837	4.92	11.3	34.43	34.43	32.79	tr|H0VIX9|H0VIX9_CAVPO	Uncharacterized protein (Fragment) OS=Cavia porcellus GN=YBX3 PE=4 SV=1	CAVPO	7
1838	4.92	4.97	31.87	20	20	tr|H0VJC2|H0VJC2_CAVPO	Uncharacterized protein (Fragment) OS=Cavia porcellus GN=C1orf123 PE=4 SV=1	CAVPO	3
1839	4.92	6.78	20.39	15.92	10.12	tr|H0W828|H0W828_CAVPO	Uncharacterized protein OS=Cavia porcellus GN=FXR2 PE=4 SV=1	CAVPO	5
1840	4.91	5.14	26.74	17.67	10.93	tr|H0UVR9|H0UVR9_CAVPO	Uncharacterized protein OS=Cavia porcellus GN=Eif5 PE=4 SV=1	CAVPO	4
1841	4.91	4.96	26.67	26.67	26.67	tr|H0V9W8|H0V9W8_CAVPO	Uncharacterized protein OS=Cavia porcellus GN=POLR2H PE=4 SV=1	CAVPO	3
1842	4.91	8.3	7.456	3.651	2.537	tr|H0VH35|H0VH35_CAVPO	Uncharacterized protein OS=Cavia porcellus GN=FLNB PE=4 SV=1	CAVPO	6
1843	4.91	5.04	39.52	28.14	22.75	tr|H0VXL0|H0VXL0_CAVPO	Uncharacterized protein OS=Cavia porcellus GN=CGGBP1 PE=4 SV=1	CAVPO	3
1844	4.9	5.03	33.24	22.79	10.72	tr|H0UTA9|H0UTA9_CAVPO	Uncharacterized protein OS=Cavia porcellus GN=MECR PE=4 SV=1	CAVPO	2
1845	4.9	5.03	22.34	12.71	12.71	tr|H0V6R0|H0V6R0_CAVPO	Uncharacterized protein OS=Cavia porcellus GN=NMRAL1 PE=4 SV=1	CAVPO	3
1846	4.89	5.05	34.98	31.39	31.39	tr|H0V7J9|H0V7J9_CAVPO	Uncharacterized protein OS=Cavia porcellus GN=SMNDC1 PE=4 SV=1	CAVPO	4
1847	4.89	5.08	27.54	24.06	19.25	tr|H0VRC9|H0VRC9_CAVPO	Uncharacterized protein OS=Cavia porcellus GN=PEX14 PE=4 SV=1	CAVPO	3

1848	4.88	5.16	22.01	18.53	10.04	tr|H0VUP1|H0VUP1_CAVPO	Uncharacterized protein OS=Cavia porcellus GN=DNAJC9 PE=4 SV=1	CAVPO	3
1849	4.88	4.94	17.06	17.06	17.06	tr|H0W2S4|H0W2S4_CAVPO	Uncharacterized protein OS=Cavia porcellus GN=REEP6 PE=4 SV=1	CAVPO	3
1850	4.87	6.41	24.62	19.08	19.08	tr|H0UTP9|H0UTP9_CAVPO	Uncharacterized protein (Fragment) OS=Cavia porcellus GN=KHDRBS3 PE=4 SV=1	CAVPO	5
1851	4.87	5.04	22.89	13.81	10.1	tr|H0VJ14|H0VJ14_CAVPO	Uncharacterized protein (Fragment) OS=Cavia porcellus GN=UBXN4 PE=4 SV=1	CAVPO	3
1852	4.86	5.09	15.66	14.41	14.41	tr|H0V3E8|H0V3E8_CAVPO	Uncharacterized protein OS=Cavia porcellus GN=PI4K2A PE=4 SV=1	CAVPO	4
1853	4.86	4.98	17.6	14	14	tr|H0VPJ6|H0VPJ6_CAVPO	Uncharacterized protein OS=Cavia porcellus GN=SBDS PE=4 SV=1	CAVPO	3
1854	4.85	5.06	25.05	13.32	11.13	tr|H0V1G8|H0V1G8_CAVPO	Uncharacterized protein OS=Cavia porcellus GN=SRP54 PE=3 SV=1	CAVPO	4
1855	4.85	7.29	18.27	11.96	11.96	tr|H0VES7|H0VES7_CAVPO	Uncharacterized protein OS=Cavia porcellus GN=PDCL PE=4 SV=1	CAVPO	5
1856	4.85	5.02	24.87	24.87	15.28	tr|H0VFQ1|H0VFQ1_CAVPO	Uncharacterized protein (Fragment) OS=Cavia porcellus GN=UBA5 PE=4 SV=1	CAVPO	3
1857	4.84	4.9	11.2	7.662	7.662	tr|H0UTB2|H0UTB2_CAVPO	Uncharacterized protein OS=Cavia porcellus GN=NUP54 PE=4 SV=1	CAVPO	3
1858	4.84	4.89	50.47	50.47	50.47	tr|H0VDP4|H0VDP4_CAVPO	Uncharacterized protein OS=Cavia porcellus GN=C19orf25 PE=4 SV=1	CAVPO	3
1859	4.84	5.33	5.998	4.405	3.28	tr|H0VN42|H0VN42_CAVPO	Uncharacterized protein OS=Cavia porcellus GN=UBE4A PE=4 SV=1	CAVPO	3
1860	4.84	5.01	29.6	20.58	13.72	tr|H0VQW2|H0VQW2_CAVPO	Uncharacterized protein OS=Cavia porcellus GN=MLEC PE=4 SV=1	CAVPO	3
1861	4.82	5.15	43.13	34.6	15.64	tr|H0VCT1|H0VCT1_CAVPO	Uncharacterized protein OS=Cavia porcellus GN=PITHD1 PE=4 SV=1	CAVPO	3

1862	4.82	5.18	10.03	10.03	7.326	tr|H0VFS6|H0VFS6_CAVPO	Uncharacterized protein OS=Cavia porcellus GN=QRICH1 PE=4 SV=1	CAVPO	4
1863	4.81	4.9	70.67	62.67	56	tr|H0UVY0|H0UVY0_CAVPO	Guanine nucleotide-binding protein subunit gamma (Fragment) OS=Cavia porcellus GN=GNG2 PE=3 SV=1	CAVPO	6
1864	4.8	4.9	29.44	16.5	6.345	tr|H0VIX1|H0VIX1_CAVPO	Uncharacterized protein OS=Cavia porcellus GN=SIRT2 PE=4 SV=1	CAVPO	2
1865	4.8	4.87	15.79	13.16	10.53	tr|H0VSK5|H0VSK5_CAVPO	Uncharacterized protein OS=Cavia porcellus GN=DCAF7 PE=4 SV=1	CAVPO	3
1866	4.8	17.5	46.02	42.61	42.61	tr|H0VZG7|H0VZG7_CAVPO	Uncharacterized protein (Fragment) OS=Cavia porcellus GN=ANP32B PE=4 SV=1	CAVPO	14
1867	4.79	4.9	17.72	12.22	4.277	tr|H0VT64|H0VT64_CAVPO	Uncharacterized protein OS=Cavia porcellus GN=Rabgef1 PE=4 SV=1	CAVPO	2
1868	4.78	4.87	15.71	15.24	6.905	tr|H0UYM8|H0UYM8_CAVPO	Uncharacterized protein OS=Cavia porcellus GN=GMPPA PE=4 SV=1	CAVPO	2
1869	4.77	4.91	13.03	7.563	7.563	tr|H0VTF9|H0VTF9_CAVPO	Uncharacterized protein OS=Cavia porcellus GN=CPE PE=4 SV=1	CAVPO	4
1870	4.77	4.82	18.82	18.82	18.82	tr|Q9JHM1|Q9JHM1_CAVPO	Glutathione peroxidase OS=Cavia porcellus GN=GP-GPx4 PE=2 SV=2	CAVPO	3
1871	4.76	4.88	10.38	8.197	8.197	tr|H0V8T1|H0V8T1_CAVPO	Uncharacterized protein (Fragment) OS=Cavia porcellus GN=UBFD1 PE=4 SV=1	CAVPO	3
1872	4.76	4.97	18.26	12.58	10.55	tr|H0W0L8|H0W0L8_CAVPO	Uncharacterized protein OS=Cavia porcellus GN=DNAJC7 PE=4 SV=1	CAVPO	4
1873	4.76	5	26.63	6.699	6.699	tr|H0WDC2|H0WDC2_CAVPO	Uncharacterized protein OS=Cavia porcellus GN=KBTBD11 PE=4 SV=1	CAVPO	4
1874	4.75	4.89	40.91	26.36	26.36	tr|H0VLW3|H0VLW3_CAVPO	Uncharacterized protein OS=Cavia porcellus GN=PHF5A PE=4 SV=1	CAVPO	3
1875	4.75	4.91	25.37	14.16	9.735	tr|H0VR09|H0VR09_CAVPO	Uncharacterized protein OS=Cavia porcellus	CAVPO	3

							GN=DNAJB1 PE=4 SV=1		
1876	4.74	4.9	47.62	34.69	22.45	tr|H0VNH9|H0VNH9_CAVPO	Uncharacterized protein OS=Cavia porcellus GN=NUDT2 PE=3 SV=1	CAVPO	3
1877	4.74	4.84	15.38	8.524	2.703	tr|H0VZS6|H0VZS6_CAVPO	Uncharacterized protein (Fragment) OS=Cavia porcellus GN=CTNND2 PE=4 SV=1	CAVPO	2
1878	4.72	4.84	13.02	8.58	8.58	tr|H0V4D3|H0V4D3_CAVPO	Uncharacterized protein (Fragment) OS=Cavia porcellus GN=NFYC PE=4 SV=1	CAVPO	3
1879	4.72	4.85	9.158	9.158	9.158	tr|H0V9F8|H0V9F8_CAVPO	Uncharacterized protein OS=Cavia porcellus GN=PEF1 PE=4 SV=1	CAVPO	3
1880	4.72	4.91	16.09	13.86	7.178	tr|H0VB53|H0VB53_CAVPO	Uncharacterized protein OS=Cavia porcellus GN=SNX5 PE=4 SV=1	CAVPO	3
1881	4.71	5.03	22.99	22.99	13.37	tr|H0V2L2|H0V2L2_CAVPO	Uncharacterized protein (Fragment) OS=Cavia porcellus GN=APOO PE=4 SV=1	CAVPO	2
1882	4.71	4.77	29.14	29.14	22.86	tr|H0W524|H0W524_CAVPO	Uncharacterized protein OS=Cavia porcellus GN=C19orf43 PE=4 SV=1	CAVPO	3
1883	4.7	4.97	10.37	6.786	3.841	tr|H0V1P7|H0V1P7_CAVPO	Uncharacterized protein (Fragment) OS=Cavia porcellus GN=SMEK1 PE=4 SV=1	CAVPO	3
1884	4.68	4.73	37.66	22.08	22.08	tr|H0UVJ3|H0UVJ3_CAVPO	Uncharacterized protein OS=Cavia porcellus GN=ISCA2 PE=4 SV=1	CAVPO	3
1885	4.68	4.85	30.17	30.17	30.17	tr|H0UWZ8|H0UWZ8_CAVPO	Uncharacterized protein OS=Cavia porcellus GN=HDDC3 PE=4 SV=1	CAVPO	4
1886	4.67	4.88	28.88	21.88	10.33	tr|H0UUF2|H0UUF2_CAVPO	Uncharacterized protein OS=Cavia porcellus GN=UCHL5 PE=4 SV=1	CAVPO	3
1887	4.67	4.82	40	39.13	29.57	tr|H0UXL5|H0UXL5_CAVPO	Uncharacterized protein (Fragment) OS=Cavia porcellus GN=Ndufa7 PE=4 SV=1	CAVPO	3
1888	4.67	4.74	10.83	9.32	2.519	tr|H0V902|H0V902_CAVPO	Uncharacterized protein OS=Cavia porcellus GN=UFL1 PE=4 SV=1	CAVPO	2

1889	4.67	4.89	9.047	4.455	2.262	tr|H0VKV4|H0VKV4_CAVPO	Uncharacterized protein (Fragment) OS=Cavia porcellus GN=DIP2B PE=4 SV=1	CAVPO	3
1890	4.67	4.84	17.08	5.282	5.282	tr|H0VQY1|H0VQY1_CAVPO	Uncharacterized protein (Fragment) OS=Cavia porcellus GN=RABGGTA PE=4 SV=1	CAVPO	3
1891	4.66	5.01	15.13	7.968	4.734	tr|H0VN55|H0VN55_CAVPO	Uncharacterized protein OS=Cavia porcellus GN=NAA15 PE=4 SV=1	CAVPO	4
1892	4.66	4.8	15.68	11.35	8.468	tr|H0VSQ3|H0VSQ3_CAVPO	Uncharacterized protein OS=Cavia porcellus GN=PIGS PE=4 SV=1	CAVPO	3
1893	4.65	5.15	23.59	13.29	6.202	tr|H0UTF9|H0UTF9_CAVPO	Uncharacterized protein OS=Cavia porcellus GN=MGEA5 PE=4 SV=1	CAVPO	4
1894	4.65	9.71	14.46	11.42	11.42	tr|H0WE04|H0WE04_CAVPO	Uncharacterized protein OS=Cavia porcellus GN=PICALM PE=4 SV=1	CAVPO	6
1895	4.64	4.77	20	14.23	14.23	tr|H0WCT9|H0WCT9_CAVPO	Uncharacterized protein OS=Cavia porcellus GN=HAGH PE=3 SV=1	CAVPO	3
1896	4.63	4.7	28.8	14.24	11.39	tr|H0V3V8|H0V3V8_CAVPO	Uncharacterized protein (Fragment) OS=Cavia porcellus GN=DECR1 PE=4 SV=1	CAVPO	3
1897	4.62	4.75	28.74	23.95	16.17	tr|H0W2P9|H0W2P9_CAVPO	Ubiquitin-fold modifier-conjugating enzyme 1 OS=Cavia porcellus GN=UFC1 PE=3 SV=1	CAVPO	3
1898	4.61	5.08	20	13.44	13.44	tr|H0VI22|H0VI22_CAVPO	Uncharacterized protein OS=Cavia porcellus GN=GPM6B PE=4 SV=1	CAVPO	9
1899	4.59	4.63	33.33	33.33	33.33	tr|H0VE12|H0VE12_CAVPO	NADH dehydrogenase [ubiquinone] 1 alpha subcomplex subunit 2 OS=Cavia porcellus GN=NDUFA2 PE=3 SV=1	CAVPO	3
1900	4.59	4.71	28.55	13.16	4.786	tr|H0VPE1|H0VPE1_CAVPO	Uncharacterized protein OS=Cavia porcellus GN=GAD2 PE=3 SV=1	CAVPO	2
1901	4.58	4.63	24.55	19.7	7.576	tr|H0V1C6|H0V1C6_CAVPO	Uncharacterized protein OS=Cavia porcellus GN=RCN3 PE=4 SV=1	CAVPO	2
1902	4.57	4.83	17.16	12.38	5.116	tr|H0UXH2|H0UXH2_CAVPO	Uncharacterized protein (Fragment) OS=Cavia	CAVPO	3

							porcellus GN=PDE1C PE=4 SV=1		
1903	4.57	4.99	44	32	18.29	tr|H0VBM9|H0VBM9_CAVPO	Uncharacterized protein OS=Cavia porcellus GN=ARF6 PE=3 SV=1	CAVPO	5
1904	4.57	43.6	74.85	72.42	72.42	tr|Q0QEP1|Q0QEP1_CAVPO	ATP synthase subunit beta (Fragment) OS=Cavia porcellus GN=ATP5B PE=2 SV=1	CAVPO	49
1905	4.56	12.8	37.31	37.31	32.64	tr|H0W5I4|H0W5I4_CAVPO	Uncharacterized protein OS=Cavia porcellus GN=NCALD PE=4 SV=1	CAVPO	8
1906	4.55	4.71	17.42	9.343	4.293	tr|H0VNI0|H0VNI0_CAVPO	Ribonucleoside-diphosphate reductase OS=Cavia porcellus GN=RRM1 PE=3 SV=1	CAVPO	3
1907	4.55	19.5	63.41	63.41	63.41	tr|H0VTD0|H0VTD0_CAVPO	Uncharacterized protein OS=Cavia porcellus GN=RAB1A PE=3 SV=1	CAVPO	15
1908	4.54	6.67	16.35	13.65	7.778	tr|H0UTG7|H0UTG7_CAVPO	Uncharacterized protein (Fragment) OS=Cavia porcellus GN=KLC4 PE=4 SV=1	CAVPO	3
1909	4.52	4.71	21.44	9.897	7.423	tr|H0WBT1|H0WBT1_CAVPO	Uncharacterized protein OS=Cavia porcellus GN=NAE1 PE=4 SV=1	CAVPO	3
1910	4.52	4.63	33.91	33.91	33.91	tr|H0WDD1|H0WDD1_CAVPO	Uncharacterized protein (Fragment) OS=Cavia porcellus GN=Rps26 PE=4 SV=1	CAVPO	3
1911	4.51	4.63	19.8	11.22	11.22	tr|H0W5B3|H0W5B3_CAVPO	Uncharacterized protein OS=Cavia porcellus GN=STUB1 PE=4 SV=1	CAVPO	3
1912	4.49	4.53	23.32	19.69	19.69	tr|H0V2X3|H0V2X3_CAVPO	Uncharacterized protein OS=Cavia porcellus GN=CSRP2 PE=4 SV=1	CAVPO	3
1913	4.49	4.6	13.33	7.619	7.619	tr|H0V8E3|H0V8E3_CAVPO	Uncharacterized protein OS=Cavia porcellus GN=CRELD1 PE=4 SV=1	CAVPO	3
1914	4.49	4.74	24.59	22.93	17.96	tr|H0WB33|H0WB33_CAVPO	Uroporphyrinogen decarboxylase (Fragment) OS=Cavia porcellus GN=UROD PE=3 SV=1	CAVPO	4
1915	4.48	4.53	17.7	15.04	7.375	tr|H0UTY8|H0UTY8_CAVPO	Uncharacterized protein (Fragment) OS=Cavia porcellus GN=TMX4 PE=4 SV=1	CAVPO	2

1916	4.48	7.07	52.78	42.13	28.24	tr|H0VX73|H0VX73_CAVPO	Uncharacterized protein OS=Cavia porcellus GN=Rab4b PE=3 SV=1	CAVPO	7
1917	4.47	4.73	13.55	11.16	6.972	tr|H0V9C5|H0V9C5_CAVPO	Uncharacterized protein (Fragment) OS=Cavia porcellus GN=CSAD PE=3 SV=1	CAVPO	3
1918	4.47	25.7	18.02	14.27	10.93	tr|H0VM29|H0VM29_CAVPO	Uncharacterized protein OS=Cavia porcellus GN=CYFIP1 PE=4 SV=1	CAVPO	12
1919	4.47	4.59	47.06	28.43	28.43	tr|H0W597|H0W597_CAVPO	Uncharacterized protein OS=Cavia porcellus GN=Basp1 PE=4 SV=1	CAVPO	5
1920	4.46	5.34	35.9	35.9	35.9	tr|H0UUY8|H0UUY8_CAVPO	Uncharacterized protein OS=Cavia porcellus GN=GLRX5 PE=4 SV=1	CAVPO	4
1921	4.46	4.57	11.5	11.5	8	tr|H0VCX2|H0VCX2_CAVPO	Uncharacterized protein (Fragment) OS=Cavia porcellus GN=Crlf3 PE=4 SV=1	CAVPO	3
1922	4.46	4.51	18.18	14.66	12.61	tr|H0VHN6|H0VHN6_CAVPO	Uncharacterized protein OS=Cavia porcellus GN=CREB1 PE=4 SV=1	CAVPO	2
1923	4.45	4.5	15.58	8.846	5.769	tr|H0V9E0|H0V9E0_CAVPO	Uncharacterized protein OS=Cavia porcellus GN=Ppil2 PE=4 SV=1	CAVPO	2
1924	4.44	4.62	13.91	10.6	3.863	tr|H0VNV1|H0VNV1_CAVPO	Uncharacterized protein OS=Cavia porcellus GN=PRPF40A PE=4 SV=1	CAVPO	3
1925	4.43	4.49	4.3	1.906	1.197	tr|H0V463|H0V463_CAVPO	Uncharacterized protein OS=Cavia porcellus GN=Dido1 PE=4 SV=1	CAVPO	2
1926	4.43	4.63	69.47	54.74	38.95	tr|H0VEJ0|H0VEJ0_CAVPO	Uncharacterized protein OS=Cavia porcellus GN=Timm13 PE=4 SV=1	CAVPO	3
1927	4.43	4.48	17.32	14.57	8.268	tr|H0VG08|H0VG08_CAVPO	Uncharacterized protein (Fragment) OS=Cavia porcellus GN=TMEM33 PE=4 SV=1	CAVPO	2
1928	4.42	4.74	9.387	5.887	2.625	tr|H0VN77|H0VN77_CAVPO	Uncharacterized protein OS=Cavia porcellus GN=STAG1 PE=4 SV=1	CAVPO	3
1929	4.41	4.46	12.86	11.75	4.435	tr|H0VD93|H0VD93_CAVPO	Tubulin gamma chain OS=Cavia porcellus GN=TUBG1 PE=3 SV=1	CAVPO	2

1930	4.41	4.45	19.26	19.26	16.39	tr∣H0VKN9∣H0VKN9_CAVPO	Uncharacterized protein OS=Cavia porcellus GN=TBCB PE=4 SV=1	CAVPO	3
1931	4.41	4.45	13.77	13.77	10.93	tr∣H0VL62∣H0VL62_CAVPO	Uncharacterized protein OS=Cavia porcellus GN=MLF2 PE=4 SV=1	CAVPO	2
1932	4.4	9.84	19.24	14.74	10.35	tr∣H0V966∣H0V966_CAVPO	Uncharacterized protein (Fragment) OS=Cavia porcellus GN=TNPO2 PE=4 SV=1	CAVPO	6
1933	4.39	4.99	32.44	23.08	13.71	tr∣H0UYF1∣H0UYF1_CAVPO	Uncharacterized protein (Fragment) OS=Cavia porcellus GN=BPHL PE=4 SV=1	CAVPO	2
1934	4.39	4.53	14.01	10.75	5.95	tr∣H0V3K6∣H0V3K6_CAVPO	Importin subunit alpha OS=Cavia porcellus GN=KPNA3 PE=3 SV=1	CAVPO	4
1935	4.39	7.08	22.55	21.7	19.15	tr∣H0V3W8∣H0V3W8_CAVPO	Uncharacterized protein OS=Cavia porcellus GN=TMED9 PE=3 SV=1	CAVPO	4
1936	4.39	4.53	38.73	38.73	14.79	tr∣H0WAE0∣H0WAE0_CAVPO	Uncharacterized protein (Fragment) OS=Cavia porcellus GN=FAIM PE=4 SV=1	CAVPO	2
1937	4.38	4.71	36.59	25.99	5.198	tr∣H0V8H2∣H0V8H2_CAVPO	Uncharacterized protein OS=Cavia porcellus GN=Ddx19a PE=4 SV=1	CAVPO	2
1938	4.38	4.59	34.12	21.18	21.18	tr∣H0VGE1∣H0VGE1_CAVPO	Uncharacterized protein OS=Cavia porcellus GN=COX4I2 PE=4 SV=1	CAVPO	3
1939	4.38	4.76	15.32	10.99	7.387	tr∣H0VS36∣H0VS36_CAVPO	Uncharacterized protein (Fragment) OS=Cavia porcellus GN=ASPSCR1 PE=4 SV=1	CAVPO	3
1940	4.38	4.49	11.6	11.6	11.6	tr∣Q9TEH1∣Q9TEH1_CAVPO	NADH-ubiquinone oxidoreductase chain 1 OS=Cavia porcellus GN=NADH1 PE=3 SV=1	CAVPO	4
1941	4.37	4.55	57.99	21.92	16.89	tr∣H0VNE8∣H0VNE8_CAVPO	Uncharacterized protein OS=Cavia porcellus GN=TRAPPC4 PE=4 SV=1	CAVPO	3
1942	4.37	4.49	37.86	37.86	37.86	tr∣H0VVE0∣H0VVE0_CAVPO	Uncharacterized protein OS=Cavia porcellus GN=Ndufa11 PE=4 SV=1	CAVPO	3
1943	4.36	5.68	23.52	12.02	6.969	tr∣H0VM19∣H0VM19_CAVPO	Uncharacterized protein (Fragment) OS=Cavia porcellus GN=SEPT9 PE=3 SV=1	CAVPO	5

1944	4.35	4.63	24.34	20.58	10.84	tr|H0UXX3|H0UXX3_CAVPO	Uncharacterized protein OS=Cavia porcellus GN=EIF2B3 PE=4 SV=1	CAVPO	3
1945	4.35	5.85	16.67	9.005	6.317	tr|H0VBS7|H0VBS7_CAVPO	Uncharacterized protein OS=Cavia porcellus GN=DCLK2 PE=4 SV=1	CAVPO	3
1946	4.35	4.52	5.605	4.557	1.152	tr|H0VGS4|H0VGS4_CAVPO	Uncharacterized protein (Fragment) OS=Cavia porcellus GN=ARID1A PE=4 SV=1	CAVPO	2
1947	4.34	4.48	17.65	17.65	8.497	tr|H0VCQ2|H0VCQ2_CAVPO	Uncharacterized protein OS=Cavia porcellus GN=SAP18 PE=4 SV=1	CAVPO	2
1948	4.34	8.52	27.42	21.37	18.15	tr|H0VP72|H0VP72_CAVPO	Uncharacterized protein OS=Cavia porcellus GN=TPM4 PE=3 SV=1	CAVPO	5
1949	4.33	4.36	61.63	39.53	39.53	tr|H0V1Y7|H0V1Y7_CAVPO	Uncharacterized protein OS=Cavia porcellus GN=Bola2b PE=3 SV=1	CAVPO	3
1950	4.33	4.4	19.16	13.36	4.745	tr|H0V5R8|H0V5R8_CAVPO	Uncharacterized protein (Fragment) OS=Cavia porcellus GN=STAU1 PE=4 SV=1	CAVPO	2
1951	4.33	4.41	16.72	13.78	8.211	tr|H0V7D4|H0V7D4_CAVPO	Uncharacterized protein OS=Cavia porcellus GN=ARFIP2 PE=4 SV=1	CAVPO	2
1952	4.33	4.49	26.07	24.07	17.19	tr|H0W6G0|H0W6G0_CAVPO	Uncharacterized protein OS=Cavia porcellus GN=SYAP1 PE=4 SV=1	CAVPO	4
1953	4.32	4.42	18.07	18.07	10.84	tr|H0V1L6|H0V1L6_CAVPO	40S ribosomal protein S6 OS=Cavia porcellus GN=Rps6 PE=3 SV=1	CAVPO	2
1954	4.32	4.45	17.2	17.2	17.2	tr|H0VHR8|H0VHR8_CAVPO	Uncharacterized protein OS=Cavia porcellus GN=NUP43 PE=4 SV=1	CAVPO	4
1955	4.32	4.4	58.43	41.57	41.57	tr|H0W3G1|H0W3G1_CAVPO	Uncharacterized protein OS=Cavia porcellus GN=TIMM9 PE=4 SV=1	CAVPO	3
1956	4.31	4.42	9.25	6.283	6.283	tr|H0VWM2|H0VWM2_CAVPO	Uncharacterized protein (Fragment) OS=Cavia porcellus GN=TTC39C PE=4 SV=1	CAVPO	3
1957	4.29	4.44	13.49	11.11	8.532	tr|H0VAT2|H0VAT2_CAVPO	Uncharacterized protein OS=Cavia porcellus GN=EIF2B4 PE=3 SV=1	CAVPO	3

1958	4.29	4.6	20.83	13.1	10.91	tr|H0VMD1|H0VMD1_CAVPO	Serine hydroxymethyltransferase OS=Cavia porcellus GN=SHMT2 PE=1 SV=1	CAVPO	4
1959	4.29	4.43	36.36	36.36	36.36	tr|H0VPK7|H0VPK7_CAVPO	Uncharacterized protein OS=Cavia porcellus GN=MAP1LC3A PE=3 SV=1	CAVPO	4
1960	4.28	4.58	7.95	4.184	2.762	tr|H0UUH9|H0UUH9_CAVPO	Uncharacterized protein OS=Cavia porcellus GN=DHX30 PE=4 SV=1	CAVPO	3
1961	4.28	4.42	8.147	6.439	4.862	tr|H0VFA5|H0VFA5_CAVPO	Uncharacterized protein (Fragment) OS=Cavia porcellus GN=PPP1R21 PE=4 SV=1	CAVPO	4
1962	4.28	4.3	31.71	26.83	16.59	tr|H0W0F1|H0W0F1_CAVPO	Uncharacterized protein OS=Cavia porcellus GN=Ppp1r2 PE=4 SV=1	CAVPO	2
1963	4.27	4.45	22.51	17.63	10.67	tr|H0V4C4|H0V4C4_CAVPO	Uncharacterized protein OS=Cavia porcellus GN=CSTF1 PE=4 SV=1	CAVPO	3
1964	4.27	4.3	20.31	19.79	15.63	tr|H0VKA8|H0VKA8_CAVPO	Uncharacterized protein OS=Cavia porcellus GN=SEC11C PE=4 SV=1	CAVPO	2
1965	4.26	4.37	15.14	15.14	15.14	tr|H0W4C8|H0W4C8_CAVPO	Uncharacterized protein OS=Cavia porcellus GN=ICMT PE=4 SV=1	CAVPO	3
1966	4.25	4.28	9.896	4.028	2.877	tr|H0V892|H0V892_CAVPO	Uncharacterized protein OS=Cavia porcellus GN=GTF3C3 PE=4 SV=1	CAVPO	2
1967	4.24	4.43	39.13	29.95	19.81	tr|H0UYC6|H0UYC6_CAVPO	Uncharacterized protein OS=Cavia porcellus GN=SPRYD4 PE=4 SV=1	CAVPO	3
1968	4.23	4.25	17.66	9.24	9.24	tr|H0VMB1|H0VMB1_CAVPO	Uncharacterized protein (Fragment) OS=Cavia porcellus GN=CTSA PE=4 SV=1	CAVPO	4
1969	4.23	4.26	37.56	37.56	22.44	tr|H0VN29|H0VN29_CAVPO	Uncharacterized protein (Fragment) OS=Cavia porcellus GN=TPD52L1 PE=4 SV=1	CAVPO	3
1970	4.22	4.53	22.86	12.76	6.286	tr|H0UWQ5|H0UWQ5_CAVPO	Uncharacterized protein (Fragment) OS=Cavia porcellus GN=MPP7 PE=4 SV=1	CAVPO	3
1971	4.21	4.23	30.11	22.16	17.05	tr|H0VHX6|H0VHX6_CAVPO	Uncharacterized protein OS=Cavia porcellus GN=TPPP3 PE=4 SV=1	CAVPO	2

1972	4.21	35.6	62.22	55.66	53.85	tr|H0VXL5|H0VXL5_CAVPO	Uncharacterized protein OS=Cavia porcellus PE=4 SV=1	CAVPO	67
1973	4.2	18.7	44.65	44.34	38.53	tr|H0VAY6|H0VAY6_CAVPO	Serine/threonine-protein phosphatase OS=Cavia porcellus GN=PPP1CB PE=3 SV=1	CAVPO	10
1974	4.18	4.35	15.14	11.78	6.49	tr|H0VHE3|H0VHE3_CAVPO	Phosphatidate cytidylyltransferase (Fragment) OS=Cavia porcellus GN=CDS1 PE=3 SV=1	CAVPO	2
1975	4.18	4.2	38	37	37	tr|H0VL18|H0VL18_CAVPO	SH3 domain-binding glutamic acid-rich-like protein OS=Cavia porcellus GN=SH3BGRL PE=3 SV=1	CAVPO	3
1976	4.16	4.26	17.7	12.03	6.529	tr|H0UWM0|H0UWM0_CAVPO	Uncharacterized protein (Fragment) OS=Cavia porcellus GN=PDE12 PE=4 SV=1	CAVPO	2
1977	4.16	4.22	35.75	31.09	19.69	tr|H0W5F0|H0W5F0_CAVPO	Uncharacterized protein (Fragment) OS=Cavia porcellus GN=Bid PE=4 SV=1	CAVPO	2
1978	4.15	4.32	18.91	14.9	14.9	tr|H0V0U3|H0V0U3_CAVPO	Methionine aminopeptidase (Fragment) OS=Cavia porcellus GN=METAP1 PE=3 SV=1	CAVPO	3
1979	4.15	4.33	6.327	6.327	6.327	tr|H0VEW5|H0VEW5_CAVPO	Uncharacterized protein OS=Cavia porcellus GN=PLD3 PE=4 SV=1	CAVPO	3
1980	4.14	4.16	29.12	15.33	11.88	tr|H0V401|H0V401_CAVPO	Uncharacterized protein OS=Cavia porcellus GN=EMC3 PE=4 SV=1	CAVPO	2
1981	4.14	4.29	27.06	23.43	10.89	tr|H0VI83|H0VI83_CAVPO	Uncharacterized protein (Fragment) OS=Cavia porcellus GN=PSMD8 PE=4 SV=1	CAVPO	3
1982	4.13	8.21	18.72	15.27	13.05	tr|H0VC01|H0VC01_CAVPO	Uncharacterized protein OS=Cavia porcellus GN=PDK3 PE=4 SV=1	CAVPO	4
1983	4.13	4.24	16.76	14.8	11.73	tr|H0VX00|H0VX00_CAVPO	Uncharacterized protein OS=Cavia porcellus GN=HSPBP1 PE=4 SV=1	CAVPO	3
1984	4.12	9.06	21.03	16.33	16.33	tr|H0VBU1|H0VBU1_CAVPO	Uncharacterized protein (Fragment) OS=Cavia porcellus GN=Syn2 PE=4 SV=1	CAVPO	6
1985	4.12	4.23	21.29	15.07	11.48	tr|H0VEC6|H0VEC6_CAVPO	Uncharacterized protein (Fragment) OS=Cavia	CAVPO	3

							porcellus GN=FAF2 PE=4 SV=1		
1986	4.12	4.48	18.09	16.58	11.56	tr|H0VQI8|H0VQI8_CAVPO	Uncharacterized protein OS=Cavia porcellus GN=SSSCA1 PE=4 SV=1	CAVPO	2
1987	4.12	4.26	19.46	19.46	9.728	tr|H0W286|H0W286_CAVPO	Uncharacterized protein OS=Cavia porcellus GN=ADO PE=4 SV=1	CAVPO	3
1988	4.12	4.14	21	21	16.5	tr|H0W9V3|H0W9V3_CAVPO	Uncharacterized protein OS=Cavia porcellus GN=LOC100721711 PE=4 SV=1	CAVPO	2
1989	4.11	4.26	45.07	40.14	32.39	tr|H0V6Q6|H0V6Q6_CAVPO	Uncharacterized protein OS=Cavia porcellus GN=TOMM22 PE=4 SV=1	CAVPO	3
1990	4.1	4.11	9.548	6.03	4.355	tr|H0UWR2|H0UWR2_CAVPO	Uncharacterized protein OS=Cavia porcellus GN=MBD1 PE=4 SV=1	CAVPO	2
1991	4.1	8.11	24.53	16.23	12.45	tr|H0V6P5|H0V6P5_CAVPO	Uncharacterized protein OS=Cavia porcellus GN=SRSF5 PE=4 SV=1	CAVPO	4
1992	4.1	12.5	49.03	43.24	27.8	tr|H0VQ88|H0VQ88_CAVPO	Uncharacterized protein OS=Cavia porcellus GN=LUC7L PE=4 SV=1	CAVPO	6
1993	4.1	4.11	29.61	23.46	13.97	tr|H0VZ14|H0VZ14_CAVPO	Uncharacterized protein OS=Cavia porcellus GN=ZNF428 PE=4 SV=1	CAVPO	2
1994	4.1	4.23	11.77	6.339	2.199	tr|H0W578|H0W578_CAVPO	Uncharacterized protein OS=Cavia porcellus GN=CHAMP1 PE=4 SV=1	CAVPO	2
1995	4.1	8.11	44.06	43.36	43.36	tr|H0W5I5|H0W5I5_CAVPO	Histone H2A OS=Cavia porcellus GN=H2AFX PE=3 SV=1	CAVPO	13
1996	4.09	4.1	39.78	39.78	39.78	tr|H0UUI3|H0UUI3_CAVPO	Uncharacterized protein OS=Cavia porcellus GN=HIGD1A PE=4 SV=1	CAVPO	3
1997	4.09	4.1	9.717	9.717	9.717	tr|H0UWW1|H0UWW1_CAVPO	Uncharacterized protein (Fragment) OS=Cavia porcellus GN=ZNF207 PE=4 SV=1	CAVPO	4
1998	4.09	4.1	10.24	5.903	3.472	tr|H0VKP4|H0VKP4_CAVPO	Uncharacterized protein OS=Cavia porcellus GN=CDKN2AIP PE=4 SV=1	CAVPO	2

1999	4.09	5.56	35.93	23.38	20.78	tr|H0VPX6|H0VPX6_CAVPO	Uncharacterized protein OS=Cavia porcellus GN=Dnajb6 PE=4 SV=1	CAVPO	4
2000	4.08	4.11	9.128	4.966	3.087	tr|H0UWU1|H0UWU1_CAVPO	Uncharacterized protein OS=Cavia porcellus GN=CUL2 PE=3 SV=1	CAVPO	2
2001	4.08	4.19	32.31	20	12.31	tr|H0UY31|H0UY31_CAVPO	Uncharacterized protein OS=Cavia porcellus GN=Ssu72 PE=4 SV=1	CAVPO	3
2002	4.08	4.25	10.03	8.883	3.868	tr|H0UYP4|H0UYP4_CAVPO	Uncharacterized protein (Fragment) OS=Cavia porcellus GN=SEC63 PE=4 SV=1	CAVPO	2
2003	4.08	4.1	14.35	9.647	6.353	tr|H0VTA3|H0VTA3_CAVPO	Alpha-tubulin N-acetyltransferase 1 OS=Cavia porcellus GN=ATAT1 PE=1 SV=1	CAVPO	2
2004	4.07	4.09	9.766	7.856	5.52	tr|H0V0Q2|H0V0Q2_CAVPO	Uncharacterized protein OS=Cavia porcellus GN=THUMPD2 PE=4 SV=1	CAVPO	2
2005	4.07	4.08	38.46	38.46	38.46	tr|H0VA13|H0VA13_CAVPO	Uncharacterized protein (Fragment) OS=Cavia porcellus GN=COX7A2 PE=4 SV=1	CAVPO	3
2006	4.07	6.13	21.81	18.1	14.15	tr|H0VJJ8|H0VJJ8_CAVPO	Uncharacterized protein OS=Cavia porcellus GN=TMPO PE=4 SV=1	CAVPO	6
2007	4.07	4.22	16.22	16.22	16.22	tr|H0VMU1|H0VMU1_CAVPO	Uncharacterized protein OS=Cavia porcellus GN=SRM PE=3 SV=1	CAVPO	4
2008	4.07	4.08	56.52	56.52	56.52	tr|H0VWA0|H0VWA0_CAVPO	Uncharacterized protein OS=Cavia porcellus GN=S100B PE=4 SV=1	CAVPO	3
2009	4.07	4.2	11.62	5.755	4.886	tr|H0W2T3|H0W2T3_CAVPO	Uncharacterized protein (Fragment) OS=Cavia porcellus GN=WDR17 PE=4 SV=1	CAVPO	3
2010	4.05	4.16	7.204	4.086	2.151	tr|H0V2P2|H0V2P2_CAVPO	Uncharacterized protein OS=Cavia porcellus GN=CC2D1A PE=4 SV=1	CAVPO	2
2011	4.05	4.15	19.53	11.66	8.455	tr|H0V6Q2|H0V6Q2_CAVPO	Uncharacterized protein (Fragment) OS=Cavia porcellus GN=PBX1 PE=3 SV=1	CAVPO	2
2012	4.05	12.1	28.61	20.06	17.99	tr|H0VJX8|H0VJX8_CAVPO	Uncharacterized protein OS=Cavia porcellus GN=SRSF6 PE=4 SV=1	CAVPO	7

2013	4.05	4.37	77.94	69.12	44.12	tr|H0WE10|H0WE10_CAVPO	Guanine nucleotide-binding protein subunit gamma OS=Cavia porcellus GN=GNG7 PE=3 SV=1	CAVPO	2
2014	4.04	4.09	9.556	5.249	3.903	tr|H0V1E7|H0V1E7_CAVPO	Uncharacterized protein OS=Cavia porcellus GN=NUP88 PE=4 SV=1	CAVPO	2
2015	4.04	4.16	54.11	43.84	43.84	tr|H0VQJ8|H0VQJ8_CAVPO	Uncharacterized protein OS=Cavia porcellus GN=CYB5B PE=3 SV=1	CAVPO	3
2016	4.03	4.03	10.18	4.909	4.909	tr|H0V144|H0V144_CAVPO	Uncharacterized protein (Fragment) OS=Cavia porcellus GN=PDPK1 PE=4 SV=1	CAVPO	2
2017	4.03	4.04	19.17	10.36	10.36	tr|H0VCC1|H0VCC1_CAVPO	Uncharacterized protein OS=Cavia porcellus GN=C16orf80 PE=4 SV=1	CAVPO	2
2018	4.03	11.4	45.12	41.46	30.49	tr|H0WDH3|H0WDH3_CAVPO	Uncharacterized protein OS=Cavia porcellus GN=LOC100715281 PE=4 SV=1	CAVPO	7
2019	4.02	6.22	18.26	9.211	7.895	tr|H0V1J5|H0V1J5_CAVPO	Uncharacterized protein (Fragment) OS=Cavia porcellus GN=FMR1 PE=4 SV=1	CAVPO	4
2020	4.02	8.03	10.72	7.32	7.32	tr|H0V406|H0V406_CAVPO	Reticulon OS=Cavia porcellus GN=RTN3 PE=4 SV=1	CAVPO	5
2021	4.02	4.18	13.72	6.342	3.54	tr|H0V4Q0|H0V4Q0_CAVPO	Uncharacterized protein OS=Cavia porcellus GN=TAF6 PE=4 SV=1	CAVPO	2
2022	4.02	4.03	33.57	26.43	16.43	tr|H0VFW4|H0VFW4_CAVPO	Uncharacterized protein OS=Cavia porcellus GN=RPL23 PE=3 SV=1	CAVPO	2
2023	4.02	4.02	13.16	7.895	7.895	tr|H0VVG1|H0VVG1_CAVPO	Uncharacterized protein OS=Cavia porcellus GN=ZNF787 PE=4 SV=1	CAVPO	2
2024	4.02	8.74	21.71	16.57	14.57	tr|H0W958|H0W958_CAVPO	Uncharacterized protein (Fragment) OS=Cavia porcellus GN=SH3GL1 PE=4 SV=1	CAVPO	6
2025	4.01	4.01	28.23	16.75	16.75	tr|H0UYI6|H0UYI6_CAVPO	D-tyrosyl-tRNA(Tyr) deacylase OS=Cavia porcellus GN=DTD1 PE=3 SV=1	CAVPO	2
2026	4.01	4.01	23.03	19.66	19.66	tr|H0VCC7|H0VCC7_CAVPO	Uncharacterized protein (Fragment) OS=Cavia	CAVPO	4

							porcellus GN=RPL11 PE=3 SV=1		
2027	4.01	6.71	22.76	16.21	16.21	tr|H0VHW0|H0VHW0_CAVPO	Uncharacterized protein (Fragment) OS=Cavia porcellus GN=OPCML PE=4 SV=1	CAVPO	4
2028	4.01	4.02	16.11	6.389	6.389	tr|H0VRJ9|H0VRJ9_CAVPO	Uncharacterized protein OS=Cavia porcellus GN=MRPS22 PE=4 SV=1	CAVPO	2
2029	4.01	14.8	41.78	38.54	33.69	tr|H0VZ49|H0VZ49_CAVPO	Uncharacterized protein (Fragment) OS=Cavia porcellus GN=GNB2 PE=4 SV=1	CAVPO	22
2030	4.01	4.03	43.27	37.5	37.5	tr|H0W053|H0W053_CAVPO	Uncharacterized protein (Fragment) OS=Cavia porcellus GN=ERH PE=4 SV=1	CAVPO	4
2031	4.01	4.02	21.51	11.83	11.83	tr|H0WC92|H0WC92_CAVPO	Uncharacterized protein OS=Cavia porcellus GN=H1FX PE=3 SV=1	CAVPO	2
2032	4	10.6	41.8	41.27	41.27	tr|H0UT66|H0UT66_CAVPO	Uncharacterized protein OS=Cavia porcellus GN=HRAS PE=4 SV=1	CAVPO	7
2033	4	4	4.662	4.662	4.662	tr|H0V1X0|H0V1X0_CAVPO	Uncharacterized protein (Fragment) OS=Cavia porcellus GN=SLC35B2 PE=4 SV=1	CAVPO	2
2034	4	4	51.75	51.75	51.75	tr|H0V2W3|H0V2W3_CAVPO	Uncharacterized protein OS=Cavia porcellus GN=Rplp1 PE=3 SV=1	CAVPO	4
2035	4	4	18.1	17.24	17.24	tr|H0V3Q3|H0V3Q3_CAVPO	Uncharacterized protein (Fragment) OS=Cavia porcellus GN=VKORC1L1 PE=4 SV=1	CAVPO	2
2036	4	4	16.13	16.13	16.13	tr|H0V5L0|H0V5L0_CAVPO	Uncharacterized protein OS=Cavia porcellus GN=KIAA1143 PE=4 SV=1	CAVPO	2
2037	4	6.44	12.03	12.03	12.03	tr|H0V6Y9|H0V6Y9_CAVPO	Uncharacterized protein OS=Cavia porcellus PE=4 SV=1	CAVPO	4
2038	4	4.11	25.67	12.64	9.579	tr|H0VAE6|H0VAE6_CAVPO	Uncharacterized protein OS=Cavia porcellus GN=PCSK1N PE=4 SV=1	CAVPO	2
2039	4	4	21.46	21.46	21.46	tr|H0VAU1|H0VAU1_CAVPO	Uncharacterized protein (Fragment) OS=Cavia porcellus GN=MARCKSL1 PE=4 SV=1	CAVPO	4

2040	4	4	24.07	24.07	18.52	tr|H0VCM4|H0VCM4_CAVPO	Uncharacterized protein (Fragment) OS=Cavia porcellus GN=SNRPC PE=3 SV=1	CAVPO	2
2041	4	4.02	31.32	21.43	21.43	tr|H0VF55|H0VF55_CAVPO	Uncharacterized protein OS=Cavia porcellus GN=C11orf58 PE=4 SV=1	CAVPO	4
2042	4	27.5	35.09	30.76	29.13	tr|H0VG24|H0VG24_CAVPO	Uncharacterized protein OS=Cavia porcellus GN=Hsd17b4 PE=3 SV=1	CAVPO	14
2043	4	4	17.28	17.28	17.28	tr|H0VI16|H0VI16_CAVPO	Uncharacterized protein (Fragment) OS=Cavia porcellus GN=NEDD8 PE=4 SV=1	CAVPO	2
2044	4	4.02	19.41	15.29	15.29	tr|H0VIW2|H0VIW2_CAVPO	Uncharacterized protein OS=Cavia porcellus GN=SAT2 PE=4 SV=1	CAVPO	3
2045	4	6.01	33.16	28.06	20.41	tr|H0VJ69|H0VJ69_CAVPO	Uncharacterized protein OS=Cavia porcellus GN=RALA PE=4 SV=1	CAVPO	3
2046	4	19.7	47.54	42.32	39.71	tr|H0VN63|H0VN63_CAVPO	Uncharacterized protein OS=Cavia porcellus GN=RBM4 PE=4 SV=1	CAVPO	11
2047	4	4	24.79	24.79	24.79	tr|H0VS59|H0VS59_CAVPO	Uncharacterized protein (Fragment) OS=Cavia porcellus GN=FHIT PE=4 SV=1	CAVPO	2
2048	4	6	5.323	5.323	5.323	tr|H0VS95|H0VS95_CAVPO	Transporter OS=Cavia porcellus GN=SLC6A6 PE=3 SV=1	CAVPO	3
2049	4	4	20	11.43	11.43	tr|H0VVN6|H0VVN6_CAVPO	Uncharacterized protein OS=Cavia porcellus GN=CIRBP PE=4 SV=1	CAVPO	3
2050	4	4	32.89	15.79	15.79	tr|H0W5R7|H0W5R7_CAVPO	Uncharacterized protein OS=Cavia porcellus GN=FIS1 PE=4 SV=1	CAVPO	2
2051	4	4.01	33.7	25	25	tr|H0W8S9|H0W8S9_CAVPO	Uncharacterized protein OS=Cavia porcellus GN=LOC100721790 PE=4 SV=1	CAVPO	2
2052	4	4	13.69	13.69	13.69	tr|H0WAJ1|H0WAJ1_CAVPO	Uncharacterized protein OS=Cavia porcellus GN=PTP4A2 PE=4 SV=1	CAVPO	2
2053	4	15.9	74.6	69.05	60.32	tr|H0WBT4|H0WBT4_CAVPO	Histone H2B OS=Cavia porcellus GN=HIST1H2BB PE=3 SV=1	CAVPO	31

2054	4	4.08	6.043	6.043	6.043	tr|Q9TEG9|Q9TEG9_CAVPO	Cytochrome c oxidase subunit 1 OS=Cavia porcellus GN=COI PE=3 SV=1	CAVPO	3
2055	3.99	4.12	13.28	13.28	11.11	tr|H0WAC5|H0WAC5_CAVPO	Uncharacterized protein (Fragment) OS=Cavia porcellus GN=LOC100721198 PE=4 SV=1	CAVPO	3
2056	3.98	4.21	14.85	7.723	7.723	tr|H0V9S3|H0V9S3_CAVPO	Uncharacterized protein OS=Cavia porcellus GN=FAM114A2 PE=4 SV=1	CAVPO	3
2057	3.98	4.21	19.34	14.63	8.711	tr|H0VGL9|H0VGL9_CAVPO	Uncharacterized protein OS=Cavia porcellus GN=FUBP3 PE=4 SV=1	CAVPO	3
2058	3.97	4.09	34.3	34.3	16.86	tr|H0V2B1|H0V2B1_CAVPO	Uncharacterized protein OS=Cavia porcellus GN=LOC100718753 PE=4 SV=1	CAVPO	2
2059	3.97	4.08	26.55	10.73	7.627	tr|H0VC30|H0VC30_CAVPO	Uncharacterized protein (Fragment) OS=Cavia porcellus GN=VPS36 PE=4 SV=1	CAVPO	2
2060	3.97	4.05	37.5	37.5	37.5	tr|H0VS34|H0VS34_CAVPO	Uncharacterized protein (Fragment) OS=Cavia porcellus GN=SEC61B PE=4 SV=1	CAVPO	3
2061	3.97	4.08	17.05	16.48	12.5	tr|H0VST8|H0VST8_CAVPO	60S ribosomal protein L18a OS=Cavia porcellus GN=Rpl18a PE=3 SV=1	CAVPO	2
2062	3.96	4.06	16.4	8.907	4.453	tr|H0V3E1|H0V3E1_CAVPO	UDP-glucose 6-dehydrogenase OS=Cavia porcellus GN=UGDH PE=3 SV=1	CAVPO	2
2063	3.96	4.21	16.69	9.7	6.99	tr|H0VIR4|H0VIR4_CAVPO	Uncharacterized protein (Fragment) OS=Cavia porcellus GN=KIF3A PE=4 SV=1	CAVPO	4
2064	3.96	4.13	12.98	6.711	4.73	tr|H0W3R1|H0W3R1_CAVPO	Uncharacterized protein (Fragment) OS=Cavia porcellus GN=DNMT3A PE=4 SV=1	CAVPO	3
2065	3.95	4.03	9.742	9.742	7.736	tr|H0V0H6|H0V0H6_CAVPO	Uncharacterized protein (Fragment) OS=Cavia porcellus GN=PTER PE=4 SV=1	CAVPO	2
2066	3.95	27.8	54.64	48.28	46.95	tr|H0VGS3|H0VGS3_CAVPO	Uncharacterized protein OS=Cavia porcellus GN=ACTA1 PE=3 SV=1	CAVPO	39
2067	3.95	4.04	17.99	5.913	5.913	tr|H0W3Y1|H0W3Y1_CAVPO	Uncharacterized protein (Fragment) OS=Cavia porcellus GN=MTCH1 PE=3 SV=1	CAVPO	2

2068	3.94	4.04	16.28	7.849	6.105	tr|H0VNF2|H0VNF2_CAVPO	Uncharacterized protein OS=Cavia porcellus GN=NAGK PE=4 SV=1	CAVPO	2
2069	3.93	4.01	33.81	33.81	26.62	tr|H0V206|H0V206_CAVPO	Uncharacterized protein (Fragment) OS=Cavia porcellus GN=NUDT3 PE=3 SV=1	CAVPO	2
2070	3.93	4.21	19.48	13.75	13.75	tr|H0V576|H0V576_CAVPO	Uncharacterized protein (Fragment) OS=Cavia porcellus GN=LRPAP1 PE=4 SV=1	CAVPO	4
2071	3.93	4.15	11.07	7.946	2.198	tr|H0VKE1|H0VKE1_CAVPO	Uncharacterized protein (Fragment) OS=Cavia porcellus GN=EIF5B PE=4 SV=1	CAVPO	2
2072	3.92	4.02	14.78	14.78	10.48	tr|H0VAI6|H0VAI6_CAVPO	Uncharacterized protein OS=Cavia porcellus GN=HEPACAM PE=4 SV=1	CAVPO	2
2073	3.92	4.11	15.45	8.485	5.758	tr|H0VF49|H0VF49_CAVPO	Uncharacterized protein OS=Cavia porcellus GN=LEO1 PE=4 SV=1	CAVPO	4
2074	3.92	4	19.47	19.47	19.47	tr|H0W2L5|H0W2L5_CAVPO	Uncharacterized protein OS=Cavia porcellus GN=PDDC1 PE=4 SV=1	CAVPO	2
2075	3.92	4.05	8.715	8.715	8.715	tr|Q9TEG2|Q9TEG2_CAVPO	NADH-ubiquinone oxidoreductase chain 4 (Fragment) OS=Cavia porcellus GN=NADH4 PE=3 SV=1	CAVPO	4
2076	3.91	4.18	10.49	8.663	4.103	tr|H0UXQ6|H0UXQ6_CAVPO	Uncharacterized protein OS=Cavia porcellus GN=ABCD3 PE=3 SV=1	CAVPO	2
2077	3.91	4.1	11.99	7.659	4.769	tr|H0VVF4|H0VVF4_CAVPO	Uncharacterized protein OS=Cavia porcellus GN=MDM1 PE=4 SV=1	CAVPO	3
2078	3.9	4.23	22.63	15.7	6.697	tr|H0VB05|H0VB05_CAVPO	Uncharacterized protein OS=Cavia porcellus GN=TRNT1 PE=3 SV=1	CAVPO	3
2079	3.9	4.25	7.775	4.313	3.916	tr|H0VVG8|H0VVG8_CAVPO	Uncharacterized protein (Fragment) OS=Cavia porcellus GN=CASZ1 PE=4 SV=1	CAVPO	4
2080	3.89	3.96	13.85	13.85	13.85	tr|H0WBA4|H0WBA4_CAVPO	Uncharacterized protein (Fragment) OS=Cavia porcellus GN=ATP6V0C PE=3 SV=1	CAVPO	2
2081	3.89	3.96	15.83	15.83	15.83	tr|H0WBH9|H0WBH9_CAVPO	Uncharacterized protein (Fragment) OS=Cavia	CAVPO	2

							porcellus GN=ACOT13 PE=4 SV=1		
2082	3.88	3.99	27.1	21.94	14.84	tr|H0V294|H0V294_CAVPO	Fibroblast growth factor (Fragment) OS=Cavia porcellus GN=FGF1 PE=3 SV=1	CAVPO	2
2083	3.86	10.9	42.03	28.57	26.37	tr|H0VGZ1|H0VGZ1_CAVPO	Uncharacterized protein (Fragment) OS=Cavia porcellus GN=ELAVL2 PE=4 SV=1	CAVPO	13
2084	3.86	4.13	14.66	13.54	8.72	tr|H0VKA6|H0VKA6_CAVPO	Uncharacterized protein OS=Cavia porcellus GN=PCCB PE=4 SV=1	CAVPO	3
2085	3.85	3.93	2.526	1.973	1.973	tr|H0V7B5|H0V7B5_CAVPO	Uncharacterized protein (Fragment) OS=Cavia porcellus GN=NPC1 PE=4 SV=1	CAVPO	2
2086	3.84	4.04	7.366	6.846	3.12	tr|H0VGS6|H0VGS6_CAVPO	Uncharacterized protein OS=Cavia porcellus GN=NUP133 PE=4 SV=1	CAVPO	3
2087	3.82	3.97	13.64	8.079	3.591	tr|H0W4J0|H0W4J0_CAVPO	Uncharacterized protein OS=Cavia porcellus GN=ISYNA1 PE=4 SV=1	CAVPO	2
2088	3.82	3.97	21.39	12.05	12.05	tr|H0W7Y7|H0W7Y7_CAVPO	Uncharacterized protein OS=Cavia porcellus GN=GIPC1 PE=4 SV=1	CAVPO	3
2089	3.82	3.97	29.72	27.31	13.25	tr|H0WCI4|H0WCI4_CAVPO	Uncharacterized protein OS=Cavia porcellus GN=HDHD3 PE=4 SV=1	CAVPO	2
2090	3.81	3.91	14.44	10.28	7.5	tr|H0V089|H0V089_CAVPO	Uncharacterized protein OS=Cavia porcellus GN=VSX2 PE=3 SV=1	CAVPO	2
2091	3.81	13.2	30.84	22.8	18.32	tr|H0V4K5|H0V4K5_CAVPO	Uncharacterized protein OS=Cavia porcellus GN=EHD3 PE=4 SV=1	CAVPO	8
2092	3.81	3.98	25.32	20.46	8.951	tr|H0VT92|H0VT92_CAVPO	Uncharacterized protein OS=Cavia porcellus GN=ILKAP PE=3 SV=1	CAVPO	3
2093	3.8	4.14	9.979	7.511	4.077	tr|H0VCS9|H0VCS9_CAVPO	Uncharacterized protein (Fragment) OS=Cavia porcellus GN=NUP107 PE=4 SV=1	CAVPO	3
2094	3.79	3.98	9.682	7.162	5.438	tr|H0UVN5|H0UVN5_CAVPO	Elongation factor G, mitochondrial OS=Cavia porcellus GN=GFM1 PE=3 SV=1	CAVPO	3

2095	3.79	3.96	5.751	4.257	2.539	tr|H0VDA2|H0VDA2_CAVPO	Uncharacterized protein (Fragment) OS=Cavia porcellus GN=CNTNAP1 PE=4 SV=1	CAVPO	3
2096	3.79	3.89	20.77	7.923	7.923	tr|H0VR36|H0VR36_CAVPO	Uncharacterized protein (Fragment) OS=Cavia porcellus GN=MAP2K4 PE=4 SV=1	CAVPO	2
2097	3.78	4	29.82	18.18	8.727	tr|H0V3E4|H0V3E4_CAVPO	Uncharacterized protein OS=Cavia porcellus GN=POLR2C PE=4 SV=1	CAVPO	2
2098	3.78	4.07	40.94	33.56	30.87	tr|H0VIZ9|H0VIZ9_CAVPO	Uncharacterized protein (Fragment) OS=Cavia porcellus GN=Rps16 PE=3 SV=1	CAVPO	5
2099	3.78	3.9	16.24	5.791	4.096	tr|H0VJY9|H0VJY9_CAVPO	Uncharacterized protein OS=Cavia porcellus GN=TMCC2 PE=4 SV=1	CAVPO	2
2100	3.78	3.95	28.8	28.8	21.2	tr|H0W306|H0W306_CAVPO	Uncharacterized protein OS=Cavia porcellus GN=RABL5 PE=4 SV=1	CAVPO	3
2101	3.76	3.91	11.92	6.908	5.699	tr|H0VI47|H0VI47_CAVPO	Uncharacterized protein OS=Cavia porcellus GN=CDC40 PE=4 SV=1	CAVPO	3
2102	3.76	3.9	9.991	3.268	3.268	tr|H0VTC5|H0VTC5_CAVPO	Uncharacterized protein (Fragment) OS=Cavia porcellus GN=ANKS1A PE=4 SV=1	CAVPO	3
2103	3.76	3.98	21.03	14.48	11.72	tr|H0W6L3|H0W6L3_CAVPO	Uncharacterized protein OS=Cavia porcellus GN=MBD3 PE=4 SV=1	CAVPO	3
2104	3.75	3.83	9	5.25	5.25	tr|H0VVS4|H0VVS4_CAVPO	Uncharacterized protein (Fragment) OS=Cavia porcellus GN=BSCL2 PE=4 SV=1	CAVPO	3
2105	3.74	4.72	9.724	6.242	3.721	tr|H0VQR1|H0VQR1_CAVPO	Uncharacterized protein OS=Cavia porcellus GN=STT3B PE=4 SV=1	CAVPO	4
2106	3.74	3.89	11.01	6.709	6.709	tr|H0WB62|H0WB62_CAVPO	Receptor-type tyrosine-protein phosphatase OS=Cavia porcellus GN=PTPRA PE=3 SV=1	CAVPO	4
2107	3.73	3.86	13.68	10.26	10.26	tr|H0V7M8|H0V7M8_CAVPO	Uncharacterized protein OS=Cavia porcellus GN=ARMC6 PE=4 SV=1	CAVPO	3
2108	3.73	3.85	36.48	22.01	15.09	tr|H0VFR9|H0VFR9_CAVPO	Uncharacterized protein (Fragment) OS=Cavia porcellus GN=NDUFAF3 PE=4 SV=1	CAVPO	2

2109	3.73	3.96	5.993	3.12	2.135	tr|H0VH14|H0VH14_CAVPO	Uncharacterized protein OS=Cavia porcellus GN=VCPIP1 PE=4 SV=1	CAVPO	2
2110	3.72	3.91	11.42	8.199	6.003	tr|H0VBH3|H0VBH3_CAVPO	Uncharacterized protein OS=Cavia porcellus GN=PRPF3 PE=4 SV=1	CAVPO	3
2111	3.72	4.66	9.428	2.441	2.441	tr|H0VPJ3|H0VPJ3_CAVPO	Uncharacterized protein (Fragment) OS=Cavia porcellus GN=WDR33 PE=4 SV=1	CAVPO	3
2112	3.7	3.97	15.95	15.95	13.23	tr|H0VXR3|H0VXR3_CAVPO	Uncharacterized protein OS=Cavia porcellus GN=RPL8 PE=3 SV=1	CAVPO	3
2113	3.69	3.93	34.06	25.33	19.21	tr|H0VEH1|H0VEH1_CAVPO	Uncharacterized protein (Fragment) OS=Cavia porcellus GN=NIPSNAP3A PE=4 SV=1	CAVPO	4
2114	3.69	3.83	17.8	11.3	8.757	tr|H0VR47|H0VR47_CAVPO	Uncharacterized protein OS=Cavia porcellus GN=L3HYPDH PE=4 SV=1	CAVPO	2
2115	3.68	3.87	9.95	9.95	7.131	tr|Q9TEG1|Q9TEG1_CAVPO	NADH-ubiquinone oxidoreductase chain 5 OS=Cavia porcellus GN=NADH5 PE=3 SV=1	CAVPO	3
2116	3.66	3.86	11.16	6.803	4.082	tr|H0UXT7|H0UXT7_CAVPO	Uncharacterized protein OS=Cavia porcellus GN=DDX50 PE=4 SV=1	CAVPO	3
2117	3.66	3.79	22.43	7.477	7.477	tr|H0VIZ6|H0VIZ6_CAVPO	Uncharacterized protein OS=Cavia porcellus GN=PAF1 PE=4 SV=1	CAVPO	3
2118	3.66	3.72	12	8.333	8.333	tr|H0W4Q2|H0W4Q2_CAVPO	Uncharacterized protein OS=Cavia porcellus GN=SLC25A35 PE=3 SV=1	CAVPO	2
2119	3.65	3.82	11.72	6.345	2.759	tr|H0UX18|H0UX18_CAVPO	Uncharacterized protein OS=Cavia porcellus GN=BRD3 PE=4 SV=1	CAVPO	2
2120	3.64	3.73	22.54	22.54	14.79	tr|H0UYN3|H0UYN3_CAVPO	Uncharacterized protein OS=Cavia porcellus GN=LAMTOR2 PE=4 SV=1	CAVPO	4
2121	3.64	3.75	36.73	35.71	18.37	tr|H0W8Z0|H0W8Z0_CAVPO	Uncharacterized protein OS=Cavia porcellus GN=NDUFB3 PE=4 SV=1	CAVPO	2
2122	3.63	3.74	21.55	21.55	21.55	tr|H0V1N1|H0V1N1_CAVPO	Uncharacterized protein OS=Cavia porcellus GN=NDUFS6 PE=4 SV=1	CAVPO	2

2123	3.63	3.82	29.51	16.39	16.39	tr|H0V4M4|H0V4M4_CAVPO	Uncharacterized protein OS=Cavia porcellus GN=UBE2H PE=3 SV=1	CAVPO	2
2124	3.62	3.86	11.07	8.544	4.332	tr|H0V4M6|H0V4M6_CAVPO	Uncharacterized protein (Fragment) OS=Cavia porcellus GN=CARS PE=3 SV=1	CAVPO	2
2125	3.6	3.75	21.7	16.04	10.69	tr|H0UWM1|H0UWM1_CAVPO	Uncharacterized protein OS=Cavia porcellus GN=UBLCP1 PE=4 SV=1	CAVPO	3
2126	3.6	3.69	20.5	12.97	7.531	tr|H0VHT8|H0VHT8_CAVPO	Uncharacterized protein OS=Cavia porcellus GN=LYPLAL1 PE=4 SV=1	CAVPO	2
2127	3.6	3.69	16.9	12.21	8.216	tr|H0VMN1|H0VMN1_CAVPO	Uncharacterized protein OS=Cavia porcellus GN=ZC3H15 PE=4 SV=1	CAVPO	2
2128	3.6	3.68	20	20	20	tr|H0VTA8|H0VTA8_CAVPO	Uncharacterized protein OS=Cavia porcellus GN=Trmt112 PE=4 SV=1	CAVPO	2
2129	3.6	3.71	16.74	14.32	7.93	tr|H0W1Y8|H0W1Y8_CAVPO	Uncharacterized protein (Fragment) OS=Cavia porcellus GN=NOVA2 PE=4 SV=1	CAVPO	3
2130	3.6	3.7	13.24	6.721	5.092	tr|H0WBZ5|H0WBZ5_CAVPO	Uncharacterized protein OS=Cavia porcellus GN=ALG11 PE=4 SV=1	CAVPO	2
2131	3.59	3.64	30.16	30.16	30.16	tr|H0VC82|H0VC82_CAVPO	Uncharacterized protein OS=Cavia porcellus GN=SRP9 PE=4 SV=1	CAVPO	2
2132	3.58	3.76	15.74	6.085	3.175	tr|H0UU40|H0UU40_CAVPO	Uncharacterized protein OS=Cavia porcellus GN=LIMA1 PE=4 SV=1	CAVPO	2
2133	3.58	3.92	17.08	16.8	6.612	tr|H0V6R3|H0V6R3_CAVPO	Uncharacterized protein (Fragment) OS=Cavia porcellus GN=FAM50A PE=4 SV=1	CAVPO	2
2134	3.58	3.65	11.48	7.65	7.65	tr|H0W0J3|H0W0J3_CAVPO	Histone-lysine N-methyltransferase SETD7 OS=Cavia porcellus GN=SETD7 PE=3 SV=1	CAVPO	2
2135	3.58	3.91	22.19	15.93	5.744	tr|H0W3L7|H0W3L7_CAVPO	Uncharacterized protein OS=Cavia porcellus GN=COPG2 PE=4 SV=1	CAVPO	2
2136	3.57	3.75	21.84	17.48	9.223	tr|H0UVS9|H0UVS9_CAVPO	Uncharacterized protein OS=Cavia porcellus GN=BLVRB PE=4 SV=1	CAVPO	2

2137	3.57	4.65	7.779	5.375	5.375	tr|H0VPD7|H0VPD7_CAVPO	Choline transporter-like protein 2 OS=Cavia porcellus GN=SLC44A2 PE=4 SV=1	CAVPO	4
2138	3.57	3.66	9.189	4.865	4.865	tr|H0W2N8|H0W2N8_CAVPO	Uncharacterized protein (Fragment) OS=Cavia porcellus GN=PGS1 PE=4 SV=1	CAVPO	2
2139	3.56	3.71	10.63	7.975	3.544	tr|H0VXN9|H0VXN9_CAVPO	Signal transducer and activator of transcription (Fragment) OS=Cavia porcellus GN=Stat5b PE=3 SV=1	CAVPO	3
2140	3.55	3.62	24.6	24.6	24.6	tr|H0WDR4|H0WDR4_CAVPO	Uncharacterized protein OS=Cavia porcellus GN=C12orf57 PE=4 SV=1	CAVPO	2
2141	3.54	3.66	24.84	24.84	24.84	tr|H0V0N9|H0V0N9_CAVPO	Single-stranded DNA-binding protein OS=Cavia porcellus GN=SSBP1 PE=3 SV=1	CAVPO	3
2142	3.54	3.7	10	5.596	4.495	tr|H0VSF2|H0VSF2_CAVPO	Uncharacterized protein OS=Cavia porcellus GN=FUK PE=3 SV=1	CAVPO	3
2143	3.52	3.68	11.68	9.137	5.584	tr|H0UVK8|H0UVK8_CAVPO	Uncharacterized protein (Fragment) OS=Cavia porcellus GN=PAG1 PE=4 SV=1	CAVPO	2
2144	3.52	3.61	19.61	14.71	11.27	tr|H0UY44|H0UY44_CAVPO	Ribosomal protein L15 OS=Cavia porcellus GN=RPL15 PE=3 SV=1	CAVPO	2
2145	3.51	3.7	24.51	20.33	8.357	tr|H0UY67|H0UY67_CAVPO	Uncharacterized protein (Fragment) OS=Cavia porcellus GN=GDAP1 PE=4 SV=1	CAVPO	3
2146	3.51	3.59	10.67	4.296	4.296	tr|H0V0J4|H0V0J4_CAVPO	Uncharacterized protein OS=Cavia porcellus GN=MPP5 PE=4 SV=1	CAVPO	2
2147	3.51	3.7	5.858	4.15	1.871	tr|H0VNI9|H0VNI9_CAVPO	Uncharacterized protein (Fragment) OS=Cavia porcellus GN=ZMYND8 PE=4 SV=1	CAVPO	2
2148	3.5	3.82	18.4	18.4	16.87	tr|H0UXN6|H0UXN6_CAVPO	Uncharacterized protein OS=Cavia porcellus GN=MLST8 PE=4 SV=1	CAVPO	3
2149	3.5	3.72	6.05	3.139	2.055	tr|H0V0P1|H0V0P1_CAVPO	Uncharacterized protein (Fragment) OS=Cavia porcellus GN=RP1L1 PE=4 SV=1	CAVPO	3
2150	3.5	3.69	11.73	5.537	4.072	tr|H0V143|H0V143_CAVPO	Uncharacterized protein OS=Cavia porcellus	CAVPO	2

							GN=XRCC6 PE=4 SV=1		
2151	3.49	3.57	5.882	2.222	1.373	tr|H0VG49|H0VG49_CAVPO	Uncharacterized protein (Fragment) OS=Cavia porcellus GN=ZNF638 PE=4 SV=1	CAVPO	2
2152	3.49	3.62	23.51	12.91	8.609	tr|H0VUW5|H0VUW5_CAVPO	Uncharacterized protein (Fragment) OS=Cavia porcellus GN=WDR61 PE=4 SV=1	CAVPO	2
2153	3.49	3.61	24.49	24.49	24.49	tr|H0W4B9|H0W4B9_CAVPO	Uncharacterized protein OS=Cavia porcellus GN=UBE2D3 PE=3 SV=1	CAVPO	6
2154	3.48	4.26	7.058	4.235	4.235	tr|H0UU87|H0UU87_CAVPO	Ubiquitin carboxyl-terminal hydrolase OS=Cavia porcellus GN=USP15 PE=3 SV=1	CAVPO	4
2155	3.48	3.65	10.75	10.57	4.839	tr|H0VHQ3|H0VHQ3_CAVPO	Uncharacterized protein OS=Cavia porcellus GN=ABHD16A PE=4 SV=1	CAVPO	2
2156	3.48	4.11	6.089	5.057	3.199	tr|H0VHU4|H0VHU4_CAVPO	Uncharacterized protein (Fragment) OS=Cavia porcellus GN=EPHB2 PE=4 SV=1	CAVPO	2
2157	3.48	3.58	17.71	11.44	7.749	tr|H0VIF1|H0VIF1_CAVPO	Uncharacterized protein (Fragment) OS=Cavia porcellus GN=MTX2 PE=4 SV=1	CAVPO	2
2158	3.48	3.63	4.251	1.756	1.756	tr|H0VLG3|H0VLG3_CAVPO	Uncharacterized protein (Fragment) OS=Cavia porcellus GN=SCAF4 PE=4 SV=1	CAVPO	2
2159	3.48	3.79	21.74	12.11	6.832	tr|H0VUC7|H0VUC7_CAVPO	Uncharacterized protein OS=Cavia porcellus GN=PPWD1 PE=4 SV=1	CAVPO	3
2160	3.47	3.55	12.3	10.71	7.517	tr|H0UXX6|H0UXX6_CAVPO	Uncharacterized protein OS=Cavia porcellus GN=MFAP1 PE=4 SV=1	CAVPO	3
2161	3.47	3.56	12.87	9.885	7.816	tr|H0V0B1|H0V0B1_CAVPO	Uncharacterized protein OS=Cavia porcellus GN=NXN PE=4 SV=1	CAVPO	2
2162	3.46	3.62	19.27	12.5	12.5	tr|H0VIM5|H0VIM5_CAVPO	Uncharacterized protein OS=Cavia porcellus GN=BAX PE=4 SV=1	CAVPO	2
2163	3.46	3.52	39.06	37.5	37.5	tr|H0VIM6|H0VIM6_CAVPO	Uncharacterized protein OS=Cavia porcellus GN=UQCR10 PE=4 SV=1	CAVPO	2

2164	3.46	3.54	28.39	28.39	15.48	tr|H0WA58|H0WA58_CAVPO	Uncharacterized protein (Fragment) OS=Cavia porcellus GN=HINT3 PE=4 SV=1	CAVPO	2
2165	3.45	3.56	41.75	27.18	27.18	tr|H0VM11|H0VM11_CAVPO	Uncharacterized protein (Fragment) OS=Cavia porcellus GN=PCBD1 PE=3 SV=1	CAVPO	3
2166	3.45	3.52	48.25	40.35	40.35	tr|H0VS83|H0VS83_CAVPO	Uncharacterized protein OS=Cavia porcellus GN=COX7A2L PE=4 SV=1	CAVPO	2
2167	3.45	3.75	7.527	7.348	4.659	tr|H0W1K7|H0W1K7_CAVPO	Uncharacterized protein (Fragment) OS=Cavia porcellus GN=YTHDF2 PE=4 SV=1	CAVPO	2
2168	3.44	3.56	3.955	2.637	2.637	tr|H0UU68|H0UU68_CAVPO	Uncharacterized protein OS=Cavia porcellus GN=TSC22D1 PE=4 SV=1	CAVPO	3
2169	3.44	3.63	11.78	7.36	2.159	tr|H0UUS1|H0UUS1_CAVPO	Uncharacterized protein OS=Cavia porcellus GN=IDE PE=3 SV=1	CAVPO	2
2170	3.43	3.51	8.812	8.812	8.812	tr|H0UXA9|H0UXA9_CAVPO	Uncharacterized protein (Fragment) OS=Cavia porcellus GN=RNF170 PE=4 SV=1	CAVPO	2
2171	3.43	5.62	9.156	7.181	5.566	tr|H0V113|H0V113_CAVPO	Uncharacterized protein OS=Cavia porcellus GN=CPNE6 PE=4 SV=1	CAVPO	3
2172	3.42	3.57	34.59	28.65	12.97	tr|H0V773|H0V773_CAVPO	Uncharacterized protein (Fragment) OS=Cavia porcellus GN=MANF PE=4 SV=1	CAVPO	2
2173	3.42	3.53	50	34.88	24.42	tr|H0VLF5|H0VLF5_CAVPO	Uncharacterized protein OS=Cavia porcellus GN=SF3B5 PE=4 SV=1	CAVPO	2
2174	3.42	6.35	19.63	11.16	9.04	tr|H0VUI7|H0VUI7_CAVPO	Uncharacterized protein (Fragment) OS=Cavia porcellus GN=MTA1 PE=4 SV=1	CAVPO	6
2175	3.42	3.68	35.1	22.6	15.87	tr|H0WAS1|H0WAS1_CAVPO	Uncharacterized protein OS=Cavia porcellus GN=MESDC2 PE=4 SV=1	CAVPO	3
2176	3.41	3.68	20.66	20.66	14.43	tr|H0V476|H0V476_CAVPO	Serine/threonine-protein phosphatase OS=Cavia porcellus GN=PPP6C PE=3 SV=1	CAVPO	3
2177	3.41	3.49	33.09	19.12	19.12	tr|H0V6E4|H0V6E4_CAVPO	Uncharacterized protein OS=Cavia porcellus GN=SNAPIN PE=4 SV=1	CAVPO	2

2178	3.41	12.2	54.44	51.11	47.78	tr|H0VGN3|H0VGN3_CAVPO	Uncharacterized protein OS=Cavia porcellus GN=ARF4 PE=3 SV=1	CAVPO	8
2179	3.41	3.63	28.07	22.81	22.81	tr|H0WDP2|H0WDP2_CAVPO	2'-deoxynucleoside 5'-phosphate N-hydrolase 1 OS=Cavia porcellus GN=DNPH1 PE=3 SV=1	CAVPO	2
2180	3.4	3.47	8.219	6.575	6.575	tr|H0UYB2|H0UYB2_CAVPO	Uncharacterized protein OS=Cavia porcellus GN=LOC100713047 PE=3 SV=1	CAVPO	2
2181	3.4	3.52	20.38	16.61	6.583	tr|H0W9L8|H0W9L8_CAVPO	Uncharacterized protein OS=Cavia porcellus GN=RDH14 PE=3 SV=1	CAVPO	2
2182	3.39	3.58	15.13	9.963	9.963	tr|H0V512|H0V512_CAVPO	Uncharacterized protein (Fragment) OS=Cavia porcellus GN=AQP11 PE=3 SV=1	CAVPO	2
2183	3.38	3.51	5.312	2.049	2.049	tr|H0V3B4|H0V3B4_CAVPO	Uncharacterized protein (Fragment) OS=Cavia porcellus GN=NBEA PE=4 SV=1	CAVPO	3
2184	3.37	3.47	14.43	3.659	2.846	tr|H0UVL4|H0UVL4_CAVPO	Uncharacterized protein (Fragment) OS=Cavia porcellus GN=FARP1 PE=4 SV=1	CAVPO	2
2185	3.37	3.52	5.373	3.289	2.083	tr|H0VG12|H0VG12_CAVPO	Uncharacterized protein (Fragment) OS=Cavia porcellus GN=Mthfd1l PE=3 SV=1	CAVPO	2
2186	3.37	3.54	25.53	15.32	10.21	tr|H0VV40|H0VV40_CAVPO	Uncharacterized protein (Fragment) OS=Cavia porcellus GN=SRA1 PE=4 SV=1	CAVPO	2
2187	3.36	3.59	25.32	14.14	5.907	tr|H0VSU8|H0VSU8_CAVPO	Uncharacterized protein OS=Cavia porcellus GN=GABRB2 PE=3 SV=1	CAVPO	2
2188	3.36	3.58	33.19	25.33	16.59	tr|H0VXH6|H0VXH6_CAVPO	Uncharacterized protein (Fragment) OS=Cavia porcellus GN=CLIC6 PE=4 SV=1	CAVPO	3
2189	3.35	3.47	6.355	2.983	2.983	tr|H0VB80|H0VB80_CAVPO	Signal transducer and activator of transcription OS=Cavia porcellus GN=STAT3 PE=3 SV=1	CAVPO	2
2190	3.35	3.44	58.97	41.03	29.91	tr|H0VCA9|H0VCA9_CAVPO	Uncharacterized protein OS=Cavia porcellus GN=C12orf23 PE=4 SV=1	CAVPO	2
2191	3.34	3.47	5.128	4.103	4.103	tr|H0V4N4|H0V4N4_CAVPO	Uncharacterized protein OS=Cavia porcellus	CAVPO	2

							GN=ZW10 PE=4 SV=1		
2192	3.34	4.03	24.92	18.36	7.213	tr|H0VFX9|H0VFX9_CAVPO	Uncharacterized protein OS=Cavia porcellus GN=EIF2B1 PE=3 SV=1	CAVPO	2
2193	3.33	3.4	11.25	4.279	4.279	tr|H0VL40|H0VL40_CAVPO	Uncharacterized protein OS=Cavia porcellus GN=TBRG4 PE=4 SV=1	CAVPO	2
2194	3.32	3.42	4.229	4.229	2.865	tr|H0VP63|H0VP63_CAVPO	Uncharacterized protein OS=Cavia porcellus GN=ZC3H14 PE=4 SV=1	CAVPO	2
2195	3.31	3.4	30.14	27.4	16.44	tr|H0VM93|H0VM93_CAVPO	Uncharacterized protein OS=Cavia porcellus GN=RBPMS PE=4 SV=1	CAVPO	2
2196	3.3	3.65	18.8	18.55	9.023	tr|H0V2C4|H0V2C4_CAVPO	Uncharacterized protein OS=Cavia porcellus GN=ABHD12 PE=4 SV=1	CAVPO	4
2197	3.29	3.38	35.81	22.97	18.24	tr|H0WA12|H0WA12_CAVPO	Uncharacterized protein OS=Cavia porcellus GN=EDF1 PE=4 SV=1	CAVPO	2
2198	3.28	3.4	8.423	6.989	4.48	tr|H0V1E6|H0V1E6_CAVPO	Uncharacterized protein OS=Cavia porcellus GN=ARIH1 PE=4 SV=1	CAVPO	2
2199	3.28	3.34	10.89	9.556	7.778	tr|H0W2W9|H0W2W9_CAVPO	Uncharacterized protein OS=Cavia porcellus GN=SF3A2 PE=4 SV=1	CAVPO	2
2200	3.28	3.74	38.1	38.1	38.1	tr|H0W4Y4|H0W4Y4_CAVPO	Uncharacterized protein OS=Cavia porcellus GN=MYCBP PE=4 SV=1	CAVPO	3
2201	3.28	3.34	20	20	20	tr|H0W7S0|H0W7S0_CAVPO	Uncharacterized protein OS=Cavia porcellus GN=RPL32 PE=4 SV=1	CAVPO	3
2202	3.28	3.43	39.62	27.36	19.81	tr|H0WCA3|H0WCA3_CAVPO	60S ribosomal protein L36 OS=Cavia porcellus GN=RPL36 PE=3 SV=1	CAVPO	2
2203	3.26	17.3	25.19	22.71	20.8	tr|H0VBD6|H0VBD6_CAVPO	Serine/threonine-protein phosphatase (Fragment) OS=Cavia porcellus GN=PPP3CB PE=3 SV=1	CAVPO	11
2204	3.26	3.32	16.42	16.42	16.42	tr|H0VGG2|H0VGG2_CAVPO	Uncharacterized protein OS=Cavia porcellus GN=BOLA1 PE=3 SV=1	CAVPO	2

2205	3.26	3.65	15.59	9.197	2.962	tr|H0VXE6|H0VXE6_CAVPO	Uncharacterized protein OS=Cavia porcellus GN=SON PE=4 SV=1	CAVPO	2
2206	3.25	3.33	7.399	4.036	3.139	tr|H0UYQ5|H0UYQ5_CAVPO	Uncharacterized protein OS=Cavia porcellus GN=RBM27 PE=4 SV=1	CAVPO	2
2207	3.25	3.39	6.555	4.603	2.301	tr|H0V541|H0V541_CAVPO	Uncharacterized protein (Fragment) OS=Cavia porcellus GN=NUP160 PE=4 SV=1	CAVPO	2
2208	3.25	3.37	16.25	11.87	8.75	tr|H0V602|H0V602_CAVPO	Pyrroline-5-carboxylate reductase OS=Cavia porcellus GN=Pycr2 PE=3 SV=1	CAVPO	2
2209	3.24	3.32	6.798	3.448	2.562	tr|H0UXK3|H0UXK3_CAVPO	Uncharacterized protein OS=Cavia porcellus GN=EGFLAM PE=4 SV=1	CAVPO	2
2210	3.24	3.49	13.88	8.373	8.373	tr|H0UYT9|H0UYT9_CAVPO	Uncharacterized protein OS=Cavia porcellus GN=BCS1L PE=3 SV=1	CAVPO	3
2211	3.24	3.45	22.6	8.312	6.234	tr|H0V990|H0V990_CAVPO	Uncharacterized protein (Fragment) OS=Cavia porcellus GN=PRPF38B PE=4 SV=1	CAVPO	2
2212	3.24	3.32	9.296	7.789	5.151	tr|H0VFK6|H0VFK6_CAVPO	Uncharacterized protein (Fragment) OS=Cavia porcellus GN=SEL1L PE=4 SV=1	CAVPO	2
2213	3.23	3.75	8.514	4.452	1.781	tr|H0V1B3|H0V1B3_CAVPO	Uncharacterized protein (Fragment) OS=Cavia porcellus GN=PTPRD PE=4 SV=1	CAVPO	3
2214	3.22	3.49	17.08	14.99	5.123	tr|H0V9C6|H0V9C6_CAVPO	Uncharacterized protein (Fragment) OS=Cavia porcellus GN=HMGCS1 PE=4 SV=1	CAVPO	2
2215	3.22	3.51	13.76	8.734	5.24	tr|H0VIE8|H0VIE8_CAVPO	Uncharacterized protein (Fragment) OS=Cavia porcellus GN=SLC25A23 PE=3 SV=1	CAVPO	2
2216	3.22	3.29	7.796	5.376	5.376	tr|H0VIS9|H0VIS9_CAVPO	Uncharacterized protein (Fragment) OS=Cavia porcellus GN=LPGAT1 PE=4 SV=1	CAVPO	2
2217	3.22	3.29	18.75	18.75	18.75	tr|H0VXQ6|H0VXQ6_CAVPO	Uncharacterized protein OS=Cavia porcellus GN=RPL22 PE=4 SV=1	CAVPO	2
2218	3.21	3.28	8.798	8.798	8.798	tr|H0UWD6|H0UWD6_CAVPO	Uncharacterized protein OS=Cavia porcellus GN=PRRT2 PE=4 SV=1	CAVPO	2

2219	3.21	3.31	9.663	5.281	2.809	tr|H0VNQ8|H0VNQ8_CAVPO	Uncharacterized protein OS=Cavia porcellus GN=ERMP1 PE=4 SV=1	CAVPO	2
2220	3.2	3.43	35.05	19.59	8.763	tr|H0W169|H0W169_CAVPO	Uncharacterized protein OS=Cavia porcellus GN=RPS9 PE=3 SV=1	CAVPO	2
2221	3.19	12.2	29.78	24.26	16.91	tr|H0UTB5|H0UTB5_CAVPO	Uncharacterized protein OS=Cavia porcellus GN=PAK1 PE=4 SV=1	CAVPO	7
2222	3.19	3.25	29.86	15.97	15.97	tr|H0V641|H0V641_CAVPO	Uncharacterized protein OS=Cavia porcellus GN=CCDC58 PE=4 SV=1	CAVPO	2
2223	3.19	3.25	8.193	7.143	7.143	tr|H0VTJ3|H0VTJ3_CAVPO	Uncharacterized protein OS=Cavia porcellus GN=SHPK PE=4 SV=1	CAVPO	2
2224	3.18	3.26	21.49	17.11	12.72	tr|H0VKB1|H0VKB1_CAVPO	GTP:AMP phosphotransferase AK3, mitochondrial OS=Cavia porcellus GN=AK3 PE=3 SV=1	CAVPO	2
2225	3.18	25.9	16.57	13.76	11.34	tr|H0W3K3|H0W3K3_CAVPO	Calcium-transporting ATPase OS=Cavia porcellus GN=ATP2B2 PE=3 SV=1	CAVPO	14
2226	3.17	3.36	18.45	18.45	18.45	tr|H0VAX4|H0VAX4_CAVPO	Uncharacterized protein OS=Cavia porcellus GN=CABP4 PE=4 SV=1	CAVPO	4
2227	3.17	3.23	27.38	27.38	27.38	tr|H0VFK4|H0VFK4_CAVPO	Uncharacterized protein (Fragment) OS=Cavia porcellus GN=VMA21 PE=4 SV=1	CAVPO	2
2228	3.17	3.25	6.171	2.314	1.613	tr|H0VLQ2|H0VLQ2_CAVPO	Uncharacterized protein OS=Cavia porcellus GN=TRAPPC8 PE=4 SV=1	CAVPO	2
2229	3.15	3.26	7.475	4.87	3.398	tr|H0UTT2|H0UTT2_CAVPO	Uncharacterized protein OS=Cavia porcellus GN=Gria2 PE=4 SV=1	CAVPO	2
2230	3.15	3.4	29.75	17.85	5.263	tr|H0V1H6|H0V1H6_CAVPO	Uncharacterized protein OS=Cavia porcellus GN=Vps4a PE=3 SV=1	CAVPO	2
2231	3.15	5.46	57.49	34.82	24.29	tr|H0VLZ8|H0VLZ8_CAVPO	Uncharacterized protein (Fragment) OS=Cavia porcellus GN=PPA2 PE=4 SV=1	CAVPO	4
2232	3.15	3.27	13.68	12.42	5.474	tr|H0VX81|H0VX81_CAVPO	Uncharacterized protein OS=Cavia porcellus	CAVPO	2

							GN=PARS2 PE=3 SV=1		
2233	3.14	10.1	17.51	7.856	5.948	tr|H0V126|H0V126_CAVPO	Uncharacterized protein (Fragment) OS=Cavia porcellus GN=CUL4B PE=3 SV=1	CAVPO	5
2234	3.14	3.32	4.664	4.664	4.664	tr|H0VM74|H0VM74_CAVPO	Transporter (Fragment) OS=Cavia porcellus GN=SLC6A17 PE=3 SV=1	CAVPO	3
2235	3.14	3.21	31.28	24.17	24.17	tr|H0VNF8|H0VNF8_CAVPO	Uncharacterized protein OS=Cavia porcellus GN=SDF2 PE=4 SV=1	CAVPO	3
2236	3.13	3.32	23.01	20.35	17.26	tr|H0V9Q2|H0V9Q2_CAVPO	Uncharacterized protein OS=Cavia porcellus GN=SPCS2 PE=4 SV=1	CAVPO	3
2237	3.13	3.2	35.57	35.57	28.19	tr|H0W809|H0W809_CAVPO	Uncharacterized protein OS=Cavia porcellus GN=CARHSP1 PE=4 SV=1	CAVPO	2
2238	3.12	3.19	8.257	8.257	8.257	tr|H0UY91|H0UY91_CAVPO	Uncharacterized protein (Fragment) OS=Cavia porcellus GN=GET4 PE=4 SV=1	CAVPO	2
2239	3.12	3.2	27.73	27.73	27.73	tr|H0VLC2|H0VLC2_CAVPO	NADH dehydrogenase [ubiquinone] 1 subunit C2 OS=Cavia porcellus GN=Ndufc2 PE=3 SV=1	CAVPO	3
2240	3.12	3.31	10.76	3.553	2.194	tr|H0VPG0|H0VPG0_CAVPO	Uncharacterized protein (Fragment) OS=Cavia porcellus GN=C16orf62 PE=4 SV=1	CAVPO	2
2241	3.12	3.19	13.37	13.37	13.37	tr|H0WBL0|H0WBL0_CAVPO	Uncharacterized protein (Fragment) OS=Cavia porcellus GN=DUSP3 PE=4 SV=1	CAVPO	2
2242	3.09	3.24	21.82	11.6	1.796	tr|H0V6A2|H0V6A2_CAVPO	Oxysterol-binding protein (Fragment) OS=Cavia porcellus GN=OSBP PE=3 SV=1	CAVPO	1
2243	3.09	3.18	16.7	7.675	5.643	tr|H0VDD4|H0VDD4_CAVPO	Uncharacterized protein OS=Cavia porcellus GN=TBL2 PE=4 SV=1	CAVPO	2
2244	3.09	20.1	47.8	39.02	39.02	tr|H0W8M5|H0W8M5_CAVPO	Uncharacterized protein (Fragment) OS=Cavia porcellus PE=4 SV=1	CAVPO	19
2245	3.08	3.25	50	32.86	18.57	tr|H0VDL2|H0VDL2_CAVPO	Uncharacterized protein (Fragment) OS=Cavia porcellus GN=RPL38 PE=3 SV=1	CAVPO	2

2246	3.08	3.18	9.79	7.459	3.963	tr|H0VEI2|H0VEI2_CAVPO	Uncharacterized protein OS=Cavia porcellus GN=POFUT2 PE=4 SV=1	CAVPO	2
2247	3.08	3.25	13.47	8.367	4.898	tr|H0VQ38|H0VQ38_CAVPO	Uncharacterized protein (Fragment) OS=Cavia porcellus GN=ZC3HC1 PE=4 SV=1	CAVPO	2
2248	3.08	3.15	4.773	4.773	4.773	tr|H0VT01|H0VT01_CAVPO	Uncharacterized protein (Fragment) OS=Cavia porcellus GN=FNTB PE=4 SV=1	CAVPO	2
2249	3.08	3.14	6.931	6.931	6.931	tr|H0W301|H0W301_CAVPO	Queuine tRNA-ribosyltransferase OS=Cavia porcellus GN=QTRT1 PE=3 SV=1	CAVPO	2
2250	3.07	3.22	19.11	9.333	9.333	tr|H0VWZ9|H0VWZ9_CAVPO	Uncharacterized protein OS=Cavia porcellus GN=PSPH PE=4 SV=1	CAVPO	2
2251	3.07	3.17	11.25	4.861	2.917	tr|H0W9S8|H0W9S8_CAVPO	Uncharacterized protein (Fragment) OS=Cavia porcellus GN=EXOC8 PE=4 SV=1	CAVPO	2
2252	3.06	3.29	29.51	24.31	19.79	tr|H0V0R5|H0V0R5_CAVPO	Uncharacterized protein OS=Cavia porcellus GN=STX1A PE=3 SV=1	CAVPO	4
2253	3.06	3.19	16.41	12.83	3.448	tr|H0VHW2|H0VHW2_CAVPO	Uncharacterized protein OS=Cavia porcellus GN=MCCC1 PE=3 SV=1	CAVPO	2
2254	3.05	3.15	3.129	3.129	3.129	tr|H0V1N6|H0V1N6_CAVPO	Uncharacterized protein OS=Cavia porcellus GN=EPB41L5 PE=4 SV=1	CAVPO	2
2255	3.05	3.26	8.915	7.132	4.309	tr|H0VBK4|H0VBK4_CAVPO	Uncharacterized protein OS=Cavia porcellus GN=ARMC8 PE=4 SV=1	CAVPO	3
2256	3.04	3.17	31.73	14.42	7.692	tr|H0V2M8|H0V2M8_CAVPO	Uncharacterized protein (Fragment) OS=Cavia porcellus GN=GAR1 PE=4 SV=1	CAVPO	2
2257	3.04	3.15	10.14	7.134	2.879	tr|H0VTP2|H0VTP2_CAVPO	Uncharacterized protein OS=Cavia porcellus GN=STRN3 PE=4 SV=1	CAVPO	2
2258	3.03	3.1	40.91	40.91	40.91	tr|H0UT88|H0UT88_CAVPO	Uncharacterized protein (Fragment) OS=Cavia porcellus GN=EIF4EBP2 PE=4 SV=1	CAVPO	2
2259	3.03	3.13	8.686	5.345	5.345	tr|H0VEK1|H0VEK1_CAVPO	Uncharacterized protein OS=Cavia porcellus GN=NT5DC1 PE=4 SV=1	CAVPO	2

2260	3.03	5.25	43.81	20.95	16.67	tr|H0W717|H0W717_CAVPO	Uncharacterized protein (Fragment) OS=Cavia porcellus GN=RAB12 PE=3 SV=1	CAVPO	5
2261	3.03	7.34	35.02	29.95	29.95	tr|H0W7H0|H0W7H0_CAVPO	Uncharacterized protein (Fragment) OS=Cavia porcellus GN=TAGLN2 PE=4 SV=1	CAVPO	6
2262	3.03	9.72	41.08	41.08	34.05	tr|H0WCU1|H0WCU1_CAVPO	Uncharacterized protein OS=Cavia porcellus GN=RAP1A PE=4 SV=1	CAVPO	6
2263	3.02	3.11	17.53	13.51	7.471	tr|H0V5E2|H0V5E2_CAVPO	Uncharacterized protein OS=Cavia porcellus GN=LMAN2L PE=4 SV=1	CAVPO	2
2264	3.02	3.28	10.73	3.663	3.066	tr|H0VK30|H0VK30_CAVPO	Uncharacterized protein OS=Cavia porcellus GN=PLEKHA6 PE=4 SV=1	CAVPO	3
2265	3.02	3.09	10.9	4.135	4.135	tr|H0VP88|H0VP88_CAVPO	Uncharacterized protein OS=Cavia porcellus GN=ADCK4 PE=4 SV=1	CAVPO	2
2266	3.02	3.34	15.44	11.62	4.434	tr|H0VVY4|H0VVY4_CAVPO	Uncharacterized protein OS=Cavia porcellus GN=ACADVL PE=3 SV=1	CAVPO	3
2267	3.01	3.18	15.32	10.28	5.47	tr|H0UYT8|H0UYT8_CAVPO	Annexin OS=Cavia porcellus GN=ANXA7 PE=3 SV=1	CAVPO	2
2268	3.01	3.08	19.32	15.34	15.34	tr|H0V0H7|H0V0H7_CAVPO	Uncharacterized protein OS=Cavia porcellus GN=DR1 PE=4 SV=1	CAVPO	2
2269	3.01	3.17	7.063	2.194	1.231	tr|H0VA03|H0VA03_CAVPO	Uncharacterized protein (Fragment) OS=Cavia porcellus GN=ANK1 PE=4 SV=1	CAVPO	2
2270	3.01	3.12	21.59	13.64	9.091	tr|H0VLN6|H0VLN6_CAVPO	Uncharacterized protein OS=Cavia porcellus GN=VPS25 PE=4 SV=1	CAVPO	2
2271	3	3.14	14.97	14.97	14.97	tr|H0V6L9|H0V6L9_CAVPO	Uncharacterized protein OS=Cavia porcellus GN=TMEM35 PE=4 SV=1	CAVPO	2
2272	3	3.19	6.026	6.026	6.026	tr|H0VWW3|H0VWW3_CAVPO	Uncharacterized protein OS=Cavia porcellus GN=RIC8A PE=4 SV=1	CAVPO	3
2273	3	3.26	19.11	19.11	13.38	tr|H0VZ88|H0VZ88_CAVPO	Uncharacterized protein (Fragment) OS=Cavia porcellus GN=Rpl24 PE=4 SV=1	CAVPO	2

2274	2.99	3.05	23.73	23.73	23.73	tr|H0VI03|H0VI03_CAVPO	Uncharacterized protein OS=Cavia porcellus GN=FAM103A1 PE=4 SV=1	CAVPO	2
2275	2.99	3.13	37.32	27.54	8.696	tr|H0VJ55|H0VJ55_CAVPO	Uncharacterized protein OS=Cavia porcellus GN=FAM151B PE=4 SV=1	CAVPO	2
2276	2.99	3.05	18.68	12.45	12.45	tr|H0W2N0|H0W2N0_CAVPO	Uncharacterized protein (Fragment) OS=Cavia porcellus PE=4 SV=1	CAVPO	2
2277	2.98	3.15	9.235	5.277	5.277	tr|H0VMQ8|H0VMQ8_CAVPO	Uncharacterized protein (Fragment) OS=Cavia porcellus GN=LAMP2 PE=4 SV=1	CAVPO	2
2278	2.98	3.07	25.42	22.74	12.71	tr|H0VS52|H0VS52_CAVPO	Uncharacterized protein OS=Cavia porcellus GN=PEX19 PE=4 SV=1	CAVPO	2
2279	2.97	3.21	10.46	10.09	9.174	tr|H0V2E2|H0V2E2_CAVPO	Uncharacterized protein OS=Cavia porcellus GN=DCAF11 PE=4 SV=1	CAVPO	3
2280	2.97	3.3	45	25.62	18.13	tr|H0W4Y5|H0W4Y5_CAVPO	Uncharacterized protein (Fragment) OS=Cavia porcellus GN=EFHD2 PE=4 SV=1	CAVPO	3
2281	2.97	3.05	14	9.949	7.42	tr|H0WCG0|H0WCG0_CAVPO	Uncharacterized protein OS=Cavia porcellus GN=Bbs1 PE=4 SV=1	CAVPO	2
2282	2.96	3.07	8.04	8.04	6.533	tr|H0UT90|H0UT90_CAVPO	Uncharacterized protein (Fragment) OS=Cavia porcellus GN=ATG4B PE=4 SV=1	CAVPO	2
2283	2.95	17.9	43.87	41.6	35.33	tr|H0VF74|H0VF74_CAVPO	Uncharacterized protein (Fragment) OS=Cavia porcellus GN=PRKACB PE=4 SV=1	CAVPO	10
2284	2.95	5.52	20.65	14.72	6.135	tr|H0VGQ5|H0VGQ5_CAVPO	Uncharacterized protein OS=Cavia porcellus GN=Cdk9 PE=4 SV=1	CAVPO	3
2285	2.93	3.12	10.21	8.314	5.701	tr|H0UU62|H0UU62_CAVPO	Uncharacterized protein (Fragment) OS=Cavia porcellus GN=GLA PE=4 SV=1	CAVPO	2
2286	2.93	3	17.54	11.4	11.4	tr|H0UWC8|H0UWC8_CAVPO	Ribulose-phosphate 3-epimerase OS=Cavia porcellus GN=Rpe PE=3 SV=1	CAVPO	2
2287	2.93	3.19	9.012	6.914	2.716	tr|H0UYK3|H0UYK3_CAVPO	Uncharacterized protein (Fragment) OS=Cavia porcellus GN=TTC7B PE=4 SV=1	CAVPO	2

2288	2.93	3.17	20.08	13.12	4.573	tr|H0V027|H0V027_CAVPO	Uncharacterized protein OS=Cavia porcellus GN=PDHX PE=3 SV=1	CAVPO	2
2289	2.93	3.1	37.74	36.79	17.92	tr|H0V0C5|H0V0C5_CAVPO	Uncharacterized protein OS=Cavia porcellus GN=NDUFS5 PE=4 SV=1	CAVPO	3
2290	2.93	3.08	9.111	7.158	5.64	tr|H0V1Y3|H0V1Y3_CAVPO	Coronin OS=Cavia porcellus GN=CORO1A PE=3 SV=1	CAVPO	2
2291	2.93	3.03	10.25	5.95	4.793	tr|H0VHH3|H0VHH3_CAVPO	Uncharacterized protein OS=Cavia porcellus GN=SH3BP1 PE=4 SV=1	CAVPO	2
2292	2.93	3.26	27.66	24.62	9.898	tr|H0WD57|H0WD57_CAVPO	Uncharacterized protein OS=Cavia porcellus GN=ALKBH5 PE=4 SV=1	CAVPO	2
2293	2.91	2.99	6.861	2.922	2.922	tr|H0V1H4|H0V1H4_CAVPO	Uncharacterized protein (Fragment) OS=Cavia porcellus GN=PDXDC1 PE=3 SV=1	CAVPO	2
2294	2.91	3.01	11.58	11.38	4.391	tr|H0VB66|H0VB66_CAVPO	Uncharacterized protein OS=Cavia porcellus GN=TFCP2 PE=4 SV=1	CAVPO	2
2295	2.9	7.91	25.5	25.5	11.26	tr|H0V9Y2|H0V9Y2_CAVPO	Peptidyl-prolyl cis-trans isomerase E OS=Cavia porcellus GN=PPIE PE=3 SV=1	CAVPO	8
2296	2.9	2.98	25.27	19.23	15.38	tr|Q920K4|Q920K4_CAVPO	Ferritin OS=Cavia porcellus GN=Fth1 PE=2 SV=1	CAVPO	2
2297	2.89	2.96	12.4	9.69	9.69	tr|H0UW36|H0UW36_CAVPO	Uncharacterized protein OS=Cavia porcellus GN=Fam192a PE=4 SV=1	CAVPO	2
2298	2.89	2.99	32.69	16.67	11.54	tr|H0UZT4|H0UZT4_CAVPO	Uncharacterized protein OS=Cavia porcellus GN=NCBP2 PE=4 SV=1	CAVPO	2
2299	2.89	3.07	11.15	3.925	3.925	tr|H0VSA3|H0VSA3_CAVPO	Uncharacterized protein OS=Cavia porcellus GN=SRPR PE=4 SV=1	CAVPO	2
2300	2.88	2.94	19.19	13.8	13.8	tr|H0UUH5|H0UUH5_CAVPO	Uncharacterized protein OS=Cavia porcellus GN=CFDP1 PE=4 SV=1	CAVPO	3
2301	2.88	3.07	19.62	12.1	2.688	tr|H0V2X4|H0V2X4_CAVPO	Uncharacterized protein OS=Cavia porcellus GN=CDC73 PE=4 SV=1	CAVPO	1

2302	2.88	3.08	24.03	24.03	24.03	tr|H0V899|H0V899_CAVPO	Uncharacterized protein (Fragment) OS=Cavia porcellus GN=CHCHD4 PE=4 SV=1	CAVPO	3
2303	2.88	3.04	10.15	2.977	2.977	tr|H0VUW1|H0VUW1_CAVPO	Uncharacterized protein (Fragment) OS=Cavia porcellus GN=KIF3B PE=4 SV=1	CAVPO	2
2304	2.87	3.49	12.08	8.679	6.604	tr|H0VHJ3|H0VHJ3_CAVPO	Uncharacterized protein (Fragment) OS=Cavia porcellus GN=HEXA PE=4 SV=1	CAVPO	3
2305	2.87	3.01	14.29	9.524	9.524	tr|H0VKW1|H0VKW1_CAVPO	Uncharacterized protein (Fragment) OS=Cavia porcellus GN=TMED5 PE=3 SV=1	CAVPO	3
2306	2.87	3.15	26.13	21.62	14.86	tr|H0W786|H0W786_CAVPO	Uncharacterized protein OS=Cavia porcellus GN=CHMP2A PE=4 SV=1	CAVPO	3
2307	2.86	2.95	10.38	7.186	3.194	tr|H0V9Q3|H0V9Q3_CAVPO	Uncharacterized protein (Fragment) OS=Cavia porcellus GN=CDK5RAP3 PE=4 SV=1	CAVPO	2
2308	2.86	3.03	27.76	25.08	10.37	tr|H0VSL6|H0VSL6_CAVPO	Uncharacterized protein OS=Cavia porcellus GN=Rqcd1 PE=4 SV=1	CAVPO	2
2309	2.86	3.11	28.93	15.98	7.713	tr|H0WA77|H0WA77_CAVPO	Uncharacterized protein OS=Cavia porcellus GN=KIAA1045 PE=4 SV=1	CAVPO	2
2310	2.85	2.98	9.034	5.919	5.919	tr|H0VBJ7|H0VBJ7_CAVPO	Uncharacterized protein OS=Cavia porcellus GN=CD47 PE=4 SV=1	CAVPO	2
2311	2.85	3.04	11.64	6.119	4.478	tr|H0VIT3|H0VIT3_CAVPO	Uncharacterized protein (Fragment) OS=Cavia porcellus GN=PCCA PE=3 SV=1	CAVPO	2
2312	2.84	2.99	23.24	23.24	23.24	tr|H0VRI3|H0VRI3_CAVPO	Uncharacterized protein OS=Cavia porcellus GN=VAMP4 PE=4 SV=1	CAVPO	2
2313	2.84	2.95	12.21	4.469	0.993	tr|H0VT70|H0VT70_CAVPO	Uncharacterized protein (Fragment) OS=Cavia porcellus GN=ITGAV PE=3 SV=1	CAVPO	1
2314	2.83	2.92	5.351	2.085	0.7644	tr|H0V2T4|H0V2T4_CAVPO	Uncharacterized protein OS=Cavia porcellus GN=GAPVD1 PE=4 SV=1	CAVPO	1
2315	2.82	2.88	30.43	30.43	30.43	tr|H0W364|H0W364_CAVPO	Uncharacterized protein OS=Cavia porcellus GN=RPS28 PE=3 SV=1	CAVPO	2

2316	2.82	5.45	15.09	9.434	6.761	tr|H0W486|H0W486_CAVPO	Uncharacterized protein (Fragment) OS=Cavia porcellus GN=CNGA3 PE=4 SV=1	CAVPO	4
2317	2.82	2.92	17.72	12.86	3.883	tr|H0W8E5|H0W8E5_CAVPO	Uncharacterized protein OS=Cavia porcellus GN=FKBP8 PE=4 SV=1	CAVPO	1
2318	2.81	2.87	7.767	7.767	7.767	tr|H0W889|H0W889_CAVPO	Uncharacterized protein (Fragment) OS=Cavia porcellus GN=ZADH2 PE=4 SV=1	CAVPO	2
2319	2.81	2.88	42.86	41.07	17.86	tr|H0W9V1|H0W9V1_CAVPO	60S ribosomal protein L27 (Fragment) OS=Cavia porcellus GN=RPL27 PE=3 SV=1	CAVPO	2
2320	2.8	2.94	7.769	7.769	7.769	tr|H0V0G2|H0V0G2_CAVPO	Uncharacterized protein (Fragment) OS=Cavia porcellus GN=POFUT1 PE=4 SV=1	CAVPO	3
2321	2.8	3.05	17.11	8.48	3.509	tr|H0VIV6|H0VIV6_CAVPO	Uncharacterized protein OS=Cavia porcellus GN=CPSF3 PE=4 SV=1	CAVPO	2
2322	2.8	4.83	5.019	3.726	2.51	tr|H0VN26|H0VN26_CAVPO	Uncharacterized protein OS=Cavia porcellus GN=GAK PE=4 SV=1	CAVPO	3
2323	2.8	3.01	7.224	5.524	4.108	tr|H0VT85|H0VT85_CAVPO	Uncharacterized protein (Fragment) OS=Cavia porcellus GN=PRMT7 PE=4 SV=1	CAVPO	3
2324	2.79	2.94	15.73	9.363	9.363	tr|H0V732|H0V732_CAVPO	Hydroxypyruvate isomerase OS=Cavia porcellus GN=HYI PE=3 SV=1	CAVPO	2
2325	2.79	3	9.872	5.641	5.641	tr|H0VKU6|H0VKU6_CAVPO	Uncharacterized protein OS=Cavia porcellus GN=STRN PE=4 SV=1	CAVPO	3
2326	2.79	2.88	32.1	23.46	15.43	tr|H0W8L3|H0W8L3_CAVPO	Uncharacterized protein OS=Cavia porcellus GN=Btf3 PE=4 SV=1	CAVPO	1
2327	2.78	2.84	10.45	7.666	7.666	tr|H0V2G2|H0V2G2_CAVPO	Uncharacterized protein (Fragment) OS=Cavia porcellus GN=TTC1 PE=4 SV=1	CAVPO	2
2328	2.78	2.96	5.443	2.582	1.954	tr|H0VK33|H0VK33_CAVPO	Uncharacterized protein (Fragment) OS=Cavia porcellus GN=BAZ1B PE=4 SV=1	CAVPO	2
2329	2.77	2.95	11.9	9.762	9.762	tr|H0VDC7|H0VDC7_CAVPO	Uncharacterized protein OS=Cavia porcellus GN=ACAA1 PE=3 SV=1	CAVPO	3

2330	2.77	2.92	13.12	6.807	3.837	tr|H0VLE5|H0VLE5_CAVPO	Uncharacterized protein OS=Cavia porcellus GN=Zc3h11a PE=4 SV=1	CAVPO	2
2331	2.76	2.89	7.692	7.692	7.692	tr|H0V487|H0V487_CAVPO	Uncharacterized protein OS=Cavia porcellus GN=MPDU1 PE=4 SV=1	CAVPO	2
2332	2.76	2.99	7.174	2.343	1.098	tr|H0VKL2|H0VKL2_CAVPO	Uncharacterized protein OS=Cavia porcellus GN=TNIK PE=4 SV=1	CAVPO	2
2333	2.75	2.81	12.02	7.625	7.625	tr|H0VA66|H0VA66_CAVPO	Uncharacterized protein OS=Cavia porcellus GN=DHRS13 PE=4 SV=1	CAVPO	2
2334	2.75	2.89	12.13	8.787	8.787	tr|H0VRT5|H0VRT5_CAVPO	Uncharacterized protein OS=Cavia porcellus GN=PSME2 PE=4 SV=1	CAVPO	2
2335	2.75	2.81	4.038	4.038	4.038	tr|H0VUG3|H0VUG3_CAVPO	Uncharacterized protein OS=Cavia porcellus GN=DYNC1I1 PE=4 SV=1	CAVPO	2
2336	2.74	2.9	13.85	6.24	2.892	tr|H0UV41|H0UV41_CAVPO	Uncharacterized protein OS=Cavia porcellus GN=THOC1 PE=4 SV=1	CAVPO	2
2337	2.74	2.81	6.366	6.366	4.107	tr|H0UXZ3|H0UXZ3_CAVPO	Uncharacterized protein (Fragment) OS=Cavia porcellus GN=GDA PE=4 SV=1	CAVPO	2
2338	2.74	2.8	6.785	6.785	6.785	tr|H0UZ87|H0UZ87_CAVPO	Uncharacterized protein OS=Cavia porcellus GN=CLYBL PE=4 SV=1	CAVPO	2
2339	2.74	3.11	25.85	22.93	8.293	tr|H0VN30|H0VN30_CAVPO	Uncharacterized protein (Fragment) OS=Cavia porcellus GN=HDDC2 PE=4 SV=1	CAVPO	1
2340	2.73	2.95	16.72	11.67	6.309	tr|H0UZS3|H0UZS3_CAVPO	Uncharacterized protein OS=Cavia porcellus GN=PRKAG1 PE=4 SV=1	CAVPO	2
2341	2.73	2.81	40.71	30	30	tr|H0V5R4|H0V5R4_CAVPO	Uncharacterized protein OS=Cavia porcellus GN=PTRHD1 PE=4 SV=1	CAVPO	2
2342	2.73	3.11	13.61	11.91	5.671	tr|H0V6C3|H0V6C3_CAVPO	Uncharacterized protein (Fragment) OS=Cavia porcellus GN=SNTB1 PE=4 SV=1	CAVPO	3
2343	2.72	2.92	31.62	31.62	12.82	tr|H0VNL2|H0VNL2_CAVPO	Uncharacterized protein OS=Cavia porcellus GN=GABARAPL2 PE=3 SV=1	CAVPO	2

2344	2.72	2.97	6.855	4.637	4.637	tr|H0VNP5|H0VNP5_CAVPO	Uncharacterized protein OS=Cavia porcellus GN=DLGAP4 PE=4 SV=1	CAVPO	4
2345	2.71	2.77	34.57	23.46	23.46	tr|H0V0R1|H0V0R1_CAVPO	Uncharacterized protein OS=Cavia porcellus GN=ABRACL PE=4 SV=1	CAVPO	3
2346	2.71	2.87	19.79	10.93	4.33	tr|H0V4N8|H0V4N8_CAVPO	Uncharacterized protein OS=Cavia porcellus GN=WDR13 PE=4 SV=1	CAVPO	2
2347	2.71	2.85	12.97	9.73	9.73	tr|H0V6H0|H0V6H0_CAVPO	Uncharacterized protein OS=Cavia porcellus GN=PTGES2 PE=3 SV=1	CAVPO	2
2348	2.71	2.77	47.87	39.36	31.91	tr|H0VZ22|H0VZ22_CAVPO	Uncharacterized protein (Fragment) OS=Cavia porcellus GN=LSM2 PE=4 SV=1	CAVPO	2
2349	2.7	2.76	18.01	9.962	9.962	tr|H0UV59|H0UV59_CAVPO	Uncharacterized protein OS=Cavia porcellus GN=C1orf35 PE=4 SV=1	CAVPO	2
2350	2.7	2.76	17.62	17.62	12.95	tr|H0V9I9|H0V9I9_CAVPO	Uncharacterized protein OS=Cavia porcellus GN=AP3S1 PE=4 SV=1	CAVPO	2
2351	2.7	2.9	21.63	16.11	7.692	tr|H0VCL7|H0VCL7_CAVPO	Uncharacterized protein OS=Cavia porcellus GN=ACTR10 PE=3 SV=1	CAVPO	2
2352	2.69	11.8	27.76	24.94	14.59	tr|H0VR65|H0VR65_CAVPO	Uncharacterized protein OS=Cavia porcellus GN=RBBP7 PE=4 SV=1	CAVPO	7
2353	2.68	2.73	8.911	8.911	8.911	tr|H0W8X0|H0W8X0_CAVPO	Uncharacterized protein OS=Cavia porcellus GN=RNF126 PE=4 SV=1	CAVPO	2
2354	2.67	2.82	5.689	5.689	5.689	tr|H0VFA2|H0VFA2_CAVPO	Uncharacterized protein OS=Cavia porcellus GN=HSD17B7 PE=4 SV=1	CAVPO	2
2355	2.66	2.8	6.602	3.851	2.889	tr|H0UTD8|H0UTD8_CAVPO	Uncharacterized protein OS=Cavia porcellus GN=GPD2 PE=3 SV=1	CAVPO	2
2356	2.66	4.46	22.38	13.81	8.84	tr|H0UZG7|H0UZG7_CAVPO	Uncharacterized protein (Fragment) OS=Cavia porcellus GN=TMOD3 PE=4 SV=1	CAVPO	3
2357	2.66	2.84	5.371	2.16	1.401	tr|H0V1R3|H0V1R3_CAVPO	Uncharacterized protein OS=Cavia porcellus GN=CDC42BPB PE=4 SV=1	CAVPO	2

2358	2.66	5.08	23.78	17.59	14.98	tr|H0V212|H0V212_CAVPO	Serine/threonine-protein phosphatase OS=Cavia porcellus GN=PPP4C PE=3 SV=1	CAVPO	3
2359	2.66	2.84	38.62	26.21	15.86	tr|H0W6X3|H0W6X3_CAVPO	Uncharacterized protein (Fragment) OS=Cavia porcellus GN=Fam136a PE=4 SV=1	CAVPO	2
2360	2.66	2.71	25.35	25.35	25.35	tr|H0WAV0|H0WAV0_CAVPO	Protein kish (Fragment) OS=Cavia porcellus GN=TMEM167A PE=3 SV=1	CAVPO	2
2361	2.65	8.85	29.87	19.25	11.95	tr|H0UWX4|H0UWX4_CAVPO	Serine/threonine-protein phosphatase 2A 55 kDa regulatory subunit B (Fragment) OS=Cavia porcellus GN=PPP2R2D PE=3 SV=1	CAVPO	5
2362	2.65	2.81	17.12	6.849	5.308	tr|H0V832|H0V832_CAVPO	Uncharacterized protein (Fragment) OS=Cavia porcellus GN=EIF2A PE=4 SV=1	CAVPO	2
2363	2.65	2.72	21.7	21.7	9.906	tr|H0WAW0|H0WAW0_CAVPO	Uncharacterized protein OS=Cavia porcellus GN=DRAP1 PE=4 SV=1	CAVPO	2
2364	2.64	2.83	10.28	3.72	2.626	tr|H0UV06|H0UV06_CAVPO	Anoctamin OS=Cavia porcellus GN=ANO2 PE=3 SV=1	CAVPO	2
2365	2.64	2.7	21.96	14.12	9.804	tr|H0VJG3|H0VJG3_CAVPO	Uncharacterized protein OS=Cavia porcellus GN=STX6 PE=4 SV=1	CAVPO	2
2366	2.63	2.74	8.68	5.257	1.711	tr|H0VDM9|H0VDM9_CAVPO	Uncharacterized protein (Fragment) OS=Cavia porcellus GN=EFR3A PE=4 SV=1	CAVPO	1
2367	2.63	2.76	28.87	19.59	19.59	tr|H0VKR6|H0VKR6_CAVPO	Uncharacterized protein (Fragment) OS=Cavia porcellus GN=SUMO1 PE=4 SV=1	CAVPO	2
2368	2.63	2.7	47.37	47.37	44.21	tr|H0VQ54|H0VQ54_CAVPO	Uncharacterized protein OS=Cavia porcellus GN=Fxyd6 PE=4 SV=1	CAVPO	2
2369	2.62	2.68	6.086	3.181	3.181	tr|H0VAY4|H0VAY4_CAVPO	Uncharacterized protein OS=Cavia porcellus GN=PIK3R1 PE=4 SV=1	CAVPO	2
2370	2.62	2.67	6.953	3.254	3.254	tr|H0VLA4|H0VLA4_CAVPO	Uncharacterized protein OS=Cavia porcellus GN=PTCD3 PE=4 SV=1	CAVPO	2
2371	2.62	2.67	31.11	22.22	22.22	tr|H0W599|H0W599_CAVPO	Uncharacterized protein OS=Cavia porcellus	CAVPO	2

							GN=TIMM10 PE=4 SV=1		
2372	2.62	2.71	4.808	3.486	2.524	tr|H0WBV5|H0WBV5_CAVPO	Uncharacterized protein OS=Cavia porcellus GN=TMEM63B PE=4 SV=1	CAVPO	2
2373	2.61	2.74	6.376	3.326	2.98	tr|H0V776|H0V776_CAVPO	Uncharacterized protein (Fragment) OS=Cavia porcellus GN=VPRBP PE=4 SV=1	CAVPO	2
2374	2.61	2.83	17.18	12.02	7.634	tr|H0V9U1|H0V9U1_CAVPO	Uncharacterized protein (Fragment) OS=Cavia porcellus GN=WDR26 PE=4 SV=1	CAVPO	3
2375	2.6	2.65	19.3	9.81	9.81	tr|H0VCR4|H0VCR4_CAVPO	Uncharacterized protein (Fragment) OS=Cavia porcellus GN=ABHD11 PE=4 SV=1	CAVPO	2
2376	2.6	5.03	21.96	21.96	18.04	tr|H0VQW3|H0VQW3_CAVPO	Uncharacterized protein OS=Cavia porcellus GN=UNC119B PE=4 SV=1	CAVPO	3
2377	2.59	2.63	26.51	26.51	26.51	tr|H0VVE2|H0VVE2_CAVPO	Uncharacterized protein (Fragment) OS=Cavia porcellus GN=NDUFB4 PE=4 SV=1	CAVPO	2
2378	2.58	2.63	6.43	5.322	1.441	tr|H0VBG8|H0VBG8_CAVPO	Uncharacterized protein OS=Cavia porcellus GN=LARS2 PE=3 SV=1	CAVPO	1
2379	2.58	2.64	7.895	3.684	2.211	tr|H0VEJ7|H0VEJ7_CAVPO	Uncharacterized protein OS=Cavia porcellus GN=XRN2 PE=4 SV=1	CAVPO	2
2380	2.58	2.73	9.066	9.066	6.319	tr|H0VHY0|H0VHY0_CAVPO	Uncharacterized protein (Fragment) OS=Cavia porcellus GN=Atxn3 PE=4 SV=1	CAVPO	2
2381	2.58	2.63	7.216	4.742	4.742	tr|H0W1Q8|H0W1Q8_CAVPO	Uncharacterized protein (Fragment) OS=Cavia porcellus GN=MTMR1 PE=4 SV=1	CAVPO	2
2382	2.58	2.7	5.321	3.274	3.274	tr|H0WB99|H0WB99_CAVPO	Uncharacterized protein OS=Cavia porcellus GN=IRF2BPL PE=4 SV=1	CAVPO	2
2383	2.57	2.61	7.167	7.167	7.167	tr|H0V6E3|H0V6E3_CAVPO	Uncharacterized protein OS=Cavia porcellus GN=MSMO1 PE=4 SV=1	CAVPO	2
2384	2.57	2.63	15.91	9.596	3.535	tr|H0VEX5|H0VEX5_CAVPO	Uncharacterized protein OS=Cavia porcellus GN=CHID1 PE=3 SV=1	CAVPO	1

2385	2.57	2.64	7.233	1.766	1.766	tr|H0VRG4|H0VRG4_CAVPO	Uncharacterized protein OS=Cavia porcellus GN=DAB2IP PE=4 SV=1	CAVPO	2
2386	2.56	2.61	4.848	3.03	3.03	tr|H0V845|H0V845_CAVPO	Uncharacterized protein OS=Cavia porcellus GN=SLC7A5 PE=4 SV=1	CAVPO	2
2387	2.56	2.62	25.48	22.12	22.12	tr|H0VCW8|H0VCW8_CAVPO	Uncharacterized protein OS=Cavia porcellus GN=CRIP2 PE=4 SV=1	CAVPO	2
2388	2.55	2.65	6.217	1.421	0.8289	tr|H0UTK3|H0UTK3_CAVPO	Uncharacterized protein OS=Cavia porcellus GN=PBRM1 PE=4 SV=1	CAVPO	1
2389	2.55	2.85	19.62	15.26	8.719	tr|H0VHP6|H0VHP6_CAVPO	Uncharacterized protein OS=Cavia porcellus GN=DRG1 PE=4 SV=1	CAVPO	3
2390	2.55	2.61	13.05	9.071	4.425	tr|H0VVK3|H0VVK3_CAVPO	Uncharacterized protein (Fragment) OS=Cavia porcellus GN=PPM1F PE=3 SV=1	CAVPO	1
2391	2.54	2.61	18	16.29	10.29	tr|H0UUM8|H0UUM8_CAVPO	Uncharacterized protein OS=Cavia porcellus GN=ATP6AP2 PE=4 SV=1	CAVPO	3
2392	2.54	2.58	12.34	8.442	8.442	tr|H0VLX3|H0VLX3_CAVPO	Uncharacterized protein OS=Cavia porcellus GN=SAP30BP PE=4 SV=1	CAVPO	2
2393	2.53	2.58	16.31	8.873	6.475	tr|H0W9H9|H0W9H9_CAVPO	Uncharacterized protein (Fragment) OS=Cavia porcellus GN=ALDH5A1 PE=3 SV=1	CAVPO	2
2394	2.52	2.68	5.74	2.189	1.479	tr|H0UYC9|H0UYC9_CAVPO	Uncharacterized protein OS=Cavia porcellus GN=KDM3B PE=4 SV=1	CAVPO	2
2395	2.52	2.6	21.4	18.6	6.512	tr|H0UYD1|H0UYD1_CAVPO	Uncharacterized protein OS=Cavia porcellus GN=RPL10 PE=4 SV=1	CAVPO	2
2396	2.52	2.56	15.04	10.18	10.18	tr|H0W603|H0W603_CAVPO	Uncharacterized protein OS=Cavia porcellus GN=OVCA2 PE=4 SV=1	CAVPO	2
2397	2.51	2.55	40	21.38	21.38	tr|H0V238|H0V238_CAVPO	Uncharacterized protein (Fragment) OS=Cavia porcellus GN=Rps15 PE=3 SV=1	CAVPO	3
2398	2.51	2.58	19.65	10.95	3.234	tr|H0VIZ2|H0VIZ2_CAVPO	Uncharacterized protein (Fragment) OS=Cavia porcellus GN=CPOX PE=4 SV=1	CAVPO	1

2399	2.5	2.54	26.41	14.72	10.39	tr|H0UYT6|H0UYT6_CAVPO	Uncharacterized protein OS=Cavia porcellus GN=STX7 PE=3 SV=1	CAVPO	2
2400	2.5	2.72	22.48	14.52	5.621	tr|H0VKN1|H0VKN1_CAVPO	Uncharacterized protein OS=Cavia porcellus GN=FLOT1 PE=4 SV=1	CAVPO	2
2401	2.5	2.62	3.374	1.018	1.018	tr|H0VQ95|H0VQ95_CAVPO	Uncharacterized protein OS=Cavia porcellus GN=SBF1 PE=4 SV=1	CAVPO	2
2402	2.49	2.59	12.31	10.21	10.21	tr|H0VE49|H0VE49_CAVPO	Uncharacterized protein OS=Cavia porcellus GN=CTSV PE=3 SV=1	CAVPO	2
2403	2.49	2.53	15.58	7.609	4.71	tr|H0VN65|H0VN65_CAVPO	Uncharacterized protein OS=Cavia porcellus GN=CCS PE=4 SV=1	CAVPO	1
2404	2.49	2.61	7.857	4.762	4.762	tr|H0W0K2|H0W0K2_CAVPO	Uncharacterized protein OS=Cavia porcellus GN=NUCB2 PE=4 SV=1	CAVPO	2
2405	2.48	2.54	14.66	9.971	2.639	tr|H0V4D2|H0V4D2_CAVPO	Uncharacterized protein OS=Cavia porcellus GN=Bbs5 PE=4 SV=1	CAVPO	1
2406	2.48	2.52	17.54	17.54	17.54	tr|H0VAS5|H0VAS5_CAVPO	Uncharacterized protein (Fragment) OS=Cavia porcellus GN=COPZ1 PE=4 SV=1	CAVPO	2
2407	2.48	2.61	10.42	8.958	3.125	tr|H0VLA0|H0VLA0_CAVPO	Uncharacterized protein (Fragment) OS=Cavia porcellus GN=RBM42 PE=4 SV=1	CAVPO	2
2408	2.48	2.52	8.056	7.778	7.778	tr|H0VPQ3|H0VPQ3_CAVPO	Uncharacterized protein (Fragment) OS=Cavia porcellus GN=WBP4 PE=4 SV=1	CAVPO	2
2409	2.48	2.54	15.84	11.17	2.857	tr|H0VY96|H0VY96_CAVPO	Uncharacterized protein OS=Cavia porcellus GN=PELO PE=4 SV=1	CAVPO	1
2410	2.47	2.63	11.92	7.277	3.137	tr|H0V676|H0V676_CAVPO	Uncharacterized protein OS=Cavia porcellus GN=TRAPPC12 PE=4 SV=1	CAVPO	2
2411	2.47	2.52	9.063	4.455	1.382	tr|H0VFE9|H0VFE9_CAVPO	Uncharacterized protein (Fragment) OS=Cavia porcellus GN=ADRBK2 PE=3 SV=1	CAVPO	1
2412	2.47	2.58	14.91	14.91	14.91	tr|H0WBW8|H0WBW8_CAVPO	Uncharacterized protein OS=Cavia porcellus GN=LOC100726034 PE=4 SV=1	CAVPO	2

2413	2.46	2.88	9.829	7.479	4.487	tr|H0VJF1|H0VJF1_CAVPO	Uncharacterized protein OS=Cavia porcellus GN=C16orf58 PE=4 SV=1	CAVPO	2
2414	2.46	2.52	9.399	8.616	3.655	tr|H0VLL0|H0VLL0_CAVPO	Ubiquitin carboxyl-terminal hydrolase (Fragment) OS=Cavia porcellus GN=USP10 PE=3 SV=1	CAVPO	2
2415	2.46	2.61	6.502	4.161	2.471	tr|H0VSL8|H0VSL8_CAVPO	Phosphoinositide phospholipase C (Fragment) OS=Cavia porcellus GN=PLCD4 PE=4 SV=1	CAVPO	2
2416	2.45	2.58	35.63	29.89	18.97	tr|H0V9K8|H0V9K8_CAVPO	Uncharacterized protein OS=Cavia porcellus GN=C20orf27 PE=4 SV=1	CAVPO	2
2417	2.45	2.49	23.73	14.69	14.69	tr|H0VII1|H0VII1_CAVPO	Uncharacterized protein OS=Cavia porcellus GN=H1F0 PE=3 SV=1	CAVPO	3
2418	2.45	2.5	9.237	6.225	2.209	tr|H0VLS4|H0VLS4_CAVPO	Uncharacterized protein OS=Cavia porcellus GN=TH PE=4 SV=1	CAVPO	1
2419	2.45	2.52	7.419	5.806	2.473	tr|H0VZQ5|H0VZQ5_CAVPO	Uncharacterized protein (Fragment) OS=Cavia porcellus GN=SCAF1 PE=4 SV=1	CAVPO	2
2420	2.43	2.47	40	23.53	9.412	tr|H0UVF7|H0UVF7_CAVPO	Uncharacterized protein (Fragment) OS=Cavia porcellus GN=RRAS2 PE=4 SV=1	CAVPO	1
2421	2.43	2.48	31.2	24.8	18.4	tr|H0VWJ2|H0VWJ2_CAVPO	Uncharacterized protein OS=Cavia porcellus GN=RPL31 PE=4 SV=1	CAVPO	2
2422	2.42	2.68	8.195	3.424	0.9234	tr|H0V7Q2|H0V7Q2_CAVPO	Uncharacterized protein OS=Cavia porcellus GN=GOLGB1 PE=4 SV=1	CAVPO	2
2423	2.42	2.54	11.99	3.894	3.894	tr|H0VZ66|H0VZ66_CAVPO	Uncharacterized protein (Fragment) OS=Cavia porcellus GN=RECQL PE=4 SV=1	CAVPO	2
2424	2.41	2.89	28.66	15.31	12.7	tr|H0VGB4|H0VGB4_CAVPO	Uncharacterized protein OS=Cavia porcellus GN=AKR1E2 PE=4 SV=1	CAVPO	5
2425	2.41	2.45	57.14	26.79	26.79	tr|H0VVM0|H0VVM0_CAVPO	Uncharacterized protein OS=Cavia porcellus GN=ARPP19 PE=4 SV=1	CAVPO	2
2426	2.4	2.44	5.546	3.578	1.431	tr|H0V2D3|H0V2D3_CAVPO	Uncharacterized protein OS=Cavia porcellus	CAVPO	1

							GN=WASF1 PE=4 SV=1		
2427	2.4	2.51	8.556	8.556	8.556	tr|H0V2Z1|H0V2Z1_CAVPO	Uncharacterized protein OS=Cavia porcellus GN=GPN1 PE=4 SV=1	CAVPO	2
2428	2.4	2.6	54.43	24.05	8.861	tr|H0VDD7|H0VDD7_CAVPO	Uncharacterized protein OS=Cavia porcellus GN=TRAPPC6B PE=4 SV=1	CAVPO	1
2429	2.4	2.53	16.99	16.99	16.99	tr|H0VDV8|H0VDV8_CAVPO	Uncharacterized protein OS=Cavia porcellus GN=ORMDL2 PE=4 SV=1	CAVPO	2
2430	2.4	2.54	10.46	6.971	3.351	tr|H0VS86|H0VS86_CAVPO	Uncharacterized protein OS=Cavia porcellus GN=WDR91 PE=4 SV=1	CAVPO	2
2431	2.39	2.45	11.14	6.627	4.217	tr|H0V0L1|H0V0L1_CAVPO	Uncharacterized protein (Fragment) OS=Cavia porcellus GN=PARVA PE=4 SV=1	CAVPO	1
2432	2.39	2.54	17.19	11.31	7.014	tr|H0V390|H0V390_CAVPO	Uncharacterized protein OS=Cavia porcellus GN=Acot2 PE=4 SV=1	CAVPO	3
2433	2.39	2.45	6.438	1.793	0.978	tr|H0VW81|H0VW81_CAVPO	Uncharacterized protein (Fragment) OS=Cavia porcellus GN=BCR PE=4 SV=1	CAVPO	1
2434	2.38	2.54	12.71	10.7	3.846	tr|H0UWD7|H0UWD7_CAVPO	Uncharacterized protein OS=Cavia porcellus GN=NR2C2 PE=3 SV=1	CAVPO	2
2435	2.38	2.53	8.215	3.688	0.922	tr|H0VCM5|H0VCM5_CAVPO	Uncharacterized protein (Fragment) OS=Cavia porcellus GN=CUX1 PE=4 SV=1	CAVPO	1
2436	2.38	2.44	21.1	13.99	6.193	tr|H0VDG8|H0VDG8_CAVPO	Uncharacterized protein (Fragment) OS=Cavia porcellus GN=SREK1 PE=4 SV=1	CAVPO	2
2437	2.38	2.43	7.426	6.271	1.32	tr|H0VQE2|H0VQE2_CAVPO	Uncharacterized protein OS=Cavia porcellus GN=GPR37 PE=4 SV=1	CAVPO	1
2438	2.38	2.46	19.85	15.46	3.866	tr|H0VSH9|H0VSH9_CAVPO	Uncharacterized protein (Fragment) OS=Cavia porcellus GN=INPP5A PE=4 SV=1	CAVPO	1
2439	2.37	2.44	30.29	19.92	4.149	tr|H0V2J9|H0V2J9_CAVPO	Uncharacterized protein OS=Cavia porcellus GN=EMC7 PE=4 SV=1	CAVPO	1

2440	2.37	2.41	13.17	9.16	4.58	tr|H0VPK5|H0VPK5_CAVPO	Uncharacterized protein (Fragment) OS=Cavia porcellus PE=4 SV=1	CAVPO	2
2441	2.36	6.12	25.25	25.25	19.7	tr|H0VRN5|H0VRN5_CAVPO	Uncharacterized protein OS=Cavia porcellus GN=SAR1B PE=3 SV=1	CAVPO	4
2442	2.36	2.49	27.35	21.37	21.37	tr|H0VWS5|H0VWS5_CAVPO	Transcription elongation factor SPT4 OS=Cavia porcellus GN=SUPT4H1 PE=3 SV=1	CAVPO	2
2443	2.36	2.4	47	18.5	8	tr|H0W240|H0W240_CAVPO	Uncharacterized protein OS=Cavia porcellus GN=NUDT16 PE=4 SV=1	CAVPO	1
2444	2.36	2.4	20.52	19.65	6.55	tr|H0W4Y6|H0W4Y6_CAVPO	Synaptogyrin OS=Cavia porcellus GN=SYNGR3 PE=3 SV=1	CAVPO	1
2445	2.34	21.1	50	47.08	47.08	tr|H0V4U1|H0V4U1_CAVPO	Serine/threonine-protein phosphatase OS=Cavia porcellus GN=PPP2CB PE=3 SV=1	CAVPO	13
2446	2.34	2.66	14.08	8.169	3.662	tr|H0VA56|H0VA56_CAVPO	Uncharacterized protein (Fragment) OS=Cavia porcellus GN=HOMER1 PE=4 SV=1	CAVPO	1
2447	2.34	2.38	7.882	6.897	3.941	tr|H0VGH2|H0VGH2_CAVPO	Uncharacterized protein OS=Cavia porcellus GN=TAF15 PE=4 SV=1	CAVPO	2
2448	2.34	2.38	28.18	20.91	6.364	tr|H0VTD8|H0VTD8_CAVPO	Uncharacterized protein OS=Cavia porcellus GN=RPL35A PE=4 SV=1	CAVPO	1
2449	2.33	2.36	11.11	11.11	11.11	tr|H0V3P5|H0V3P5_CAVPO	Uncharacterized protein (Fragment) OS=Cavia porcellus GN=SNRPD1 PE=4 SV=1	CAVPO	1
2450	2.33	2.36	7.774	7.774	7.774	tr|H0V3W0|H0V3W0_CAVPO	Uncharacterized protein OS=Cavia porcellus GN=UBXN1 PE=4 SV=1	CAVPO	2
2451	2.33	2.36	12.08	9.434	6.038	tr|H0VZM9|H0VZM9_CAVPO	Uncharacterized protein OS=Cavia porcellus GN=AQP5 PE=3 SV=1	CAVPO	1
2452	2.33	2.36	8.759	8.759	2.676	tr|H0WBR9|H0WBR9_CAVPO	Uncharacterized protein (Fragment) OS=Cavia porcellus GN=NAGA PE=4 SV=1	CAVPO	1
2453	2.32	2.36	38.85	19.42	9.353	tr|H0UZ92|H0UZ92_CAVPO	Uncharacterized protein (Fragment) OS=Cavia porcellus GN=TRAPPC2L PE=4 SV=1	CAVPO	1

2454	2.32	2.35	15.71	9.286	8.571	tr|H0UZP2|H0UZP2_CAVPO	Peptidyl-prolyl cis-trans isomerase OS=Cavia porcellus GN=FKBP2 PE=4 SV=1	CAVPO	1
2455	2.32	2.44	7.627	5.085	5.085	tr|H0VXH0|H0VXH0_CAVPO	Uncharacterized protein (Fragment) OS=Cavia porcellus GN=IRF2BP1 PE=4 SV=1	CAVPO	2
2456	2.32	2.39	9.704	3.618	2.138	tr|H0W8X9|H0W8X9_CAVPO	Uncharacterized protein OS=Cavia porcellus GN=EPM2AIP1 PE=4 SV=1	CAVPO	1
2457	2.31	2.44	18.64	10.08	10.08	tr|H0VJ98|H0VJ98_CAVPO	Uncharacterized protein OS=Cavia porcellus GN=VPS37A PE=4 SV=1	CAVPO	3
2458	2.31	2.43	4.501	3.001	2.207	tr|H0VJL4|H0VJL4_CAVPO	Uncharacterized protein OS=Cavia porcellus GN=TRAPPC11 PE=4 SV=1	CAVPO	2
2459	2.3	2.38	7.882	7.882	4.926	tr|H0VLT5|H0VLT5_CAVPO	Uncharacterized protein OS=Cavia porcellus GN=ARL6IP1 PE=4 SV=1	CAVPO	1
2460	2.29	5.8	8.569	4.409	3.161	tr|H0V5Q5|H0V5Q5_CAVPO	Uncharacterized protein OS=Cavia porcellus GN=PPFIA1 PE=4 SV=1	CAVPO	3
2461	2.29	2.63	6.81	3.853	2.151	tr|H0VCZ3|H0VCZ3_CAVPO	Uncharacterized protein OS=Cavia porcellus GN=ERC1 PE=4 SV=1	CAVPO	2
2462	2.29	2.41	2.957	1.163	1.163	tr|H0VDJ5|H0VDJ5_CAVPO	Uncharacterized protein OS=Cavia porcellus GN=MPDZ PE=4 SV=1	CAVPO	2
2463	2.29	2.32	12.93	5.122	3.171	tr|H0W5Z0|H0W5Z0_CAVPO	Uncharacterized protein OS=Cavia porcellus GN=AUP1 PE=4 SV=1	CAVPO	1
2464	2.28	2.44	13.81	9.524	4.524	tr|H0UX61|H0UX61_CAVPO	Uncharacterized protein (Fragment) OS=Cavia porcellus GN=HAT1 PE=4 SV=1	CAVPO	2
2465	2.28	2.46	18.45	11.26	4.854	tr|H0VII4|H0VII4_CAVPO	Uncharacterized protein (Fragment) OS=Cavia porcellus GN=GTF3C5 PE=4 SV=1	CAVPO	2
2466	2.28	2.32	24.18	18.95	7.19	tr|H0VUF9|H0VUF9_CAVPO	Uncharacterized protein OS=Cavia porcellus GN=CSDC2 PE=4 SV=1	CAVPO	1
2467	2.27	2.42	15.25	9.746	4.025	tr|H0VE54|H0VE54_CAVPO	Uncharacterized protein (Fragment) OS=Cavia porcellus GN=Nampt PE=4 SV=1	CAVPO	2

2468	2.27	2.3	20.53	14.74	5.263	tr|H0VI91|H0VI91_CAVPO	Uncharacterized protein OS=Cavia porcellus GN=DCTN6 PE=4 SV=1	CAVPO	1
2469	2.26	2.34	16.24	5.412	3.294	tr|H0UXR3|H0UXR3_CAVPO	Uncharacterized protein OS=Cavia porcellus GN=AADAT PE=4 SV=1	CAVPO	1
2470	2.26	2.44	19.57	13.66	7.143	tr|H0VJY0|H0VJY0_CAVPO	Uncharacterized protein (Fragment) OS=Cavia porcellus GN=RBBP5 PE=4 SV=1	CAVPO	2
2471	2.25	2.37	11.8	3.675	3.675	tr|H0UTF7|H0UTF7_CAVPO	Amidophosphoribosyltransferase OS=Cavia porcellus GN=PPAT PE=3 SV=1	CAVPO	2
2472	2.25	2.38	8.511	3.319	2.213	tr|H0UTZ8|H0UTZ8_CAVPO	Phosphoinositide phospholipase C OS=Cavia porcellus GN=PLCB4 PE=4 SV=1	CAVPO	2
2473	2.25	2.39	8.636	4.922	1.295	tr|H0VGV7|H0VGV7_CAVPO	Uncharacterized protein OS=Cavia porcellus GN=TECPR1 PE=4 SV=1	CAVPO	1
2474	2.25	2.45	30.61	15.31	6.463	tr|H0VMN7|H0VMN7_CAVPO	Uncharacterized protein OS=Cavia porcellus GN=C5orf51 PE=4 SV=1	CAVPO	2
2475	2.25	2.38	10.72	6.369	1.38	tr|H0VNK8|H0VNK8_CAVPO	Uncharacterized protein OS=Cavia porcellus GN=ERAP1 PE=4 SV=1	CAVPO	1
2476	2.25	2.38	8.407	8.407	8.407	tr|Q9TEG6|Q9TEG6_CAVPO	ATP synthase subunit a OS=Cavia porcellus GN=ATPase 6 PE=3 SV=1	CAVPO	2
2477	2.24	2.28	9.724	3.728	1.783	tr|H0V1I3|H0V1I3_CAVPO	Uncharacterized protein OS=Cavia porcellus GN=VPS33B PE=4 SV=1	CAVPO	1
2478	2.24	2.37	7.143	7.143	4.898	tr|H0VBV6|H0VBV6_CAVPO	Uncharacterized protein OS=Cavia porcellus GN=TOM1 PE=4 SV=1	CAVPO	2
2479	2.24	2.27	10.86	7.566	3.618	tr|H0VMA1|H0VMA1_CAVPO	Uncharacterized protein (Fragment) OS=Cavia porcellus GN=ACOT8 PE=4 SV=1	CAVPO	1
2480	2.24	2.26	7.035	4.774	2.764	tr|H0VN40|H0VN40_CAVPO	Uncharacterized protein OS=Cavia porcellus GN=C17orf75 PE=4 SV=1	CAVPO	1
2481	2.23	2.26	10.54	3.514	1.597	tr|H0UYA2|H0UYA2_CAVPO	Uncharacterized protein OS=Cavia porcellus GN=PEX5L PE=4 SV=1	CAVPO	1

2482	2.23	2.38	16.14	16.14	9.259	tr|H0VGF2|H0VGF2_CAVPO	Uncharacterized protein (Fragment) OS=Cavia porcellus GN=VASP PE=4 SV=1	CAVPO	2
2483	2.22	6.03	15.76	14.35	7.882	tr|H0UUM0|H0UUM0_CAVPO	Uncharacterized protein OS=Cavia porcellus GN=DLG3 PE=4 SV=1	CAVPO	5
2484	2.22	2.25	39.32	17.09	17.09	tr|H0VJW6|H0VJW6_CAVPO	Uncharacterized protein OS=Cavia porcellus GN=Rpa3 PE=4 SV=1	CAVPO	2
2485	2.22	2.26	6.639	2.075	2.075	tr|H0VLI1|H0VLI1_CAVPO	Uncharacterized protein OS=Cavia porcellus GN=INTS4 PE=4 SV=1	CAVPO	2
2486	2.22	2.36	10.34	10.34	2.842	tr|H0W205|H0W205_CAVPO	Uncharacterized protein OS=Cavia porcellus GN=MCAT PE=4 SV=1	CAVPO	1
2487	2.21	2.32	6.491	2.86	1.76	tr|H0UVD6|H0UVD6_CAVPO	Uncharacterized protein OS=Cavia porcellus GN=HACE1 PE=4 SV=1	CAVPO	1
2488	2.21	2.37	6.869	5.945	4.359	tr|H0V8P7|H0V8P7_CAVPO	Uncharacterized protein OS=Cavia porcellus GN=MFN2 PE=4 SV=1	CAVPO	3
2489	2.21	2.35	18.55	10.71	6.119	tr|H0V9I4|H0V9I4_CAVPO	Uncharacterized protein OS=Cavia porcellus GN=ACBD3 PE=4 SV=1	CAVPO	2
2490	2.21	2.24	31.63	16.84	6.122	tr|H0VJB0|H0VJB0_CAVPO	Uncharacterized protein (Fragment) OS=Cavia porcellus GN=NAA10 PE=4 SV=1	CAVPO	1
2491	2.21	2.24	22.79	11.26	7.775	tr|H0VRR7|H0VRR7_CAVPO	Uncharacterized protein (Fragment) OS=Cavia porcellus GN=Cep41 PE=4 SV=1	CAVPO	2
2492	2.2	2.22	23.32	16.58	7.772	tr|H0UVN3|H0UVN3_CAVPO	Uncharacterized protein OS=Cavia porcellus GN=CSRP1 PE=4 SV=1	CAVPO	2
2493	2.2	2.26	13.61	7.483	2.948	tr|H0V2K7|H0V2K7_CAVPO	Uncharacterized protein (Fragment) OS=Cavia porcellus GN=ACOT9 PE=4 SV=1	CAVPO	1
2494	2.2	2.26	5.707	3.442	1.812	tr|H0V9H6|H0V9H6_CAVPO	Uncharacterized protein (Fragment) OS=Cavia porcellus GN=CCDC136 PE=4 SV=1	CAVPO	1
2495	2.2	16.1	34.04	27.02	24.56	tr|H0V9Q6|H0V9Q6_CAVPO	Uncharacterized protein OS=Cavia porcellus GN=TPM3 PE=3 SV=1	CAVPO	8

2496	2.19	2.32	10.94	4.34	4.34	tr|H0VAE0|H0VAE0_CAVPO	Uncharacterized protein OS=Cavia porcellus GN=OPTN PE=4 SV=1	CAVPO	2
2497	2.19	2.23	17.83	11.09	1.957	tr|H0VP96|H0VP96_CAVPO	Uncharacterized protein OS=Cavia porcellus GN=CCDC6 PE=4 SV=1	CAVPO	1
2498	2.19	2.35	13.76	6.237	3.656	tr|H0W0L9|H0W0L9_CAVPO	Uncharacterized protein (Fragment) OS=Cavia porcellus GN=OLFM1 PE=4 SV=1	CAVPO	2
2499	2.19	2.21	17.11	17.11	15.79	tr|H0W2T4|H0W2T4_CAVPO	Uncharacterized protein OS=Cavia porcellus GN=SNRPG PE=4 SV=1	CAVPO	1
2500	2.19	2.22	17.67	5.511	2.107	tr|H0WDS3|H0WDS3_CAVPO	Uncharacterized protein OS=Cavia porcellus GN=CCDC22 PE=4 SV=1	CAVPO	1
2501	2.18	2.21	19.2	16.91	3.725	tr|H0UVF2|H0UVF2_CAVPO	Uncharacterized protein OS=Cavia porcellus GN=GKAP1 PE=4 SV=1	CAVPO	1
2502	2.18	2.2	4.583	4.583	2.708	tr|H0VJM4|H0VJM4_CAVPO	Uncharacterized protein OS=Cavia porcellus GN=DHCR7 PE=4 SV=1	CAVPO	1
2503	2.18	2.34	12.44	7.68	2.611	tr|H0VLI9|H0VLI9_CAVPO	Uncharacterized protein (Fragment) OS=Cavia porcellus GN=DPY19L1 PE=4 SV=1	CAVPO	2
2504	2.18	2.21	19.43	14.86	7.429	tr|Q9JKP6|Q9JKP6_CAVPO	Ferritin OS=Cavia porcellus PE=2 SV=1	CAVPO	1
2505	2.17	2.19	33.71	33.71	31.46	tr|H0V273|H0V273_CAVPO	Uncharacterized protein OS=Cavia porcellus GN=HMGN4 PE=4 SV=1	CAVPO	3
2506	2.17	4.32	10.73	7.309	3.266	tr|H0V3F9|H0V3F9_CAVPO	Uncharacterized protein (Fragment) OS=Cavia porcellus GN=SLC27A4 PE=4 SV=1	CAVPO	2
2507	2.17	2.2	6.025	2.895	1.095	tr|H0V792|H0V792_CAVPO	Uncharacterized protein OS=Cavia porcellus GN=GIGYF2 PE=4 SV=1	CAVPO	1
2508	2.17	2.2	15.38	9.67	2.637	tr|H0VM37|H0VM37_CAVPO	Uncharacterized protein OS=Cavia porcellus GN=HTRA2 PE=4 SV=1	CAVPO	1
2509	2.17	2.21	11.4	8.772	4.825	tr|H0VM72|H0VM72_CAVPO	Uncharacterized protein OS=Cavia porcellus GN=CD9 PE=4 SV=1	CAVPO	1

2510	2.17	2.19	12.88	5.828	3.374	tr|H0W9U4|H0W9U4_CAVPO	Uncharacterized protein OS=Cavia porcellus GN=STX16-NPEPL1 PE=3 SV=1	CAVPO	1
2511	2.16	2.29	7.527	3.465	3.465	tr|H0UTR3|H0UTR3_CAVPO	Tuftelin-interacting protein 11 OS=Cavia porcellus GN=TFIP11 PE=3 SV=1	CAVPO	2
2512	2.15	2.29	17.14	13.04	3.325	tr|H0VLY7|H0VLY7_CAVPO	Uncharacterized protein OS=Cavia porcellus GN=GALK1 PE=3 SV=1	CAVPO	1
2513	2.15	2.19	11.95	9.61	7.013	tr|H0VRF7|H0VRF7_CAVPO	Uncharacterized protein OS=Cavia porcellus GN=SMARCB1 PE=4 SV=1	CAVPO	2
2514	2.15	2.17	20.15	19.4	11.19	tr|H0W8T1|H0W8T1_CAVPO	40S ribosomal protein S24 (Fragment) OS=Cavia porcellus GN=LOC100724673 PE=3 SV=1	CAVPO	1
2515	2.14	6.2	14.7	12.99	6.838	tr|H0UYM9|H0UYM9_CAVPO	Uncharacterized protein (Fragment) OS=Cavia porcellus GN=UBQLN4 PE=4 SV=1	CAVPO	4
2516	2.14	2.67	13.51	6.892	3.243	tr|H0VAY0|H0VAY0_CAVPO	Uncharacterized protein OS=Cavia porcellus GN=RBM12B PE=4 SV=1	CAVPO	2
2517	2.14	2.17	18.8	5	2.8	tr|H0VFS9|H0VFS9_CAVPO	Uncharacterized protein (Fragment) OS=Cavia porcellus GN=PPIG PE=4 SV=1	CAVPO	1
2518	2.14	2.22	9.241	2.483	1.379	tr|H0VH82|H0VH82_CAVPO	Uncharacterized protein OS=Cavia porcellus GN=TARS2 PE=3 SV=1	CAVPO	1
2519	2.14	2.16	4.318	3.203	1.811	tr|H0VKY3|H0VKY3_CAVPO	Uncharacterized protein (Fragment) OS=Cavia porcellus GN=TRIM67 PE=4 SV=1	CAVPO	1
2520	2.14	3.18	18.09	6.202	6.202	tr|H0VNF4|H0VNF4_CAVPO	Uncharacterized protein (Fragment) OS=Cavia porcellus GN=NADK2 PE=4 SV=1	CAVPO	2
2521	2.13	2.3	9.58	7.059	7.059	tr|H0V2Y8|H0V2Y8_CAVPO	Uncharacterized protein (Fragment) OS=Cavia porcellus GN=ZNF512 PE=4 SV=1	CAVPO	3
2522	2.13	2.18	8.798	5.15	3.004	tr|H0VD78|H0VD78_CAVPO	Uncharacterized protein OS=Cavia porcellus GN=MPP1 PE=4 SV=1	CAVPO	1
2523	2.13	2.26	18.93	8.214	4.286	tr|H0VLP3|H0VLP3_CAVPO	Uncharacterized protein OS=Cavia porcellus	CAVPO	1

							GN=NMNAT1 PE=4 SV=1		
2524	2.13	2.15	22.81	22.81	11.4	tr|H0VZE8|H0VZE8_CAVPO	Uncharacterized protein (Fragment) OS=Cavia porcellus GN=LOC100728264 PE=4 SV=1	CAVPO	1
2525	2.12	2.14	7.495	5.484	2.559	tr|H0V0P4|H0V0P4_CAVPO	Uncharacterized protein OS=Cavia porcellus GN=DDX56 PE=4 SV=1	CAVPO	1
2526	2.12	2.17	15.09	15.09	5.189	tr|H0W938|H0W938_CAVPO	Uncharacterized protein (Fragment) OS=Cavia porcellus GN=MOB1A PE=4 SV=1	CAVPO	1
2527	2.11	7.36	17.23	14.04	10.67	tr|H0UTJ4|H0UTJ4_CAVPO	Uncharacterized protein (Fragment) OS=Cavia porcellus GN=PPP2R5C PE=4 SV=1	CAVPO	5
2528	2.11	2.22	15.11	7.418	5.22	tr|H0VMC7|H0VMC7_CAVPO	Uncharacterized protein OS=Cavia porcellus GN=Ist1 PE=4 SV=1	CAVPO	1
2529	2.11	2.14	29.79	6.507	4.11	tr|H0VNK1|H0VNK1_CAVPO	Uncharacterized protein (Fragment) OS=Cavia porcellus GN=CCDC50 PE=4 SV=1	CAVPO	1
2530	2.11	2.13	9.091	6.364	3.333	tr|H0VQZ3|H0VQZ3_CAVPO	Uncharacterized protein (Fragment) OS=Cavia porcellus GN=KDSR PE=3 SV=1	CAVPO	1
2531	2.11	2.95	6.667	3.74	3.74	tr|H0VT91|H0VT91_CAVPO	Uncharacterized protein OS=Cavia porcellus GN=LRIT1 PE=4 SV=1	CAVPO	2
2532	2.11	2.25	28.64	25.13	18.09	tr|H0VUJ9|H0VUJ9_CAVPO	Uncharacterized protein (Fragment) OS=Cavia porcellus GN=MEMO1 PE=4 SV=1	CAVPO	2
2533	2.11	2.14	3.285	1.951	1.129	tr|H0W0U9|H0W0U9_CAVPO	Uncharacterized protein OS=Cavia porcellus GN=VPS18 PE=4 SV=1	CAVPO	1
2534	2.11	2.15	10.96	3.373	1.686	tr|H0W1A0|H0W1A0_CAVPO	Uncharacterized protein OS=Cavia porcellus GN=STXBP2 PE=4 SV=1	CAVPO	1
2535	2.1	3.52	8.333	6.481	3.009	tr|H0V8B3|H0V8B3_CAVPO	Uncharacterized protein OS=Cavia porcellus GN=KCNV2 PE=4 SV=1	CAVPO	2
2536	2.09	2.11	10.37	4.259	2.963	tr|H0V1T9|H0V1T9_CAVPO	Uncharacterized protein OS=Cavia porcellus GN=PPM1G PE=3 SV=1	CAVPO	1

2537	2.09	4.38	13.91	6.653	4.435	tr|H0V5K0|H0V5K0_CAVPO	Uncharacterized protein OS=Cavia porcellus GN=CDK16 PE=4 SV=1	CAVPO	3
2538	2.09	2.3	25.71	25.71	12.86	tr|H0V9Z8|H0V9Z8_CAVPO	Uncharacterized protein OS=Cavia porcellus GN=LAMP5 PE=4 SV=1	CAVPO	3
2539	2.09	2.11	4.49	2.309	0.898	tr|H0VBS9|H0VBS9_CAVPO	Uncharacterized protein (Fragment) OS=Cavia porcellus GN=LAMC1 PE=4 SV=1	CAVPO	1
2540	2.09	2.11	3.201	3.201	1.677	tr|H0VT86|H0VT86_CAVPO	Uncharacterized protein OS=Cavia porcellus GN=SMPD3 PE=4 SV=1	CAVPO	1
2541	2.09	2.24	19.03	7.386	7.386	tr|H0VWG7|H0VWG7_CAVPO	Uncharacterized protein OS=Cavia porcellus GN=VPS37C PE=4 SV=1	CAVPO	2
2542	2.09	2.22	9.259	4.444	4.444	tr|H0WCA0|H0WCA0_CAVPO	Uncharacterized protein (Fragment) OS=Cavia porcellus GN=PPAP2B PE=4 SV=1	CAVPO	1
2543	2.08	2.22	9.163	3.791	3.791	tr|H0UY53|H0UY53_CAVPO	Uncharacterized protein (Fragment) OS=Cavia porcellus GN=PARN PE=4 SV=1	CAVPO	2
2544	2.08	2.09	4.737	3.509	2.105	tr|H0V439|H0V439_CAVPO	Uncharacterized protein OS=Cavia porcellus GN=MSTO1 PE=4 SV=1	CAVPO	1
2545	2.08	2.09	23.94	23.94	23.94	tr|H0VRF4|H0VRF4_CAVPO	Uncharacterized protein OS=Cavia porcellus GN=CHCHD10 PE=4 SV=1	CAVPO	2
2546	2.08	2.1	12.92	3.197	1.535	tr|H0VSW8|H0VSW8_CAVPO	Uncharacterized protein OS=Cavia porcellus GN=KIAA1468 PE=4 SV=1	CAVPO	1
2547	2.08	2.1	7.941	6.176	3.235	tr|H0VSX8|H0VSX8_CAVPO	Uncharacterized protein (Fragment) OS=Cavia porcellus GN=CD2BP2 PE=4 SV=1	CAVPO	1
2548	2.08	2.09	6.342	3.687	1.917	tr|H0W5D6|H0W5D6_CAVPO	Uncharacterized protein (Fragment) OS=Cavia porcellus GN=RSBN1L PE=4 SV=1	CAVPO	1
2549	2.08	2.09	6.667	5.067	2.933	tr|H0WAU1|H0WAU1_CAVPO	Uncharacterized protein OS=Cavia porcellus GN=MEIS3 PE=3 SV=1	CAVPO	1
2550	2.07	2.97	22.27	13.64	2.955	tr|H0V248|H0V248_CAVPO	Glycylpeptide N-tetradecanoyltransferase (Fragment) OS=Cavia porcellus GN=NMT2	CAVPO	1

							PE=3 SV=1		
2551	2.07	2.08	40	20.67	9.333	tr|H0V922|H0V922_CAVPO	Uncharacterized protein OS=Cavia porcellus GN=C5orf24 PE=4 SV=1	CAVPO	1
2552	2.07	2.09	18.04	5.836	3.183	tr|H0VJR0|H0VJR0_CAVPO	Uncharacterized protein OS=Cavia porcellus GN=HM13 PE=4 SV=1	CAVPO	1
2553	2.07	2.09	21.56	11.93	5.046	tr|H0VK89|H0VK89_CAVPO	Eukaryotic translation initiation factor 3 subunit K OS=Cavia porcellus GN=EIF3K PE=3 SV=1	CAVPO	1
2554	2.06	2.17	4.097	4.097	2.048	tr|H0UVT8|H0UVT8_CAVPO	Uncharacterized protein (Fragment) OS=Cavia porcellus GN=MLLT1 PE=4 SV=1	CAVPO	1
2555	2.06	2.1	7.98	4.072	2.28	tr|H0UW96|H0UW96_CAVPO	Uncharacterized protein (Fragment) OS=Cavia porcellus GN=MEN1 PE=4 SV=1	CAVPO	1
2556	2.06	3.3	12.5	12.5	6	tr|H0UYN8|H0UYN8_CAVPO	Uncharacterized protein OS=Cavia porcellus GN=Habp4 PE=4 SV=1	CAVPO	2
2557	2.06	2.27	16.14	10.76	4.933	tr|H0VGT2|H0VGT2_CAVPO	Uncharacterized protein OS=Cavia porcellus GN=EPDR1 PE=4 SV=1	CAVPO	1
2558	2.06	29.5	59.78	42.98	41.6	tr|H0VIV8|H0VIV8_CAVPO	Uncharacterized protein (Fragment) OS=Cavia porcellus GN=HNRNPA3 PE=4 SV=1	CAVPO	29
2559	2.06	2.07	4.944	2.097	1.423	tr|H0VMQ3|H0VMQ3_CAVPO	Uncharacterized protein OS=Cavia porcellus GN=RPTOR PE=4 SV=1	CAVPO	1
2560	2.06	2.44	5.364	1.756	0.6502	tr|H0VND7|H0VND7_CAVPO	Uncharacterized protein (Fragment) OS=Cavia porcellus GN=LAMA1 PE=4 SV=1	CAVPO	2
2561	2.06	2.15	25.85	21.61	10.59	tr|H0WD78|H0WD78_CAVPO	Uncharacterized protein OS=Cavia porcellus PE=4 SV=1	CAVPO	1
2562	2.05	2.06	6.204	1.846	0.8863	tr|H0UV20|H0UV20_CAVPO	DNA mismatch repair protein MSH6 OS=Cavia porcellus GN=MSH6 PE=3 SV=1	CAVPO	1
2563	2.05	3.66	12.98	4.606	2.68	tr|H0V152|H0V152_CAVPO	Uncharacterized protein OS=Cavia porcellus GN=PPFIA3 PE=4 SV=1	CAVPO	3

2564	2.05	2.05	5.893	2.143	2.143	tr|H0V154|H0V154_CAVPO	Uncharacterized protein OS=Cavia porcellus GN=TDRKH PE=4 SV=1	CAVPO	1
2565	2.05	2.13	12.66	6.846	3.112	tr|H0VP19|H0VP19_CAVPO	Uncharacterized protein (Fragment) OS=Cavia porcellus GN=STK39 PE=4 SV=1	CAVPO	1
2566	2.05	2.06	32.63	23.16	12.63	tr|H0WCS2|H0WCS2_CAVPO	Uncharacterized protein OS=Cavia porcellus GN=Sumo2 PE=4 SV=1	CAVPO	1
2567	2.04	3.32	6.076	1.83	1.245	tr|H0UW50|H0UW50_CAVPO	Uncharacterized protein OS=Cavia porcellus GN=BRD4 PE=4 SV=1	CAVPO	2
2568	2.04	2.21	19.52	8.571	8.571	tr|H0UWG3|H0UWG3_CAVPO	Uncharacterized protein OS=Cavia porcellus GN=POLR2E PE=3 SV=1	CAVPO	2
2569	2.04	2.05	8.685	3.474	3.474	tr|H0UXR7|H0UXR7_CAVPO	Uncharacterized protein OS=Cavia porcellus GN=INPP1 PE=4 SV=1	CAVPO	1
2570	2.04	2.05	4.183	2.471	2.471	tr|H0V4R0|H0V4R0_CAVPO	Uncharacterized protein (Fragment) OS=Cavia porcellus GN=PNKP PE=4 SV=1	CAVPO	1
2571	2.04	2.52	8.045	1.727	0.5055	tr|H0VB25|H0VB25_CAVPO	Uncharacterized protein (Fragment) OS=Cavia porcellus GN=ATRX PE=4 SV=1	CAVPO	1
2572	2.04	6.06	18.92	17.84	17.84	tr|H0VCA7|H0VCA7_CAVPO	Uncharacterized protein (Fragment) OS=Cavia porcellus GN=CBX1 PE=4 SV=1	CAVPO	3
2573	2.04	2.04	17.34	11.56	11.56	tr|H0VFU8|H0VFU8_CAVPO	Uncharacterized protein OS=Cavia porcellus GN=GCSH PE=3 SV=1	CAVPO	1
2574	2.04	2.05	10.89	5.611	5.611	tr|H0VHN1|H0VHN1_CAVPO	Elongation factor Ts (Fragment) OS=Cavia porcellus GN=TSFM PE=3 SV=1	CAVPO	1
2575	2.04	2.05	9.649	2.193	2.193	tr|H0VKT4|H0VKT4_CAVPO	Uncharacterized protein (Fragment) OS=Cavia porcellus GN=EPS8 PE=4 SV=1	CAVPO	1
2576	2.04	2.04	6.992	4.025	2.542	tr|H0VLY6|H0VLY6_CAVPO	Uncharacterized protein OS=Cavia porcellus GN=MBOAT7 PE=4 SV=1	CAVPO	1
2577	2.04	2.05	10.41	2.048	2.048	tr|H0VQH8|H0VQH8_CAVPO	Uncharacterized protein (Fragment) OS=Cavia porcellus GN=PLBD2 PE=4 SV=1	CAVPO	1

2578	2.04	2.05	13.44	4.743	4.743	tr|H0VSC4|H0VSC4_CAVPO	Uncharacterized protein OS=Cavia porcellus GN=FGFR1OP2 PE=4 SV=1	CAVPO	1
2579	2.04	2.05	3.463	3.463	1.385	tr|H0VU91|H0VU91_CAVPO	Uncharacterized protein (Fragment) OS=Cavia porcellus GN=INPP4B PE=4 SV=1	CAVPO	1
2580	2.04	2.2	12.33	9.692	5.286	tr|H0VWH5|H0VWH5_CAVPO	Uncharacterized protein (Fragment) OS=Cavia porcellus GN=ANKRD33B PE=4 SV=1	CAVPO	1
2581	2.04	2.07	5.649	0.9932	0.6828	tr|H0W7Q9|H0W7Q9_CAVPO	Cytosine-specific methyltransferase OS=Cavia porcellus GN=DNMT1 PE=3 SV=1	CAVPO	1
2582	2.03	2.03	20.71	8.571	8.571	tr|H0UUV9|H0UUV9_CAVPO	Uncharacterized protein OS=Cavia porcellus GN=CCDC126 PE=4 SV=1	CAVPO	1
2583	2.03	2.04	7.27	1.646	1.646	tr|H0VHU6|H0VHU6_CAVPO	Uncharacterized protein OS=Cavia porcellus GN=TBK1 PE=4 SV=1	CAVPO	1
2584	2.03	2.03	34.33	26.12	26.12	tr|H0VLJ0|H0VLJ0_CAVPO	Uncharacterized protein (Fragment) OS=Cavia porcellus GN=PFDN4 PE=4 SV=1	CAVPO	2
2585	2.03	2.05	7.537	1.884	1.884	tr|H0VLL6|H0VLL6_CAVPO	Dystrobrevin OS=Cavia porcellus GN=DTNA PE=3 SV=1	CAVPO	1
2586	2.03	2.18	13.77	8.945	6.798	tr|H0VM09|H0VM09_CAVPO	Uncharacterized protein (Fragment) OS=Cavia porcellus GN=SGPL1 PE=3 SV=1	CAVPO	2
2587	2.03	2.21	13.47	8.632	5.684	tr|H0VSK3|H0VSK3_CAVPO	Uncharacterized protein OS=Cavia porcellus GN=SPTLC1 PE=4 SV=1	CAVPO	2
2588	2.03	2.03	13.79	8.966	8.966	tr|H0VSU4|H0VSU4_CAVPO	Uncharacterized protein OS=Cavia porcellus GN=TOMM20 PE=4 SV=1	CAVPO	2
2589	2.03	2.09	6.531	5.102	3.061	tr|H0VVP8|H0VVP8_CAVPO	Uncharacterized protein (Fragment) OS=Cavia porcellus GN=KCNA3 PE=4 SV=1	CAVPO	1
2590	2.03	2.04	9.477	3.268	3.268	tr|H0W265|H0W265_CAVPO	Uncharacterized protein (Fragment) OS=Cavia porcellus GN=CTSZ PE=3 SV=1	CAVPO	1
2591	2.03	2.04	17.19	4.688	4.688	tr|H0W4R5|H0W4R5_CAVPO	Uncharacterized protein (Fragment) OS=Cavia porcellus GN=CA8 PE=4 SV=1	CAVPO	1

2592	2.03	2.13	7.324	5.634	2.817	tr|H0WCR1|H0WCR1_CAVPO	Uncharacterized protein OS=Cavia porcellus GN=IMPAD1 PE=4 SV=1	CAVPO	1
2593	2.03	2.03	10.62	6.227	6.227	tr|O35477|O35477_CAVPO	Green-sensitive opsin (Fragment) OS=Cavia porcellus PE=2 SV=1	CAVPO	2
2594	2.02	2.52	7.759	5.345	2.759	tr|H0UZS1|H0UZS1_CAVPO	Uncharacterized protein (Fragment) OS=Cavia porcellus GN=YTHDF3 PE=4 SV=1	CAVPO	1
2595	2.02	2.03	23.61	5.093	5.093	tr|H0V4R1|H0V4R1_CAVPO	Uncharacterized protein OS=Cavia porcellus GN=CNPY4 PE=4 SV=1	CAVPO	1
2596	2.02	2.02	16.74	8.372	8.372	tr|H0V5Q2|H0V5Q2_CAVPO	Uncharacterized protein (Fragment) OS=Cavia porcellus GN=SCN2B PE=4 SV=1	CAVPO	1
2597	2.02	2.02	10.74	3.306	3.306	tr|H0V829|H0V829_CAVPO	Uncharacterized protein OS=Cavia porcellus GN=AGPAT5 PE=4 SV=1	CAVPO	1
2598	2.02	2.02	8.251	4.29	4.29	tr|H0V8E1|H0V8E1_CAVPO	Uncharacterized protein (Fragment) OS=Cavia porcellus GN=YIF1B PE=4 SV=1	CAVPO	1
2599	2.02	2.03	8.841	2.161	2.161	tr|H0VA23|H0VA23_CAVPO	Uncharacterized protein (Fragment) OS=Cavia porcellus GN=ARSA PE=4 SV=1	CAVPO	1
2600	2.02	2.04	18.64	7.91	7.91	tr|H0VD27|H0VD27_CAVPO	Uncharacterized protein OS=Cavia porcellus GN=CUTA PE=4 SV=1	CAVPO	1
2601	2.02	2.03	24.55	12.73	12.73	tr|H0VDL5|H0VDL5_CAVPO	Uncharacterized protein OS=Cavia porcellus GN=SRP14 PE=4 SV=1	CAVPO	1
2602	2.02	2.03	6.144	2.331	2.331	tr|H0VK74|H0VK74_CAVPO	Uncharacterized protein OS=Cavia porcellus GN=PRCC PE=4 SV=1	CAVPO	1
2603	2.02	2.35	11.07	6.086	3.596	tr|H0VL16|H0VL16_CAVPO	Uncharacterized protein (Fragment) OS=Cavia porcellus GN=APLP2 PE=4 SV=1	CAVPO	3
2604	2.02	2.02	15.53	7.767	7.767	tr|H0VQ30|H0VQ30_CAVPO	Uncharacterized protein (Fragment) OS=Cavia porcellus GN=LSM7 PE=4 SV=1	CAVPO	1
2605	2.02	2.03	9.375	2.539	2.539	tr|H0VQ36|H0VQ36_CAVPO	Uncharacterized protein (Fragment) OS=Cavia porcellus GN=PODXL PE=4 SV=1	CAVPO	1

2606	2.02	2.03	8.088	8.088	8.088	tr|H0VRN6|H0VRN6_CAVPO	Uncharacterized protein (Fragment) OS=Cavia porcellus GN=Fau PE=4 SV=1	CAVPO	2
2607	2.02	2.02	22.97	7.432	7.432	tr|H0VZC2|H0VZC2_CAVPO	Uncharacterized protein OS=Cavia porcellus GN=Rpl27a PE=3 SV=1	CAVPO	1
2608	2.02	2.15	10.63	10.63	10.63	tr|H0W6U9|H0W6U9_CAVPO	Uncharacterized protein OS=Cavia porcellus GN=AP1S2 PE=4 SV=1	CAVPO	2
2609	2.02	2.02	5.191	1.679	1.679	tr|H0W7E3|H0W7E3_CAVPO	Uncharacterized protein (Fragment) OS=Cavia porcellus GN=NUP85 PE=4 SV=1	CAVPO	1
2610	2.02	2.03	6.278	3.587	3.587	tr|H0W7S7|H0W7S7_CAVPO	Uncharacterized protein OS=Cavia porcellus GN=Sigmar1 PE=4 SV=1	CAVPO	1
2611	2.01	2.01	7.325	1.704	1.704	tr|H0UVL2|H0UVL2_CAVPO	Uncharacterized protein (Fragment) OS=Cavia porcellus GN=FNBP1 PE=4 SV=1	CAVPO	1
2612	2.01	6.59	12.05	9.178	9.178	tr|H0UXP0|H0UXP0_CAVPO	Uncharacterized protein OS=Cavia porcellus GN=FAM98A PE=4 SV=1	CAVPO	4
2613	2.01	2.01	25	12.88	12.88	tr|H0UXX5|H0UXX5_CAVPO	Uncharacterized protein (Fragment) OS=Cavia porcellus GN=HYPK PE=4 SV=1	CAVPO	1
2614	2.01	2.12	6.096	4.064	2.322	tr|H0UZQ4|H0UZQ4_CAVPO	Uncharacterized protein (Fragment) OS=Cavia porcellus GN=Sp3 PE=4 SV=1	CAVPO	1
2615	2.01	2.02	4.645	3.005	3.005	tr|H0V0Q3|H0V0Q3_CAVPO	Uncharacterized protein (Fragment) OS=Cavia porcellus GN=GTF2A1 PE=4 SV=1	CAVPO	1
2616	2.01	2.01	5.371	2.054	2.054	tr|H0V0S0|H0V0S0_CAVPO	Uncharacterized protein OS=Cavia porcellus GN=CLPX PE=4 SV=1	CAVPO	1
2617	2.01	2.02	9.034	4.673	4.673	tr|H0V9E1|H0V9E1_CAVPO	Annexin (Fragment) OS=Cavia porcellus GN=ANXA4 PE=3 SV=1	CAVPO	1
2618	2.01	2.01	14.94	5.364	5.364	tr|H0V9I0|H0V9I0_CAVPO	Uncharacterized protein (Fragment) OS=Cavia porcellus GN=MRPL28 PE=4 SV=1	CAVPO	1
2619	2.01	2.02	15.91	9.091	9.091	tr|H0V9I8|H0V9I8_CAVPO	Uncharacterized protein OS=Cavia porcellus GN=FABP4 PE=3 SV=1	CAVPO	2

2620	2.01	2.02	3.834	2.738	1.205	tr|H0VB33|H0VB33_CAVPO	Uncharacterized protein (Fragment) OS=Cavia porcellus GN=RASA1 PE=4 SV=1	CAVPO	1
2621	2.01	2.01	5.714	3.117	3.117	tr|H0VB97|H0VB97_CAVPO	Uncharacterized protein OS=Cavia porcellus GN=LIMS1 PE=4 SV=1	CAVPO	1
2622	2.01	2.02	6.267	2.4	2.4	tr|H0VCS6|H0VCS6_CAVPO	Uncharacterized protein (Fragment) OS=Cavia porcellus GN=TCEB3 PE=4 SV=1	CAVPO	1
2623	2.01	2.02	11.01	7.048	7.048	tr|H0VEN2|H0VEN2_CAVPO	Uncharacterized protein (Fragment) OS=Cavia porcellus GN=NCCRP1 PE=4 SV=1	CAVPO	1
2624	2.01	2.02	14.39	6.439	6.439	tr|H0VF48|H0VF48_CAVPO	Uncharacterized protein OS=Cavia porcellus GN=C12orf5 PE=4 SV=1	CAVPO	1
2625	2.01	2.01	7.65	6.557	6.557	tr|H0W5K7|H0W5K7_CAVPO	Uncharacterized protein OS=Cavia porcellus GN=JAGN1 PE=4 SV=1	CAVPO	1
2626	2.01	2.02	9.117	3.989	3.989	tr|H0W753|H0W753_CAVPO	Uncharacterized protein OS=Cavia porcellus GN=NECAB1 PE=4 SV=1	CAVPO	1
2627	2.01	2.14	16.56	5.664	3.486	tr|H0WAP8|H0WAP8_CAVPO	Uncharacterized protein OS=Cavia porcellus GN=SON PE=4 SV=1	CAVPO	1
2628	2.01	2.18	22.63	12.11	6.842	tr|H0WB83|H0WB83_CAVPO	Uncharacterized protein (Fragment) OS=Cavia porcellus GN=Dnal1 PE=4 SV=1	CAVPO	1
2629	2.01	2.01	13.14	13.14	13.14	tr|H0WD05|H0WD05_CAVPO	Uncharacterized protein OS=Cavia porcellus GN=AGPAT1 PE=4 SV=1	CAVPO	2
2630	2.01	2.01	5.013	2.375	2.375	tr|Q9TEF9|Q9TEF9_CAVPO	Cytochrome b OS=Cavia porcellus GN=cytb PE=3 SV=1	CAVPO	1
2631	2	2	5.43	5.43	5.43	tr|H0UTS1|H0UTS1_CAVPO	Uncharacterized protein (Fragment) OS=Cavia porcellus GN=SYNGR1 PE=4 SV=1	CAVPO	1
2632	2	2	2.033	2.033	2.033	tr|H0UU74|H0UU74_CAVPO	Uncharacterized protein OS=Cavia porcellus GN=PRAME PE=4 SV=1	CAVPO	1
2633	2	2	13.13	8.586	8.586	tr|H0UVV0|H0UVV0_CAVPO	Inosine triphosphate pyrophosphatase OS=Cavia porcellus GN=ITPA PE=3 SV=1	CAVPO	1

2634	2	2	25.88	14.12	14.12	tr|H0UW09|H0UW09_CAVPO	Uncharacterized protein OS=Cavia porcellus GN=MANBAL PE=4 SV=1	CAVPO	1
2635	2	2	2.23	2.23	2.23	tr|H0UWB9|H0UWB9_CAVPO	Uncharacterized protein (Fragment) OS=Cavia porcellus GN=SLC2A12 PE=3 SV=1	CAVPO	1
2636	2	2	3.036	1.469	1.469	tr|H0UX28|H0UX28_CAVPO	Uncharacterized protein OS=Cavia porcellus GN=MPRIP PE=4 SV=1	CAVPO	1
2637	2	2	2.817	1.921	1.921	tr|H0UXN0|H0UXN0_CAVPO	Uncharacterized protein OS=Cavia porcellus GN=E4F1 PE=4 SV=1	CAVPO	1
2638	2	2	5.342	3.205	3.205	tr|H0UY76|H0UY76_CAVPO	Uncharacterized protein (Fragment) OS=Cavia porcellus GN=FAM63B PE=4 SV=1	CAVPO	1
2639	2	2	3.896	3.896	3.896	tr|H0UY88|H0UY88_CAVPO	Uncharacterized protein (Fragment) OS=Cavia porcellus GN=TMED1 PE=3 SV=1	CAVPO	1
2640	2	2	9.917	9.917	9.917	tr|H0UZI9|H0UZI9_CAVPO	Protein yippee-like (Fragment) OS=Cavia porcellus GN=YPEL5 PE=3 SV=1	CAVPO	1
2641	2	2	3.249	3.249	3.249	tr|H0V0S2|H0V0S2_CAVPO	Uncharacterized protein OS=Cavia porcellus GN=TRIM14 PE=4 SV=1	CAVPO	1
2642	2	4.21	10	10	7.2	tr|H0V0U1|H0V0U1_CAVPO	Uncharacterized protein OS=Cavia porcellus GN=OTX2 PE=3 SV=1	CAVPO	2
2643	2	2	12.42	12.42	12.42	tr|H0V0U2|H0V0U2_CAVPO	Uncharacterized protein OS=Cavia porcellus GN=NHP2 PE=4 SV=1	CAVPO	1
2644	2	2	12	12	12	tr|H0V151|H0V151_CAVPO	Uncharacterized protein OS=Cavia porcellus GN=RBM3 PE=4 SV=1	CAVPO	1
2645	2	2.01	11.42	4.152	4.152	tr|H0V1C1|H0V1C1_CAVPO	Uncharacterized protein OS=Cavia porcellus GN=ATPAF2 PE=4 SV=1	CAVPO	1
2646	2	2	9.677	9.677	9.677	tr|H0V298|H0V298_CAVPO	Uncharacterized protein OS=Cavia porcellus GN=TMEM254 PE=4 SV=1	CAVPO	1
2647	2	2	11.22	11.22	11.22	tr|H0V2B0|H0V2B0_CAVPO	Uncharacterized protein OS=Cavia porcellus GN=RNASEK PE=4 SV=1	CAVPO	2

2648	2	2	4.154	4.154	4.154	tr|H0V2M9|H0V2M9_CAVPO	Uncharacterized protein OS=Cavia porcellus GN=RRH PE=3 SV=1	CAVPO	1
2649	2	2.02	9.95	6.468	6.468	tr|H0V2U0|H0V2U0_CAVPO	Uncharacterized protein OS=Cavia porcellus GN=FXN PE=4 SV=1	CAVPO	1
2650	2	4.06	10.8	5.634	5.634	tr|H0V4S4|H0V4S4_CAVPO	Uncharacterized protein OS=Cavia porcellus GN=ACTL6B PE=3 SV=1	CAVPO	2
2651	2	2	25.86	25.86	25.86	tr|H0V555|H0V555_CAVPO	Uncharacterized protein OS=Cavia porcellus GN=USMG5 PE=4 SV=1	CAVPO	1
2652	2	2.01	2.585	1.497	1.497	tr|H0V624|H0V624_CAVPO	Uncharacterized protein OS=Cavia porcellus GN=PNISR PE=4 SV=1	CAVPO	1
2653	2	10.5	53.8	46.2	46.2	tr|H0V6D2|H0V6D2_CAVPO	Uncharacterized protein OS=Cavia porcellus GN=Rps27a PE=4 SV=1	CAVPO	14
2654	2	2	3.869	2.227	1.641	tr|H0V6N6|H0V6N6_CAVPO	Uncharacterized protein OS=Cavia porcellus GN=TMEM245 PE=4 SV=1	CAVPO	1
2655	2	2	23.6	15.73	15.73	tr|H0V6V5|H0V6V5_CAVPO	Uncharacterized protein OS=Cavia porcellus GN=COX19 PE=4 SV=1	CAVPO	1
2656	2	2	9.655	9.655	9.655	tr|H0V6W0|H0V6W0_CAVPO	Uncharacterized protein (Fragment) OS=Cavia porcellus GN=C7orf50 PE=4 SV=1	CAVPO	1
2657	2	2	7.975	7.975	7.975	tr|H0V7H8|H0V7H8_CAVPO	Uncharacterized protein OS=Cavia porcellus GN=Vkorc1 PE=4 SV=1	CAVPO	1
2658	2	5.77	57.3	49.44	49.44	tr|H0V8N4|H0V8N4_CAVPO	Uncharacterized protein OS=Cavia porcellus GN=DYNLL1 PE=4 SV=1	CAVPO	3
2659	2	2	10.75	10.75	10.75	tr|H0V9A1|H0V9A1_CAVPO	Uncharacterized protein OS=Cavia porcellus GN=SH3BGRL3 PE=4 SV=1	CAVPO	1
2660	2	2	14.39	14.39	14.39	tr|H0V9T3|H0V9T3_CAVPO	Uncharacterized protein OS=Cavia porcellus GN=CNIH4 PE=4 SV=1	CAVPO	1
2661	2	3.14	33.33	33.33	33.33	tr|H0VCF8|H0VCF8_CAVPO	Uncharacterized protein (Fragment) OS=Cavia porcellus GN=VAMP1 PE=4 SV=1	CAVPO	3

2662	2	2	7.853	7.853	7.853	tr|H0VCP2|H0VCP2_CAVPO	Uncharacterized protein OS=Cavia porcellus GN=NTPCR PE=4 SV=1	CAVPO	1
2663	2	2.01	7.527	5.018	5.018	tr|H0VD08|H0VD08_CAVPO	Uncharacterized protein (Fragment) OS=Cavia porcellus GN=RFC3 PE=4 SV=1	CAVPO	1
2664	2	2.01	4.723	1.629	1.629	tr|H0VDT1|H0VDT1_CAVPO	Uncharacterized protein OS=Cavia porcellus GN=CRTC1 PE=4 SV=1	CAVPO	1
2665	2	2.03	8.032	4.819	4.819	tr|H0VDY4|H0VDY4_CAVPO	Uncharacterized protein (Fragment) OS=Cavia porcellus GN=Cd63 PE=4 SV=1	CAVPO	1
2666	2	2	2.376	2.376	2.376	tr|H0VEI1|H0VEI1_CAVPO	Uncharacterized protein OS=Cavia porcellus GN=NRF1 PE=4 SV=1	CAVPO	1
2667	2	2	6.011	6.011	6.011	tr|H0VER5|H0VER5_CAVPO	Uncharacterized protein OS=Cavia porcellus GN=GNPNAT1 PE=4 SV=1	CAVPO	1
2668	2	2	2.506	2.506	2.506	tr|H0VF14|H0VF14_CAVPO	Uncharacterized protein (Fragment) OS=Cavia porcellus GN=ENTPD5 PE=3 SV=1	CAVPO	1
2669	2	2.03	6.422	2.752	2.752	tr|H0VFJ7|H0VFJ7_CAVPO	Uncharacterized protein OS=Cavia porcellus GN=SPATS2 PE=4 SV=1	CAVPO	2
2670	2	2.04	2.652	1.641	1.641	tr|H0VG98|H0VG98_CAVPO	Uncharacterized protein (Fragment) OS=Cavia porcellus GN=KIFAP3 PE=4 SV=1	CAVPO	1
2671	2	2	3.903	2.23	2.23	tr|H0VHY3|H0VHY3_CAVPO	Uncharacterized protein OS=Cavia porcellus GN=CWF19L1 PE=4 SV=1	CAVPO	1
2672	2	2	12.07	5.46	5.46	tr|H0VII8|H0VII8_CAVPO	Uncharacterized protein OS=Cavia porcellus GN=NFYA PE=4 SV=1	CAVPO	1
2673	2	2	3.019	3.019	3.019	tr|H0VKC3|H0VKC3_CAVPO	Palmitoyltransferase OS=Cavia porcellus GN=ZDHHC21 PE=3 SV=1	CAVPO	1
2674	2	2	4.79	4.79	4.79	tr|H0VM12|H0VM12_CAVPO	Uncharacterized protein (Fragment) OS=Cavia porcellus GN=SDHC PE=4 SV=1	CAVPO	1
2675	2	2	4.333	4.333	4.333	tr|H0VP35|H0VP35_CAVPO	Uncharacterized protein (Fragment) OS=Cavia porcellus GN=OTUD6B PE=4 SV=1	CAVPO	1

2676	2	2	14.06	14.06	14.06	tr|H0VPC5|H0VPC5_CAVPO	Uncharacterized protein OS=Cavia porcellus GN=NDUFB6 PE=4 SV=1	CAVPO	1
2677	2	2.01	4.971	2.924	2.924	tr|H0VPE0|H0VPE0_CAVPO	Uncharacterized protein OS=Cavia porcellus GN=TUSC3 PE=4 SV=1	CAVPO	1
2678	2	2	4.255	4.255	4.255	tr|H0VPE5|H0VPE5_CAVPO	Uncharacterized protein OS=Cavia porcellus GN=TMCO1 PE=4 SV=1	CAVPO	1
2679	2	2	16.05	16.05	16.05	tr|H0VQC3|H0VQC3_CAVPO	Uncharacterized protein OS=Cavia porcellus GN=COX7A1 PE=4 SV=1	CAVPO	1
2680	2	2	14.47	7.895	7.895	tr|H0VQQ5|H0VQQ5_CAVPO	Uncharacterized protein OS=Cavia porcellus GN=OARD1 PE=4 SV=1	CAVPO	1
2681	2	13	48.19	47.15	47.15	tr|H0VRA2|H0VRA2_CAVPO	Uncharacterized protein OS=Cavia porcellus GN=RHOC PE=3 SV=1	CAVPO	10
2682	2	2	3.141	3.141	3.141	tr|H0VRL2|H0VRL2_CAVPO	Uncharacterized protein OS=Cavia porcellus GN=SH3BP5L PE=4 SV=1	CAVPO	1
2683	2	2	5.495	5.495	5.495	tr|H0VT54|H0VT54_CAVPO	Uncharacterized protein (Fragment) OS=Cavia porcellus GN=ZBTB8OS PE=4 SV=1	CAVPO	1
2684	2	2.01	10.03	3.762	3.762	tr|H0VTA9|H0VTA9_CAVPO	Uncharacterized protein (Fragment) OS=Cavia porcellus GN=C6orf136 PE=4 SV=1	CAVPO	1
2685	2	2	4.598	4.598	4.598	tr|H0VTS8|H0VTS8_CAVPO	Uncharacterized protein OS=Cavia porcellus GN=ENOPH1 PE=3 SV=1	CAVPO	1
2686	2	2	22.37	13.16	13.16	tr|H0VW34|H0VW34_CAVPO	Uncharacterized protein OS=Cavia porcellus GN=NDUFC1 PE=4 SV=1	CAVPO	2
2687	2	2	7.965	7.965	7.965	tr|H0VX65|H0VX65_CAVPO	Uncharacterized protein OS=Cavia porcellus GN=RNF7 PE=4 SV=1	CAVPO	1
2688	2	2	12.04	12.04	12.04	tr|H0VXJ0|H0VXJ0_CAVPO	Peptidyl-prolyl cis-trans isomerase OS=Cavia porcellus GN=FKBP1B PE=4 SV=1	CAVPO	1
2689	2	2	23.91	23.91	23.91	tr|H0VZA3|H0VZA3_CAVPO	Uncharacterized protein OS=Cavia porcellus GN=DPM3 PE=4 SV=1	CAVPO	3

2690	2	2	5.699	5.699	5.699	tr|H0VZC7|H0VZC7_CAVPO	Uncharacterized protein (Fragment) OS=Cavia porcellus GN=WIBG PE=4 SV=1	CAVPO	1
2691	2	2	2.882	2.882	2.882	tr|H0VZP8|H0VZP8_CAVPO	Protein tweety homolog OS=Cavia porcellus GN=TTYH1 PE=3 SV=1	CAVPO	1
2692	2	2	4.286	4.286	4.286	tr|H0W0I5|H0W0I5_CAVPO	Uncharacterized protein (Fragment) OS=Cavia porcellus GN=EVL PE=4 SV=1	CAVPO	1
2693	2	2	2.57	2.57	2.57	tr|H0W1C9|H0W1C9_CAVPO	Uncharacterized protein OS=Cavia porcellus GN=DMAP1 PE=4 SV=1	CAVPO	1
2694	2	2.02	21.92	12.33	12.33	tr|H0W1K9|H0W1K9_CAVPO	Uncharacterized protein (Fragment) OS=Cavia porcellus GN=NDUFS7 PE=4 SV=1	CAVPO	1
2695	2	2	8.824	4.202	4.202	tr|H0W1T3|H0W1T3_CAVPO	Uncharacterized protein OS=Cavia porcellus GN=Bcl2 PE=4 SV=1	CAVPO	1
2696	2	2	19.57	19.57	19.57	tr|H0W255|H0W255_CAVPO	Uncharacterized protein OS=Cavia porcellus GN=RPL37A PE=3 SV=1	CAVPO	2
2697	2	4.11	22.4	22.4	22.4	tr|H0W268|H0W268_CAVPO	Uncharacterized protein OS=Cavia porcellus GN=Map1lc3b PE=3 SV=1	CAVPO	3
2698	2	2.1	18.97	10.92	4.023	tr|H0W2Q7|H0W2Q7_CAVPO	Uncharacterized protein OS=Cavia porcellus GN=TPRKB PE=3 SV=1	CAVPO	1
2699	2	2	27.91	27.91	27.91	tr|H0W2V7|H0W2V7_CAVPO	Uncharacterized protein OS=Cavia porcellus GN=NDUFA3 PE=4 SV=1	CAVPO	1
2700	2	2.53	21	13.5	10.5	tr|H0W3H4|H0W3H4_CAVPO	Uncharacterized protein OS=Cavia porcellus GN=LOC100726016 PE=4 SV=1	CAVPO	2
2701	2	2.01	18.13	18.13	18.13	tr|H0W4C5|H0W4C5_CAVPO	Uncharacterized protein OS=Cavia porcellus GN=TXN2 PE=4 SV=1	CAVPO	2
2702	2	2	4.783	4.783	4.783	tr|H0W5L6|H0W5L6_CAVPO	Uncharacterized protein (Fragment) OS=Cavia porcellus GN=SCAMP4 PE=4 SV=1	CAVPO	1
2703	2	2	8.553	8.553	8.553	tr|H0W5P1|H0W5P1_CAVPO	Uncharacterized protein OS=Cavia porcellus GN=PLP2 PE=4 SV=1	CAVPO	1

2704	2	2	6.593	2.747	2.747	tr|H0W6B7|H0W6B7_CAVPO	Uncharacterized protein OS=Cavia porcellus GN=SSBP4 PE=4 SV=1	CAVPO	1
2705	2	2	52.78	52.78	52.78	tr|H0W6V6|H0W6V6_CAVPO	Uncharacterized protein OS=Cavia porcellus GN=MARCKS PE=4 SV=1	CAVPO	1
2706	2	2	9.059	4.878	4.878	tr|H0W7C0|H0W7C0_CAVPO	Uncharacterized protein OS=Cavia porcellus GN=PHYHD1 PE=4 SV=1	CAVPO	1
2707	2	2	3.863	3.863	3.863	tr|H0W7N2|H0W7N2_CAVPO	Uncharacterized protein OS=Cavia porcellus GN=BCL2L1 PE=4 SV=1	CAVPO	1
2708	2	2	7.746	7.746	7.746	tr|H0W8R8|H0W8R8_CAVPO	Uncharacterized protein OS=Cavia porcellus GN=TXNL4A PE=4 SV=1	CAVPO	1
2709	2	2.03	36.51	25.4	25.4	tr|H0W9D8|H0W9D8_CAVPO	Uncharacterized protein OS=Cavia porcellus GN=COX17 PE=4 SV=1	CAVPO	1
2710	2	2	9.924	9.924	9.924	tr|H0W9K4|H0W9K4_CAVPO	Uncharacterized protein (Fragment) OS=Cavia porcellus GN=LEPROTL1 PE=4 SV=1	CAVPO	1
2711	2	2	1.518	1.518	1.518	tr|H0WAJ5|H0WAJ5_CAVPO	Uncharacterized protein OS=Cavia porcellus GN=PDP1 PE=3 SV=1	CAVPO	1
2712	2	2.01	7.123	4.843	3.134	tr|H0WAV2|H0WAV2_CAVPO	Uncharacterized protein OS=Cavia porcellus PE=4 SV=1	CAVPO	1
2713	2	2	12.02	12.02	12.02	tr|H0WB32|H0WB32_CAVPO	Uncharacterized protein OS=Cavia porcellus PE=3 SV=1	CAVPO	2
2714	2	2.01	5.777	1.513	1.513	tr|H0WB76|H0WB76_CAVPO	Uncharacterized protein OS=Cavia porcellus GN=PLOD1 PE=4 SV=1	CAVPO	1
2715	2	2	6.599	6.599	6.599	tr|H0WCN0|H0WCN0_CAVPO	Uncharacterized protein OS=Cavia porcellus GN=C11orf73 PE=4 SV=1	CAVPO	1
2716	2	2	8.054	8.054	8.054	tr|H0WCP2|H0WCP2_CAVPO	Uncharacterized protein OS=Cavia porcellus GN=OSTC PE=4 SV=1	CAVPO	1
2717	2	2	8.667	8.667	8.667	tr|H0WDW3|H0WDW3_CAVPO	Uncharacterized protein (Fragment) OS=Cavia porcellus GN=MCFD2 PE=4 SV=1	CAVPO	1

2718	2	22.4	46.17	38.69	30.29	tr|Q0QF16|Q0QF16_CAVPO	Succinate dehydrogenase complex subunit A (Fragment) OS=Cavia porcellus GN=SDHA PE=2 SV=1	CAVPO	13
2719	2	15.8	60.77	60.22	50.83	tr|Q80XX5|Q80XX5_CAVPO	Protein gene product 9.5 (Fragment) OS=Cavia porcellus PE=2 SV=1	CAVPO	12
2720	2	2	13.04	13.04	13.04	tr|Q9TEG4|Q9TEG4_CAVPO	NADH-ubiquinone oxidoreductase chain 3 (Fragment) OS=Cavia porcellus GN=NADH3 PE=3 SV=1	CAVPO	1
2721	2	2	3.448	3.448	3.448	tr|Q9TEG5|Q9TEG5_CAVPO	Cytochrome c oxidase subunit 3 (Fragment) OS=Cavia porcellus GN=COIII PE=3 SV=1	CAVPO	1
2722	1.98	2.07	8.918	2.778	1.754	tr|H0V5F1|H0V5F1_CAVPO	Uncharacterized protein OS=Cavia porcellus GN=EXOC7 PE=4 SV=1	CAVPO	1
2723	1.97	2.02	8.464	3.135	3.135	tr|H0V3E0|H0V3E0_CAVPO	Uncharacterized protein OS=Cavia porcellus GN=COQ9 PE=4 SV=1	CAVPO	1
2724	1.97	2.01	5.573	3.406	3.406	tr|H0V8X5|H0V8X5_CAVPO	Uncharacterized protein (Fragment) OS=Cavia porcellus GN=BABAM1 PE=4 SV=1	CAVPO	1
2725	1.97	6.95	24.02	15.23	5.859	tr|H0VJS5|H0VJS5_CAVPO	Uncharacterized protein OS=Cavia porcellus GN=LYN PE=4 SV=1	CAVPO	3
2726	1.97	2.06	15.69	10.04	1.883	tr|H0VTI4|H0VTI4_CAVPO	Uncharacterized protein OS=Cavia porcellus GN=AKT3 PE=4 SV=1	CAVPO	1
2727	1.97	2.02	3.182	0.7486	0.7486	tr|H0W041|H0W041_CAVPO	Uncharacterized protein OS=Cavia porcellus GN=AHDC1 PE=4 SV=1	CAVPO	1
2728	1.96	2	14.85	14.85	14.85	tr|H0V3G5|H0V3G5_CAVPO	Ubiquitin-related modifier 1 OS=Cavia porcellus GN=URM1 PE=1 SV=1	CAVPO	1
2729	1.96	2	1.488	1.488	1.488	tr|H0VI62|H0VI62_CAVPO	Uncharacterized protein (Fragment) OS=Cavia porcellus GN=COLEC12 PE=4 SV=1	CAVPO	1
2730	1.96	2.28	11.34	4.193	3.416	tr|H0VT97|H0VT97_CAVPO	Uncharacterized protein (Fragment) OS=Cavia porcellus GN=WDR70 PE=4 SV=1	CAVPO	2

2731	1.96	2.16	8.199	3.297	2.367	tr|H0VTX6|H0VTX6_CAVPO	Uncharacterized protein OS=Cavia porcellus GN=Gtf2a1l PE=4 SV=1	CAVPO	2
2732	1.96	2	4.197	1.825	1.825	tr|H0W011|H0W011_CAVPO	Uncharacterized protein OS=Cavia porcellus GN=GPR108 PE=4 SV=1	CAVPO	1
2733	1.95	2.04	4.466	1.326	1.326	tr|H0V515|H0V515_CAVPO	Uncharacterized protein (Fragment) OS=Cavia porcellus GN=RSF1 PE=4 SV=1	CAVPO	1
2734	1.95	2.07	17.09	14.55	6.909	tr|H0W4X6|H0W4X6_CAVPO	Uncharacterized protein OS=Cavia porcellus GN=ROGDI PE=4 SV=1	CAVPO	1
2735	1.94	2.13	7.269	7.269	5.305	tr|H0V682|H0V682_CAVPO	Uncharacterized protein OS=Cavia porcellus GN=MEF2D PE=3 SV=1	CAVPO	2
2736	1.93	2.08	5.948	4.833	4.833	tr|H0UT23|H0UT23_CAVPO	Uncharacterized protein OS=Cavia porcellus GN=NAPRT1 PE=4 SV=1	CAVPO	2
2737	1.93	2.01	10.04	6.024	6.024	tr|H0UTC8|H0UTC8_CAVPO	Uncharacterized protein OS=Cavia porcellus GN=TSPAN7 PE=4 SV=1	CAVPO	1
2738	1.93	2.09	6.566	4.377	4.377	tr|H0VB64|H0VB64_CAVPO	Uncharacterized protein (Fragment) OS=Cavia porcellus GN=SNX9 PE=4 SV=1	CAVPO	2
2739	1.93	2.01	39.6	16.83	16.83	tr|H0VI67|H0VI67_CAVPO	Uncharacterized protein (Fragment) OS=Cavia porcellus GN=ENY2 PE=3 SV=1	CAVPO	1
2740	1.92	4.06	8.866	8.866	4.824	tr|H0UYH6|H0UYH6_CAVPO	Uncharacterized protein OS=Cavia porcellus GN=SEC23B PE=4 SV=1	CAVPO	4
2741	1.92	2	8.276	8.276	8.276	tr|H0V9R1|H0V9R1_CAVPO	Uncharacterized protein (Fragment) OS=Cavia porcellus GN=LAGE3 PE=4 SV=1	CAVPO	1
2742	1.92	2	5.655	3.571	3.571	tr|H0VAF9|H0VAF9_CAVPO	Uncharacterized protein OS=Cavia porcellus GN=PHYH PE=4 SV=1	CAVPO	1
2743	1.92	2	7.205	4.899	4.899	tr|H0VJT5|H0VJT5_CAVPO	Uncharacterized protein OS=Cavia porcellus GN=HMG20A PE=4 SV=1	CAVPO	1
2744	1.92	2	8.333	8.333	8.333	tr|H0VLB7|H0VLB7_CAVPO	Uncharacterized protein (Fragment) OS=Cavia porcellus GN=REEP1 PE=4 SV=1	CAVPO	1

2745	1.92	6.22	29.44	18.57	6.897	tr|H0VMW3|H0VMW3_CAVPO	Uncharacterized protein OS=Cavia porcellus GN=GNA13 PE=4 SV=1	CAVPO	3
2746	1.92	2.07	19.05	11.43	11.43	tr|H0W0T2|H0W0T2_CAVPO	Uncharacterized protein OS=Cavia porcellus GN=EMC8 PE=4 SV=1	CAVPO	2
2747	1.91	3.93	32.48	18.47	10.19	tr|H0V0E2|H0V0E2_CAVPO	Uncharacterized protein (Fragment) OS=Cavia porcellus GN=ARGLU1 PE=4 SV=1	CAVPO	2
2748	1.91	24.9	19.51	17.85	13.82	tr|H0V645|H0V645_CAVPO	Uncharacterized protein (Fragment) OS=Cavia porcellus GN=ATP2B4 PE=4 SV=1	CAVPO	14
2749	1.91	1.98	6	2.2	2.2	tr|H0WBE1|H0WBE1_CAVPO	Uncharacterized protein OS=Cavia porcellus GN=LOC100715321 PE=3 SV=1	CAVPO	1
2750	1.9	2.05	11.66	8.282	4.908	tr|H0VKS0|H0VKS0_CAVPO	Uncharacterized protein (Fragment) OS=Cavia porcellus GN=USF2 PE=4 SV=1	CAVPO	1
2751	1.9	2.08	8.318	6.691	6.691	tr|H0VQI1|H0VQI1_CAVPO	Uncharacterized protein OS=Cavia porcellus GN=GNS PE=3 SV=1	CAVPO	4
2752	1.89	1.99	5.514	3.759	3.759	tr|H0VEY3|H0VEY3_CAVPO	Uncharacterized protein OS=Cavia porcellus GN=DAP3 PE=4 SV=1	CAVPO	1
2753	1.88	1.99	3.563	1.448	1.448	tr|H0VE90|H0VE90_CAVPO	Uncharacterized protein OS=Cavia porcellus GN=EPS15 PE=4 SV=1	CAVPO	1
2754	1.88	2.08	9.905	8	4.381	tr|H0VPN1|H0VPN1_CAVPO	Uncharacterized protein (Fragment) OS=Cavia porcellus GN=RPE65 PE=3 SV=1	CAVPO	2
2755	1.88	2.12	17.52	9.335	1.535	tr|H0W016|H0W016_CAVPO	Uncharacterized protein (Fragment) OS=Cavia porcellus GN=VPS51 PE=4 SV=1	CAVPO	1
2756	1.87	1.97	27.36	14.62	9.434	tr|H0V326|H0V326_CAVPO	Uncharacterized protein OS=Cavia porcellus GN=MED20 PE=4 SV=1	CAVPO	1
2757	1.87	1.98	8.554	4.684	4.684	tr|H0VB50|H0VB50_CAVPO	Uncharacterized protein (Fragment) OS=Cavia porcellus GN=PLEKHO2 PE=4 SV=1	CAVPO	1
2758	1.86	2.03	7.568	6.119	3.704	tr|H0VB61|H0VB61_CAVPO	Uncharacterized protein OS=Cavia porcellus GN=GPAA1 PE=4 SV=1	CAVPO	2

2759	1.86	2.09	7.174	4.636	1.049	tr|H0VS14|H0VS14_CAVPO	Uncharacterized protein OS=Cavia porcellus GN=PLXNB2 PE=4 SV=1	CAVPO	1
2760	1.86	1.96	6.197	2.778	2.778	tr|H0W319|H0W319_CAVPO	Uncharacterized protein OS=Cavia porcellus GN=SLC39A7 PE=4 SV=1	CAVPO	1
2761	1.85	2.07	10.55	10.55	5.275	tr|H0UZJ7|H0UZJ7_CAVPO	Uncharacterized protein OS=Cavia porcellus GN=SCPEP1 PE=4 SV=1	CAVPO	2
2762	1.85	1.96	3.501	1.681	1.681	tr|H0V233|H0V233_CAVPO	Uncharacterized protein OS=Cavia porcellus GN=CAPN1 PE=4 SV=1	CAVPO	1
2763	1.85	1.96	2.857	2.857	2.857	tr|H0VFI4|H0VFI4_CAVPO	Protein tweety homolog OS=Cavia porcellus GN=Ttyh3 PE=3 SV=1	CAVPO	1
2764	1.85	2.01	6.773	3.984	3.984	tr|H0VG94|H0VG94_CAVPO	Uncharacterized protein OS=Cavia porcellus GN=WASF3 PE=4 SV=1	CAVPO	2
2765	1.85	2	12.47	6.112	6.112	tr|H0VQH6|H0VQH6_CAVPO	Uncharacterized protein (Fragment) OS=Cavia porcellus GN=Pcid2 PE=4 SV=1	CAVPO	2
2766	1.85	1.96	6.552	6.552	6.552	tr|H0VZ63|H0VZ63_CAVPO	Uncharacterized protein OS=Cavia porcellus GN=Tmx2 PE=4 SV=1	CAVPO	1
2767	1.85	1.96	41.13	8.871	8.871	tr|H0W6C3|H0W6C3_CAVPO	Uncharacterized protein OS=Cavia porcellus GN=LOC100729172 PE=4 SV=1	CAVPO	1
2768	1.85	1.96	13.82	13.82	13.82	tr|H0WDI3|H0WDI3_CAVPO	Uncharacterized protein (Fragment) OS=Cavia porcellus PE=4 SV=1	CAVPO	1
2769	1.84	2	23.45	14.48	14.48	tr|H0UXL1|H0UXL1_CAVPO	Uncharacterized protein OS=Cavia porcellus GN=TRAPPC1 PE=4 SV=1	CAVPO	2
2770	1.84	2.06	5.639	4.289	2.065	tr|H0UZK5|H0UZK5_CAVPO	Uncharacterized protein (Fragment) OS=Cavia porcellus GN=SORBS1 PE=4 SV=1	CAVPO	2
2771	1.84	1.94	11.94	5.97	5.97	tr|H0VBH6|H0VBH6_CAVPO	Uncharacterized protein (Fragment) OS=Cavia porcellus GN=NUBP2 PE=3 SV=1	CAVPO	1
2772	1.84	2	7.509	7.509	7.509	tr|H0VVR0|H0VVR0_CAVPO	Uncharacterized protein (Fragment) OS=Cavia porcellus GN=QPRT PE=4 SV=1	CAVPO	2

2773	1.83	1.96	9.729	7.692	3.394	tr|H0VHJ0|H0VHJ0_CAVPO	Uncharacterized protein OS=Cavia porcellus GN=PRRC1 PE=4 SV=1	CAVPO	1
2774	1.83	1.98	17.13	9.562	9.562	tr|H0VZW3|H0VZW3_CAVPO	Uncharacterized protein OS=Cavia porcellus GN=C11orf68 PE=4 SV=1	CAVPO	2
2775	1.83	1.94	14.35	3.797	3.797	tr|H0W8X1|H0W8X1_CAVPO	Uncharacterized protein OS=Cavia porcellus GN=NHEJ1 PE=4 SV=1	CAVPO	1
2776	1.82	1.98	5.684	4	4	tr|H0UXY7|H0UXY7_CAVPO	Uncharacterized protein OS=Cavia porcellus GN=GABRG2 PE=3 SV=1	CAVPO	2
2777	1.82	1.98	10.84	7.74	7.74	tr|H0UYS9|H0UYS9_CAVPO	Uncharacterized protein OS=Cavia porcellus GN=CACNG2 PE=4 SV=1	CAVPO	2
2778	1.82	1.91	18.01	18.01	13.66	tr|H0VA36|H0VA36_CAVPO	Uncharacterized protein (Fragment) OS=Cavia porcellus GN=FAM96B PE=4 SV=1	CAVPO	1
2779	1.82	1.92	16.75	8.134	8.134	tr|H0WAT7|H0WAT7_CAVPO	Uncharacterized protein OS=Cavia porcellus GN=LOC100720044 PE=4 SV=1	CAVPO	1
2780	1.82	1.92	10.53	10.53	10.53	tr|H0WCB1|H0WCB1_CAVPO	Uncharacterized protein OS=Cavia porcellus GN=CYSTM1 PE=4 SV=1	CAVPO	1
2781	1.81	4.16	19.21	8.884	8.884	tr|H0V8W0|H0V8W0_CAVPO	Oxysterol-binding protein OS=Cavia porcellus GN=OSBPL2 PE=3 SV=1	CAVPO	3
2782	1.81	1.98	10.43	5.298	5.298	tr|H0VFF6|H0VFF6_CAVPO	Uncharacterized protein OS=Cavia porcellus GN=Scg2 PE=4 SV=1	CAVPO	2
2783	1.8	1.91	9.47	6.421	2.889	tr|H0UVU0|H0UVU0_CAVPO	Uncharacterized protein OS=Cavia porcellus GN=KIAA1467 PE=4 SV=1	CAVPO	1
2784	1.8	1.89	8.547	8.547	8.547	tr|H0UW44|H0UW44_CAVPO	Uncharacterized protein OS=Cavia porcellus GN=POLR2J PE=4 SV=1	CAVPO	1
2785	1.8	1.89	6.355	4.013	4.013	tr|H0UWT5|H0UWT5_CAVPO	Uncharacterized protein OS=Cavia porcellus GN=GGPS1 PE=3 SV=1	CAVPO	1
2786	1.8	2.18	4.826	3.475	2.317	tr|H0UZ06|H0UZ06_CAVPO	Uncharacterized protein OS=Cavia porcellus GN=VEZF1 PE=4 SV=1	CAVPO	1

2787	1.8	4.4	13.72	7.337	4.212	tr|H0VV11|H0VV11_CAVPO	Protein kinase C epsilon type OS=Cavia porcellus GN=PRKCE PE=3 SV=1	CAVPO	3
2788	1.8	1.9	4.871	3.152	3.152	tr|H0VZQ8|H0VZQ8_CAVPO	Uncharacterized protein (Fragment) OS=Cavia porcellus PE=4 SV=1	CAVPO	1
2789	1.8	1.89	12.93	3.155	3.155	tr|H0W151|H0W151_CAVPO	Uncharacterized protein (Fragment) OS=Cavia porcellus GN=IGLON5 PE=4 SV=1	CAVPO	1
2790	1.79	12.1	57.05	42.95	29.53	tr|H0VQP0|H0VQP0_CAVPO	Uncharacterized protein OS=Cavia porcellus GN=CALML3 PE=4 SV=1	CAVPO	7
2791	1.79	1.92	7.9	3.95	1.351	tr|H0VRE9|H0VRE9_CAVPO	Uncharacterized protein OS=Cavia porcellus GN=ARVCF PE=4 SV=1	CAVPO	1
2792	1.79	1.87	14.85	6.931	6.931	tr|H0VRY6|H0VRY6_CAVPO	Uncharacterized protein OS=Cavia porcellus GN=ISOC2 PE=4 SV=1	CAVPO	1
2793	1.78	1.87	51.85	38.89	20.37	tr|H0V106|H0V106_CAVPO	Uncharacterized protein OS=Cavia porcellus GN=RPS29 PE=4 SV=1	CAVPO	1
2794	1.78	1.94	11.53	11.53	4.511	tr|H0VA04|H0VA04_CAVPO	Uncharacterized protein OS=Cavia porcellus GN=SEC62 PE=4 SV=1	CAVPO	2
2795	1.78	1.9	6.901	3.125	1.563	tr|H0VC04|H0VC04_CAVPO	Uncharacterized protein (Fragment) OS=Cavia porcellus GN=PDE8A PE=4 SV=1	CAVPO	1
2796	1.78	2.05	11.14	11.14	4.106	tr|H0VCF9|H0VCF9_CAVPO	Uncharacterized protein OS=Cavia porcellus GN=THOC6 PE=4 SV=1	CAVPO	1
2797	1.77	1.91	3.896	2.381	1.19	tr|H0V195|H0V195_CAVPO	Uncharacterized protein OS=Cavia porcellus GN=EXOC2 PE=4 SV=1	CAVPO	1
2798	1.77	4.12	8.893	4.53	2.349	tr|H0VKQ9|H0VKQ9_CAVPO	Uncharacterized protein OS=Cavia porcellus GN=PKP4 PE=4 SV=1	CAVPO	2
2799	1.77	1.96	14.12	14.12	3.435	tr|H0VYG6|H0VYG6_CAVPO	Uncharacterized protein OS=Cavia porcellus GN=CA13 PE=4 SV=1	CAVPO	1
2800	1.76	2.88	18.72	6.828	3.965	tr|H0V3R7|H0V3R7_CAVPO	Uncharacterized protein OS=Cavia porcellus GN=GABPA PE=3 SV=1	CAVPO	2

2801	1.76	1.9	27.61	12.88	7.362	tr|H0VJ39|H0VJ39_CAVPO	Uncharacterized protein OS=Cavia porcellus GN=ARL2BP PE=4 SV=1	CAVPO	1
2802	1.74	1.87	47.37	47.37	47.37	tr|H0VI06|H0VI06_CAVPO	Uncharacterized protein OS=Cavia porcellus GN=PKIA PE=4 SV=1	CAVPO	2
2803	1.74	1.88	18.26	8.982	4.79	tr|H0VLZ2|H0VLZ2_CAVPO	Uncharacterized protein (Fragment) OS=Cavia porcellus GN=GRHPR PE=3 SV=1	CAVPO	1
2804	1.74	1.82	4.036	1.495	1.495	tr|H0VNU2|H0VNU2_CAVPO	Uncharacterized protein OS=Cavia porcellus GN=DYM PE=4 SV=1	CAVPO	1
2805	1.73	1.86	8.108	2.838	1.486	tr|H0UZ14|H0UZ14_CAVPO	Uncharacterized protein (Fragment) OS=Cavia porcellus GN=PAPOLA PE=4 SV=1	CAVPO	1
2806	1.72	1.97	18.32	11.82	3.767	tr|H0V429|H0V429_CAVPO	Uncharacterized protein OS=Cavia porcellus GN=EPN2 PE=4 SV=1	CAVPO	2
2807	1.72	1.8	2.693	2.693	2.693	tr|H0VKE4|H0VKE4_CAVPO	Uncharacterized protein (Fragment) OS=Cavia porcellus GN=SLC24A3 PE=4 SV=1	CAVPO	1
2808	1.72	8.55	28.84	28.84	28.84	tr|H0W6H9|H0W6H9_CAVPO	Uncharacterized protein OS=Cavia porcellus GN=HIST1H1A PE=3 SV=1	CAVPO	6
2809	1.72	1.82	22.87	13.83	13.83	tr|H0W843|H0W843_CAVPO	60S ribosomal protein L18 (Fragment) OS=Cavia porcellus GN=RPL18 PE=3 SV=1	CAVPO	2
2810	1.7	1.8	2.759	2.759	2.759	tr|H0VEG0|H0VEG0_CAVPO	Uncharacterized protein (Fragment) OS=Cavia porcellus GN=GLOD4 PE=4 SV=1	CAVPO	1
2811	1.7	1.8	6.587	4.192	4.192	tr|H0VKS4|H0VKS4_CAVPO	Uncharacterized protein OS=Cavia porcellus GN=RDH13 PE=3 SV=1	CAVPO	1
2812	1.69	1.84	10.57	6.857	2.857	tr|H0VAF4|H0VAF4_CAVPO	Uncharacterized protein OS=Cavia porcellus GN=RP2 PE=4 SV=1	CAVPO	1
2813	1.69	1.79	11.62	5.808	3.03	tr|H0VCQ9|H0VCQ9_CAVPO	Uncharacterized protein (Fragment) OS=Cavia porcellus GN=C9orf41 PE=4 SV=1	CAVPO	1
2814	1.69	2.01	6.289	5.464	2.371	tr|H0W4M4|H0W4M4_CAVPO	Uncharacterized protein OS=Cavia porcellus GN=Slc8a1 PE=4 SV=1	CAVPO	2

2815	1.68	1.79	12.67	1.811	1.811	tr|H0UTI1|H0UTI1_CAVPO	Uncharacterized protein OS=Cavia porcellus GN=HOOK3 PE=4 SV=1	CAVPO	1
2816	1.68	1.75	3.623	1.863	1.863	tr|H0UYY7|H0UYY7_CAVPO	Uncharacterized protein OS=Cavia porcellus GN=CTDP1 PE=4 SV=1	CAVPO	1
2817	1.68	1.84	15.53	11.17	5.722	tr|H0V5M3|H0V5M3_CAVPO	Uncharacterized protein OS=Cavia porcellus GN=PCYT1A PE=4 SV=1	CAVPO	1
2818	1.68	1.77	3.969	1.639	0.9491	tr|H0VDJ2|H0VDJ2_CAVPO	Uncharacterized protein OS=Cavia porcellus GN=HDAC6 PE=4 SV=1	CAVPO	1
2819	1.68	1.77	12.9	6.989	6.989	tr|H0W4A3|H0W4A3_CAVPO	Uncharacterized protein (Fragment) OS=Cavia porcellus GN=MEA1 PE=4 SV=1	CAVPO	1
2820	1.68	1.78	4.305	2.152	2.152	tr|H0W9B1|H0W9B1_CAVPO	Uncharacterized protein (Fragment) OS=Cavia porcellus GN=SEMA7A PE=4 SV=1	CAVPO	1
2821	1.67	1.76	12.97	3.956	3.956	tr|H0UXD5|H0UXD5_CAVPO	Uncharacterized protein OS=Cavia porcellus GN=DDX47 PE=3 SV=1	CAVPO	1
2822	1.67	1.76	9.717	5.668	5.668	tr|H0VIP7|H0VIP7_CAVPO	Uncharacterized protein OS=Cavia porcellus GN=TIMM21 PE=4 SV=1	CAVPO	1
2823	1.67	1.91	6.781	4.82	1.062	tr|H0VZ04|H0VZ04_CAVPO	Uncharacterized protein OS=Cavia porcellus GN=PPP1R9B PE=4 SV=1	CAVPO	1
2824	1.66	1.84	5	4.327	3.462	tr|H0UUN3|H0UUN3_CAVPO	Uncharacterized protein OS=Cavia porcellus GN=IPO9 PE=4 SV=1	CAVPO	2
2825	1.66	1.82	12.83	7.965	4.867	tr|H0VLM2|H0VLM2_CAVPO	Uncharacterized protein OS=Cavia porcellus GN=Ilk PE=4 SV=1	CAVPO	2
2826	1.66	1.76	9.333	3.238	3.238	tr|H0VR41|H0VR41_CAVPO	Uncharacterized protein OS=Cavia porcellus GN=FBXO7 PE=4 SV=1	CAVPO	1
2827	1.66	1.76	15.66	15.66	8.434	tr|H0VRN7|H0VRN7_CAVPO	Uncharacterized protein OS=Cavia porcellus GN=MRPL49 PE=4 SV=1	CAVPO	1
2828	1.66	1.74	4.494	4.494	4.494	tr|H0VV44|H0VV44_CAVPO	Proteasome assembly chaperone 2 OS=Cavia porcellus GN=PSMG2 PE=3 SV=1	CAVPO	1

2829	1.66	1.74	5.338	5.338	5.338	tr|H0VV49|H0VV49_CAVPO	Uncharacterized protein OS=Cavia porcellus GN=WBSCR22 PE=4 SV=1	CAVPO	1
2830	1.65	1.81	8.087	6.065	4.51	tr|H0V2U3|H0V2U3_CAVPO	Uncharacterized protein (Fragment) OS=Cavia porcellus GN=CAPN5 PE=4 SV=1	CAVPO	2
2831	1.65	1.78	7.494	2.685	1.342	tr|H0V305|H0V305_CAVPO	Phosphatidylinositol 3-kinase OS=Cavia porcellus GN=PIK3C3 PE=3 SV=1	CAVPO	1
2832	1.65	1.76	6.567	4.925	2.985	tr|H0VHH6|H0VHH6_CAVPO	Uncharacterized protein (Fragment) OS=Cavia porcellus GN=ABCB7 PE=3 SV=1	CAVPO	1
2833	1.65	1.74	7.895	4.386	4.386	tr|H0VQX0|H0VQX0_CAVPO	Uncharacterized protein OS=Cavia porcellus GN=CCDC97 PE=4 SV=1	CAVPO	1
2834	1.65	1.75	11.22	6.341	6.341	tr|H0WAM5|H0WAM5_CAVPO	Uncharacterized protein (Fragment) OS=Cavia porcellus GN=RBFOX2 PE=4 SV=1	CAVPO	1
2835	1.64	1.75	14.76	7.619	2.381	tr|H0UXE0|H0UXE0_CAVPO	Uncharacterized protein OS=Cavia porcellus GN=RBM22 PE=4 SV=1	CAVPO	1
2836	1.64	1.78	10.81	7.143	1.832	tr|H0V1K8|H0V1K8_CAVPO	Uncharacterized protein OS=Cavia porcellus GN=ELP3 PE=4 SV=1	CAVPO	1
2837	1.64	1.75	1.655	0.5079	0.1966	tr|H0V266|H0V266_CAVPO	Uncharacterized protein (Fragment) OS=Cavia porcellus GN=GPR98 PE=4 SV=1	CAVPO	1
2838	1.64	1.81	3.122	2.153	1.292	tr|H0V6N3|H0V6N3_CAVPO	Uncharacterized protein OS=Cavia porcellus GN=PIGN PE=4 SV=1	CAVPO	1
2839	1.64	1.82	1.683	0.6731	0.3846	tr|H0VPW4|H0VPW4_CAVPO	Uncharacterized protein OS=Cavia porcellus GN=RALGAPA1 PE=4 SV=1	CAVPO	1
2840	1.63	1.73	3.477	1.987	1.987	tr|H0V8J8|H0V8J8_CAVPO	Uncharacterized protein (Fragment) OS=Cavia porcellus GN=LRRFIP1 PE=4 SV=1	CAVPO	1
2841	1.63	1.71	20.47	5.848	5.848	tr|H0VN21|H0VN21_CAVPO	Uncharacterized protein (Fragment) OS=Cavia porcellus GN=SPRYD7 PE=4 SV=1	CAVPO	1
2842	1.62	3.61	8.987	5.163	5.163	tr|H0VI87|H0VI87_CAVPO	Uncharacterized protein (Fragment) OS=Cavia porcellus GN=TXNRD2 PE=3 SV=1	CAVPO	2

2843	1.62	1.79	16.88	5.928	3.866	tr|H0W0H3|H0W0H3_CAVPO	Uncharacterized protein OS=Cavia porcellus GN=CUL1 PE=3 SV=1	CAVPO	2
2844	1.62	1.72	5.534	4.743	4.743	tr|H0W3V2|H0W3V2_CAVPO	Uncharacterized protein (Fragment) OS=Cavia porcellus GN=TMUB1 PE=4 SV=1	CAVPO	1
2845	1.61	1.88	23.44	18.1	4.748	tr|H0UWI9|H0UWI9_CAVPO	Uncharacterized protein OS=Cavia porcellus GN=ABHD6 PE=4 SV=1	CAVPO	1
2846	1.61	3.83	25.35	18.78	12.68	tr|H0VHL4|H0VHL4_CAVPO	Uncharacterized protein OS=Cavia porcellus GN=RAB39B PE=3 SV=1	CAVPO	4
2847	1.6	1.7	2.913	0.9015	0.9015	tr|H0UVH5|H0UVH5_CAVPO	Uncharacterized protein OS=Cavia porcellus GN=NCOA1 PE=4 SV=1	CAVPO	1
2848	1.6	1.88	27.5	20.83	5.417	tr|H0V2B4|H0V2B4_CAVPO	Uncharacterized protein OS=Cavia porcellus GN=GSTT1 PE=3 SV=1	CAVPO	1
2849	1.6	1.78	9.747	5.316	4.304	tr|H0V2C9|H0V2C9_CAVPO	Uncharacterized protein (Fragment) OS=Cavia porcellus GN=AGAP3 PE=4 SV=1	CAVPO	2
2850	1.6	1.75	5.33	3.303	1.652	tr|H0V6K7|H0V6K7_CAVPO	Uncharacterized protein OS=Cavia porcellus GN=IKBKAP PE=4 SV=1	CAVPO	1
2851	1.6	1.7	20.29	10.14	10.14	tr|H0VRH7|H0VRH7_CAVPO	Uncharacterized protein OS=Cavia porcellus GN=GOLT1B PE=4 SV=1	CAVPO	1
2852	1.6	1.68	2.302	2.302	2.302	tr|H0VSP7|H0VSP7_CAVPO	Uncharacterized protein (Fragment) OS=Cavia porcellus GN=SP4 PE=4 SV=1	CAVPO	1
2853	1.6	1.72	11.72	2.241	2.241	tr|H0VTD7|H0VTD7_CAVPO	Uncharacterized protein OS=Cavia porcellus GN=NELFB PE=4 SV=1	CAVPO	1
2854	1.6	1.73	4.732	2.681	1.42	tr|H0VU96|H0VU96_CAVPO	Uncharacterized protein OS=Cavia porcellus GN=KLHL22 PE=4 SV=1	CAVPO	1
2855	1.59	1.69	5.045	1.141	0.7207	tr|H0V0H5|H0V0H5_CAVPO	Uncharacterized protein (Fragment) OS=Cavia porcellus GN=Ankrd26 PE=4 SV=1	CAVPO	1
2856	1.59	1.68	2.128	2.128	2.128	tr|H0V8K1|H0V8K1_CAVPO	Uncharacterized protein OS=Cavia porcellus GN=DAXX PE=4 SV=1	CAVPO	1

2857	1.59	1.74	4.332	4.332	4.332	tr|H0W643|H0W643_CAVPO	Uncharacterized protein (Fragment) OS=Cavia porcellus GN=C20orf112 PE=4 SV=1	CAVPO	2
2858	1.59	1.71	6.988	4.337	2.41	tr|H0WCZ4|H0WCZ4_CAVPO	Uncharacterized protein OS=Cavia porcellus GN=CSNK1G2 PE=3 SV=1	CAVPO	1
2859	1.58	1.76	8.25	4.771	1.193	tr|H0VMT6|H0VMT6_CAVPO	DNA ligase OS=Cavia porcellus GN=LIG3 PE=3 SV=1	CAVPO	1
2860	1.57	1.66	5.512	5.512	5.512	tr|H0UU18|H0UU18_CAVPO	Uncharacterized protein OS=Cavia porcellus GN=ETHE1 PE=4 SV=1	CAVPO	1
2861	1.57	1.65	7.194	1.871	1.871	tr|H0UZF7|H0UZF7_CAVPO	Uncharacterized protein OS=Cavia porcellus GN=NUFIP2 PE=4 SV=1	CAVPO	1
2862	1.57	1.65	2.628	1.445	1.445	tr|H0V7H6|H0V7H6_CAVPO	Uncharacterized protein (Fragment) OS=Cavia porcellus GN=LOC100729034 PE=4 SV=1	CAVPO	1
2863	1.57	6.6	32.57	21.56	21.56	tr|H0VQQ4|H0VQQ4_CAVPO	Uncharacterized protein OS=Cavia porcellus GN=RAB4A PE=3 SV=1	CAVPO	6
2864	1.57	1.76	7.468	5.519	3.571	tr|H0VYF3|H0VYF3_CAVPO	Uncharacterized protein OS=Cavia porcellus GN=RTFDC1 PE=4 SV=1	CAVPO	1
2865	1.57	1.64	9.79	7.692	7.692	tr|H0W2M3|H0W2M3_CAVPO	Uncharacterized protein (Fragment) OS=Cavia porcellus GN=Rps23 PE=3 SV=1	CAVPO	1
2866	1.57	1.64	7.258	7.258	7.258	tr|H0W8Q0|H0W8Q0_CAVPO	Uncharacterized protein (Fragment) OS=Cavia porcellus GN=FDX1 PE=4 SV=1	CAVPO	1
2867	1.55	1.69	19.77	13.95	5.426	tr|H0V061|H0V061_CAVPO	Uncharacterized protein OS=Cavia porcellus GN=SNAP29 PE=4 SV=1	CAVPO	1
2868	1.55	1.72	16.98	10.61	2.918	tr|H0V3Y0|H0V3Y0_CAVPO	Uncharacterized protein OS=Cavia porcellus GN=Nck1 PE=4 SV=1	CAVPO	1
2869	1.54	1.62	4.633	4.633	4.633	tr|H0V0E8|H0V0E8_CAVPO	Uncharacterized protein (Fragment) OS=Cavia porcellus GN=SCD5 PE=3 SV=1	CAVPO	1
2870	1.54	1.63	3.046	1.32	1.32	tr|H0VF52|H0VF52_CAVPO	Alanine--tRNA ligase, cytoplasmic OS=Cavia porcellus GN=AARS2 PE=3 SV=1	CAVPO	1

2871	1.54	1.63	42.47	42.47	42.47	tr|H0W113|H0W113_CAVPO	Uncharacterized protein OS=Cavia porcellus GN=HOPX PE=3 SV=1	CAVPO	2
2872	1.53	1.7	9.873	9.873	1.592	tr|H0VP15|H0VP15_CAVPO	Uncharacterized protein OS=Cavia porcellus GN=BCAM PE=4 SV=1	CAVPO	1
2873	1.52	1.69	8.761	8.761	6.344	tr|H0UUF1|H0UUF1_CAVPO	Uncharacterized protein (Fragment) OS=Cavia porcellus GN=RABGGTB PE=4 SV=1	CAVPO	2
2874	1.52	1.65	12.71	12.71	9.322	tr|H0UVY3|H0UVY3_CAVPO	Uncharacterized protein OS=Cavia porcellus GN=YIPF6 PE=4 SV=1	CAVPO	2
2875	1.52	1.6	18.52	18.52	12.96	tr|H0UW76|H0UW76_CAVPO	Uncharacterized protein (Fragment) OS=Cavia porcellus GN=SH3BGRL2 PE=4 SV=1	CAVPO	1
2876	1.52	1.71	9.171	2.099	1.105	tr|H0V9H5|H0V9H5_CAVPO	Uncharacterized protein OS=Cavia porcellus GN=CNTROB PE=4 SV=1	CAVPO	1
2877	1.52	1.6	4.933	4.933	4.933	tr|H0VYU9|H0VYU9_CAVPO	Uncharacterized protein OS=Cavia porcellus GN=NTMT1 PE=4 SV=1	CAVPO	1
2878	1.52	1.66	33.64	20	20	tr|H0W915|H0W915_CAVPO	Parvalbumin alpha OS=Cavia porcellus GN=PVALB PE=4 SV=1	CAVPO	2
2879	1.51	1.61	8.664	8.664	3.249	tr|H0V9C3|H0V9C3_CAVPO	Uncharacterized protein OS=Cavia porcellus GN=RSU1 PE=4 SV=1	CAVPO	1
2880	1.51	1.59	6.452	6.452	6.452	tr|H0VSR1|H0VSR1_CAVPO	Uncharacterized protein OS=Cavia porcellus GN=CA6 PE=4 SV=1	CAVPO	1
2881	1.51	1.72	12.4	8.527	5.426	tr|H0W400|H0W400_CAVPO	Uncharacterized protein OS=Cavia porcellus GN=SNF8 PE=4 SV=1	CAVPO	1
2882	1.51	1.59	21.21	21.21	21.21	tr|H0W6X9|H0W6X9_CAVPO	Uncharacterized protein (Fragment) OS=Cavia porcellus GN=FXYD1 PE=4 SV=1	CAVPO	1
2883	1.5	1.64	12.08	6.144	1.059	tr|H0UWZ9|H0UWZ9_CAVPO	Uncharacterized protein OS=Cavia porcellus GN=UNC45A PE=4 SV=1	CAVPO	1
2884	1.5	2.5	14.69	7.992	4.695	tr|H0V4Z3|H0V4Z3_CAVPO	Uncharacterized protein OS=Cavia porcellus GN=RNF40 PE=4 SV=1	CAVPO	3

2885	1.5	1.61	7.714	3.226	1.683	tr|H0VXQ9|H0VXQ9_CAVPO	Uncharacterized protein (Fragment) OS=Cavia porcellus GN=BRSK2 PE=4 SV=1	CAVPO	1
2886	1.49	1.59	4.14	4.14	4.14	tr|H0UUP1|H0UUP1_CAVPO	Uncharacterized protein (Fragment) OS=Cavia porcellus GN=Rdh10 PE=3 SV=1	CAVPO	1
2887	1.49	1.59	7.077	7.077	4.308	tr|H0UWL4|H0UWL4_CAVPO	Uncharacterized protein OS=Cavia porcellus GN=TMED8 PE=4 SV=1	CAVPO	1
2888	1.49	1.58	5.642	0.7052	0.7052	tr|H0VBM3|H0VBM3_CAVPO	Uncharacterized protein (Fragment) OS=Cavia porcellus GN=UACA PE=4 SV=1	CAVPO	1
2889	1.49	1.58	2.259	1.335	1.335	tr|H0VCC6|H0VCC6_CAVPO	Uncharacterized protein OS=Cavia porcellus GN=PITPNM3 PE=4 SV=1	CAVPO	1
2890	1.49	1.65	8.601	5.248	3.936	tr|H0VF84|H0VF84_CAVPO	Uncharacterized protein OS=Cavia porcellus GN=AKAP8 PE=4 SV=1	CAVPO	2
2891	1.49	1.59	10.44	4.016	4.016	tr|H0VL55|H0VL55_CAVPO	Uncharacterized protein OS=Cavia porcellus GN=ING4 PE=4 SV=1	CAVPO	1
2892	1.49	1.58	7.338	2.725	2.725	tr|H0VLF6|H0VLF6_CAVPO	Uncharacterized protein (Fragment) OS=Cavia porcellus GN=NRBP2 PE=4 SV=1	CAVPO	1
2893	1.49	1.57	9.091	9.091	9.091	tr|H0VQ57|H0VQ57_CAVPO	Uncharacterized protein OS=Cavia porcellus GN=Fabp7 PE=3 SV=1	CAVPO	1
2894	1.49	1.73	23.79	10.13	3.524	tr|H0VWJ1|H0VWJ1_CAVPO	Uncharacterized protein OS=Cavia porcellus GN=LOC100714943 PE=4 SV=1	CAVPO	1
2895	1.48	1.62	1.766	0.9566	0.9566	tr|H0V6H9|H0V6H9_CAVPO	Uncharacterized protein OS=Cavia porcellus GN=KIAA1462 PE=4 SV=1	CAVPO	2
2896	1.48	1.65	17.78	17.78	11.67	tr|H0VCL1|H0VCL1_CAVPO	Uncharacterized protein OS=Cavia porcellus GN=SPCS3 PE=4 SV=1	CAVPO	2
2897	1.48	1.63	17.88	12.58	12.58	tr|H0VRQ2|H0VRQ2_CAVPO	Uncharacterized protein OS=Cavia porcellus GN=AIF1L PE=4 SV=1	CAVPO	2
2898	1.48	1.56	4.225	2.414	2.414	tr|H0VY18|H0VY18_CAVPO	Uncharacterized protein OS=Cavia porcellus GN=NACC1 PE=4 SV=1	CAVPO	1

2899	1.48	1.58	6.697	1.37	1.37	tr|H0W9I0|H0W9I0_CAVPO	Uncharacterized protein OS=Cavia porcellus GN=ABCG5 PE=3 SV=1	CAVPO	1
2900	1.47	1.62	13.85	11.36	4.155	tr|H0UZK1|H0UZK1_CAVPO	Uncharacterized protein OS=Cavia porcellus GN=NSDHL PE=3 SV=1	CAVPO	1
2901	1.47	1.55	4.669	4.669	4.669	tr|H0V8M3|H0V8M3_CAVPO	Uncharacterized protein (Fragment) OS=Cavia porcellus GN=PIGX PE=4 SV=1	CAVPO	1
2902	1.47	1.58	8.264	4.339	2.273	tr|H0V8P0|H0V8P0_CAVPO	Uncharacterized protein OS=Cavia porcellus GN=THNSL2 PE=4 SV=1	CAVPO	1
2903	1.47	1.6	18.59	6.197	2.817	tr|H0VC23|H0VC23_CAVPO	Uncharacterized protein OS=Cavia porcellus GN=STX5 PE=3 SV=1	CAVPO	1
2904	1.47	17.5	36.97	36.67	33.94	tr|H0VIK4|H0VIK4_CAVPO	Serine/threonine-protein phosphatase OS=Cavia porcellus GN=PPP1CA PE=3 SV=1	CAVPO	9
2905	1.47	1.62	5.371	3.886	1.943	tr|H0VJW2|H0VJW2_CAVPO	Uncharacterized protein OS=Cavia porcellus GN=MIOS PE=4 SV=1	CAVPO	2
2906	1.47	1.55	56.76	18.92	18.92	tr|H0W1Q3|H0W1Q3_CAVPO	Uncharacterized protein OS=Cavia porcellus GN=TOMM6 PE=4 SV=1	CAVPO	1
2907	1.47	1.56	6.706	6.706	2.17	tr|H0W9X7|H0W9X7_CAVPO	Uncharacterized protein OS=Cavia porcellus GN=TCP11L1 PE=4 SV=1	CAVPO	1
2908	1.46	1.55	41.41	31.31	31.31	tr|H0VLD2|H0VLD2_CAVPO	Uncharacterized protein (Fragment) OS=Cavia porcellus GN=HMGN3 PE=4 SV=1	CAVPO	2
2909	1.44	1.54	5.866	1.536	1.536	tr|H0UYU8|H0UYU8_CAVPO	Uncharacterized protein OS=Cavia porcellus GN=MAD1L1 PE=4 SV=1	CAVPO	1
2910	1.44	1.52	3.939	3.939	3.939	tr|H0VER6|H0VER6_CAVPO	Uncharacterized protein (Fragment) OS=Cavia porcellus GN=PLSCR4 PE=4 SV=1	CAVPO	1
2911	1.44	1.53	5.472	3.962	2.642	tr|H0W0F2|H0W0F2_CAVPO	Uncharacterized protein (Fragment) OS=Cavia porcellus GN=BTD PE=4 SV=1	CAVPO	1
2912	1.44	1.61	6.375	1.57	0.4282	tr|H0W3I1|H0W3I1_CAVPO	Uncharacterized protein (Fragment) OS=Cavia porcellus GN=PDE4DIP PE=4 SV=1	CAVPO	1

2913	1.44	1.58	11.22	9.756	3.171	tr|H0W7G7|H0W7G7_CAVPO	Uncharacterized protein OS=Cavia porcellus GN=RMDN2 PE=4 SV=1	CAVPO	1
2914	1.43	1.52	12.47	2.036	2.036	tr|H0VH23|H0VH23_CAVPO	Uncharacterized protein OS=Cavia porcellus GN=Kin PE=4 SV=1	CAVPO	1
2915	1.43	1.55	10.33	1.976	1.976	tr|H0VYW1|H0VYW1_CAVPO	Uncharacterized protein OS=Cavia porcellus PE=4 SV=1	CAVPO	1
2916	1.43	1.63	18.87	14.37	2.817	tr|H0W5G9|H0W5G9_CAVPO	Uncharacterized protein (Fragment) OS=Cavia porcellus GN=MTX1 PE=4 SV=1	CAVPO	1
2917	1.42	1.53	7.674	7.674	4.556	tr|H0V0B9|H0V0B9_CAVPO	Uncharacterized protein (Fragment) OS=Cavia porcellus GN=IGSF11 PE=4 SV=1	CAVPO	1
2918	1.42	1.59	9.378	4.177	1.279	tr|H0VDN6|H0VDN6_CAVPO	Uncharacterized protein OS=Cavia porcellus GN=CTR9 PE=4 SV=1	CAVPO	1
2919	1.42	1.49	4.669	4.669	4.669	tr|H0VE62|H0VE62_CAVPO	Uncharacterized protein OS=Cavia porcellus GN=YIPF5 PE=4 SV=1	CAVPO	1
2920	1.41	1.49	13.41	13.41	13.41	tr|H0VIC3|H0VIC3_CAVPO	Uncharacterized protein (Fragment) OS=Cavia porcellus GN=TIMM8B PE=4 SV=1	CAVPO	1
2921	1.41	1.52	8.115	4.296	2.148	tr|H0VJG9|H0VJG9_CAVPO	Uncharacterized protein OS=Cavia porcellus GN=ANKRD10 PE=4 SV=1	CAVPO	1
2922	1.4	6.2	16.88	12.86	8.521	tr|H0VS09|H0VS09_CAVPO	Uncharacterized protein OS=Cavia porcellus GN=KLC2 PE=4 SV=1	CAVPO	4
2923	1.4	1.49	26.89	9.244	9.244	tr|H0VWH9|H0VWH9_CAVPO	Uncharacterized protein OS=Cavia porcellus GN=NUDT15 PE=4 SV=1	CAVPO	1
2924	1.39	1.61	8.229	4.613	2.369	tr|H0V8E2|H0V8E2_CAVPO	Uncharacterized protein OS=Cavia porcellus GN=Rasa4 PE=4 SV=1	CAVPO	2
2925	1.39	1.51	19	4.977	3.167	tr|H0VDV5|H0VDV5_CAVPO	Uncharacterized protein OS=Cavia porcellus GN=CCDC91 PE=4 SV=1	CAVPO	1
2926	1.39	1.53	5.176	5.176	1.294	tr|H0VHP8|H0VHP8_CAVPO	Uncharacterized protein OS=Cavia porcellus GN=RFT1 PE=4 SV=1	CAVPO	1

2927	1.39	1.47	12.7	10.05	10.05	tr|H0VUF0|H0VUF0_CAVPO	Uncharacterized protein OS=Cavia porcellus GN=TMEM205 PE=4 SV=1	CAVPO	1
2928	1.38	1.46	11.04	3.084	3.084	tr|H0UUP6|H0UUP6_CAVPO	Uncharacterized protein (Fragment) OS=Cavia porcellus GN=LRIT3 PE=4 SV=1	CAVPO	1
2929	1.38	1.53	9.645	6.937	3.892	tr|H0VD49|H0VD49_CAVPO	Granulins (Fragment) OS=Cavia porcellus GN=GRN PE=4 SV=1	CAVPO	2
2930	1.38	1.51	4.992	3.48	1.967	tr|H0VHS2|H0VHS2_CAVPO	Uncharacterized protein (Fragment) OS=Cavia porcellus GN=WDR48 PE=4 SV=1	CAVPO	1
2931	1.38	1.48	9.253	3.915	3.915	tr|H0W4R1|H0W4R1_CAVPO	Uncharacterized protein (Fragment) OS=Cavia porcellus GN=MTX3 PE=4 SV=1	CAVPO	1
2932	1.37	1.47	3.419	3.419	1.852	tr|H0UZ93|H0UZ93_CAVPO	Uncharacterized protein (Fragment) OS=Cavia porcellus GN=ATG7 PE=4 SV=1	CAVPO	1
2933	1.37	1.45	19.08	6.87	6.87	tr|H0V1U9|H0V1U9_CAVPO	Uncharacterized protein OS=Cavia porcellus GN=EMC10 PE=4 SV=1	CAVPO	1
2934	1.37	1.5	12.64	6.319	3.846	tr|H0V758|H0V758_CAVPO	Uncharacterized protein (Fragment) OS=Cavia porcellus GN=ADA PE=3 SV=1	CAVPO	1
2935	1.37	1.46	5.803	1.85	1.85	tr|H0VP36|H0VP36_CAVPO	Uncharacterized protein OS=Cavia porcellus GN=SLC24A1 PE=4 SV=1	CAVPO	2
2936	1.37	1.62	6.308	4.673	2.804	tr|H0VQN2|H0VQN2_CAVPO	Histone deacetylase OS=Cavia porcellus GN=HDAC3 PE=3 SV=1	CAVPO	1
2937	1.36	1.46	8.316	4.99	2.495	tr|H0VEG8|H0VEG8_CAVPO	Uncharacterized protein (Fragment) OS=Cavia porcellus GN=RCOR3 PE=4 SV=1	CAVPO	1
2938	1.36	1.5	12.48	3.248	3.248	tr|H0VGE4|H0VGE4_CAVPO	Uncharacterized protein OS=Cavia porcellus GN=SWAP70 PE=4 SV=1	CAVPO	2
2939	1.36	3.58	15.94	14.54	4.382	tr|H0VHE6|H0VHE6_CAVPO	Uncharacterized protein (Fragment) OS=Cavia porcellus GN=KPNA4 PE=4 SV=1	CAVPO	3
2940	1.35	1.49	2.414	1.892	1.892	tr|H0UX17|H0UX17_CAVPO	Uncharacterized protein (Fragment) OS=Cavia porcellus PE=4 SV=1	CAVPO	2

2941	1.35	1.46	4.284	1.463	1.463	tr|H0V0Q8|H0V0Q8_CAVPO	Uncharacterized protein (Fragment) OS=Cavia porcellus GN=LARP1 PE=4 SV=1	CAVPO	1
2942	1.35	1.71	3.565	0.3753	0.05447	tr|H0V1Y5|H0V1Y5_CAVPO	Uncharacterized protein (Fragment) OS=Cavia porcellus GN=Ttn PE=4 SV=1	CAVPO	1
2943	1.35	1.43	6.312	6.312	2.658	tr|H0VEC0|H0VEC0_CAVPO	Uncharacterized protein OS=Cavia porcellus GN=SLC25A20 PE=3 SV=1	CAVPO	1
2944	1.35	1.48	11.58	3.107	1.554	tr|H0VEF9|H0VEF9_CAVPO	Uncharacterized protein (Fragment) OS=Cavia porcellus GN=RTF1 PE=4 SV=1	CAVPO	2
2945	1.34	1.53	10.62	5.645	3.495	tr|H0UUL0|H0UUL0_CAVPO	Uncharacterized protein OS=Cavia porcellus GN=REPS1 PE=4 SV=1	CAVPO	2
2946	1.34	1.45	2.532	2.532	1.718	tr|H0UXX2|H0UXX2_CAVPO	Uncharacterized protein (Fragment) OS=Cavia porcellus GN=MAGI1 PE=4 SV=1	CAVPO	1
2947	1.33	1.41	6.993	6.993	6.993	tr|H0UTN2|H0UTN2_CAVPO	Uncharacterized protein OS=Cavia porcellus GN=CLDND2 PE=4 SV=1	CAVPO	1
2948	1.33	1.46	5.735	2.398	2.398	tr|H0VGZ0|H0VGZ0_CAVPO	Uncharacterized protein (Fragment) OS=Cavia porcellus GN=LIMCH1 PE=4 SV=1	CAVPO	2
2949	1.33	4.89	19.78	5.806	5.806	tr|H0VIA2|H0VIA2_CAVPO	Uncharacterized protein OS=Cavia porcellus GN=KRT7 PE=3 SV=1	CAVPO	3
2950	1.33	1.46	16.8	5.785	2.755	tr|H0VIM1|H0VIM1_CAVPO	Uncharacterized protein OS=Cavia porcellus GN=RFC4 PE=4 SV=1	CAVPO	1
2951	1.33	1.43	15.32	2.078	2.078	tr|H0VKW0|H0VKW0_CAVPO	Uncharacterized protein (Fragment) OS=Cavia porcellus GN=SPHK1 PE=4 SV=1	CAVPO	1
2952	1.33	5.56	12.1	5.832	5.832	tr|H0VP43|H0VP43_CAVPO	Uncharacterized protein (Fragment) OS=Cavia porcellus GN=MAPK10 PE=4 SV=1	CAVPO	3
2953	1.33	1.42	11.74	2.347	2.347	tr|H0VRT8|H0VRT8_CAVPO	Uncharacterized protein (Fragment) OS=Cavia porcellus GN=NEU1 PE=4 SV=1	CAVPO	1
2954	1.33	1.57	4.444	4.444	4.444	tr|H0VS85|H0VS85_CAVPO	Uncharacterized protein (Fragment) OS=Cavia porcellus GN=SMAP1 PE=4 SV=1	CAVPO	2

2955	1.33	14.7	35.9	27.99	13.59	tr|H0W4G1|H0W4G1_CAVPO	Uncharacterized protein (Fragment) OS=Cavia porcellus GN=AHCYL2 PE=3 SV=1	CAVPO	7
2956	1.32	1.48	4.455	3.517	1.29	tr|H0UYF2|H0UYF2_CAVPO	Uncharacterized protein (Fragment) OS=Cavia porcellus GN=COG5 PE=4 SV=1	CAVPO	1
2957	1.32	1.48	15.93	9.615	6.044	tr|H0V1C7|H0V1C7_CAVPO	Uncharacterized protein OS=Cavia porcellus GN=DRG2 PE=4 SV=1	CAVPO	2
2958	1.32	1.72	9.329	4.956	2.624	tr|H0VRL8|H0VRL8_CAVPO	Uncharacterized protein (Fragment) OS=Cavia porcellus GN=HOMER2 PE=4 SV=1	CAVPO	1
2959	1.32	1.42	11.52	4.783	1.957	tr|H0W1K3|H0W1K3_CAVPO	Annexin (Fragment) OS=Cavia porcellus GN=ANXA11 PE=3 SV=1	CAVPO	1
2960	1.31	1.4	2.647	1.418	1.418	tr|H0V032|H0V032_CAVPO	Uncharacterized protein OS=Cavia porcellus GN=MICAL1 PE=4 SV=1	CAVPO	1
2961	1.31	1.41	6.417	2.523	1.081	tr|H0VC13|H0VC13_CAVPO	Uncharacterized protein OS=Cavia porcellus GN=KIAA1107 PE=4 SV=1	CAVPO	1
2962	1.31	1.39	8.081	4.04	4.04	tr|H0VN24|H0VN24_CAVPO	Uncharacterized protein OS=Cavia porcellus GN=DSCR3 PE=4 SV=1	CAVPO	1
2963	1.29	1.45	9.383	6.42	4.691	tr|H0V616|H0V616_CAVPO	Uncharacterized protein OS=Cavia porcellus GN=RBM17 PE=4 SV=1	CAVPO	2
2964	1.29	1.46	14.49	12.37	5.3	tr|H0W3U1|H0W3U1_CAVPO	Uncharacterized protein (Fragment) OS=Cavia porcellus GN=PPAP2C PE=4 SV=1	CAVPO	1
2965	1.29	1.38	28.66	4.459	4.459	tr|H0W9S6|H0W9S6_CAVPO	Uncharacterized protein OS=Cavia porcellus GN=Nudt11 PE=3 SV=1	CAVPO	1
2966	1.28	1.37	1.939	1.939	1.939	tr|H0UX09|H0UX09_CAVPO	Uncharacterized protein (Fragment) OS=Cavia porcellus GN=AVL9 PE=4 SV=1	CAVPO	1
2967	1.28	1.36	7.568	7.568	7.568	tr|H0V6J9|H0V6J9_CAVPO	Uncharacterized protein OS=Cavia porcellus GN=SSR3 PE=4 SV=1	CAVPO	1
2968	1.28	1.37	4.743	4.743	4.743	tr|H0VI27|H0VI27_CAVPO	Uncharacterized protein (Fragment) OS=Cavia porcellus GN=CUTC PE=3 SV=1	CAVPO	1

2969	1.28	1.36	20.3	4.95	4.95	tr|H0VSK6|H0VSK6_CAVPO	Uncharacterized protein OS=Cavia porcellus GN=BCL7B PE=4 SV=1	CAVPO	1
2970	1.27	1.45	16.29	9.551	2.247	tr|H0UT47|H0UT47_CAVPO	Uncharacterized protein OS=Cavia porcellus GN=BAIAP2 PE=4 SV=1	CAVPO	1
2971	1.27	1.45	17.36	12.69	5.959	tr|H0UW30|H0UW30_CAVPO	Uncharacterized protein OS=Cavia porcellus GN=NAPEPLD PE=4 SV=1	CAVPO	2
2972	1.27	1.5	10.85	7.39	1.549	tr|H0V193|H0V193_CAVPO	Uncharacterized protein OS=Cavia porcellus GN=VPS16 PE=4 SV=1	CAVPO	1
2973	1.27	1.46	23.11	19.12	8.613	tr|H0VPA7|H0VPA7_CAVPO	Uncharacterized protein (Fragment) OS=Cavia porcellus GN=GPT2 PE=4 SV=1	CAVPO	2
2974	1.26	5.79	18.88	18.88	18.88	tr|H0VL19|H0VL19_CAVPO	Proteasome subunit alpha type OS=Cavia porcellus GN=PSMA8 PE=3 SV=1	CAVPO	4
2975	1.26	1.35	5.622	1.807	1.807	tr|H0W964|H0W964_CAVPO	Uncharacterized protein OS=Cavia porcellus GN=LOC100719074 PE=4 SV=1	CAVPO	1
2976	1.25	1.43	11.49	3.908	1.149	tr|H0UV24|H0UV24_CAVPO	Uncharacterized protein OS=Cavia porcellus GN=BBS9 PE=4 SV=1	CAVPO	1
2977	1.25	1.39	4.658	4.658	2.329	tr|H0V4R5|H0V4R5_CAVPO	Uncharacterized protein OS=Cavia porcellus GN=TBC1D17 PE=4 SV=1	CAVPO	1
2978	1.25	1.32	6.916	4.611	4.611	tr|H0VSA1|H0VSA1_CAVPO	Uncharacterized protein OS=Cavia porcellus GN=FAM118B PE=4 SV=1	CAVPO	1
2979	1.25	1.35	16.43	10.14	5.314	tr|H0W1B9|H0W1B9_CAVPO	Uncharacterized protein (Fragment) OS=Cavia porcellus GN=RPL7 PE=3 SV=1	CAVPO	1
2980	1.24	1.32	5.383	1.305	1.305	tr|D3YE96|D3YE96_CAVPO	Acetylcholinesterase OS=Cavia porcellus GN=AchE PE=2 SV=1	CAVPO	1
2981	1.24	1.32	8.257	8.257	8.257	tr|H0UUS7|H0UUS7_CAVPO	Transcription initiation factor IIA subunit 2 OS=Cavia porcellus GN=GTF2A2 PE=3 SV=1	CAVPO	1
2982	1.24	1.39	12.26	9.748	5.975	tr|H0UWY6|H0UWY6_CAVPO	Uncharacterized protein (Fragment) OS=Cavia porcellus GN=SLC25A1 PE=3 SV=1	CAVPO	2

2983	1.24	1.39	11.26	9.066	3.571	tr|H0VG28|H0VG28_CAVPO	Uncharacterized protein (Fragment) OS=Cavia porcellus GN=Cxadr PE=4 SV=1	CAVPO	1
2984	1.24	1.34	9.222	1.989	1.989	tr|H0VI15|H0VI15_CAVPO	Uncharacterized protein (Fragment) OS=Cavia porcellus GN=YTHDC1 PE=4 SV=1	CAVPO	1
2985	1.24	1.38	17.11	17.11	11.4	tr|H0VK21|H0VK21_CAVPO	Uncharacterized protein OS=Cavia porcellus GN=C2orf49 PE=4 SV=1	CAVPO	2
2986	1.24	1.39	16.51	16.51	5.66	tr|H0VKD3|H0VKD3_CAVPO	Uncharacterized protein OS=Cavia porcellus GN=PDCD10 PE=4 SV=1	CAVPO	1
2987	1.24	1.38	5	5	5	tr|H0VUD0|H0VUD0_CAVPO	Uncharacterized protein (Fragment) OS=Cavia porcellus GN=PIP4K2C PE=4 SV=1	CAVPO	2
2988	1.24	1.32	6.417	3.209	3.209	tr|H0VW65|H0VW65_CAVPO	Uncharacterized protein (Fragment) OS=Cavia porcellus GN=PTAR1 PE=4 SV=1	CAVPO	1
2989	1.24	1.62	12.99	11.02	1.772	tr|H0W0F6|H0W0F6_CAVPO	Uncharacterized protein OS=Cavia porcellus GN=ARFGAP3 PE=4 SV=1	CAVPO	1
2990	1.24	1.34	14.33	7.164	2.687	tr|H0W372|H0W372_CAVPO	Uncharacterized protein OS=Cavia porcellus GN=B3GAT3 PE=4 SV=1	CAVPO	1
2991	1.23	1.38	20.92	15.69	4.902	tr|H0UY57|H0UY57_CAVPO	Uncharacterized protein (Fragment) OS=Cavia porcellus GN=UFD1L PE=4 SV=1	CAVPO	1
2992	1.23	1.31	6.128	4.457	4.457	tr|H0V1M2|H0V1M2_CAVPO	Uncharacterized protein OS=Cavia porcellus GN=SLC35B1 PE=4 SV=1	CAVPO	1
2993	1.23	1.38	9.128	4.905	4.087	tr|H0V219|H0V219_CAVPO	Uncharacterized protein (Fragment) OS=Cavia porcellus GN=RPS6KA1 PE=4 SV=1	CAVPO	2
2994	1.23	1.32	3.538	0.2984	0.2984	tr|H0V6W7|H0V6W7_CAVPO	Uncharacterized protein OS=Cavia porcellus GN=ACACA PE=3 SV=1	CAVPO	1
2995	1.23	1.4	21.43	15.34	0	tr|H0V9U4|H0V9U4_CAVPO	Uncharacterized protein OS=Cavia porcellus GN=DPF3 PE=4 SV=1	CAVPO	0
2996	1.23	1.34	6.481	2.16	1.337	tr|H0VEN1|H0VEN1_CAVPO	Uncharacterized protein (Fragment) OS=Cavia porcellus GN=INTS3 PE=4 SV=1	CAVPO	1

2997	1.23	1.33	10.05	5.667	3.291	tr|H0VQA5|H0VQA5_CAVPO	Uncharacterized protein (Fragment) OS=Cavia porcellus GN=FOXK1 PE=4 SV=1	CAVPO	1
2998	1.23	3.47	35.61	34.09	18.94	tr|H0VWS6|H0VWS6_CAVPO	Uncharacterized protein (Fragment) OS=Cavia porcellus GN=CPLX1 PE=4 SV=1	CAVPO	2
2999	1.22	1.3	3.759	2.105	2.105	tr|H0UZ30|H0UZ30_CAVPO	Uncharacterized protein OS=Cavia porcellus GN=OS9 PE=4 SV=1	CAVPO	1
3000	1.22	1.31	7.572	1.336	1.336	tr|H0VPE2|H0VPE2_CAVPO	Uncharacterized protein OS=Cavia porcellus GN=Glra1 PE=3 SV=1	CAVPO	1
3001	1.22	1.38	19.49	13.1	3.514	tr|H0VQY8|H0VQY8_CAVPO	Uncharacterized protein OS=Cavia porcellus GN=Rmdn1 PE=4 SV=1	CAVPO	1
3002	1.22	1.44	10.8	8.483	2.571	tr|H0W0S2|H0W0S2_CAVPO	Uncharacterized protein OS=Cavia porcellus GN=CBX8 PE=4 SV=1	CAVPO	1
3003	1.21	1.36	13.79	10.68	3.107	tr|H0UUH7|H0UUH7_CAVPO	Uncharacterized protein OS=Cavia porcellus GN=TTC8 PE=4 SV=1	CAVPO	1
3004	1.21	1.34	6.649	3.089	1.813	tr|H0UWC2|H0UWC2_CAVPO	Uncharacterized protein OS=Cavia porcellus GN=GOLGA3 PE=4 SV=1	CAVPO	2
3005	1.21	1.35	3.532	3.532	3.532	tr|H0V0H9|H0V0H9_CAVPO	Uncharacterized protein (Fragment) OS=Cavia porcellus GN=WASL PE=4 SV=1	CAVPO	2
3006	1.21	1.42	15.63	8.807	3.409	tr|H0VQ62|H0VQ62_CAVPO	Uncharacterized protein (Fragment) OS=Cavia porcellus GN=GPD1L PE=3 SV=1	CAVPO	1
3007	1.21	9.71	22.55	13.86	8.872	tr|H0VTP1|H0VTP1_CAVPO	Uncharacterized protein OS=Cavia porcellus GN=YES1 PE=4 SV=1	CAVPO	5
3008	1.21	1.35	15.75	5.503	3.416	tr|H0VYA5|H0VYA5_CAVPO	Uncharacterized protein (Fragment) OS=Cavia porcellus GN=PVRL2 PE=4 SV=1	CAVPO	2
3009	1.21	1.3	2.401	0.8212	0.8212	tr|H0WBX5|H0WBX5_CAVPO	Uncharacterized protein (Fragment) OS=Cavia porcellus GN=CAMSAP1 PE=4 SV=1	CAVPO	1
3010	1.2	3.51	36.02	18.96	10.9	tr|H0V1C5|H0V1C5_CAVPO	Synaptosomal-associated protein OS=Cavia porcellus GN=SNAP23 PE=3 SV=1	CAVPO	3

3011	1.2	1.35	1.721	0.6287	0.2978	tr|H0V1Z4|H0V1Z4_CAVPO	Uncharacterized protein OS=Cavia porcellus GN=FRY PE=4 SV=1	CAVPO	1
3012	1.2	1.35	7.523	2.808	1.103	tr|H0V3W2|H0V3W2_CAVPO	Uncharacterized protein OS=Cavia porcellus GN=SEC23IP PE=4 SV=1	CAVPO	1
3013	1.2	1.28	2.679	2.679	2.679	tr|H0V6T7|H0V6T7_CAVPO	Uncharacterized protein OS=Cavia porcellus GN=GPR107 PE=4 SV=1	CAVPO	1
3014	1.19	1.31	7.707	4.624	1.927	tr|H0VHA3|H0VHA3_CAVPO	Uncharacterized protein (Fragment) OS=Cavia porcellus GN=RORB PE=3 SV=1	CAVPO	1
3015	1.19	1.28	1.267	1.267	1.267	tr|H0VXF5|H0VXF5_CAVPO	Uncharacterized protein (Fragment) OS=Cavia porcellus GN=SORCS2 PE=4 SV=1	CAVPO	1
3016	1.19	9.6	60.53	57.89	57.89	tr|H0WAD6|H0WAD6_CAVPO	Uncharacterized protein OS=Cavia porcellus PE=4 SV=1	CAVPO	8
3017	1.18	1.3	10.55	3.247	1.623	tr|H0UT14|H0UT14_CAVPO	Uncharacterized protein OS=Cavia porcellus GN=RPA1 PE=4 SV=1	CAVPO	1
3018	1.18	1.29	9.615	6.731	2.5	tr|H0VMN2|H0VMN2_CAVPO	Succinyl-CoA:3-ketoacid-coenzyme A transferase OS=Cavia porcellus GN=OXCT1 PE=3 SV=1	CAVPO	1
3019	1.17	1.25	7.895	6.842	6.842	tr|H0VTM3|H0VTM3_CAVPO	Uncharacterized protein OS=Cavia porcellus GN=TMEM9 PE=4 SV=1	CAVPO	1
3020	1.17	1.27	4.966	4.966	2.935	tr|H0VVA9|H0VVA9_CAVPO	Uncharacterized protein OS=Cavia porcellus GN=NDUFAF7 PE=4 SV=1	CAVPO	1
3021	1.16	1.33	28.9	18.81	0	tr|H0V5B4|H0V5B4_CAVPO	Uncharacterized protein OS=Cavia porcellus GN=MRPS34 PE=4 SV=1	CAVPO	0
3022	1.16	1.3	19.73	11.66	11.66	tr|H0VY33|H0VY33_CAVPO	Uncharacterized protein OS=Cavia porcellus GN=CCDC124 PE=4 SV=1	CAVPO	2
3023	1.16	1.25	6.97	2.727	2.727	tr|H0VZ46|H0VZ46_CAVPO	Uncharacterized protein (Fragment) OS=Cavia porcellus GN=OGFRL1 PE=4 SV=1	CAVPO	1
3024	1.15	1.3	8.075	8.075	5.59	tr|H0V5X2|H0V5X2_CAVPO	Uncharacterized protein OS=Cavia porcellus	CAVPO	2

							GN=CCDC47 PE=4 SV=1		
3025	1.14	1.27	1.951	1.951	1.951	tr|H0UU23|H0UU23_CAVPO	Uncharacterized protein OS=Cavia porcellus GN=NCOA2 PE=4 SV=1	CAVPO	2
3026	1.14	1.26	7.25	3.107	1.036	tr|H0UX95|H0UX95_CAVPO	Uncharacterized protein OS=Cavia porcellus GN=PRPF40B PE=4 SV=1	CAVPO	1
3027	1.14	3.36	17.87	10.97	10.97	tr|H0UZ46|H0UZ46_CAVPO	Uncharacterized protein (Fragment) OS=Cavia porcellus GN=RDH11 PE=3 SV=1	CAVPO	3
3028	1.14	1.23	9.465	4.733	3.498	tr|H0V7L9|H0V7L9_CAVPO	Uncharacterized protein (Fragment) OS=Cavia porcellus GN=FRMD5 PE=4 SV=1	CAVPO	1
3029	1.14	1.28	14.62	11.92	11.92	tr|H0V7S7|H0V7S7_CAVPO	Uncharacterized protein (Fragment) OS=Cavia porcellus GN=BCCIP PE=4 SV=1	CAVPO	2
3030	1.14	1.24	6.286	1.714	1.714	tr|H0VD64|H0VD64_CAVPO	Uncharacterized protein OS=Cavia porcellus GN=UPF2 PE=4 SV=1	CAVPO	1
3031	1.14	1.25	18.37	1.895	1.895	tr|H0VKE2|H0VKE2_CAVPO	Uncharacterized protein OS=Cavia porcellus GN=RHPN2 PE=4 SV=1	CAVPO	1
3032	1.14	1.26	8.257	1.009	1.009	tr|H0VNE9|H0VNE9_CAVPO	Uncharacterized protein OS=Cavia porcellus GN=TMF1 PE=4 SV=1	CAVPO	1
3033	1.14	1.34	44.3	29.11	4.43	tr|H0VSN8|H0VSN8_CAVPO	Uncharacterized protein OS=Cavia porcellus GN=RPS11 PE=3 SV=1	CAVPO	1
3034	1.14	1.23	33.33	14.71	14.71	tr|H0W9L6|H0W9L6_CAVPO	Uncharacterized protein OS=Cavia porcellus GN=SPCS1 PE=4 SV=1	CAVPO	1
3035	1.14	1.26	12.76	12.76	4.138	tr|H0WD55|H0WD55_CAVPO	Uncharacterized protein OS=Cavia porcellus GN=PIH1D1 PE=4 SV=1	CAVPO	1
3036	1.13	1.27	15.7	7.623	4.933	tr|H0UYL9|H0UYL9_CAVPO	Uncharacterized protein OS=Cavia porcellus GN=CHMP7 PE=4 SV=1	CAVPO	2
3037	1.13	1.21	14.09	5.705	5.705	tr|H0V3R3|H0V3R3_CAVPO	Uncharacterized protein OS=Cavia porcellus GN=JAM2 PE=4 SV=1	CAVPO	1

3038	1.13	2.63	19.51	10.37	7.407	tr|H0V658|H0V658_CAVPO	Uncharacterized protein (Fragment) OS=Cavia porcellus GN=GABRB3 PE=3 SV=1	CAVPO	2
3039	1.13	1.21	4.62	4.62	4.62	tr|H0V6P3|H0V6P3_CAVPO	Uncharacterized protein OS=Cavia porcellus GN=TEX264 PE=4 SV=1	CAVPO	1
3040	1.12	1.29	12.33	8.969	2.242	tr|H0UTP1|H0UTP1_CAVPO	Uncharacterized protein OS=Cavia porcellus GN=Bckdha PE=4 SV=1	CAVPO	1
3041	1.12	1.29	9.114	6.329	2.658	tr|H0UY51|H0UY51_CAVPO	Uncharacterized protein OS=Cavia porcellus GN=ESPN PE=4 SV=1	CAVPO	2
3042	1.12	1.21	2.455	2.455	2.455	tr|H0VLS5|H0VLS5_CAVPO	Uncharacterized protein OS=Cavia porcellus GN=BAG4 PE=4 SV=1	CAVPO	1
3043	1.12	2.82	8.673	7.143	7.143	tr|H0VQZ9|H0VQZ9_CAVPO	Uncharacterized protein OS=Cavia porcellus GN=TSC22D4 PE=4 SV=1	CAVPO	3
3044	1.12	1.3	11.22	5.179	2.343	tr|H0VTG6|H0VTG6_CAVPO	Uncharacterized protein OS=Cavia porcellus PE=4 SV=1	CAVPO	2
3045	1.11	1.21	3.733	0.5867	0.5867	tr|H0UUN0|H0UUN0_CAVPO	Uncharacterized protein OS=Cavia porcellus GN=NAV1 PE=4 SV=1	CAVPO	1
3046	1.11	1.19	3.084	3.084	3.084	tr|H0V967|H0V967_CAVPO	Uncharacterized protein (Fragment) OS=Cavia porcellus GN=ITM2B PE=4 SV=1	CAVPO	1
3047	1.11	1.2	5.794	5.794	4.486	tr|H0VQ52|H0VQ52_CAVPO	Uncharacterized protein OS=Cavia porcellus GN=SLC7A8 PE=4 SV=1	CAVPO	1
3048	1.11	1.19	7.023	5.351	5.351	tr|H0W699|H0W699_CAVPO	Uncharacterized protein OS=Cavia porcellus GN=LOC100714430 PE=4 SV=1	CAVPO	1
3049	1.1	1.21	6.785	3.716	1.777	tr|H0UTJ3|H0UTJ3_CAVPO	Uncharacterized protein OS=Cavia porcellus GN=SLC27A6 PE=4 SV=1	CAVPO	1
3050	1.1	1.26	24.07	14.11	5.394	tr|H0UWW6|H0UWW6_CAVPO	Uncharacterized protein (Fragment) OS=Cavia porcellus GN=PDCL3 PE=4 SV=1	CAVPO	1
3051	1.1	1.2	7.709	1.713	1.713	tr|H0V8T7|H0V8T7_CAVPO	Uncharacterized protein (Fragment) OS=Cavia porcellus GN=INPP5K PE=4 SV=1	CAVPO	2

3052	1.1	1.2	14.59	10.53	5.024	tr|H0VB56|H0VB56_CAVPO	Uncharacterized protein OS=Cavia porcellus GN=TRAPPC13 PE=4 SV=1	CAVPO	1
3053	1.1	1.22	17.58	11.36	5.495	tr|H0VCE0|H0VCE0_CAVPO	Uncharacterized protein OS=Cavia porcellus GN=PAQR4 PE=4 SV=1	CAVPO	1
3054	1.1	1.18	7.171	4.382	4.382	tr|H0VK66|H0VK66_CAVPO	Uncharacterized protein OS=Cavia porcellus GN=DERL1 PE=4 SV=1	CAVPO	1
3055	1.1	1.2	6.773	1.338	1.338	tr|H0VMM8|H0VMM8_CAVPO	Uncharacterized protein OS=Cavia porcellus GN=Wapal PE=4 SV=1	CAVPO	1
3056	1.09	1.4	15.19	9.409	1.344	tr|H0UVA5|H0UVA5_CAVPO	Uncharacterized protein OS=Cavia porcellus GN=TRIM3 PE=4 SV=1	CAVPO	1
3057	1.09	1.34	8.278	5.474	2.136	tr|H0VNT2|H0VNT2_CAVPO	Uncharacterized protein OS=Cavia porcellus GN=EML2 PE=4 SV=1	CAVPO	2
3058	1.09	5.27	7.494	7.494	7.494	tr|H0VTN4|H0VTN4_CAVPO	Uncharacterized protein OS=Cavia porcellus GN=MAPK8 PE=4 SV=1	CAVPO	3
3059	1.09	1.18	13.21	8.491	8.491	tr|H0VZ09|H0VZ09_CAVPO	Uncharacterized protein (Fragment) OS=Cavia porcellus GN=GATC PE=3 SV=1	CAVPO	1
3060	1.08	1.17	4.102	2.405	1.556	tr|H0V5I0|H0V5I0_CAVPO	Uncharacterized protein (Fragment) OS=Cavia porcellus GN=Bop1 PE=3 SV=1	CAVPO	1
3061	1.08	1.17	4.215	1.903	1.292	tr|H0V912|H0V912_CAVPO	Uncharacterized protein OS=Cavia porcellus GN=MAGI3 PE=4 SV=1	CAVPO	1
3062	1.08	1.17	5.252	1.969	1.969	tr|H0VGI6|H0VGI6_CAVPO	Uncharacterized protein (Fragment) OS=Cavia porcellus GN=ASAP2 PE=4 SV=1	CAVPO	1
3063	1.08	1.16	1.998	0.8226	0.8226	tr|H0VU90|H0VU90_CAVPO	Uncharacterized protein OS=Cavia porcellus GN=SETD1A PE=4 SV=1	CAVPO	1
3064	1.07	1.16	5.314	2.415	2.415	tr|H0V585|H0V585_CAVPO	Uncharacterized protein OS=Cavia porcellus GN=Fam175b PE=4 SV=1	CAVPO	1
3065	1.07	1.24	24.9	16.33	5.306	tr|H0VGR7|H0VGR7_CAVPO	Uncharacterized protein OS=Cavia porcellus GN=BDH2 PE=4 SV=1	CAVPO	1

3066	1.07	1.16	8.091	5.825	5.825	tr|H0W187|H0W187_CAVPO	Uncharacterized protein (Fragment) OS=Cavia porcellus GN=NUBP1 PE=3 SV=1	CAVPO	1
3067	1.07	1.16	11.2	5.6	2.4	tr|H0W655|H0W655_CAVPO	Uncharacterized protein (Fragment) OS=Cavia porcellus GN=RGS20 PE=4 SV=1	CAVPO	1
3068	1.06	1.21	7.214	4.353	1.493	tr|H0UZI3|H0UZI3_CAVPO	Uncharacterized protein OS=Cavia porcellus GN=ALDH16A1 PE=4 SV=1	CAVPO	1
3069	1.06	3.55	26.87	23.79	18.06	tr|H0V6W6|H0V6W6_CAVPO	Uncharacterized protein (Fragment) OS=Cavia porcellus GN=VAPA PE=4 SV=1	CAVPO	3
3070	1.06	1.14	3.704	1.961	1.961	tr|H0VA57|H0VA57_CAVPO	Uncharacterized protein (Fragment) OS=Cavia porcellus GN=B3GALTL PE=4 SV=1	CAVPO	1
3071	1.06	1.16	7.307	5.846	3.34	tr|H0VCK7|H0VCK7_CAVPO	Uncharacterized protein OS=Cavia porcellus GN=DNAJA3 PE=3 SV=1	CAVPO	1
3072	1.06	1.18	8.108	8.108	4.955	tr|H0VVQ3|H0VVQ3_CAVPO	Uncharacterized protein OS=Cavia porcellus GN=CDC34 PE=3 SV=1	CAVPO	1
3073	1.05	1.14	6.033	1.463	1.463	tr|H0V3G8|H0V3G8_CAVPO	Uncharacterized protein (Fragment) OS=Cavia porcellus GN=FAM134A PE=4 SV=1	CAVPO	1
3074	1.05	4.35	10.3	8.108	8.108	tr|H0VGM8|H0VGM8_CAVPO	Uncharacterized protein OS=Cavia porcellus GN=GATAD2B PE=4 SV=1	CAVPO	4
3075	1.05	1.13	5.419	3.448	3.448	tr|H0VRN1|H0VRN1_CAVPO	Uncharacterized protein OS=Cavia porcellus GN=TM7SF2 PE=4 SV=1	CAVPO	1
3076	1.05	1.13	2.621	2.621	2.621	tr|H0W1M5|H0W1M5_CAVPO	Uncharacterized protein OS=Cavia porcellus GN=Xiap PE=4 SV=1	CAVPO	1
3077	1.05	1.18	22.66	22.66	8.594	tr|H0WBG9|H0WBG9_CAVPO	Uncharacterized protein (Fragment) OS=Cavia porcellus GN=FAM195B PE=4 SV=1	CAVPO	1
3078	1.05	1.13	3.774	1.887	1.887	tr|H0WBJ0|H0WBJ0_CAVPO	Uncharacterized protein OS=Cavia porcellus GN=LRRC2 PE=4 SV=1	CAVPO	1
3079	1.04	3.25	5.386	2.814	1.447	tr|H0UTG3|H0UTG3_CAVPO	Uncharacterized protein OS=Cavia porcellus GN=DHX8 PE=4 SV=1	CAVPO	2

3080	1.04	1.12	7.813	4.297	4.297	tr|H0VE50|H0VE50_CAVPO	Uncharacterized protein OS=Cavia porcellus GN=SYPL1 PE=4 SV=1	CAVPO	1
3081	1.04	1.13	12.83	4.934	4.934	tr|H0VH11|H0VH11_CAVPO	Uncharacterized protein OS=Cavia porcellus GN=GULP1 PE=4 SV=1	CAVPO	1
3082	1.04	1.16	8.084	8.084	3.892	tr|H0VM28|H0VM28_CAVPO	Uncharacterized protein OS=Cavia porcellus GN=WDR54 PE=4 SV=1	CAVPO	1
3083	1.03	1.18	6.872	2.974	1.231	tr|H0UWG9|H0UWG9_CAVPO	Uncharacterized protein OS=Cavia porcellus GN=BICD1 PE=4 SV=1	CAVPO	1
3084	1.02	9.08	38.46	38.46	25	tr|H0UWS5|H0UWS5_CAVPO	Uncharacterized protein OS=Cavia porcellus GN=RAB6B PE=3 SV=1	CAVPO	7
3085	1.02	1.15	10.59	10.59	10.59	tr|H0UYQ1|H0UYQ1_CAVPO	Uncharacterized protein OS=Cavia porcellus GN=RAB23 PE=3 SV=1	CAVPO	2
3086	1.02	1.1	2.125	1.417	1.417	tr|H0V743|H0V743_CAVPO	Uncharacterized protein (Fragment) OS=Cavia porcellus GN=VPS41 PE=4 SV=1	CAVPO	1
3087	1.02	1.11	8.408	3.754	2.102	tr|H0W330|H0W330_CAVPO	Uncharacterized protein (Fragment) OS=Cavia porcellus GN=MTSS1L PE=4 SV=1	CAVPO	2
3088	1.01	1.14	13.06	11.34	3.78	tr|H0UX94|H0UX94_CAVPO	Uncharacterized protein (Fragment) OS=Cavia porcellus GN=BRCC3 PE=4 SV=1	CAVPO	1
3089	1.01	1.09	4.455	1.382	1.382	tr|H0V3Q5|H0V3Q5_CAVPO	Uncharacterized protein OS=Cavia porcellus GN=GUSB PE=4 SV=1	CAVPO	1
3090	1.01	1.1	11.85	4.502	3.081	tr|H0VNJ6|H0VNJ6_CAVPO	Uncharacterized protein (Fragment) OS=Cavia porcellus GN=ACP2 PE=4 SV=1	CAVPO	1
3091	1.01	1.1	6.921	3.103	3.103	tr|H0VVA8|H0VVA8_CAVPO	Uncharacterized protein OS=Cavia porcellus GN=FAH PE=4 SV=1	CAVPO	1
3092	1.01	1.13	12.22	7.386	3.977	tr|H0W0V2|H0W0V2_CAVPO	Uncharacterized protein (Fragment) OS=Cavia porcellus GN=RFC5 PE=4 SV=1	CAVPO	1
3093	1	1.11	35.92	24.65	10.56	tr|H0UYV9|H0UYV9_CAVPO	Uncharacterized protein OS=Cavia porcellus GN=POLR2D PE=4 SV=1	CAVPO	1

3094	1	1.2	9.515	5.519	1.427	tr|H0VMR6|H0VMR6_CAVPO	Uncharacterized protein (Fragment) OS=Cavia porcellus GN=STXBP5 PE=4 SV=1	CAVPO	1
3095	1	1.08	3.377	1.028	1.028	tr|H0VPX5|H0VPX5_CAVPO	Uncharacterized protein (Fragment) OS=Cavia porcellus GN=GFPT2 PE=4 SV=1	CAVPO	1
3096	1	1.11	29.03	28.23	14.52	tr|H0VVR8|H0VVR8_CAVPO	Uncharacterized protein OS=Cavia porcellus GN=TAX1BP3 PE=4 SV=1	CAVPO	1
3097	0.99	1.1	3.557	0.8893	0.8893	tr|H0UY81|H0UY81_CAVPO	Uncharacterized protein OS=Cavia porcellus GN=Man2b1 PE=4 SV=1	CAVPO	1
3098	0.99	1.11	7.67	2.136	1.068	tr|H0V275|H0V275_CAVPO	Protein phosphatase 1 regulatory subunit 12 OS=Cavia porcellus GN=PPP1R12A PE=3 SV=1	CAVPO	1
3099	0.99	1.12	3.517	2.164	2.164	tr|H0VBN6|H0VBN6_CAVPO	Uncharacterized protein (Fragment) OS=Cavia porcellus GN=RBM6 PE=4 SV=1	CAVPO	2
3100	0.99	1.11	6.529	6.529	3.78	tr|H0VS01|H0VS01_CAVPO	Uncharacterized protein OS=Cavia porcellus GN=SSR1 PE=4 SV=1	CAVPO	1
3101	0.99	1.13	11.51	2.188	0.8914	tr|H0W331|H0W331_CAVPO	Uncharacterized protein OS=Cavia porcellus GN=CGNL1 PE=4 SV=1	CAVPO	1
3102	0.98	1.06	1.391	1.391	1.391	tr|H0UW95|H0UW95_CAVPO	Uncharacterized protein OS=Cavia porcellus GN=EBF2 PE=3 SV=1	CAVPO	1
3103	0.98	3.46	21.12	12.81	4.719	tr|H0V9T2|H0V9T2_CAVPO	Uncharacterized protein (Fragment) OS=Cavia porcellus GN=RGS6 PE=4 SV=1	CAVPO	2
3104	0.98	1.13	12.8	6.581	2.011	tr|H0VH88|H0VH88_CAVPO	Uncharacterized protein (Fragment) OS=Cavia porcellus GN=NSRP1 PE=4 SV=1	CAVPO	1
3105	0.98	1.08	3.346	1.983	0.9913	tr|H0VNV8|H0VNV8_CAVPO	Uncharacterized protein OS=Cavia porcellus GN=SLC39A10 PE=4 SV=1	CAVPO	1
3106	0.98	1.07	29.63	29.63	12.35	tr|H0W5Y8|H0W5Y8_CAVPO	Uncharacterized protein OS=Cavia porcellus GN=CTNNBIP1 PE=4 SV=1	CAVPO	1
3107	0.97	1.05	1.831	1.831	1.831	tr|H0UUW5|H0UUW5_CAVPO	Uncharacterized protein OS=Cavia porcellus GN=GXYLT1 PE=4 SV=1	CAVPO	1

3108	0.97	1.08	5.028	3.352	2.235	tr|H0V2K9|H0V2K9_CAVPO	Uncharacterized protein OS=Cavia porcellus GN=ACSS2 PE=4 SV=1	CAVPO	1
3109	0.97	1.07	22.4	15.3	8.743	tr|H0V539|H0V539_CAVPO	Uncharacterized protein OS=Cavia porcellus GN=EMC4 PE=4 SV=1	CAVPO	1
3110	0.97	1.07	17.86	10	6.429	tr|H0V5W7|H0V5W7_CAVPO	Uncharacterized protein OS=Cavia porcellus GN=C14orf1 PE=4 SV=1	CAVPO	1
3111	0.97	2.19	14.02	5.793	2.134	tr|H0VDG0|H0VDG0_CAVPO	Uncharacterized protein (Fragment) OS=Cavia porcellus GN=SRGAP2 PE=4 SV=1	CAVPO	2
3112	0.97	1.05	3.834	3.834	3.834	tr|H0W9N9|H0W9N9_CAVPO	Uncharacterized protein OS=Cavia porcellus GN=PTGR1 PE=4 SV=1	CAVPO	1
3113	0.96	1.04	2.55	2.55	2.55	tr|H0VFV0|H0VFV0_CAVPO	Uncharacterized protein (Fragment) OS=Cavia porcellus GN=CASC4 PE=4 SV=1	CAVPO	1
3114	0.96	1.04	9.691	2.68	2.68	tr|H0VN00|H0VN00_CAVPO	Uncharacterized protein OS=Cavia porcellus GN=NLE1 PE=4 SV=1	CAVPO	1
3115	0.96	1.07	10.8	6.62	3.833	tr|H0VP55|H0VP55_CAVPO	Uncharacterized protein OS=Cavia porcellus GN=SLC25A10 PE=3 SV=1	CAVPO	1
3116	0.96	1.06	4.857	1.743	1.743	tr|H0W6Y4|H0W6Y4_CAVPO	Uncharacterized protein OS=Cavia porcellus GN=PLA2G6 PE=4 SV=1	CAVPO	1
3117	0.95	1.03	5.825	1.699	1.699	tr|H0UXC9|H0UXC9_CAVPO	Uncharacterized protein OS=Cavia porcellus GN=BCAS3 PE=4 SV=1	CAVPO	1
3118	0.95	1.08	8.083	5.774	3.233	tr|H0V1F1|H0V1F1_CAVPO	Uncharacterized protein OS=Cavia porcellus GN=MFGE8 PE=4 SV=1	CAVPO	1
3119	0.95	1.14	13.97	3.752	1.423	tr|H0VAW5|H0VAW5_CAVPO	Uncharacterized protein (Fragment) OS=Cavia porcellus GN=AMPD3 PE=4 SV=1	CAVPO	1
3120	0.95	1.12	5.316	2.409	1.08	tr|H0VIK5|H0VIK5_CAVPO	Uncharacterized protein (Fragment) OS=Cavia porcellus GN=ATP13A1 PE=3 SV=1	CAVPO	1
3121	0.95	2.07	11.07	4.024	4.024	tr|H0VZM6|H0VZM6_CAVPO	Uncharacterized protein OS=Cavia porcellus GN=PPP2R5B PE=4 SV=1	CAVPO	2

3122	0.95	1.1	27.11	15.49	4.577	tr|H0W7L5|H0W7L5_CAVPO	Uncharacterized protein (Fragment) OS=Cavia porcellus GN=NEK7 PE=3 SV=1	CAVPO	1
3123	0.94	1.04	7.46	3.968	2.063	tr|H0V0A8|H0V0A8_CAVPO	Uncharacterized protein OS=Cavia porcellus GN=PLS1 PE=4 SV=1	CAVPO	1
3124	0.94	1.09	13.77	6.587	1.996	tr|H0V2Q3|H0V2Q3_CAVPO	Uncharacterized protein OS=Cavia porcellus GN=TRAF2 PE=4 SV=1	CAVPO	1
3125	0.94	1.01	2.672	2.672	2.672	tr|H0V961|H0V961_CAVPO	Alkaline phosphatase OS=Cavia porcellus GN=ALPL PE=3 SV=1	CAVPO	1
3126	0.94	1.01	6.202	6.202	6.202	tr|H0VGV4|H0VGV4_CAVPO	Uncharacterized protein OS=Cavia porcellus GN=CHRAC1 PE=4 SV=1	CAVPO	1
3127	0.94	1.02	8.723	2.492	2.492	tr|H0W353|H0W353_CAVPO	Gap junction protein OS=Cavia porcellus GN=GJD2 PE=3 SV=1	CAVPO	1
3128	0.93	1	5.423	1.952	1.952	tr|H0UVL0|H0UVL0_CAVPO	Uncharacterized protein (Fragment) OS=Cavia porcellus GN=SHE PE=4 SV=1	CAVPO	1
3129	0.93	1.05	11.74	4.027	2.181	tr|H0V4T2|H0V4T2_CAVPO	Uncharacterized protein (Fragment) OS=Cavia porcellus GN=FNBP1L PE=4 SV=1	CAVPO	1
3130	0.93	1.13	2.527	0.9232	0.9232	tr|H0V905|H0V905_CAVPO	Uncharacterized protein OS=Cavia porcellus GN=ATG2B PE=4 SV=1	CAVPO	2
3131	0.93	1.01	3.831	1.057	1.057	tr|H0VM06|H0VM06_CAVPO	Uncharacterized protein (Fragment) OS=Cavia porcellus GN=TRPV2 PE=4 SV=1	CAVPO	1
3132	0.93	1.06	7.407	1.955	0.823	tr|H0VNJ7|H0VNJ7_CAVPO	Uncharacterized protein OS=Cavia porcellus GN=NAA25 PE=4 SV=1	CAVPO	1
3133	0.93	2.18	6.75	5.25	5.25	tr|H0W0W4|H0W0W4_CAVPO	Uncharacterized protein (Fragment) OS=Cavia porcellus GN=ECSIT PE=4 SV=1	CAVPO	2
3134	0.92	1.01	6.954	1.656	1.656	tr|H0V3A0|H0V3A0_CAVPO	Uncharacterized protein OS=Cavia porcellus GN=ERCC4 PE=4 SV=1	CAVPO	1
3135	0.92	1.11	16.1	13.37	0	tr|H0VA08|H0VA08_CAVPO	Uncharacterized protein OS=Cavia porcellus GN=SLC27A2 PE=4 SV=1	CAVPO	0

3136	0.91	1.05	6.009	3.147	2.146	tr|H0V307|H0V307_CAVPO	Uncharacterized protein OS=Cavia porcellus GN=METTL13 PE=4 SV=1	CAVPO	1
3137	0.91	1.03	7.269	3.438	1.572	tr|H0V9G0|H0V9G0_CAVPO	Uncharacterized protein (Fragment) OS=Cavia porcellus GN=HIP1R PE=4 SV=1	CAVPO	1
3138	0.91	1	16.14	6.316	3.86	tr|H0VSD0|H0VSD0_CAVPO	Uncharacterized protein OS=Cavia porcellus GN=VPS37B PE=4 SV=1	CAVPO	1
3139	0.91	0.99	1.596	1.596	1.596	tr|H0W3H0|H0W3H0_CAVPO	Uncharacterized protein (Fragment) OS=Cavia porcellus GN=MKL1 PE=4 SV=1	CAVPO	1
3140	0.9	0.98	1.499	1.499	1.499	tr|H0UVJ1|H0UVJ1_CAVPO	Uncharacterized protein (Fragment) OS=Cavia porcellus GN=AFG3L2 PE=3 SV=1	CAVPO	1
3141	0.9	1	12.16	5.707	3.226	tr|H0V2P0|H0V2P0_CAVPO	Uncharacterized protein OS=Cavia porcellus GN=JMJD6 PE=4 SV=1	CAVPO	1
3142	0.9	0.98	3.401	1.511	0.8816	tr|H0VCY3|H0VCY3_CAVPO	Uncharacterized protein (Fragment) OS=Cavia porcellus PE=4 SV=1	CAVPO	1
3143	0.89	3.45	28.3	12.71	7.674	tr|H0V1H2|H0V1H2_CAVPO	Uncharacterized protein OS=Cavia porcellus GN=ACTR3B PE=3 SV=1	CAVPO	3
3144	0.89	1.02	15.79	15.79	8.772	tr|H0V4D1|H0V4D1_CAVPO	Uncharacterized protein OS=Cavia porcellus GN=CNOT7 PE=4 SV=1	CAVPO	2
3145	0.89	0.99	2.752	0.8891	0.6351	tr|H0V5G4|H0V5G4_CAVPO	Uncharacterized protein (Fragment) OS=Cavia porcellus GN=EP300 PE=4 SV=1	CAVPO	2
3146	0.89	0.98	8.587	1.789	1.789	tr|H0V5H1|H0V5H1_CAVPO	Uncharacterized protein (Fragment) OS=Cavia porcellus GN=PRKAA1 PE=4 SV=1	CAVPO	1
3147	0.89	0.97	16.74	11.63	11.63	tr|H0V7B4|H0V7B4_CAVPO	Uncharacterized protein OS=Cavia porcellus GN=GTF3C6 PE=4 SV=1	CAVPO	2
3148	0.89	1	17.91	3.582	3.582	tr|H0V8T6|H0V8T6_CAVPO	Uncharacterized protein OS=Cavia porcellus GN=TAF7 PE=4 SV=1	CAVPO	1
3149	0.89	0.97	11.17	5.825	5.825	tr|H0VJN0|H0VJN0_CAVPO	Sulfhydryl oxidase OS=Cavia porcellus GN=GFER PE=3 SV=1	CAVPO	1

3150	0.89	0.97	9.05	9.05	9.05	tr|H0VV64|H0VV64_CAVPO	Uncharacterized protein OS=Cavia porcellus GN=SDF2L1 PE=4 SV=1	CAVPO	1
3151	0.89	1.03	8.639	1.538	1.538	tr|H0W355|H0W355_CAVPO	Uncharacterized protein OS=Cavia porcellus GN=SLMAP PE=4 SV=1	CAVPO	1
3152	0.88	0.98	3.719	2.273	2.273	tr|H0UVK1|H0UVK1_CAVPO	Uncharacterized protein (Fragment) OS=Cavia porcellus GN=FAF1 PE=4 SV=1	CAVPO	1
3153	0.88	0.96	1.852	1.852	1.852	tr|H0UYU9|H0UYU9_CAVPO	Uncharacterized protein OS=Cavia porcellus GN=ARL13B PE=4 SV=1	CAVPO	1
3154	0.88	1.2	20.5	20.5	8.368	tr|H0UYW7|H0UYW7_CAVPO	Uncharacterized protein (Fragment) OS=Cavia porcellus GN=IDI1 PE=4 SV=1	CAVPO	1
3155	0.88	0.97	5.411	3.247	3.247	tr|H0V9A9|H0V9A9_CAVPO	Uncharacterized protein (Fragment) OS=Cavia porcellus GN=FSD1L PE=4 SV=1	CAVPO	1
3156	0.88	0.97	12.54	12.54	5.018	tr|H0V9W4|H0V9W4_CAVPO	Uncharacterized protein (Fragment) OS=Cavia porcellus GN=M6PR PE=4 SV=1	CAVPO	1
3157	0.88	1	2.853	2.853	1.712	tr|H0VNT9|H0VNT9_CAVPO	Uncharacterized protein OS=Cavia porcellus GN=SLC9A6 PE=4 SV=1	CAVPO	1
3158	0.88	0.97	2.876	0.5351	0.5351	tr|H0VQ20|H0VQ20_CAVPO	Uncharacterized protein OS=Cavia porcellus GN=RALGAPB PE=4 SV=1	CAVPO	1
3159	0.88	0.96	19.23	9.615	9.615	tr|H0VYD7|H0VYD7_CAVPO	Uncharacterized protein OS=Cavia porcellus GN=COA3 PE=4 SV=1	CAVPO	1
3160	0.87	1.61	7.246	5.007	4.084	tr|H0UVC4|H0UVC4_CAVPO	Uncharacterized protein OS=Cavia porcellus GN=STRN4 PE=4 SV=1	CAVPO	3
3161	0.87	0.97	4.928	3.816	2.385	tr|H0UW79|H0UW79_CAVPO	Uncharacterized protein OS=Cavia porcellus GN=PRPF39 PE=4 SV=1	CAVPO	1
3162	0.87	0.96	13.89	3.945	2.23	tr|H0VAY5|H0VAY5_CAVPO	Uncharacterized protein OS=Cavia porcellus GN=RALGPS2 PE=4 SV=1	CAVPO	1
3163	0.87	0.95	3.182	1.477	1.477	tr|H0VB32|H0VB32_CAVPO	Uncharacterized protein (Fragment) OS=Cavia porcellus GN=DAG1 PE=4 SV=1	CAVPO	1

3164	0.87	1.11	14.62	9.745	3.248	tr|H0VF39|H0VF39_CAVPO	Uncharacterized protein OS=Cavia porcellus GN=GTF2E1 PE=4 SV=1	CAVPO	1
3165	0.87	0.95	5.446	5.446	5.446	tr|H0VLL4|H0VLL4_CAVPO	Uncharacterized protein OS=Cavia porcellus GN=CASP3 PE=3 SV=1	CAVPO	1
3166	0.86	1.86	6.982	4.617	3.153	tr|H0V157|H0V157_CAVPO	Uncharacterized protein OS=Cavia porcellus GN=GRIA3 PE=4 SV=1	CAVPO	2
3167	0.86	0.95	5.91	3.11	1.4	tr|H0VC26|H0VC26_CAVPO	Uncharacterized protein OS=Cavia porcellus GN=GCLC PE=4 SV=1	CAVPO	1
3168	0.85	0.93	1.653	1.653	1.653	tr|H0V5W6|H0V5W6_CAVPO	Uncharacterized protein (Fragment) OS=Cavia porcellus GN=SOAT2 PE=4 SV=1	CAVPO	1
3169	0.85	0.96	13.35	5.908	3.063	tr|H0VWC0|H0VWC0_CAVPO	Uncharacterized protein OS=Cavia porcellus GN=NUP50 PE=4 SV=1	CAVPO	1
3170	0.85	0.93	5.257	2.628	2.628	tr|H0W002|H0W002_CAVPO	Uncharacterized protein OS=Cavia porcellus GN=ELFN1 PE=4 SV=1	CAVPO	1
3171	0.84	0.96	22.39	22.39	13.43	tr|H0V6H5|H0V6H5_CAVPO	Uncharacterized protein OS=Cavia porcellus GN=POLR2L PE=3 SV=1	CAVPO	1
3172	0.84	3.09	9.553	2.881	1.895	tr|H0VCI8|H0VCI8_CAVPO	Uncharacterized protein OS=Cavia porcellus GN=SPAG9 PE=4 SV=1	CAVPO	2
3173	0.84	0.92	2.907	2.907	2.907	tr|H0VGC3|H0VGC3_CAVPO	Uncharacterized protein (Fragment) OS=Cavia porcellus GN=FIZ1 PE=4 SV=1	CAVPO	1
3174	0.84	0.92	8.716	5.963	5.963	tr|H0VYE5|H0VYE5_CAVPO	Uncharacterized protein OS=Cavia porcellus GN=LOC100723309 PE=4 SV=1	CAVPO	1
3175	0.83	0.91	4.276	4.276	4.276	tr|H0UXZ4|H0UXZ4_CAVPO	Uncharacterized protein OS=Cavia porcellus GN=PHF11 PE=4 SV=1	CAVPO	1
3176	0.83	0.92	5.806	2.151	2.151	tr|H0V380|H0V380_CAVPO	Uncharacterized protein OS=Cavia porcellus GN=STK38 PE=4 SV=1	CAVPO	1
3177	0.83	0.91	3.695	1.192	1.192	tr|H0V6F5|H0V6F5_CAVPO	Uncharacterized protein (Fragment) OS=Cavia porcellus GN=ARHGAP12 PE=4 SV=1	CAVPO	1

3178	0.83	0.91	3.008	3.008	3.008	tr|H0VB29|H0VB29_CAVPO	Uncharacterized protein OS=Cavia porcellus GN=Asah1 PE=4 SV=1	CAVPO	1
3179	0.83	0.91	7.805	7.805	7.805	tr|H0VDR6|H0VDR6_CAVPO	Uncharacterized protein OS=Cavia porcellus GN=PPP1R1B PE=4 SV=1	CAVPO	1
3180	0.83	0.94	6.711	6.711	6.711	tr|H0VJQ7|H0VJQ7_CAVPO	Uncharacterized protein OS=Cavia porcellus GN=REM1 PE=4 SV=1	CAVPO	2
3181	0.83	0.95	10.4	8.389	8.389	tr|H0WCV1|H0WCV1_CAVPO	Uncharacterized protein OS=Cavia porcellus GN=TMEM38A PE=4 SV=1	CAVPO	2
3182	0.82	0.89	1.987	1.192	1.192	tr|H0V0A1|H0V0A1_CAVPO	Uncharacterized protein (Fragment) OS=Cavia porcellus GN=SORT1 PE=4 SV=1	CAVPO	1
3183	0.82	0.91	8.225	4.329	4.329	tr|H0V1Z3|H0V1Z3_CAVPO	Uncharacterized protein OS=Cavia porcellus GN=SELRC1 PE=4 SV=1	CAVPO	1
3184	0.82	0.9	4.444	4.444	4.444	tr|H0VD30|H0VD30_CAVPO	Uncharacterized protein OS=Cavia porcellus GN=RPA2 PE=4 SV=1	CAVPO	1
3185	0.82	0.95	2.104	0.4099	0.1913	tr|H0VLT8|H0VLT8_CAVPO	Uncharacterized protein OS=Cavia porcellus GN=SMG1 PE=4 SV=1	CAVPO	1
3186	0.82	0.94	6.061	3.426	1.186	tr|H0W9J9|H0W9J9_CAVPO	Uncharacterized protein OS=Cavia porcellus GN=PRRC2A PE=4 SV=1	CAVPO	1
3187	0.81	0.94	4.833	1.494	1.494	tr|H0UXI7|H0UXI7_CAVPO	Uncharacterized protein (Fragment) OS=Cavia porcellus GN=EHBP1 PE=4 SV=1	CAVPO	2
3188	0.81	0.91	11.15	7.611	2.832	tr|H0V0L6|H0V0L6_CAVPO	Uncharacterized protein (Fragment) OS=Cavia porcellus GN=ASH2L PE=4 SV=1	CAVPO	1
3189	0.81	9.25	17.44	13.86	9.985	tr|H0V0W8|H0V0W8_CAVPO	Uncharacterized protein (Fragment) OS=Cavia porcellus GN=PRKCB PE=4 SV=1	CAVPO	5
3190	0.81	0.89	1.364	1.364	1.364	tr|H0V6F3|H0V6F3_CAVPO	Oxysterol-binding protein OS=Cavia porcellus GN=OSBPL11 PE=3 SV=1	CAVPO	1
3191	0.81	0.91	30.26	4.103	4.103	tr|H0VH74|H0VH74_CAVPO	Uncharacterized protein OS=Cavia porcellus GN=CHP1 PE=4 SV=1	CAVPO	1

3192	0.81	0.89	2.866	1.508	1.508	tr|H0VQ00|H0VQ00_CAVPO	Uncharacterized protein OS=Cavia porcellus GN=CHFR PE=4 SV=1	CAVPO	1
3193	0.81	1.81	19.73	19.73	19.73	tr|H0VRJ6|H0VRJ6_CAVPO	Uncharacterized protein (Fragment) OS=Cavia porcellus GN=UBE2D4 PE=3 SV=1	CAVPO	5
3194	0.8	0.93	4.025	0.3696	0.3696	tr|H0UUM7|H0UUM7_CAVPO	Uncharacterized protein OS=Cavia porcellus GN=MAST4 PE=4 SV=1	CAVPO	1
3195	0.8	0.91	8.738	6.796	3.56	tr|H0V4N7|H0V4N7_CAVPO	Uncharacterized protein OS=Cavia porcellus GN=UPRT PE=4 SV=1	CAVPO	1
3196	0.8	0.88	14.47	14.47	14.47	tr|H0V813|H0V813_CAVPO	Uncharacterized protein OS=Cavia porcellus GN=ZNF706 PE=4 SV=1	CAVPO	1
3197	0.8	0.93	19.76	10.38	2.595	tr|H0VD77|H0VD77_CAVPO	Uncharacterized protein OS=Cavia porcellus GN=DKC1 PE=4 SV=1	CAVPO	1
3198	0.8	0.88	5.128	2.885	2.885	tr|H0VFN7|H0VFN7_CAVPO	Uncharacterized protein OS=Cavia porcellus GN=YIPF3 PE=4 SV=1	CAVPO	1
3199	0.8	0.88	5.854	5.854	5.854	tr|H0WA69|H0WA69_CAVPO	Uncharacterized protein OS=Cavia porcellus GN=DUT PE=4 SV=1	CAVPO	1
3200	0.79	0.93	6.776	1.46	0.7593	tr|H0V7X8|H0V7X8_CAVPO	Uncharacterized protein OS=Cavia porcellus GN=NUP98 PE=4 SV=1	CAVPO	1
3201	0.79	0.94	2.817	1.863	0.4998	tr|H0VZH7|H0VZH7_CAVPO	Uncharacterized protein OS=Cavia porcellus GN=TNC PE=4 SV=1	CAVPO	1
3202	0.79	0.93	12.26	6.452	2.151	tr|H0WAP3|H0WAP3_CAVPO	Uncharacterized protein OS=Cavia porcellus GN=ARHGEF9 PE=4 SV=1	CAVPO	1
