# Supplementary material for: SWATH Based Quantitative Proteomics Reveals Significant Lipid Metabolism in Early Myopic Guinea Pig Retina
Source: Int J Mol Sci. 2021 Apr 29;22(9):4721. doi: 10.3390/ijms22094721 (PMC8124159; doi:10.3390/ijms22094721)
Supplement: Supplementary file 1 [file ijms-22-04721-s001.zip › Table S3.pdf]

Table S3. Peptides and transitions of proteins involved in lipid metabolism using MRM based proteomic approach.

No.	UniProt. accession number	Peptides	Transitions
1	A0A286XCE4	NIFTGLIGPMK	+2b8, +2y7, +2y9
		TYIWQIPER	+2y7, +2y6, +2y5
2	H0VS95	FPYLC[CAM]YK	+2y6, +2y5, +2y4
		FYLYPDLSR	+2y7, +2y6, +2b3
3	A0A286XGK4	EEQLQFLIR	+2y7, +2y5, +2y4
4	H0VSK3	VVVTVEQTEEELER	+2y12, +2y11, +2y9
5	H0WDS3	TFAVTDELVFK	+2y9, +2y7, +2b10
6	A0A286XMC0	TPGEALASFDYIVHEGK	+3b6, +3b11, +3y8
7	H0UU62	TQINPTGTVLFR	+2y10, +2y8, +2y9
8	B5AN23	AITIFQER	+2y6, +2y5, +2y4
		VIPELNGK	+2y6, +2y4, +2y5
		LTGMAFR	+2y3, +2y5, +2y6
