# Supplementary material for: SWATH Based Quantitative Proteomics Reveals Significant Lipid Metabolism in Early Myopic Guinea Pig Retina
Source: Int J Mol Sci. 2021 Apr 29;22(9):4721. doi: 10.3390/ijms22094721 (PMC8124159; doi:10.3390/ijms22094721)
Supplement: Supplementary file 1 [file ijms-22-04721-s001.zip › Tablel S2.pdf]

Table S2. 58 differentially expressed proteins after 4-day LIM in myopic guinea pig retina

No.	Uniprot ID	Protein name	Gene name	Length	Mass	Ratio	LOG 2 (ratio)	P value
1	H0VCR4	Protein ABHD11	ABHD11	315	34,690	1.93	0.95	0.014
2	H0VNU2	Dymeclin	DYM	669	75,927	1.80	0.84	0.023
3	H0VGV7	Tectonin beta-propeller repeat-containing protein 1	TECPR1	1166	129,743	1.76	0.81	0.023
4	H0V4D2	Bardet-Biedl syndrome 5 protein homolog	BBS5	320	36,505	1.51	0.59	0.021
5	H0UYC3	Adenylosuccinate synthetase isozyme 2	ADSS ADSS2	453	49,644	1.50	0.58	0.016
6	H0VGS3	Actin alpha cardiac muscle 1	ACTC1	377	42,019	1.45	0.53	0.003
7	H0VZA3	Dolichol-phosphate mannosyltransferase subunit 3	DPM3	92	10,028	1.42	0.5	0.030
8	H0VPG0	VPS35 endosomal protein sorting factor like	VPS35L	940	106,942	1.34	0.42	0.045
9	H0VS95	Transporter	SLC6A6	620	69,558	1.33	0.41	0.029
10	Q9TEH1	NADH-ubiquinone oxidoreductase chain 1	NADH1 ND1	319	35,846	1.32	0.4	0.043
11	H0WCP2	Oligosaccharyltransferase complex subunit OSTC	OSTC	149	16,829	1.31	0.39	0.006
12	H0VHU4	EPH receptor B2	EPHB2	949	105,886	1.31	0.39	0.016
13	H0VNF6	PRA1 family protein 3	ARL6IP5	188	21,615	1.31	0.39	0.017
14	H0VHC4	Calcineurin like phosphoesterase domain containing 1	CPPED1	238	27,382	1.31	0.39	0.035
15	H0VHE3	Phosphatidate cytidylyltransferase 1	CDS1	461	53,304	1.30	0.38	0.009
16	H0VGB4	1,5-anhydro-D-fructose reductase	AKR1E2	320	36,589	1.28	0.36	0.005
17	H0VSK3	Serine palmitoyltransferase long chain base subunit 1	SPTLC1	461	51,397	1.28	0.36	0.014
18	H0VXL5	Tubulin alpha chain	TUBA1B	451	50,152	1.28	0.35	0.018
19	H0VRA2	Ras homolog family member C	RHOC	193	22,006	1.26	0.34	0.001
20	H0V0E8	Stearoyl-CoA desaturase 5	SCD5	315	35,645	1.26	0.34	0.011
21	H0VRH7	Vesicle transport protein GOT1B	GOLT1B	138	15,426	1.26	0.33	0.022
22	H0WDS3	Coiled-coil domain containing 22	CCDC22	566	63,926	1.25	0.33	0.033
23	H0UXY7	Gamma-aminobutyric acid type A receptor gamma2 subunit	GABRG2	475	55,285	1.25	0.32	0.005
24	H0V025	Methylthioribulose-1-phosphate	APIP	242	27,317	1.24	0.32	0.018

		dehydratase						
25	H0V6H0	Prostaglandin E synthase 2	PTGES2	377	41,943	1.23	0.3	0.032
26	H0V181	3'(2'),5'-bisphosphate nucleotidase 1	BPNT1	308	33,392	1.22	0.29	0.002
27	H0V7H8	Vitamin K epoxide reductase complex subunit 1	VKORC1	163	18,149	1.22	0.29	0.029
28	H0UU62	Alpha-galactosidase	GLA	428	48,843	1.22	0.29	0.032
29	H0WD05	1-acyl-sn-glycerol-3-phosphate acyltransferase alpha	AGPAT1	283	31,717	1.23	0.29	0.041
30	H0V8H2	DEAD-box helicase 19B	DDX19B	464	52,257	1.21	0.28	0.018
31	H0VLQ2	Trafficking protein particle complex 8	TRAPPC8	1318	148,181	1.21	0.28	0.033
32	H0V1X0	Adenosine 3'-phospho 5'-phosphosulfate transporter 1	SLC35B2	432	47,515	1.21	0.28	0.042
33	H0V8U6	Isocitrate dehydrogenase [NAD] subunit, mitochondrial	Idh3g	389	42,418	1.21	0.28	0.049
34	H0V0N9	Single stranded DNA binding protein 1	SSBP1	137	15,978	1.21	0.27	0.004
35	H0WA47	Dehydrogenase/reductase 7	DHRS7	408	45,532	1.20	0.27	0.008
36	H0VJT5	High mobility group 20A	HMG20A	347	40,088	1.21	0.27	0.036
37	H0W436	Transmembrane protein 201	TMEM201	631	68,169	1.21	0.27	0.038
38	H0VPT6	Small nuclear ribonucleoprotein polypeptide F	SNRPF	86	9,725	0.83	-0.27	0.024
39	H0V710	Deleted.	SNCB	Deleted.	Deleted.	0.82	-0.28	0.047
40	H0VLL0	Ubiquitin carboxyl-terminal hydrolase	USP10	780	83,835	0.81	-0.3	0.026
41	H0VHK3	Transformer 2 alpha homolog	TRA2A	282	32,689	0.80	-0.33	0.011
42	H0V6V5	Cytochrome c oxidase assembly protein COX19	COX19	90	10,394	0.79	-0.33	0.032
43	H0VQH8	Phospholipase B-like	PLBD2	585	64,721	0.77	-0.37	0.042
44	H0UZ87	Citrate lyase beta like	CLYBL	343	37,599	0.76	-0.39	0.042
45	H0VAA0	Myosin XVIIIA	MYO18A	2048	232,907	0.75	-0.41	0.028
46	H0UT88	Eukaryotic translation initiation factor 4E-binding protein 2	EIF4EBP2	120	12,939	0.75	-0.42	0.032
47	H0VZE8	Deleted.	LOC100728264	Deleted.	Deleted.	0.73	-0.46	0.027
48	H0V9I8	Fatty acid-binding protein, adipocyte	FABP4	132	14,719	0.72	-0.48	0.002
49	H0V9Q6	Uncharacterized protein	Tpm1	248	28,703	0.72	-0.48	0.034
50	H0VFF6	Secretogranin II	SCG2	550	63,181	0.71	-0.5	0.002
51	H0VXR3	Ribosomal protein L8	RPL8	257	28,025	0.71	-0.5	0.016
52	H0VV40	Steroid receptor RNA activator	SRA1	233	25,431	0.71	-0.5	0.050

		1						
53	H0WDR4	Chromosome 12 open reading frame 57	C12orf57	126	13,196	0.70	-0.52	0.011
54	H0W915	Parvalbumin alpha	PVALB	110	12,165	0.67	-0.59	0.031
55	H0VFA5	Protein phosphatase 1 regulatory subunit 21	PPP1R21	761	86,310	0.64	-0.64	0.013
56	H0UZI9	Protein yippee-like 5	YPEL5	121	13,842	0.62	-0.7	0.026
57	H0UW36	PSME3-interacting protein	Fam192a	254	28,912	0.61	-0.71	0.018
58	H0UWL4	Transmembrane p24 trafficking protein family member 8	TMED8	326	35,935	0.54	-0.9	0.039
